# The present and future of geriatric internal medicine: a bibliometric analysis

**DOI:** 10.3389/fmed.2025.1535189

**Published:** 2025-05-14

**Authors:** Yingjie Chen, Xiuli Dong, Yan Jia

**Affiliations:** ^1^Department of Critical Care Medicine, Shandong Provincial Taishan Hospital, Tai'an, Shandong, China; ^2^School of Clinical and Basic Medical Sciences, Shandong First Medical University and Shandong Academy of Medical Science, Jinan, Shandong, China

**Keywords:** bibliometrics, geriatrics, geriatric internal medicine, internal medicine, VOSviewer

## Abstract

With the gradual progress of global aging, geriatric medicine is becoming increasingly popular, and within geriatric medicine, internal medicine holds a very important position. Understanding the situation in the field of geriatric internal medicine helps researchers gain a comprehensive understanding of this area. In this study, we analyzed and visualized relevant literature from the Web of Science Core Collection database using bibliometric methods, collecting a total of 831 articles, with a time span from 1978 to August 2024. We analyzed the overview of the field, the degree of aging and the volume of publications by country, the core journals in the field, the collaboration relationships among institutions and authors, as well as the hotspots and their changes, and discussed the results. This study provides a broad perspective for professionals in the field of geriatric internal medicine and discusses the future research development in the field.

## 1 Introduction

The global elderly population is increasing both in number and proportion, which is having an impact worldwide. According to data from the United Nations (UN) Population Division, the number of elderly people is likely to exceed 1.5 billion by 2050 (1).

With the gradual increase in the global aging population, geriatric medicine has received more and more attention (2, 3). Geriatrics is a branch of gerontology, encompassing a wide range of research areas related to the elderly, including fundamental theoretical studies, clinical medicine, epidemiology, and social medicine (4). Internal medicine is a specialty within clinical medicine that holds an extremely important position. It is not only the foundation of various clinical medical disciplines but also closely connected to them. The common diagnostic and therapeutic thinking of all clinical medical disciplines is concentrated and expressed in internal medicine (5).

The elderly have a high prevalence of chronic diseases, with 85% of seniors suffering from at least one chronic condition (6). At the same time, elderly individuals have a poor tolerance for many surgeries, the risks of surgery are high, and the benefits obtained are relatively lower compared to younger adults. Therefore, clinical treatment for the elderly holds an important position in internal medicine.

Geriatric internal medicine is an interdisciplinary field that combines internal medicine and geriatric medicine. With the increasing proportion of the elderly population, the research value of geriatric internal medicine is also gradually rising. Therefore, it is necessary to grasp the scientific output and social cooperation in this field. Bibliometric research can help achieve this goal.

Bibliometrics is a discipline that employs mathematical and statistical methods to study literature and information. It can assist researchers in systematically and comprehensively understanding the general state of their research field, identifying research clusters, and pinpointing gaps to be filled (7–9). Bibliometric analysis has become an important tool for measuring scientific output and examining how the intellectual, social, and conceptual structures of related fields evolve over time (10).

In order to conduct better bibliometric research to understand the situation in the field, we referred to the research framework and methodology guidelines (10–12). We chose the classic VOSviewer software and the Bibliometrix package as our analysis tools. The Bibliometrix package is programmed in R, so it can be quickly upgraded and integrated with other statistical R packages. This study selected relevant literature on the theme of geriatric internal medicine, exploring and analyzing the current research status, development process, and relationships in this field, along with visualizations.

## 2 Methods

### 2.1 Data sources and search strategy

Web of Science was selected as the data source for this bibliometric analysis study. On July 27, 2024, we retrieved the publications used in this study from the Web of Science Core Collection. The search strategy is: TS=(geriatric^*^ OR geratolog^*^) AND TS=(internal-medicine) AND LA=(English) AND DT=(Article OR Early Access OR Review) NOT DT=(Proceedings Paper).

**Figure 1:**
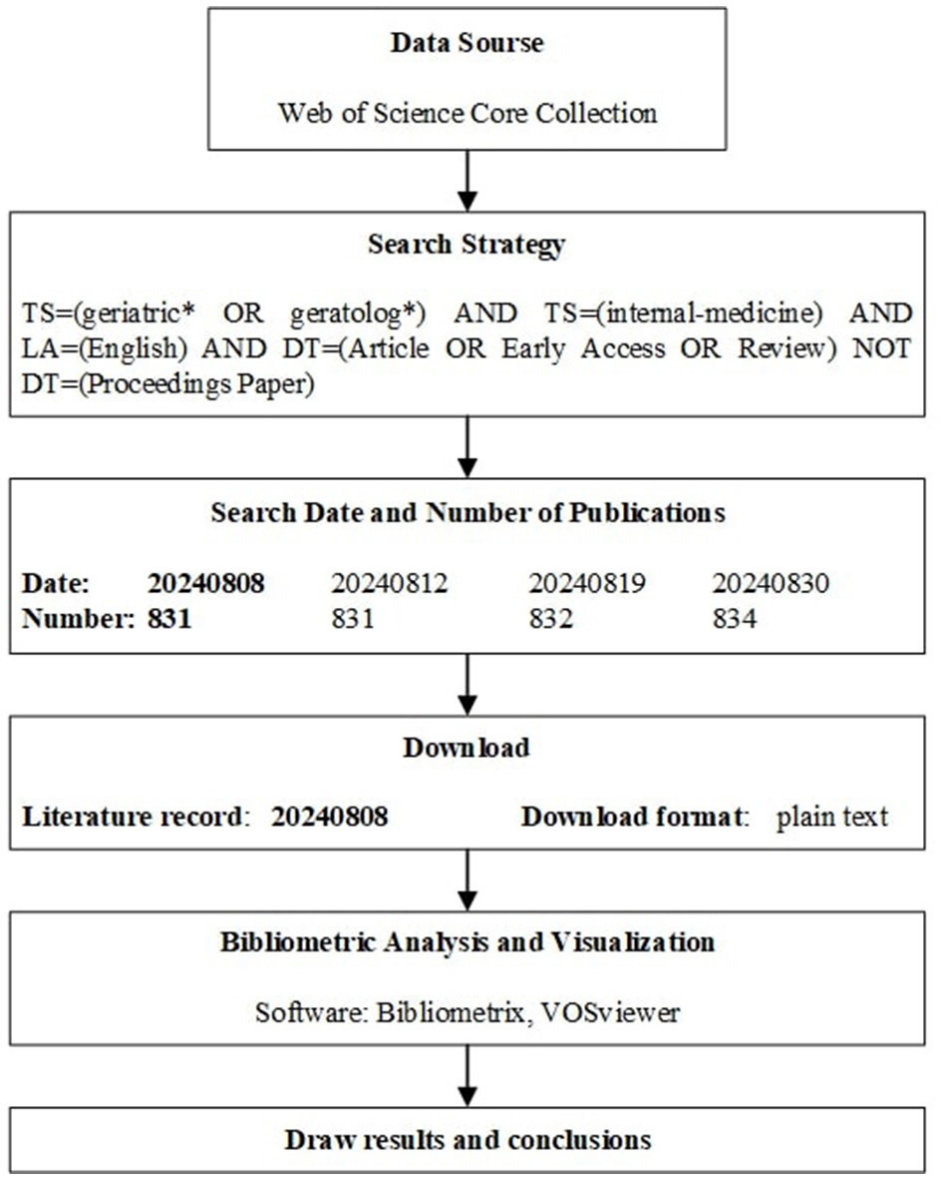
Flow-chart of the study.

The meanings of various symbols in search queries can be referenced in the Search Rules in Web of Science Help (13). We selected documents with the language set to English and the document types as Article or Early Access or Review, excluding Proceedings Papers. The filtering steps have already been reflected in the retrieval strategy. The records were then exported to a plain text file. For the sake of objectivity and reproducibility, we did not conduct any further manual screening of the literature. In order to minimize off-topic papers as much as possible, we repeatedly modified and tested the search strategy before finalizing it. During the analysis process, potential duplicate papers will be deduplicated using the software's DOI-based deduplication feature. In the following month, three more searches were conducted using the same search criteria, with at least a 3-day interval between each, to ensure the stability of the initial search. We urge that other bibliometric studies should also conduct multiple searches and ensure that the differences in search results over time remain within an acceptable range. The specific process is shown in Figure 1.

### 2.2 Analysis and visualization tools

The tools used in this study mainly include the Bibliometrix-4.3.0 package (14) in R-4.4.1 and VOSviewer version 1.6.20 (15). We also used other packages in R to analyze and create tables for the aging population data, and detailed information can be found in the R code file of the Supplementary material.

## 3 Result

### 3.1 Overview of publication status

**Figure 2:**
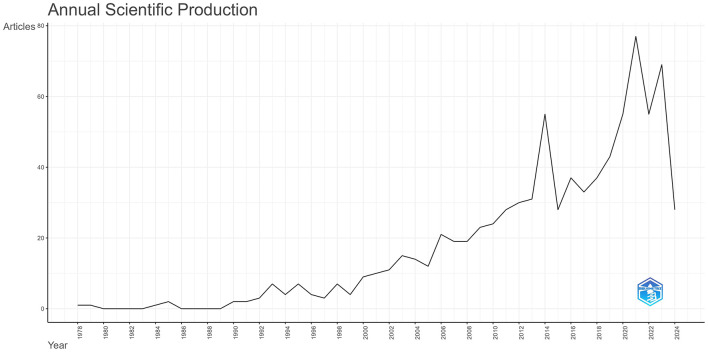
The number of publications is increasing year by year and at an accelerating pace.

This study included 831 publications. The time span is from 1978 to 2024, a total of 46 years, with the data for 2024 only including information up to July 27. Annual Scientific Production is shown in Figure 2. A total of 64 reviews, accounting for 7.70% of the total included (831). The average citations per document is 25.37.

After excluding the literature from 2024, using R for polynomial regression, there is the following relationship between the number of annual publications and the year:


$$\begin{array}{l} \text{Articles }=\;186,618.8\;-\;187.89\;*\text{ Year }+\;0.04729242\\ \;*\text{ Yea}{\text{r}}^{2}\left(\text{Adjusted R }-\text{ squared }=\;0.9204\right) \end{array}$$

According to this trend, it is expected that 66 publications related to geriatrics and internal medicine will be published in 2024, with 28 already published. The Annual Growth Rate for 2023 and prior years is 9.87%.

### 3.2 Analysis of countries/regions and institutions

The corresponding authors had a higher level of involvement in the research conception and paper writing (16). Corresponding authors from 46 countries contributed to 831 publications. Among them, the contributions from the United States and Italy far exceeded those of other countries, with the two countries together accounting for 46.4%, as shown in Table 1.

**Table 1:** Top 15 corresponding author's countries by number of publications.

**Country**	**Articles**	**Articles (%)**	**SCP^a^**	**MCP^b^**	**MCP (%)**
USA	248	29.8	228	20	8.1
Italy	138	16.6	111	27	19.6
Spain	38	4.6	29	9	23.7
Netherlands	37	4.5	33	4	10.8
Israel	32	3.9	32	0	0
Germany	31	3.7	23	8	25.8
China	29	3.5	29	0	0
Switzerland	27	3.2	15	12	44.4
Turkey	26	3.1	25	1	3.8
Canada	23	2.8	17	6	26.1
Australia	20	2.4	11	9	45
France	16	1.9	11	5	31.3
Japan	15	1.8	13	2	13.3
Denmark	14	1.7	13	1	7.1
Sweden	14	1.7	12	2	14.3

^a^SCP, single country publication.

^b^MCP, multiple countries publication.

In order to explore the relationship between the degree of population aging in a country and its published papers in the field of geriatric internal medicine, we downloaded and selected some data from the United Nations (1) and created Table 2 using R. Through the comparison of Tables 1, 2, we can see that countries with high scientific output in the field of geriatric medicine typically also have a higher degree of population aging.

**Table 2:** Population aging degree and ranking of high output countries/areas in geriatric internal medicine research.

**Region, subregion, country or area**	**Percentage of population aged 65+ (%)**	**Ranking (/237)**
Japan	29.56	3
Italy	24.22	7
Germany	22.79	13
United States Virgin Islands	22.24	16
France	21.75	18
China, Hong Kong SAR	21.65	20
Spain	20.65	28
Denmark	20.6	29
Sweden	20.54	31
Netherlands	20.16	34
Switzerland	19.61	40
Canada	19.36	42
China, Taiwan Province of China	18.33	49
United States of America	17.43	55
Australia	17.38	56
China	14.32	78
China, Macao SAR	13.48	82
Israel	12.42	89
Türkiye	10	109

**Figure 3:**
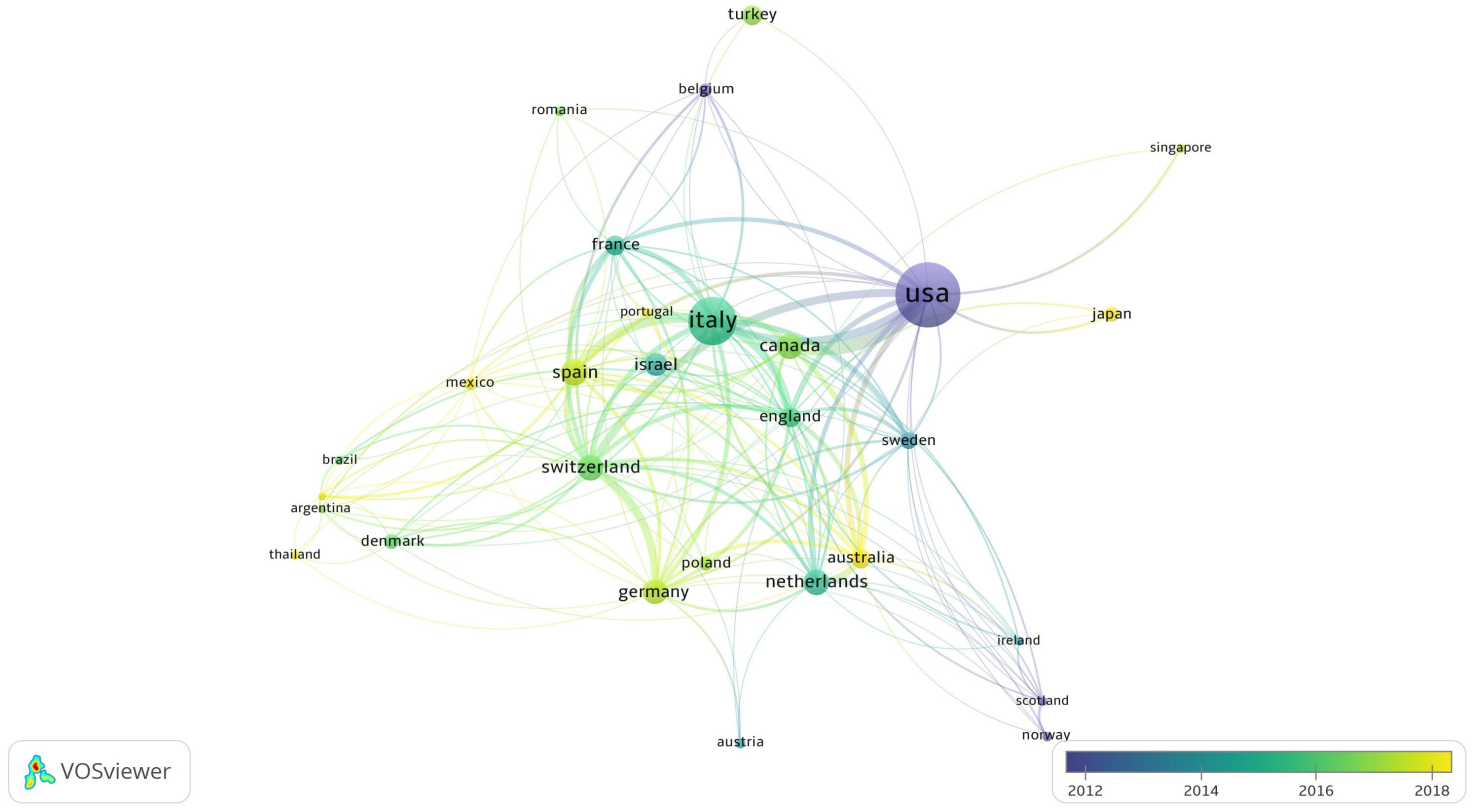
The state of cooperation between countries. The United States has the largest volume of publications, followed by Italy. Countries with serious aging populations, such as Canada, Japan, Singapore, and various European nations, come next in terms of publication volume. The timeline for U.S. publications is earlier. The publications from Japan, Australia, Mexico, Argentina, and Thailand are new.

Subsequently, we used VOSviewer (15) to analyze and visualize the collaboration among various countries. The results show that the cooperation between China, Turkiye, and other countries is not close (not represented in figure), while the cooperation situation of other countries is illustrated in Figure 3. The size of the circle represents the number of documents. The thickness of the lines between each country represents their collaboration strength. In Figure 3, the numerous and thick lines connecting to the United States indicate that it plays an important role in cooperative development in this field, while the cooperation among European countries is also relatively close. The figure also shows that the United States started research in this field relatively early. Countries such as China, Japan, Australia, and Mexico have relatively new research in this field.

**Figure 4:**
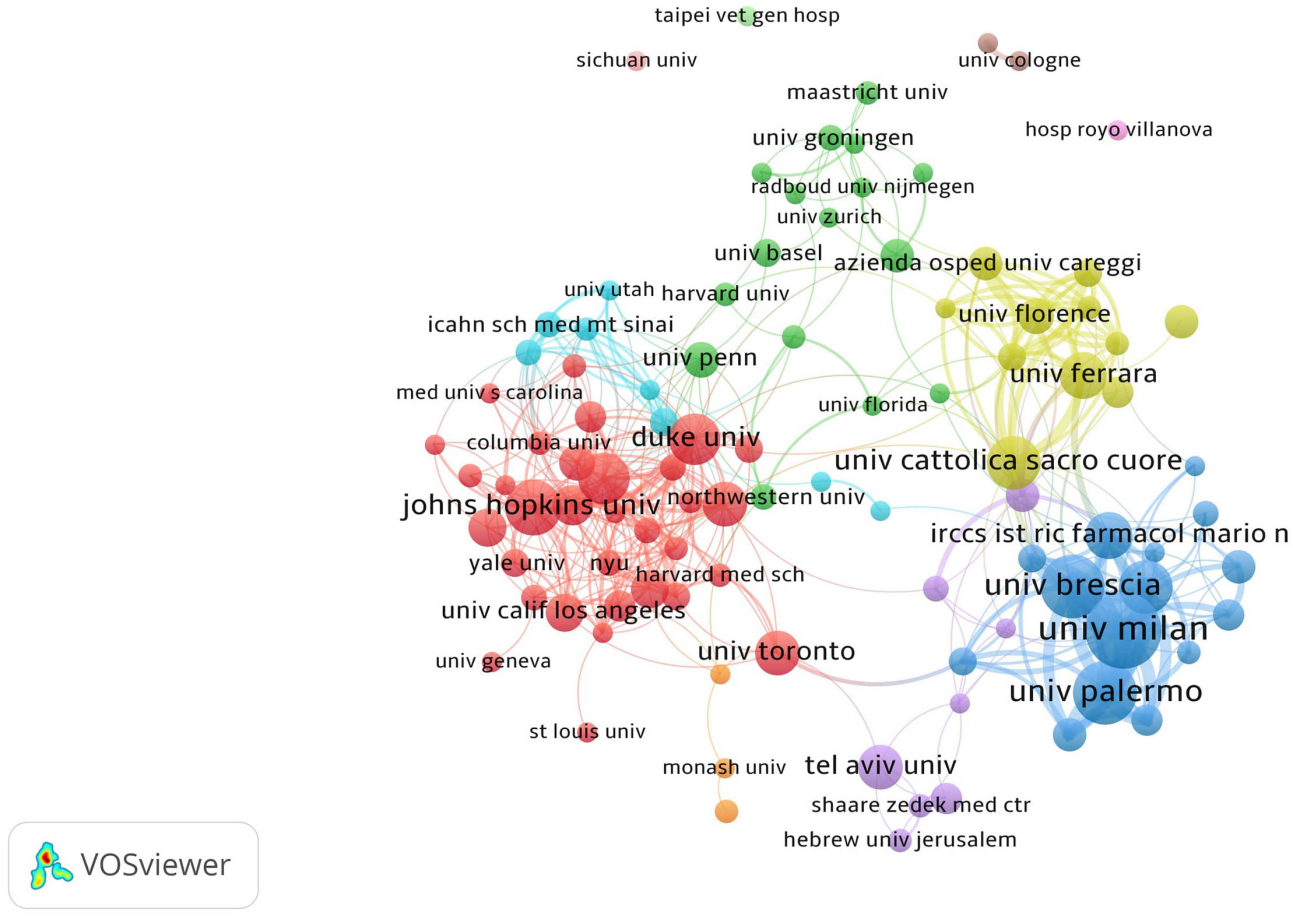
There is close cooperation among multiple clusters of institutions from Europe and the United States.

To explore the contributions of various institutions to geriatric internal medicine, the number of their publications was analyzed. IRCCS Ca Granda Ospedale Maggiore Policlinico ranks first with 109 articles, significantly higher than other institutions, as shown in Table 3: top 10 institutions by number of publications. To further investigate collaboration between institutions, we conducted a co-authorship analysis of publications. The cluster analysis of various institutions is shown in Figure 4, which is divided into 11 clusters. There are 6 clusters with more than three institutions, among which the largest cluster contains 32 institutions. These institutions mainly come from Europe and the United States.

**Table 3:** Top 10 institutions by number of publications.

**Affiliation**	**Articles**
IRCCS Ca Granda Ospedale Maggiore Policlinico	109
Istituto di Ricerche Farmacologiche Mario Negri IRCCS	60
University of Milan	60
US Department of Veterans Affairs	60
Veterans Health Administration (VHA)	59
University of California System	54
University of Florence	50
University of Ferrara	43
University of Toronto	42
Johns Hopkins University	40

### 3.3 Analysis of journals and citations

**Figure 5:**
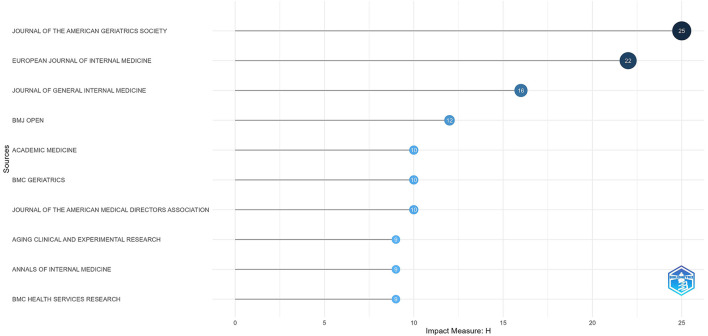
Sources' Local Impact by H index.

Journal analysis helps to identify the core journals in the field (17). Geriatrics and internal medicine are becoming increasingly popular (5, 18). All relevant publications come from 290 journals. Figure 5 shows the top ten journals ranked by H-index, among which four are from Springer Nature, two from Elsevier, and one each from Wiley, BMJ, ACP Journals, and Wolters Kluwer. The Journal of the American Geriatrics Society, European Journal of Internal Medicine, Journal of General Internal Medicine, BMJ Open, BMC Geriatrics, Internal and Emergency Medicine, Aging Clinical and Experimental Research, and Journal of the American Medical Directors Association are core journals in this field. Some regional Journals, such as European Journal of Aging and European Geriatric Medicine, also have a considerable number of publications in the field of geriatric medicine.

### 3.4 Analysis of publications and citations

Highly cited papers can reflect research hotspots to some extent (19). Table 4 shows the top 10 local cited publications. The literature in the table is relatively old, and the vast majority of it pertains to medical education and training in the field of geriatric internal medicine. This indicates that researchers recognized issues related to these areas a long time ago. In the field of geriatric internal medicine, researchers at that time focused on the overall condition and development rather than on specific diseases or particular internal treatments. Studies related to geriatric medical education, training, and policy have been ongoing and have garnered attention (20).

**Table 4:** Top 10 local cited publications.

**Title**	**Year**	**Journal**	**Local citations**	**Global citations**	**Normalized local citations**	**Normalized global citations**	**Summary**
General internal medicine and geriatrics: building a foundation to improve the training of general internists in the care of older adults (34)	2003	Annals of Internal Medicine	17	40	3.86	1.33	Provided some policies and strategies for the construction of internal medicine teams related to geriatric diseases
Improving geriatrics training in internal medicine residency programs: best practices and sustainable solutions (35)	2003	Annals of Internal Medicine	16	57	3.64	1.89	Focused on the training program in geriatrics for internal medicine residents
Knowledge and attitudes about geriatrics of medical students, internal medicine residents, and geriatric medicine fellows (36)	2005	Journal of the American Geriatrics Society	12	99	9.6	3.14	Examined the knowledge and attitudes of medical students, internal medicine residents, and geriatrics researchers regarding geriatrics
Are internal medicine residency programs adequately preparing physicians to care for the baby boomers? A national survey from the association of directors of geriatric academic programs status of geriatrics workforce study (37)	2006	Journal of the American Geriatrics Society	12	38	7	1.04	Introduced the progress in training internal medicine residents in geriatrics
Development of geriatrics-oriented faculty in general internal medicine (38)	2003	Annals of Internal Medicine	11	29	2.5	0.96	Highlighted the development of general internal medicine educators in teaching geriatrics knowledge
Physician career satisfaction across specialties (39)	2002	JAMA Internal Medicine	10	226	5	4.47	Explored the job satisfaction of cross-disciplinary physicians, noting high satisfaction among those in geriatric internal medicine
Risk factors for hospital readmission of elderly patients (40)	2013	European Journal of Internal Medicine	10	69	9.12	2.1	Investigated the risk factors for readmission in elderly patients
Defining aging phenotypes and related outcomes: clues to recognize frailty in hospitalized older patients (41)	2017	The Journals of Gerontology: Series A	10	58	11.79	2.59	Classified the frailty levels of older adults to facilitate graded care
The critical shortage of geriatrics faculty (42)	1993	Journal of the American Geriatrics Society	9	59	7	1.13	Addressed the shortage of geriatrics educators
Effects of an inpatient geriatrics rotation on internal medicine residents? Knowledge and attitudes (43)	1996	Journal of General Internal Medicine	9	33	3.27	1.1	Studied the impact of geriatrics education on residents

However, the ongoing attention means that the education and training issues in geriatric internal medicine urgently need to be addressed (20, 21). We should explore more ways to expand the geriatric medicine workforce, whether by increasing the proportion of geriatric medicine courses for undergraduates or by training internists to learn about geriatric medicine knowledge.

### 3.5 Analysis of authors and co-authorship networks

**Figure 6:**
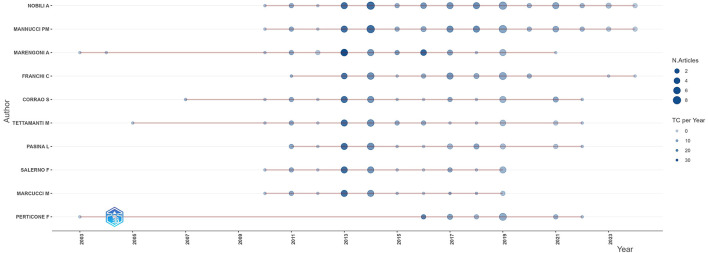
Authors' production over time.

**Figure 7:**
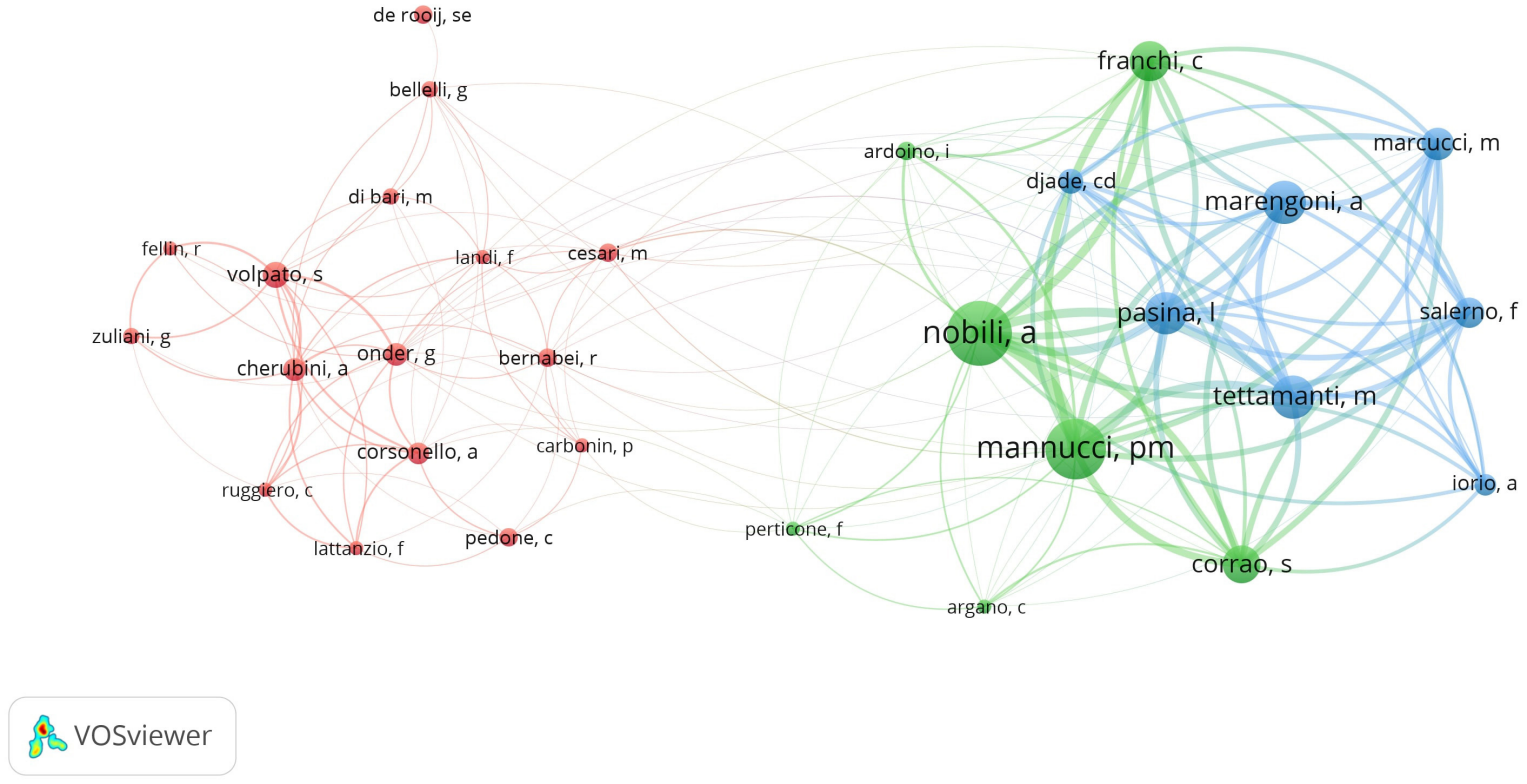
Authors' collaboration network.

A total of 5,636 authors participated in research on geriatric internal medicine, among which Nobili, Alessandro published 55 papers, and Mannucci, Pier Mannuccio published 52 papers, while others did not exceed 35 papers. Table 5 lists the top 10 most locally cited authors. They represent the most influential authors in the field of geriatric medicine. Figure 6 shows the author's scientific output over time. The size of the circles represents the number of published works, while the depth of the color indicates the total citations per year. From the chart, it can be seen that the scientific output of authoritative authors is relatively enduring, and they have shown early and consistent attention to the field of geriatric internal medicine. Figure 7 shows the collaborative relationships among authors in this field. The different colors represent different clusters of authors. The thickness of the lines indicates the strength of the relationships between the authors. There are three main clusters, with the largest cluster (red) containing 16 people, while the other two clusters contain seven people each. The authors in Figure 6 all belong to two groups of seven people. By analyzing the author's annual scientific output and their collaborative relationships, we can conclude that there is a close collaboration among authors who have stable and influential scientific contributions in this field. At the same time, there is another group of authors who collaborate closely within the field and also actively engage with authors outside the cluster.

**Table 5:** Top 10 local cited authors.

**Author**	**Local citations**	**Author**	**Local citations**
Nobili A	133	Mannucci PM	131
Marengoni A	102	Franchi C	92
Corrao S	84	Pasina L	78
Salerno F	78	Marcucci M	77
Tettamanti M	76	Djade CD	42

### 3.6 Co-occurrence of keywords and trend topics

**Figure 8:**
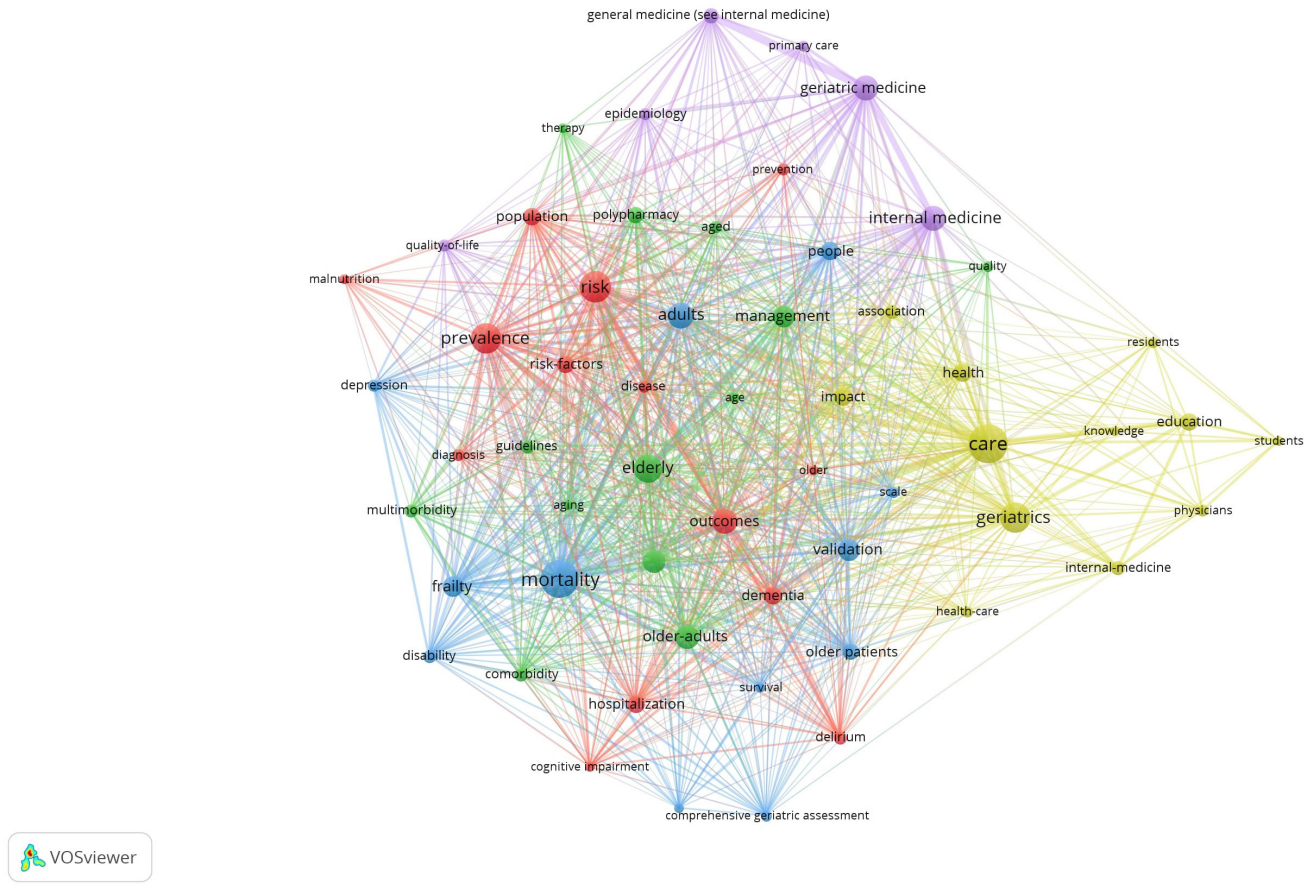
All keywords co-occurrence network.

The analysis and visualization of keywords help to showcase the research hotspots in a field and the changes in those hotspots over time. For the display of hot topics, we used VOSviewer to analyze and visualize keywords (all keywords) that appeared more than 20 times, as shown in Figure 8. The research in geriatric internal medicine mainly focuses on the hotspots listed in Table 6.

**Table 6:** Research hotspots in geriatric internal medicine.

**Clusters**	**Themes**	**Keywords**
Yellow	Health-care	health, care, health-care, residents
	Education	education, knowledge, students
Red	Risk-factors and prevention	risk, risk-factors, prevention
	Outcomes	outcomes, hospitalization
	Prevalence	population, prevalence, diagnosis
	Physical and mental disease	malnutrition, dementia, delirium, cognitive impairment
Blue	Survival and mortality rates	mortality, survival
	Assessment	validation, scale, comprehensive, geriatric assessment
	Physical and mental disease	frailty, disability, depression
Green	Quality control	management, quality
	Morbidity and therapy	multimorbidity, comorbidity, polypharmacy, therapy, guidelines
Purple	Others	primary care, epidemiology, quality-of-life

Researchers have focused on (i) Development, specifically the teaching work related to the training of geriatric internal medicine professionals and quality control; and (ii) Characteristics, the complexity of disease onset and treatment, the importance of healthcare for the elderly, various physical and mental illnesses that are common in older age, and epidemiological characteristics, among others.

**Figure 9:**
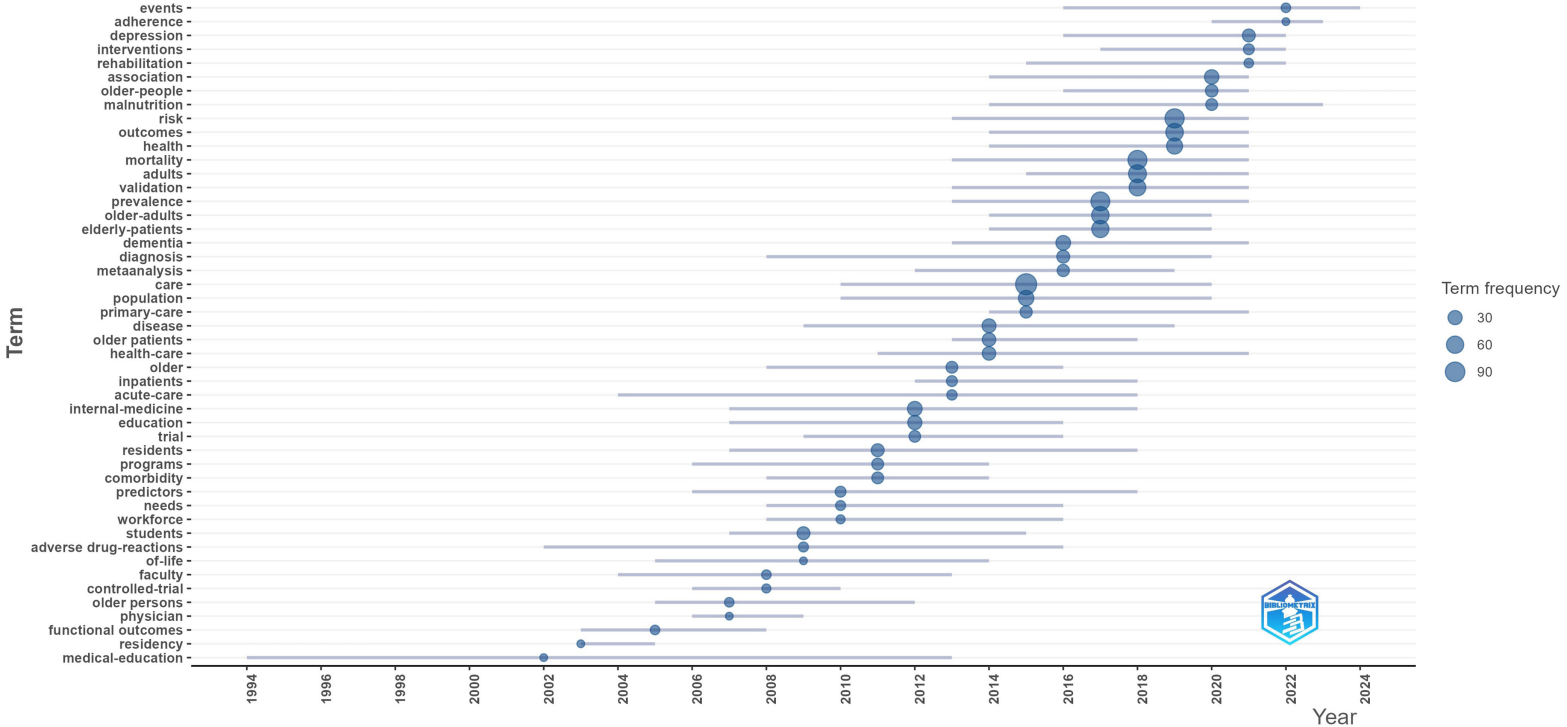
Trend topics over time.

Figure 9 shows the trending topics in geriatric internal medicine. The early hotspots in the field were mainly in education, the mid-term focused on healthcare, and in recent years, the hotspots have shifted to healthcare, healthy lifestyles, mental health, and epidemiological analysis (22). It explains that modern research on health care work has become more in-depth and comprehensive, beginning to pay attention to the mental health issues of the elderly, and that “Integration of healthcare and elderly care.” is gradually developing.

## 4 Discussion

Our research indicates that in the past half century, the development of research related to geriatric internal medicine has accelerated from slow to fast. With the increasing aging population globally, this field is developing rapidly, yet there are still many research gaps and opportunities for growth. Through the analysis of Countries/Regions and Institutions, we can see that countries with a higher and earlier degree of population aging tend to have a greater volume of publications in the field of geriatric medicine. Countries that have recently entered an aging society tend to publish newer papers. Countries around the world have relatively close research cooperation in the field of geriatric internal medicine. Countries that have published more recent papers have relatively close cooperation with various countries, indicating that researchers in this field value collaboration with other countries. Countries with established research foundations provide ample support and assistance to those that have published more recent papers. Secondly, the level of a country's development and its investment in scientific research will also have a certain impact on the volume of publications.

There are many institutions publishing articles in the field of geriatric internal medicine, and the connections between these institutions are close. Most of them are from Europe, North America, and Asian countries, where the aging population is significant, demonstrating the global concern for this field. The journal sources of the articles are diverse, showcasing the vitality and dynamism of this field within the academic community. Currently, the highly cited papers in this field are quite old, and the vast majority of the literature pertains to medical education and training in the field of geriatrics. This indicates that the research community has been paying attention to this field for quite some time and has long been aware of the issue of the lack of talent in the area, attempting to address it. However, it is regrettable that this issue has persisted since the early stages of the field's development, and it has lasted for quite some time, as can be seen from the analysis of trending keywords. In the mid-term of research field, the focus is on health care. Recently, researchers have increased their attention to healthy lifestyles, mental health, and epidemiological analysis (23, 24). In the specific process of topic evolution, the average lifespan of keywords is approximately 8 years, with medical education being an exception. There have also been instances of chasing trends, such as the topic of meta-analysis. The themes can be categorized into three periods based on their evolution over time: early, mid, and recent. The early themes mainly focused on the education of students and doctors, which highlighted the demand for talent in related fields. The mid-term themes were primarily focused on health care. In the gradual transition from the mid-term to the recent period, research on epidemiology became a hotspot. Recently, specific mental illnesses, rehabilitation, family and community, and malnutrition have become focal points, indicating that researchers are gradually taking a more comprehensive approach to this field, with a greater emphasis on a bio-psycho-social perspective in addressing the elderly population.

In the hotpots' theme of Physical and Mental Disease, the keywords include malnutrition, dementia, delirium, cognitive impairment, frailty, disability, and depression. We found that there are four items directly related to neuro-psychiatric conditions, which indicates a high level of attention to neuro-psychiatric diseases when studying internal medicine in the elderly. Next is the focus on nutritional status, which may be due to the high prevalence of these diseases. The long-term torment of chronic diseases, the gradual aging of the nervous system, and the partial or complete loss of the ability to live independently can lead to the emergence of mental illnesses in the elderly and long-term physical depletion. Secondly, in the theme of Morbidity and Therapy, multimorbidity, comorbidity, and polypharmacy are clinically relevant hotspots. In actual clinical practice, doctors weigh the treatment conflicts brought by the coexistence of multiple diseases, consider the combined effects of various medications, and take into account the frailty of the elderly during treatment. This reflects that the research hotspots in geriatric internal medicine not only include various data and indicators but also encompass studies on specific diseases and the difficulties faced by doctors in order to address clinical issues in real life. Research hotspots related to geriatrics encompass various aspects such as talent cultivation, prevention, clinical treatment, and specific diseases. This promotes the comprehensive development of geriatrics. However, researchers should not focus solely on a few specific diseases; they should conduct broader research on different diseases to meet the needs of the elderly population.

In the field of geriatric internal medicine, there is a lack of basic research on specific internal diseases in elderly patients and comprehensive treatment studies for geriatric internal medicine patients (25). Research on the interactions between various diseases and the effects of multiple medications is relatively insufficient (26). This may be due to the complex conditions of elderly patients and their low desire for non-palliative treatments (18, 27, 28). In the study of healthy lifestyles, we can consider examining how life habits before old age impact life after old age, extending the research time span, and providing a foundation for research in areas such as public health, health-care, and extending residents' lifespan (4). Since the COVID-19 pandemic, research on mental health has become a global focus (29). We can increase our investment in the study of end-of-life psychology to provide necessary support when individuals face death and helplessness, guiding and transforming the thoughts of those at the end of life. This can help them gain the confidence to overcome illness, which in turn affects their bodies positively, or to accept reality calmly, maintain a good mindset, seek their own life meaning, and create social value (30). Due to the continuous increase in the global average age of humans, there are more and more elderly patients. Our research in geriatric internal medicine is not only beneficial for elderly patients but also holds significant importance for middle-aged individuals. As the theoretical knowledge of pharmacy, medical devices, and aging continues to develop, the age of elderly individuals will increasingly rise, while the age boundaries for research will become narrower, and the scope of research will gradually expand.

This study also has some limitations. This study only searched the Web of Science Core Collection, and may have overlooked some important literature. No manual screening of the retrieved literature was conducted. Although we repeatedly tested different search strategies before finalizing one, the influence of individual cognitive limitations still exists. Large language models trained on medical data may provide more comprehensive and accurate retrieval strategies and significantly reduce individual workloads (31, 32). This paper only used the R package and VOSviewer for bibliometric research. The algorithmic differences between different software can also introduce errors and ultimately affect the conclusions. Bibliometric analysis methods focus more on analyzing quantifiable indicators but cannot directly assess the academic value of articles. Controversial and critical content, as well as research hotspots, tend to receive more citations and thus attract more attention, while emerging and niche research areas, due to insufficient data accumulation, are less likely to be noticed (33).

## 5 Conclusion

In summary, this study employs bibliometric methods to analyze relevant literature in the field of geriatric internal medicine over approximately half a century. The analysis covers the overall of the field, the correlation between the volume of publications from countries and their aging levels, the collaborative relationships among relevant institutions or authors, the core journals in the field, as well as an analysis of keywords and hotspots, and the evolution of these hotspots over time. Then, the results of the analysis were discussed, highlighting a field that has emerged in the last half-century and is somewhat related to global population aging. There is close collaboration among global institutions and authors. In the early stages, the focus was primarily on talent cultivation, while later stages have shifted to health care, healthy lifestyles, and mental health. It explains that “Integration of healthcare and elderly care.” is gradually being recognized and studied by everyone. This research provides medical personnel and researchers in the field of geriatric internal medicine with knowledge about the current state of the field and discusses its development.
