# Supplementary material for: The present and future of geriatric internal medicine: a bibliometric analysis
Source: Front Med (Lausanne). 2025 May 14;12:1535189. doi: 10.3389/fmed.2025.1535189 (PMC12116460; doi:10.3389/fmed.2025.1535189)
Supplement: Supplementary file 1 [file Data_Sheet_1.docx]

# package -----------------------------------------------------------------

# install.packages("tidyverse")

# install.packages("gt")

# install.packages("xtable")

# install.packages("flextable")

# install.packages("officer")

library(readxl)

library(stargazer)

library(gt)

library(xtable)

library(tidyverse)

library(flextable)

# data ----------------------------------------------------------------

#Data download link:

# https://population.un.org/wpp/Download/Files/1_Indicator%20(Standard)/EXCEL_FILES/2_Population/WPP2024_POP_F06_1_POPULATION_PERCENTAGE_SELECT_AGE_GROUPS_BOTH_SEXES.xlsx

# read data ---------------------------------------------------------------

df <- read_xlsx("WPP2024_POP_F06_1_POPULATION_PERCENTAGE_SELECT_AGE_GROUPS_BOTH_SEXES.xlsx",

          sheet = 1,

          skip = 16,

          col_names = T)

# select ------------------------------------------------------------------

# age == 65+

# year == 2023

#type == Country/Area

dfselect <- df |> filter(df$Year == 2023, df$Type == "Country/Area") |>

  select(`Region, subregion, country or area *`, `65+`)

# data type transfer

dfselect$`65+` <- as.numeric(dfselect$`65+`)

# rank

dfselectrank <- dfselect |>arrange(desc(`65+`)) |>

  mutate(Rank = row_number())

#filter

filter <-  dfselectrank |>

  filter(`Region, subregion, country or area *` %in% c("United States of America",

                                                       "United States Virgin Islands",

                                                       "Italy",

                                                       "Spain",

                                                       "Netherlands",

                                                       "Israel",

                                                       "Germany",

                                                       "China",

                                                       "China, Hong Kong SAR",

                                                       "China, Macao SAR",

                                                       "China, Taiwan Province of China",

                                                       "Switzerland",

                                                       "Türkiye",

                                                       "Canada",

                                                       "France",

                                                       "Australia",

                                                       "Japan",

                                                       "Sweden",

                                                       "Denmark"))

#keep 2 digit

filter$`65+` <- round(filter$`65+`, 2)

#rename

colnames(filter) <- c("Region, Subregion, Country or Area", "Percentage of population aged 65+ (%)", "Ranking (/237)")

#plot table

filter |> gt()

#to latex

?stargazer

stargazer(filter, type = "latex",

          summary = F, title = "Population Aging Degree and Ranking of High Output Countries/Areas in Geriatric Internal Medicine Research")

#print table1 to office word

library(officer)

table1 <- flextable(filter, cwidth = 2)

table1

? flextable

doc <- read_docx()

caption <- block_caption("Table2 Population Aging Degree and Ranking of High Output Countries/Areas in Geriatric Internal Medicine Research")

doc <- body_add_caption(doc,caption, pos = "on")

doc <- body_add_flextable(doc, table1,topcaption = T)

print(doc, "table2.docx")

# lm ----------------------------------------------------------------------

#read data

lmdf <- readxl::read_xlsx("Annual_Production_bibliometrix_2024-08-13.xlsx",

                          sheet = 1,

                          skip = 1,

                          col_names = T)

#not 2024

str(lmdf)

lmdf <-lmdf[1:46,]

fit <- lm(Articles ~ Year + I(Year^2), data = lmdf)

summary(fit)

plot(lmdf$Year, lmdf$Articles,

     xlab = "Year",

     ylab = "Articles")

lines(lmdf$Year, fitted(fit))

# Articles = 186618.8 - 187.89*Year + 0.04729242*Year^2

# analyse -----------------------------------------------------------------

# install.packages("bibliometrix")

# install.packages("bibliometrix", dependencies=TRUE)

#your htmltools edition may old, use this :

# remove.packages("htmltools")

# install.packages("htmltools")

#!!!!!!!!!

#open bibliometrix in html

library(bibliometrix)

biblioshiny()
