# Supplementary material for: The present and future of geriatric internal medicine: a bibliometric analysis
Source: Front Med (Lausanne). 2025 May 14;12:1535189. doi: 10.3389/fmed.2025.1535189 (PMC12116460; doi:10.3389/fmed.2025.1535189)
Supplement: Supplementary file 2 [file Data_Sheet_2.docx]

FN Clarivate Analytics Web of Science

VR 1.0

PT J

AU Tufan, F

Yuruyen, M

Kizilarslanoglu, MC

Akpinar, T

Emiksiye, S

Yesil, Y

Ozturk, ZA

Bozbulut, UB

Bolayir, B

Tasar, PT

Yavuzer, H

Sahin, S

Ulger, Z

Ozturk, GB

Halil, M

Akcicek, F

Doventas, A

Kepekci, Y

Ince, N

Karan, MA

AF Tufan, Fatih

Yuruyen, Mehmet

Kizilarslanoglu, Muhammet Cemal

Akpinar, Timur

Emiksiye, Sirhan

Yesil, Yusuf

Ozturk, Zeynel Abidin

Bozbulut, Utku Burak

Bolayir, Basak

Tasar, Pinar Tosun

Yavuzer, Hakan

Sahin, Sevnaz

Ulger, Zekeriya

Ozturk, Gulistan Bahat

Halil, Meltem

Akcicek, Fehmi

Doventas, Alper

Kepekci, Yalcin

Ince, Nurhan

Karan, Mehmet Akif

TI Geriatrics education is associated with positive attitudes toward older

people in internal medicine residents: A multicenter study

SO ARCHIVES OF GERONTOLOGY AND GERIATRICS

LA English

DT Article

DE Geriatrics education; Attitudes toward older people; Internal medicine

residents

ID STUDENTS ATTITUDES; KNOWLEDGE; DOCTORS; FELLOWS; ADULTS; SCALE

AB Objective: The number of older people is growing fast in Turkey. In this context, internal medicine residents and specialists contact older people more frequently. Thus, healthcare providers' knowledge and attitudes toward older people is becoming more important. Studies that specifically investigate internal medicine residents' attitudes toward the elderly are scarce. We aimed to investigate the attitudes of internal medicine residents toward older people.

Methods: This cross-sectional multicenter study was undertaken in the internal medicine clinics of six university state hospitals that provide education in geriatric care. All internal medicine residents working in these hospitals were invited to participate in this questionnaire study between March 2013 and December 2013. We recorded the participants' age, sex, duration of internal medicine residency, existence of relatives older than 65 years, history of geriatrics course in medical school, geriatrics rotation in internal medicine residency, and nursing home visits.

Results: A total of 274 (82.3%) of the residents participated in this study, and 83.6% of them had positive attitudes toward older people. A geriatrics rotation during internal medicine residency was the only independent factor associated with positive attitudes toward the elderly in this multivariate analysis. A geriatrics course during medical school was associated with positive attitudes in the univariate analysis, but only tended to be so in the multivariate analysis.

Conclusion: Geriatrics rotation during internal medicine residency was independently associated with positive attitudes toward older people. Generalization of geriatrics education in developing countries may translate into a better understanding and improved care for older patients. (C) 2015 Elsevier Ireland Ltd. All rights reserved.

C1 [Tufan, Fatih; Akpinar, Timur; Emiksiye, Sirhan; Ozturk, Gulistan Bahat; Karan, Mehmet Akif] Istanbul Univ, Istanbul Sch Med, Dept Internal Med, Div Geriatr, TR-34303 Istanbul, Turkey.

[Yuruyen, Mehmet; Yavuzer, Hakan; Doventas, Alper] Istanbul Univ, Cerrahpasa Med Sch, Dept Internal Med, Div Geriatr, TR-34303 Istanbul, Turkey.

[Kizilarslanoglu, Muhammet Cemal; Bozbulut, Utku Burak; Ulger, Zekeriya] Gazi Univ, Sch Med, Dept Internal Med, Div Geriatr, Ankara, Turkey.

[Yesil, Yusuf; Bolayir, Basak; Halil, Meltem] Hacettepe Univ, Sch Med, Dept Internal Med, Geriatr Unit, Ankara, Turkey.

[Ozturk, Zeynel Abidin; Kepekci, Yalcin] Gaziantep Univ, Sch Med, Dept Internal Med, Div Geriatr, Gaziantep, Turkey.

[Tasar, Pinar Tosun; Sahin, Sevnaz; Akcicek, Fehmi] Ege Univ, Sch Med, Dept Internal Med, Div Geriatr, Izmir, Turkey.

[Ince, Nurhan] Istanbul Univ, Istanbul Sch Med, Dept Publ Hlth, Istanbul, Turkey.

C3 Istanbul University; Istanbul University; Istanbul University -

Cerrahpasa; Gazi University; Hacettepe University; Gaziantep University;

Ege University; Istanbul University

RP Yuruyen, M (corresponding author), Istanbul Univ, Cerrahpasa Med Sch, Dept Internal Med, Div Geriatr, TR-34303 Istanbul, Turkey.

EM drwalker@mynet.com

RI HALIL, MELTEM GULHAN/I-9038-2013; Halil, Meltem/AAY-7677-2021; Akpinar,

Timur Selcuk/GQA-7780-2022; KARAN, Mehmet Akif/AAD-5304-2020;

Amikishiyev, Shirkhan/AAU-3713-2020; Tufan, Fatih/CAF-4137-2022;

Ã–ztÃ¼rk, Abidin/AAG-7857-2020; ince, nurhan/AAV-8801-2021; Doventas,

Alper/AAB-3966-2020; Şahin, Sevnaz/ISU-9128-2023; Doventas,

Alper/A-5005-2017; Amikishiyev, Shirkhan/ABA-9619-2021; YAVUZER,

HAKAN/Y-5492-2018

OI Amikishiyev, Shirkhan/0000-0002-8355-0176; Doventas,

Alper/0000-0001-5509-2625; Doventas, Alper/0000-0001-5509-2625;

Amikishiyev, Shirkhan/0000-0002-8355-0176; Halil, Meltem

Gulhan/0000-0001-7597-8140; Kizilarslanoglu, Muhammet

Cemal/0000-0002-7632-6811; YAVUZER, HAKAN/0000-0003-2685-6555

CR Ahmed NN, 2011, J AM GERIATR SOC, V59, P143, DOI 10.1111/j.1532-5415.2010.03235.x

Cankurtaran M, 2006, J NATL MED ASSOC, V98, P1518

Chua MPW, 2008, ANN ACAD MED SINGAP, V37, P947

Hobbs C, 2006, AUST J PHYSIOTHER, V52, P115, DOI 10.1016/S0004-9514(06)70046-0

Hweidi IM, 2005, INT NURS REV, V52, P225, DOI 10.1111/j.1466-7657.2005.00434.x

Kaempfer D, 2002, J AM DIET ASSOC, V102, P197, DOI 10.1016/S0002-8223(02)90048-9

Kearney N, 2000, ANN ONCOL, V11, P599, DOI 10.1023/A:1008327129699

Kishimoto M, 2005, J AM GERIATR SOC, V53, P99, DOI 10.1111/j.1532-5415.2005.53018.x

Koder DA, 2008, AUSTRALAS J AGEING, V27, P212, DOI 10.1111/j.1741-6612.2008.00314.x

Lee M, 2005, J AM GERIATR SOC, V53, P489, DOI 10.1111/j.1532-5415.2005.53170.x

Leung S, 2011, INTERN MED J, V41, P308, DOI 10.1111/j.1445-5994.2009.02140.x

Oakley R, 2014, AGE AGEING, V43, P442, DOI 10.1093/ageing/afu077

REUBEN DB, 1995, J AM GERIATR SOC, V43, P1430, DOI 10.1111/j.1532-5415.1995.tb06626.x

Reuben DB, 1998, J AM GERIATR SOC, V46, P1425, DOI 10.1111/j.1532-5415.1998.tb06012.x

Sahin S, 2012, ARCH GERONTOL GERIAT, V55, P205, DOI 10.1016/j.archger.2011.08.015

Samra R, 2013, J AM GERIATR SOC, V61, P1188, DOI 10.1111/jgs.12312

Usta YY, 2012, ARCH GERONTOL GERIAT, V54, P90, DOI 10.1016/j.archger.2011.02.002

NR 17

TC 12

Z9 12

U1 1

U2 30

PU ELSEVIER IRELAND LTD

PI CLARE

PA ELSEVIER HOUSE, BROOKVALE PLAZA, EAST PARK SHANNON, CO, CLARE, 00000,

IRELAND

SN 0167-4943

EI 1872-6976

J9 ARCH GERONTOL GERIAT

JI Arch. Gerontol. Geriatr.

PD MAR-APR

PY 2015

VL 60

IS 2

BP 307

EP 310

DI 10.1016/j.archger.2014.12.004

PG 4

WC Geriatrics & Gerontology

WE Science Citation Index Expanded (SCI-EXPANDED); Social Science Citation Index (SSCI)

SC Geriatrics & Gerontology

GA CB5VF

UT WOS:000349695000012

PM 25532778

DA 2024-08-08

ER

PT J

AU Simon, SR

Fabiny, AR

Kotch, J

AF Simon, SR

Fabiny, AR

Kotch, J

TI Geriatrics training in general internal medicine fellowship programs:

Current practice, barriers, and strategies for improvement

SO ANNALS OF INTERNAL MEDICINE

LA English

DT Article

ID INTEGRATING GERIATRICS; CARE; CURRICULUM; CARDIOLOGY; SOCIETY; DISEASE;

CANCER

AB To ensure its growth and prosperity, general internal medicine will need to embrace care of the elderly, research on aging, and geriatrics education as components of its core mission. Experts agree that general internal medicine fellows could benefit from increased opportunities in research on aging and geriatrics education; however, important barriers will hamper efforts to integrate geriatrics training into general internal medicine fellowship programs.

This article reviews the barriers to integration and proposes solutions for overcoming those barriers. As a result of interviews and meetings with a broad representation of general internists, geriatricians, funding agencies, and policymakers, we propose 2 interventions: 1) the development of institutional program grants to foster collaboration between general internal medicine and geriatrics faculty in the training of general internal medicine fellows and 2) the creation of a 3-year fellowship program combining general internal medicine and geriatrics.

This article discusses the importance of evaluating these and other programs intended to increase the geriatrics experience of general internal medicine fellows, and it describes the potential implications of these changes for a broad array of stakeholder institutions.

C1 Harvard Univ, Sch Med, Dept Ambulatory Care & Prevent, Harvard Pilgrim Hlth Care, Boston, MA 02215 USA.

Hebrew Rehabil Ctr Aged, Boston, MA 02131 USA.

C3 Harvard University; Harvard Medical School; Harvard Pilgrim Health Care

RP Harvard Univ, Sch Med, Dept Ambulatory Care & Prevent, Harvard Pilgrim Hlth Care, 133 Brookline Ave,6th Floor, Boston, MA 02215 USA.

EM steven_simon@hms.harvard.edu

CR *AM SOC CLIN ONC, GER ONC TRAIN PROGR

Applegate WB, 1997, J AM GERIATR SOC, V45, P641, DOI 10.1111/j.1532-5415.1997.tb03103.x

Beasley BW, 1998, J GEN INTERN MED, V13, P426, DOI 10.1046/j.1525-1497.1998.00127.x

BLOCK SD, 1994, NEW PATHWAYS MED ED

Caiola E, 2000, J GEN INTERN MED, V15, P656, DOI 10.1046/j.1525-1497.2000.06389.x

CALKINS E, 1993, T AM CLIN CLIMAT ASS, V105, P78

COHEN JJ, 1993, ANN INTERN MED, V119, P1125, DOI 10.7326/0003-4819-119-11-199312010-00010

Hafferty FW, 1998, ACAD MED, V73, P403, DOI 10.1097/00001888-199804000-00013

Kennedy BJ, 1997, CANCER, V80, P1270, DOI 10.1002/(SICI)1097-0142(19971001)80:7<1270::AID-CNCR12>3.0.CO;2-4

Larson EB, 2001, ANN INTERN MED, V134, P997, DOI 10.7326/0003-4819-134-10-200105150-00013

LUDMERER KM, 2000, ED MED STUD 10 SOTR

MCCORMICK WC, 1994, J AM GERIATR SOC, V42, P517, DOI 10.1111/j.1532-5415.1994.tb04974.x

Mebane EW, 1999, J AM GERIATR SOC, V47, P579, DOI 10.1111/j.1532-5415.1999.tb02573.x

ORLANDER JD, 1991, J GEN INTERN MED, V6, P460, DOI 10.1007/BF02598172

Pantilat SZ, 1999, WESTERN J MED, V171, P253

Pepper PV, 1999, J AM GERIATR SOC, V47, P967, DOI 10.1111/j.1532-5415.1999.tb01292.x

Rich NW, 1998, J AM GERIATR SOC, V46, P921, DOI 10.1111/j.1532-5415.1998.tb02733.x

Simon SR, 1999, ACAD MED, V74, P1253, DOI 10.1097/00001888-199911000-00022

*SOC GEN INT MED, 1996, DIR GEN INT MED FELL

Society of General Internal Medicine, 1994, J GEN INTERN MED, V9, P513

Sox HC, 2001, AM J MED, V110, P745, DOI 10.1016/S0002-9343(01)00756-2

Tishler J, 2000, J AM GERIATR SOC, V48, P961, DOI 10.1111/j.1532-5415.2000.tb06895.x

TURNBERG LA, 1994, J ROY COLL PHYS LOND, V28, P194

NR 23

TC 13

Z9 13

U1 0

U2 0

PU AMER COLL PHYSICIANS

PI PHILADELPHIA

PA INDEPENDENCE MALL WEST 6TH AND RACE ST, PHILADELPHIA, PA 19106-1572 USA

SN 0003-4819

EI 1539-3704

J9 ANN INTERN MED

JI Ann. Intern. Med.

PD OCT 7

PY 2003

VL 139

IS 7

BP 621

EP 627

DI 10.7326/0003-4819-139-7-200310070-00036

PG 7

WC Medicine, General & Internal

WE Science Citation Index Expanded (SCI-EXPANDED)

SC General & Internal Medicine

GA 729AP

UT WOS:000185748800028

PM 14530246

DA 2024-08-08

ER

PT J

AU Tzur, I

Izhakian, S

Gorelik, O

AF Tzur, Irma

Izhakian, Shimon

Gorelik, Oleg

TI Orthostatic hypotension in internal medicine wards

SO CURRENT MEDICAL RESEARCH AND OPINION

LA English

DT Review

DE Orthostatic hypotension; Hospitalization; Internal medicine; Elderly

ID BLOOD-PRESSURE; POSTURAL HYPOTENSION; ATHEROSCLEROSIS RISK;

HEART-FAILURE; LOWER-LIMB; CARDIOVASCULAR-DISEASE; PREDICTS MORTALITY;

OLDER-ADULTS; PREVALENCE; COMMON

AB Objectives: Most studies of orthostatic hypotension (OH) have focused on community-dwelling and institutionalized patient populations. Less is known about OH in hospitalized patients. Moreover, a comprehensive review of OH in internal medicine wards has not been published in the English literature. Our purpose is to provide current information regarding OH in internal medicine inpatients. Methods: A comprehensive search of medical databases was performed for potentially relevant articles, using the following keywords: postural or orthostatic hypotension, with the combination of hospitalization or internal medicine. Inclusion criteria were: population of patients hospitalized for acute disorders in internal medicine or geriatric wards with a sample size of >= 50 and publication as an original full-length article in the English language. Data from 14 selected studies are reviewed, including: pathophysiology, evaluation, prevalence, manifestations, risk factors, prognosis, and management. Results: OH is a common and often symptomatic disorder in elderly internal medicine patients. The prevalence of OH in this population ranges from 22-75%. There are substantial discrepancies between the studies reviewed regarding definitions and means of evaluating OH. OH in internal medicine wards is largely non-neurogenic and multifactorial. The main predisposing factors for OH are prolonged bed rest, hypertension, and heart failure. OH in internal medicine wards is managed mainly with non-pharmacologic interventions, and is frequently reversible. Conclusions: In internal medicine inpatients, OH warrants attention because this disorder is common, potentially dangerous, and treatable. In the hospital setting, OH should be routinely assessed on ambulation, following the current guidelines for OH definition and meaning.

C1 [Tzur, Irma; Izhakian, Shimon; Gorelik, Oleg] Assaf Harofeh Med Ctr, Dept Internal Med F, IL-70300 Zerifin, Israel.

[Tzur, Irma; Izhakian, Shimon; Gorelik, Oleg] Tel Aviv Univ, Sackler Fac Med, Ramat Aviv, Israel.

C3 Tel Aviv University; Shamir Medical Center (Assaf Harofeh); Tel Aviv

University

RP Gorelik, O (corresponding author), Assaf Harofeh Med Ctr, Dept Internal Med F, IL-70300 Zerifin, Israel.

EM internal6@asaf.health.gov.il

CR Arbogast SD, 2009, AM J MED, V122, P574, DOI 10.1016/j.amjmed.2008.10.040

Belmin J, 2000, J GERONTOL A-BIOL, V55, pM667, DOI 10.1093/gerona/55.11.M667

Biaggioni I, 2018, AM J HYPERTENS, V31, P1255, DOI 10.1093/ajh/hpy089

Biaggioni I, 2014, CURR CARDIOL REP, V16, DOI 10.1007/s11886-014-0542-z

Brignole M, 2018, KARDIOL POL, V76, P1119, DOI 10.5603/KP.2018.0161

Brignole M, 2018, EUR HEART J, V39, P1883, DOI 10.1093/eurheartj/ehy037

Chou RH, 2015, INT J CARDIOL, V195, P40, DOI 10.1016/j.ijcard.2015.05.060

Cohen N, 2003, CLIN AUTON RES, V13, P447, DOI 10.1007/s10286-003-0119-1

Eigenbrodt ML, 2000, STROKE, V31, P2307, DOI 10.1161/01.STR.31.10.2307

Fanciulli A, 2018, CLIN AUTON RES, V28, P355, DOI 10.1007/s10286-018-0529-8

Fedorowski A, 2013, J INTERN MED, V273, P322, DOI 10.1111/joim.12021

Fedorowski A, 2010, J INTERN MED, V268, P383, DOI 10.1111/j.1365-2796.2010.02261.x

Fedorowski A, 2010, EUR HEART J, V31, P85, DOI 10.1093/eurheartj/ehp329

Fedorowski A, 2009, J HYPERTENS, V27, P976, DOI 10.1097/HJH.0b013e3283279860

Feldstein C, 2012, J AM SOC HYPERTENS, V6, P27, DOI 10.1016/j.jash.2011.08.008

Finucane C, 2017, J AM GERIATR SOC, V65, P474, DOI 10.1111/jgs.14563

Finucane C, 2014, CIRCULATION, V130, P1780, DOI 10.1161/CIRCULATIONAHA.114.009831

Fortherby M, 1997, BLOOD PRESSURE, V6, P343

Franceschini N, 2010, HYPERTENSION, V56, P1054, DOI 10.1161/HYPERTENSIONAHA.110.156380

Freeman R, 2018, J AM COLL CARDIOL, V72, P1294, DOI 10.1016/j.jacc.2018.05.079

Freeman R, 2011, AUTON NEUROSCI-BASIC, V161, P46, DOI 10.1016/j.autneu.2011.02.004

Gorelik O, 2005, BLOOD PRESSURE, V14, P139, DOI 10.1080/08037050510008968

Gorelik O, 2004, CARDIOLOGY, V102, P177, DOI 10.1159/000081007

Gorelik O, 2016, HEART FAIL REV, V21, P529, DOI 10.1007/s10741-016-9541-z

Gorelik O, 2015, J AM SOC HYPERTENS, V9, P985, DOI 10.1016/j.jash.2015.10.001

Gorelik O, 2014, BLOOD PRESSURE, V23, P248, DOI 10.3109/08037051.2013.871787

Gorelik O, 2009, GERONTOLOGY, V55, P138, DOI 10.1159/000141920

Guérin A, 2016, AGING CLIN EXP RES, V28, P513, DOI 10.1007/s40520-015-0451-z

Hamrefors V, 2016, PLOS ONE, V11, DOI 10.1371/journal.pone.0154249

HOLLISTER AS, 1992, WESTERN J MED, V157, P652

KUBO SH, 1983, AM J CARDIOL, V52, P512, DOI 10.1016/0002-9149(83)90017-6

LATHERS CM, 1994, J CLIN PHARMACOL, V34, P403, DOI 10.1002/j.1552-4604.1994.tb04980.x

LIPSITZ LA, 1989, NEW ENGL J MED, V321, P952

Masaki KH, 1998, CIRCULATION, V98, P2290, DOI 10.1161/01.CIR.98.21.2290

Naschitz JE, 2007, POSTGRAD MED J, V83, DOI 10.1136/pgmj.2007.058198

Norcliffe-Kaufmann L, 2014, CLIN AUTON RES, V24, P189, DOI 10.1007/s10286-014-0229-y

O'Riordan Shelagh, 2017, Nurs Older People, V29, P20, DOI 10.7748/nop.2017.e961

Palma JA, 2015, MOVEMENT DISORD, V30, P639, DOI 10.1002/mds.26079

Panayiotou B, 1999, POSTGRAD MED J, V75, P213, DOI 10.1136/pgmj.75.882.213

Podoleanu C, 2006, J AM COLL CARDIOL, V48, P1425, DOI 10.1016/j.jacc.2006.06.052

Potocka-Plazak K, 2001, AGING CLIN EXP RES, V13, P378, DOI 10.1007/BF03351506

Puisieux F, 1999, J AM GERIATR SOC, V47, P1332, DOI 10.1111/j.1532-5415.1999.tb07434.x

RAIHA I, 1995, ARCH INTERN MED, V155, P930, DOI 10.1001/archinte.155.9.930

Ricci F, 2017, J HYPERTENS, V35, P776, DOI 10.1097/HJH.0000000000001215

Ricci F, 2015, J AM COLL CARDIOL, V66, P848, DOI 10.1016/j.jacc.2015.06.1084

Ricci F, 2015, EUR HEART J, V36, P1609, DOI 10.1093/eurheartj/ehv093

Rose KM, 2006, CIRCULATION, V114, P630, DOI 10.1161/CIRCULATIONAHA.105.598722

Rubenstein LZ, 2006, AGE AGEING, V35, P37, DOI 10.1093/ageing/afl084

RUTAN GH, 1992, HYPERTENSION, V19, P508, DOI 10.1161/01.HYP.19.6.508

Schatz IJ, 1996, NEUROLOGY, V46, P1470, DOI 10.1212/wnl.46.5.1470

Shibao C, 2013, J AM SOC HYPERTENS, V7, P317, DOI 10.1016/j.jash.2013.04.006

van Wijnen VK, 2018, J AM MED DIR ASSOC, V19, P786, DOI 10.1016/j.jamda.2018.05.031

Veronese N, 2015, AM J HYPERTENS, V28, P1248, DOI 10.1093/ajh/hpv022

Verwoert GC, 2008, J AM GERIATR SOC, V56, P1816, DOI 10.1111/j.1532-5415.2008.01946.x

Vloet LCM, 2005, J GERONTOL A-BIOL, V60, P1271, DOI 10.1093/gerona/60.10.1271

Weiss A, 2002, ARCH INTERN MED, V162, P2369, DOI 10.1001/archinte.162.20.2369

Weiss A, 2006, J GEN INTERN MED, V21, P602, DOI 10.1111/j.1525-1497.2006.00450.x

Wu JS, 2008, HYPERTENS RES, V31, P897, DOI 10.1291/hypres.31.897

Xin W, 2013, PLOS ONE, V8, DOI 10.1371/journal.pone.0063169

Yasa E, 2018, HEART, V104, P487, DOI 10.1136/heartjnl-2017-311857

Zhou Y, 2017, MEDICINE, V96, DOI 10.1097/MD.0000000000008004

NR 61

TC 7

Z9 7

U1 0

U2 4

PU TAYLOR & FRANCIS LTD

PI ABINGDON

PA 2-4 PARK SQUARE, MILTON PARK, ABINGDON OR14 4RN, OXON, ENGLAND

SN 0300-7995

EI 1473-4877

J9 CURR MED RES OPIN

JI Curr. Med. Res. Opin.

PD JUN 3

PY 2019

VL 35

IS 6

BP 947

EP 955

DI 10.1080/03007995.2018.1546679

PG 9

WC Medicine, General & Internal; Medicine, Research & Experimental

WE Science Citation Index Expanded (SCI-EXPANDED); Social Science Citation Index (SSCI)

SC General & Internal Medicine; Research & Experimental Medicine

GA HW0YP

UT WOS:000466409100002

PM 30411636

DA 2024-08-08

ER

PT J

AU KATZ, PR

KARUZA, J

HALL, N

AF KATZ, PR

KARUZA, J

HALL, N

TI RESIDENCY EDUCATION IN THE NURSING-HOME - A NATIONAL SURVEY OF

INTERNAL-MEDICINE AND FAMILY-PRACTICE PROGRAMS

SO JOURNAL OF GENERAL INTERNAL MEDICINE

LA English

DT Article

DE NURSING HOMES; FAMILY PRACTICE; INTERNAL MEDICINE; RESIDENT EDUCATION;

PROGRAM DIRECTORS

AB Objective: To assess the role of the nursing home in post-graduate medical education.

Design: A survey questionnaire addressing the following issues: 1) prevalence of nursing home rotations in internal medicine and family practice residency programs, 2) duration and type of rotation, 3) extent of residents' responsibilities, 4) patterns of faculty supervision, 5) program directors' assessments of nursing home experience, and 6) frequently encountered problems.

Participants: Directors of accredited internal medicine and family practice residency programs in the United States.

Measurements and main results: A total of 502 surveys were returned for a response rate of 60%. Nursing home rotations were more frequent in family practice programs (87%) compared with internal medicine programs (32%). Rotations in internal medicine were generally optional and limited to a short block of time compared with family practice, where rotations were most often required and longitudinal. Internal medicine residents received more intense supervision, whereas family practice residents had greater clinical responsibilities. Few faculty had formal geriatric training or certification. Reimbursement for physician services was low. Although availability of faculty, resident interest, and conflict with other clinical services were identified as problem areas, program directors in both internal medicine and family practice were supportive of nursing home rotations for their trainees.

RP KATZ, PR (corresponding author), UNIV ROCHESTER,MONROE COMMUNITY HOSP,SCH MED & DENT,435 E HENRIETTA RD,ROCHESTER,NY 14620, USA.

NR 0

TC 20

Z9 20

U1 0

U2 0

PU BLACKWELL SCIENCE INC

PI MALDEN

PA 350 MAIN ST, MALDEN, MA 02148

SN 0884-8734

J9 J GEN INTERN MED

JI J. Gen. Intern. Med.

PD JAN-FEB

PY 1992

VL 7

IS 1

BP 52

EP 56

DI 10.1007/BF02599103

PG 5

WC Health Care Sciences & Services; Medicine, General & Internal

WE Science Citation Index Expanded (SCI-EXPANDED)

SC Health Care Sciences & Services; General & Internal Medicine

GA HA095

UT WOS:A1992HA09500010

PM 1548548

DA 2024-08-08

ER

PT J

AU Kokorelias, KM

Leung, G

Jamshed, N

Grosse, A

Sinha, SK

AF Kokorelias, Kristina Marie

Leung, Grace

Jamshed, Namirah

Grosse, Anna

Sinha, Samir K.

TI Identifying the areas of low self-reported confidence of internal

medicine residents in geriatrics: a descriptive study of findings from a

structured geriatrics skills assessment survey

SO BMC MEDICAL EDUCATION

LA English

DT Article

DE Geriatrics; Medical education; Training; Needs assessment; Internal

medicine; Residency

ID INTERPROFESSIONAL EDUCATION; OLDER-ADULTS; SPECIALIST PHYSICIANS; CARE;

EXPERIENCE; MANAGEMENT; IMPROVE; UPDATE; IMPACT; SCALE

AB Background: Currently, no standardized methods exist to assess the geriatric skills and training needs of internal medicine trainees to enable them to become confident in caring for older patients. This study aimed to describe the self-reported confidence and training requirements in core geriatric skills amongst internal medicine residents in Toronto, Ontario using a standardized assessment tool. Methods: This study used a novel self-rating instrument, known as the Geriatric Skills Assessment Tool (GSAT), among incoming and current internal medicine residents at the University of Toronto, to describe self-reported confidence in performing, teaching and interest in further training with regard to 15 core geriatric skills previously identified by the American Board of Internal Medicine. Results: 190 (75.1%) out of 253 eligible incoming (Year 0) and current internal medicine residents (Years 1-3) completed the GSAT. Year 1-3 internal medicine residents who had completed a geriatric rotation reported being significantly more confident in performing 13/15 (P < 0.001 to P = 0.04) and in teaching 9/15 GSAT skills (P < 0.001 to P = 0.04). Overall, the residents surveyed identified their highest confidence in administering the Mini-Mental Status Examination and lowest confidence in assessing fall risk using a gait and balance tool, and in evaluating and managing chronic pain. Conclusion: A structured needs assessment like the GSAT can be valuable in identifying the geriatric training needs of internal medicine trainees based on their reported levels of self-confidence. Residents in internal medicine could further benefit from completing a mandatory geriatric rotation early in their training, since this may improve their overall confidence in providing care for the mostly older patients they will work with during their residency and beyond.

C1 [Kokorelias, Kristina Marie; Grosse, Anna; Sinha, Samir K.] Sinai Hlth Syst & Univ Hlth Network, Dept Med, Div Geriatr Med, Suite 475-600 Univ Ave, Toronto, ON M5G1X5, Canada.

[Kokorelias, Kristina Marie; Leung, Grace; Sinha, Samir K.] Univ Toronto, Dept Med, Div Geriatr Med, Med Sci Bldg,1 Kings Coll Cir, Toronto, ON M5S1A8, Canada.

[Jamshed, Namirah] UT Southwestern Med Ctr, Div Geriatr Med, Dallas, TX USA.

[Sinha, Samir K.] Johns Hopkins Univ Sch Med, Div Geriatr Med & Gerontol, Baltimore, MD USA.

C3 University of Toronto; Sinai Health System Toronto; Lunenfeld Tanenbaum

Research Institute; University Health Network Toronto; University of

Toronto; University of Texas System; University of Texas Southwestern

Medical Center Dallas; Johns Hopkins University

RP Sinha, SK (corresponding author), Sinai Hlth Syst & Univ Hlth Network, Dept Med, Div Geriatr Med, Suite 475-600 Univ Ave, Toronto, ON M5G1X5, Canada.; Sinha, SK (corresponding author), Univ Toronto, Dept Med, Div Geriatr Med, Med Sci Bldg,1 Kings Coll Cir, Toronto, ON M5S1A8, Canada.; Sinha, SK (corresponding author), Johns Hopkins Univ Sch Med, Div Geriatr Med & Gerontol, Baltimore, MD USA.

EM samir.sinha@sinaihealth.ca

OI Grosse, Anna/0000-0003-3160-0319

FU Sinai Health's Healthy Ageing and Geriatrics Program Research Fund

FX This work was supported by Sinai Health's Healthy Ageing and Geriatrics

Program Research Fund.

CR Ahmed NN, 2011, J AM GERIATR SOC, V59, P143, DOI 10.1111/j.1532-5415.2010.03235.x

Al Ansari M, 2021, J MED EDUC CURRIC DE, V8, DOI 10.1177/2382120521999669

Alexandraki I, 2017, J GEN INTERN MED, V32, P871, DOI 10.1007/s11606-017-4004-3

Bacsu J, 2020, CAN GERIATR J, V23, P340, DOI 10.5770/cgj.23.426

Banerjee D, 2020, ASIAN J PSYCHIATR, V51, DOI 10.1016/j.ajp.2020.102154

Basu M, 2021, CAN GERIATR J, V24, P200, DOI 10.5770/cgj.24.538

Bone AE, 2018, PALLIATIVE MED, V32, P329, DOI 10.1177/0269216317734435

Boyd C, 2019, J AM GERIATR SOC, V67, P665, DOI 10.1111/jgs.15809

Bucholz EM, 2011, ARCH SURG-CHICAGO, V146, P907, DOI 10.1001/archsurg.2011.178

Cesari M, 2016, EUR J INTERN MED, V31, P11, DOI 10.1016/j.ejim.2016.03.005

Cody R.P., 2011, SAS STAT EXAMPLE

Croasmun J. T., 2011, Journal of Adult Education, V40, P19

Di Leo G, 2020, EUR RADIOL EXP, V4, DOI 10.1186/s41747-020-0145-y

Djatche L, 2018, J CLIN PHARM THER, V43, P550, DOI 10.1111/jcpt.12688

Donelan K, 2019, HEALTH AFFAIR, V38, P941, DOI 10.1377/hlthaff.2019.00030

Douglass AB, 2021, FAM MED, V53, P599, DOI 10.22454/FamMed.2021.750646

Edlund A, 2006, J GERIATR PSYCH NEUR, V19, P83, DOI 10.1177/0891988706286509

Fillmore WJ, 2013, J ORAL MAXIL SURG, V71, P448, DOI 10.1016/j.joms.2012.05.006

Finn GM, 2012, UNSKILLED UNAWARE EF, DOI [10.1096/fasebj.26.1_supplement.204.5, DOI 10.1096/FASEBJ.26.1_SUPPLEMENT.204.5]

Flaherty E, 2019, J AM GERIATR SOC, V67, pS400, DOI 10.1111/jgs.15924

Flores-Sandoval C, 2021, GERONTOL GERIATR EDU, V42, P178, DOI 10.1080/02701960.2020.1805320

FOLSTEIN MF, 1975, J PSYCHIAT RES, V12, P189, DOI 10.1016/0022-3956(75)90026-6

Fonseca AL, 2014, AM J SURG, V207, P797, DOI 10.1016/j.amjsurg.2013.09.033

Geoffrion R, 2013, J OBSTET GYNAECOL CA, V35, P355, DOI 10.1016/S1701-2163(15)30964-6

Gómez-Belda AB, 2021, GERIATR GERONTOL INT, V21, P60, DOI 10.1111/ggi.14102

Hanlon P, 2018, LANCET PUBLIC HEALTH, V3, pE323, DOI 10.1016/S2468-2667(18)30091-4

Henry JA, 2009, RURAL REMOTE HEALTH, V9, P1, DOI DOI 10.22605/RRH1083

Hoang P, 2021, CAN GERIATR J, V24, P304, DOI 10.5770/cgj.24.503

Hodges B, 2001, ACAD MED, V76, pS87, DOI 10.1097/00001888-200110001-00029

Hogan DB, 2012, CAN GERIATR J, V15, P68, DOI 10.5770/cgj.15.41

Hunter JP, 2015, PAIN RES MANAG, V20, pE12, DOI 10.1155/2015/159580

Hurria A, 2017, J AM GERIATR SOC, V65, P680, DOI 10.1111/jgs.14708

Jamshed N, 2012, J AM GERIATRICS SOC

Kämmer JE, 2022, MED EDUC, V56, P4, DOI 10.1111/medu.14692

Klebanoff JS, 2020, BMC MED EDUC, V20, DOI 10.1186/s12909-020-02090-9

Kokorelias KM, 2022, DISABIL REHABIL, P1

Landefeld CS, 2003, ANN INTERN MED, V139, P609, DOI 10.7326/0003-4819-139-7-200310070-00034

Levine DM, 2016, JAMA INTERN MED, V176, P1778, DOI 10.1001/jamainternmed.2016.6217

Levine SA, 2008, J AM GERIATR SOC, V56, P1140, DOI 10.1111/j.1532-5415.2008.01710.x

Lim SYS, 2020, J GERIATR ONCOL, V11, P566, DOI 10.1016/j.jgo.2019.09.008

Masud T, 2022, EUR GERIATR MED, V13, P513, DOI 10.1007/s41999-021-00595-0

McGilton KS, 2018, BMC GERIATR, V18, DOI 10.1186/s12877-018-0925-x

Miller RK, 2017, J AM GERIATR SOC, V65, pE130, DOI 10.1111/jgs.14968

Molnar F, 2019, CAN FAM PHYSICIAN, V65, P39

Nagamine M., 2006, Trends in elderly hospitalizations, 1997-2004

Parmar J., 2009, CANADIAN J GERIATRIC, V12, P70

Pazan F, 2021, EUR GERIATR MED, V12, P443, DOI 10.1007/s41999-021-00479-3

Pilleron S, 2019, INT J CANCER, V144, P49, DOI 10.1002/ijc.31664

Rahmani Mariam, 2020, J Grad Med Educ, V12, P532, DOI 10.4300/JGME-D-20-00134.1

Reger GM, 2021, CURR PSYCHIAT REP, V23, DOI 10.1007/s11920-021-01273-5

Satterwhite T, 2014, ANN PLAS SURG, V72, pS84, DOI 10.1097/SAP.0000000000000139

Sedhom R, 2019, SAGE OPEN, V9, DOI 10.1177/2158244019827678

Sedhom R, 2016, AM J MED QUAL, V31, P606, DOI 10.1177/1062860616653183

Senger E, 2019, CAN MED ASSOC J, V191, pE55, DOI 10.1503/cmaj.109-5698

Shorter E., 2013, Partnership for excellence: medicine at the University of Toronto and academic hospitals

Siegler Eugenia L, 2014, J Grad Med Educ, V6, P521, DOI 10.4300/JGME-D-13-00344.1

Sleeman KE, 2019, LANCET GLOB HEALTH, V7, pE883, DOI 10.1016/S2214-109X(19)30172-X

Soulis G, 2021, EUR GERIATR MED, V12, P205, DOI 10.1007/s41999-020-00419-7

Southerland LT, 2020, ANN EMERG MED, V75, P162, DOI 10.1016/j.annemergmed.2019.08.430

The Time Higher Education, 2022, TIME HIGHER ED

Wastesson JW, 2018, EXPERT OPIN DRUG SAF, V17, P1185, DOI 10.1080/14740338.2018.1546841

Wilkening GL, 2017, ACAD PSYCHIATR, V41, P71, DOI 10.1007/s40596-016-0531-1

Williams Brent C, 2010, J Grad Med Educ, V2, P373, DOI 10.4300/JGME-D-10-00065.1

Williams VM, 2021, BRACHYTHERAPY, V20, P128, DOI 10.1016/j.brachy.2020.08.022

Wojcik BM, 2021, J SURG EDUC, V78, P1838, DOI 10.1016/j.jsurg.2021.04.003

NR 65

TC 0

Z9 0

U1 2

U2 3

PU BMC

PI LONDON

PA CAMPUS, 4 CRINAN ST, LONDON N1 9XW, ENGLAND

EI 1472-6920

J9 BMC MED EDUC

JI BMC Med. Educ.

PD DEC 15

PY 2022

VL 22

IS 1

AR 870

DI 10.1186/s12909-022-03934-2

PG 11

WC Education & Educational Research; Education, Scientific Disciplines

WE Science Citation Index Expanded (SCI-EXPANDED); Social Science Citation Index (SSCI)

SC Education & Educational Research

GA 7C1JG

UT WOS:000899575900004

PM 36522619

OA Green Published, gold

DA 2024-08-08

ER

PT J

AU Larson, EB

Fihn, SD

Kirk, LM

Levinson, W

Loge, RV

Reynolds, E

Sandy, L

Schroeder, S

Wenger, N

Williams, M

AF Larson, EB

Fihn, SD

Kirk, LM

Levinson, W

Loge, RV

Reynolds, E

Sandy, L

Schroeder, S

Wenger, N

Williams, M

TI The future of general internal medicine - Report and recommendations

from the Society of General Internal Medicine (SGIM) task force on the

domain of general internal medicine

SO JOURNAL OF GENERAL INTERNAL MEDICINE

LA English

DT Article

DE primary care; medical education; physician payment; hospitalist;

geriatrics

ID IMPROVING PRIMARY-CARE; REGIONAL-VARIATIONS; CHRONIC ILLNESS;

COLLABORATIVE CARE; CLINICAL-RESEARCH; WOMEN PHYSICIANS; SATISFACTION;

DEPRESSION; SPECIALTY; MEDICARE

AB The Society of General Internal Medicine asked a task force to redefine the domain of general internal medicine. The task force believes that the chaos and dysfunction that characterize today's medical care, and the challenges facing general internal medicine, should spur innovation. These are our recommendations: while remaining true to its core values and competencies, general internal medicine should stay both broad and deep-ranging from uncomplicated primary care to continuous care of patients with multiple, complex, chronic diseases. Postgraduate and continuing education should develop mastery. Wherever they practice, general internists should be able to lead teams and be responsible for the care their teams give, embrace changes in information systems, and aim to provide most of the care their patients require. Current financing of physician services, especially fee-for-service, must be changed to recognize the value of services performed outside the traditional face-to-face visit and give practitioners incentives to improve quality and efficiency, and provide comprehensive, ongoing care. General internal medicine residency training should be reformed to provide both broad and deep medical knowledge, as well as mastery of informatics, management, and team leadership. General internal medicine residents should have options to tailor their final 1 to 2 years to fit their practice goals, often earning a certificate of added qualification (CAQ) in special generalist fields. Research will expand to include practice and operations management, developing more effective shared decision making and transparent medical records, and promoting the close personal connection that both doctors and patients want. We believe these changes constitute a paradigm shift that can benefit patients and the public and reenergize general internal medicine.

C1 Grp Hlth Cooperat Puget Sound, Ctr Hlth Studies, Seattle, WA 98101 USA.

Univ Washington, Harborview Med Ctr, Seattle, WA 98104 USA.

Univ Texas SW, Dallas, TX USA.

Univ Toronto, Toronto, ON, Canada.

SW Montana Clin, Dillon, MT USA.

Beth Israel Deaconess Med Ctr, Boston, MA 02215 USA.

United Hlth Care, Minneapolis, MN USA.

Univ Calif San Francisco, San Francisco, CA 94143 USA.

Univ Calif Los Angeles, Med Ctr, Los Angeles, CA 90024 USA.

Emory Univ, Atlanta, GA 30322 USA.

C3 Group Health Cooperative; Harborview Medical Center; University of

Washington; University of Washington Seattle; University of Texas

System; University of Texas Southwestern Medical Center Dallas;

University of Toronto; Harvard University; Beth Israel Deaconess Medical

Center; UnitedHealth Group Incorporated; University of California

System; University of California San Francisco; University of California

System; University of California Los Angeles; University of California

Los Angeles Medical Center; Emory University

RP Grp Hlth Cooperat Puget Sound, Ctr Hlth Studies, 1730 Minor Ave,Suite 1600, Seattle, WA 98101 USA.

EM larson.e@ghc.org

RI Fihn, Stephan/ABA-1040-2020

OI Williams, Mark V/0000-0001-6107-0457

CR Anderson G., 2002, Chronic conditions: Making the case for ongoing care

Anderson GF, 2003, ARCH INTERN MED, V163, P437, DOI 10.1001/archinte.163.4.437

*ASS AM MED COLL, 2002, MED STUD GRAD QUEST

*ASS AM MED COLL, 2002, DECL MED SCH APPL CO

Auerbach AD, 2002, ANN INTERN MED, V137, P859, DOI 10.7326/0003-4819-137-11-200212030-00006

Ayanian JZ, 1997, ARCH INTERN MED, V157, P2570, DOI 10.1001/archinte.157.22.2570

Ayanian JZ, 2002, NEW ENGL J MED, V347, P1678, DOI 10.1056/NEJMsa020080

Bodenheimer T, 2002, JAMA-J AM MED ASSOC, V288, P1775, DOI 10.1001/jama.288.14.1775

Bodenheimer T, 2002, JAMA-J AM MED ASSOC, V288, P1909, DOI 10.1001/jama.288.15.1909

Casalino L, 2003, JAMA-J AM MED ASSOC, V289, P434, DOI 10.1001/jama.289.4.434

*COMM QUAL HLTH CA, 2001, CROSS QUAL CHASM NEW

DAVIDOFF F, 1981, CONN MED, V45, P167

Dorsey ER, 2003, JAMA-J AM MED ASSOC, V290, P1173, DOI 10.1001/jama.290.9.1173

Eysenbach G, 2002, AM J MED, V113, P763, DOI 10.1016/S0002-9343(02)01473-0

Fisher ES, 2003, ANN INTERN MED, V138, P288, DOI 10.7326/0003-4819-138-4-200302180-00007

Fisher ES, 2003, ANN INTERN MED, V138, P273, DOI 10.7326/0003-4819-138-4-200302180-00006

Frank E, 1999, ARCH INTERN MED, V159, P1417, DOI 10.1001/archinte.159.13.1417

Ginsburg PB, 2003, ANN INTERN MED, V138, P233, DOI 10.7326/0003-4819-138-3-200302040-00020

Goldberg H I, 1998, Jt Comm J Qual Improv, V24, P130

Grumbach K, 1999, NEW ENGL J MED, V341, P2008, DOI 10.1056/NEJM199912233412611

Haas JS, 2000, J GEN INTERN MED, V15, P122, DOI 10.1046/j.1525-1497.2000.02219.x

Halvorson G, 2003, ANN INTERN MED, V138, P232

HASLAM D, 2003, BMJ-BRIT MED J, V326, P235

Hedrick SC, 2003, J GEN INTERN MED, V18, P9, DOI 10.1046/j.1525-1497.2003.11109.x

Heisler M, 2002, J GEN INTERN MED, V17, P243, DOI 10.1046/j.1525-1497.2002.10905.x

HENING S, 1999, CHANGING LANDSCAPE C

Hoffman C., 1996, Chronic Care in America: A 21st century challenge

Horowitz CR, 1996, JOINT COMM J QUAL IM, V22, P734, DOI 10.1016/S1070-3241(16)30279-6

Indridason OS, 2003, AM HEART J, V145, P300, DOI 10.1067/mhj.2003.54

Katon W, 1999, ARCH GEN PSYCHIAT, V56, P1109, DOI 10.1001/archpsyc.56.12.1109

Katon W, 1996, ARCH GEN PSYCHIAT, V53, P924

KATON W, 1995, JAMA-J AM MED ASSOC, V273, P1026, DOI 10.1001/jama.273.13.1026

Kitahata MM, 1996, NEW ENGL J MED, V334, P701, DOI 10.1056/NEJM199603143341106

KOHN LT, 1999, COMM QUAL HLTH CAR A

Landon BE, 2003, JAMA-J AM MED ASSOC, V289, P442, DOI 10.1001/jama.289.4.442

Larson EB, 2001, ANN INTERN MED, V134, P997, DOI 10.7326/0003-4819-134-10-200105150-00013

Leigh JP, 2002, ARCH INTERN MED, V162, P1577, DOI 10.1001/archinte.162.14.1577

Lenfant C, 2003, NEW ENGL J MED, V349, P868, DOI 10.1056/NEJMsa035507

LESSER CS, 2003, FINDINGS HSC, V63, P1

Linzer M, 2000, J GEN INTERN MED, V15, P441, DOI 10.1046/j.1525-1497.2000.05239.x

McMurray JE, 2000, J GEN INTERN MED, V15, P372, DOI 10.1046/j.1525-1497.2000.9908009.x

MELZER D, 2002, ANN INTERN MED, V137, P866

MIJKA M, 2002, JAMA-J AM MED ASSOC, V287, P1792

Moore G, 2003, ANN INTERN MED, V138, P244, DOI 10.7326/0003-4819-138-3-200302040-00032

Newton DA, 2003, JAMA-J AM MED ASSOC, V290, P1179, DOI 10.1001/jama.290.9.1179

Ochs Joyce, 2002, Manag Care, V11, P48

Peabody FW, 1927, J AMER MED ASSOC, V88, P0877, DOI 10.1001/jama.1927.02680380001001

Phelps CE, 2003, ANN INTERN MED, V138, P348, DOI 10.7326/0003-4819-138-4-200302180-00016

Reinertsen JL, 2003, ANN INTERN MED, V138, P992, DOI 10.7326/0003-4819-138-12-200306170-00011

Reiser SJ, 2003, ANN INTERN MED, V138, P844, DOI 10.7326/0003-4819-138-10-200305200-00014

ROMANO M, 2003, MODERN HEALTHCA 0721, P29

Rosenberg RN, 2003, JAMA-J AM MED ASSOC, V289, P1305, DOI 10.1001/jama.289.10.1305

Rothman AA, 2003, ANN INTERN MED, V138, P256, DOI 10.7326/0003-4819-138-3-200302040-00034

Safran DG, 2003, ANN INTERN MED, V138, P248, DOI 10.7326/0003-4819-138-3-200302040-00033

Sandy LG, 2003, ANN INTERN MED, V138, P262, DOI 10.7326/0003-4819-138-3-200302040-00035

Sandy LG, 2002, NEW ENGL J MED, V347, P1971, DOI 10.1056/NEJMsb021260

Schroeder SA, 2002, ACAD MED, V77, P767, DOI 10.1097/00001888-200208000-00003

SCHROEDER SA, 1986, ANN INTERN MED, V104, P554, DOI 10.7326/0003-4819-104-4-554

Shanafelt TD, 2003, AM J MED, V114, P513, DOI 10.1016/S0002-9343(03)00117-7

Showstack J, 2003, ANN INTERN MED, V138, P268, DOI 10.7326/0003-4819-138-3-200302040-00036

SOX HC, 1994, ANN INTERN MED, V121, P616, DOI 10.7326/0003-4819-121-8-199410150-00011

Sung NS, 2003, JAMA-J AM MED ASSOC, V289, P1278, DOI 10.1001/jama.289.10.1278

THUROW LC, 1999, ATLANTIC MONTHLY, V283, P57

Wachter RM, 2002, JAMA-J AM MED ASSOC, V287, P487, DOI 10.1001/jama.287.4.487

Wagner EH, 2001, HEALTH AFFAIR, V20, P64, DOI 10.1377/hlthaff.20.6.64

Weingarten SR, 2002, ARCH INTERN MED, V162, P527, DOI 10.1001/archinte.162.5.527

Wells KB, 2000, JAMA-J AM MED ASSOC, V283, P212, DOI 10.1001/jama.283.2.212

Wilensky GR, 2003, ANN INTERN MED, V138, P350, DOI 10.7326/0003-4819-138-4-200302180-00017

Wong MD, 2002, NEW ENGL J MED, V347, P1585, DOI 10.1056/NEJMsa012979

ZUGER A, 2003, NY TIMES 0107

NR 70

TC 80

Z9 86

U1 0

U2 17

PU SPRINGER

PI NEW YORK

PA ONE NEW YORK PLAZA, SUITE 4600, NEW YORK, NY, UNITED STATES

SN 0884-8734

EI 1525-1497

J9 J GEN INTERN MED

JI J. Gen. Intern. Med.

PD JAN

PY 2004

VL 19

IS 1

BP 69

EP 77

DI 10.1111/j.1525-1497.2004.31337.x

PG 9

WC Health Care Sciences & Services; Medicine, General & Internal

WE Science Citation Index Expanded (SCI-EXPANDED)

SC Health Care Sciences & Services; General & Internal Medicine

GA 766EP

UT WOS:000188354000010

PM 14748863

OA Green Published

DA 2024-08-08

ER

PT J

AU Chang, A

Fernandez, H

Cayea, D

Chheda, S

Paniagua, M

Eckstrom, E

Day, H

AF Chang, Anna

Fernandez, Helen

Cayea, Danelle

Chheda, Shobhina

Paniagua, Miguel

Eckstrom, Elizabeth

Day, Hollis

TI Complexity in Graduate Medical Education: A Collaborative Education

Agenda for Internal Medicine and Geriatric Medicine

SO JOURNAL OF GENERAL INTERNAL MEDICINE

LA English

DT Article

DE medical education; graduate; assessment/evaluation; geriatrics; aging;

care transitions; comorbidity

ID RESIDENCY CLINICS; PRIMARY-CARE; PHYSICIAN; TRANSITIONS; QUALITY; MODEL

AB Internal medicine residents today face significant challenges in caring for an increasingly complex patient population within ever-changing education and health care environments. As a result, medical educators, health care system leaders, payers, and patients are demanding change and accountability in graduate medical education (GME). A 2012 Society of General Internal Medicine (SGIM) retreat identified medical education as an area for collaboration between internal medicine and geriatric medicine. The authors first determined a short-term research agenda for resident education by mapping selected internal medicine reporting milestones to geriatrics competencies, and listing available sample learner assessment tools. Next, the authors proposed a strategy for long-term collaboration in three priority areas in clinical medicine that are challenging for residents today: (1) team-based care, (2) transitions and readmissions, and (3) multi-morbidity. The short-term agenda focuses on learner assessment, while the long-term agenda allows for program evaluation and improvement. This model of collaboration in medical education combines the resources and expertise of internal medicine and geriatric medicine educators with the goal of increasing innovation and improving outcomes in GME targeting the needs of our residents and their patients. (C) Society of General Internal Medicine 2014

C1 [Chang, Anna] Univ Calif San Francisco, Dept Med, Div Geriatr, San Francisco, CA 94118 USA.

[Fernandez, Helen] Icahn Sch Med Mt Sinai, Brookdale Dept Geriatr & Palliat Med, New York, NY 10029 USA.

[Cayea, Danelle] Johns Hopkins Univ Sch Med, Div Geriatr Med & Gerontol, Baltimore, MD USA.

[Chheda, Shobhina] Univ Wisconsin Sch Med & Publ Hlth, Div Gen Internal Med, Dept Med, Madison, WI USA.

[Paniagua, Miguel] St Louis Univ Sch Med, Div Gen Internal Med, St Louis, MO USA.

[Eckstrom, Elizabeth] Oregon Hlth & Sci Univ, Div Gen Internal Med & Geriatr, Portland, OR 97201 USA.

[Day, Hollis] Univ Pittsburgh Sch Med, Div Gen Internal Med, Pittsburgh, PA USA.

C3 University of California System; University of California San Francisco;

Icahn School of Medicine at Mount Sinai; Johns Hopkins University;

University of Wisconsin System; Saint Louis University; Oregon Health &

Science University; Pennsylvania Commonwealth System of Higher Education

(PCSHE); University of Pittsburgh

RP Chang, A (corresponding author), Univ Calif San Francisco, Dept Med, Div Geriatr, 3333 Calif St,Suite 380, San Francisco, CA 94118 USA.

EM anna.chang@ucsf.edu

RI Paniagua, Miguel A/T-4713-2019; Chang, Anna/AAS-2652-2020; chang,

anna/JXM-8182-2024

OI Paniagua, Miguel A/0000-0003-2307-4873; Cayea,

Danelle/0000-0003-2061-2007; Day, Hollis/0000-0001-8817-5160

FU Association of Specialty Professors

FX This individual paper had no funding support, and the authors thank the

Association of Specialty Professors for the overall support of the SGIM

symposium. This work has not been presented before.

CR American Academy of Family Physicians, 2008, Del Med J, V80, P21

American Geriatrics Society Expert Panel on the Care of Older Adults with Multimorbidity, 2012, J Am Geriatr Soc, V60, P1957, DOI 10.1111/j.1532-5415.2012.04187.x

[Anonymous], 2013, ALL AC INT MED RES E

[Anonymous], 2012, AGS UPD BEERS CRIT P

[Anonymous], 2013, PORT GER ONL ED

[Anonymous], 2012, 3 MOR MAN MULT HLTH

[Anonymous], 2012, NAM Perspectives

Axon N, 2012, AGING Q3 CURRICULUM

Berwick DM, 2012, JAMA-J AM MED ASSOC, V307, P1513, DOI 10.1001/jama.2012.362

Boyd CM, 2007, GERONTOLOGIST, V47, P697, DOI 10.1093/geront/47.5.697

Brotherton SE, 2012, JAMA-J AM MED ASSOC, V308, P2264, DOI 10.1001/jama.2012.7913

Carter BL, 2009, ARCH INTERN MED, V169, P1748, DOI 10.1001/archinternmed.2009.316

Caverzagie KJ, 2013, ANN INTERN MED, V158, P557, DOI 10.7326/0003-4819-158-7-201304020-00593

Caverzagie KJ, 2013, SOC GEN INT MED ANN

Chang A, 2012, J GEN INTERN MED

Counsell SR, 2006, J AM GERIATR SOC, V54, P1136, DOI 10.1111/j.1532-5415.2006.00791.x

Cumbler E, 2009, COMPETENCY BASED TES

DuBeau Catherine, 2009, CHAMP CURRICULUM HOS

Ehrlich A, 2011, CLIN EVALUATION EXER

Ehrlich A, 2013, COMMUNICATION SKILLS

Fick D, 2012, J AM GERIATR SOC, V60, P616, DOI 10.1111/j.1532-5415.2012.03923.x

Gentili A, 2011, FALLS CLIN EVALUATIO

Green Michael L, 2009, J Grad Med Educ, V1, P5, DOI 10.4300/01.01.0003

Heflin MT, 2012, ACAD MED, V87, P618, DOI 10.1097/ACM.0b013e31824d5251

Holmboe ES, 2010, MED TEACH, V32, P676, DOI 10.3109/0142159X.2010.500704

Iglehart JK, 2010, NEW ENGL J MED, V363, P584, DOI 10.1056/NEJMhpr1006115

Jencks SF, 2009, NEW ENGL J MED, V360, P1418, DOI 10.1056/NEJMsa0803563

Johnston C. Bree, 2009, MMSE CEX

Landefeld CS, 2003, ANN INTERN MED, V139, P609, DOI 10.7326/0003-4819-139-7-200310070-00034

Leff B, 2005, ANN INTERN MED, V143, P798, DOI 10.7326/0003-4819-143-11-200512060-00008

Leigh JP, 2009, BMC HEALTH SERV RES, V9, DOI 10.1186/1472-6963-9-166

Lynn L, 2012, HEALTH AFFAIR, V31, P150, DOI 10.1377/hlthaff.2011.0907

Lynn LA, 2009, ACAD MED, V84, P1732, DOI 10.1097/ACM.0b013e3181bf6f38

Marengoni A, 2011, AGEING RES REV, V10, P430, DOI 10.1016/j.arr.2011.03.003

Nasca TJ, 2012, NEW ENGL J MED, V366, P1051, DOI 10.1056/NEJMsr1200117

Njeim Mario, 2012, J Grad Med Educ, V4, P505, DOI 10.4300/JGME-D-11-00284.1

Paniagua M, 2006, 3 DS COGNITIVE IMPAI

Parry Carla, 2009, Home Health Care Serv Q, V28, P84, DOI 10.1080/01621420903155924

Record JD, 2011, ARCH INTERN MED, V171, P858, DOI 10.1001/archinternmed.2011.156

Robinson S, 2012, POPUL HEALTH MANAG, V15, P338, DOI 10.1089/pop.2011.0095

Roche V, 2011, SYSTEMS BASED APPROA

Saba GW, 2012, ANN FAM MED, V10, P169, DOI 10.1370/afm.1353

Salas E, 2009, JT COMM J QUAL PATIE, V35, P398, DOI 10.1016/S1553-7250(09)35056-4

Snow V, 2009, J HOSP MED, V4, P364, DOI 10.1002/jhm.510

Sochalski J, 2009, HEALTH AFFAIR, V28, P179, DOI 10.1377/hlthaff.28.1.179

Sutcliffe KM, 2004, ACAD MED, V79, P186, DOI 10.1097/00001888-200402000-00019

ten Cate O, 2007, ACAD MED, V82, P542, DOI 10.1097/ACM.0b013e31805559c7

Thomas DC, 2003, ANN INTERN MED, V139, P628, DOI 10.7326/0003-4819-139-7-200310070-00037

Thompson K, 2012, INTERPROFESSIONAL TE

Wang M, 2011, DISCHARGE ASSESSMENT

Williams Brent C, 2010, J Grad Med Educ, V2, P373, DOI 10.4300/JGME-D-10-00065.1

Zwarentein M, 2009, COCHRANE DB SYST REV, DOI 10.1002/14651858.CD000072.pub2

NR 52

TC 19

Z9 20

U1 0

U2 14

PU SPRINGER

PI NEW YORK

PA 233 SPRING ST, NEW YORK, NY 10013 USA

SN 0884-8734

EI 1525-1497

J9 J GEN INTERN MED

JI J. Gen. Intern. Med.

PD JUN

PY 2014

VL 29

IS 6

BP 940

EP 946

DI 10.1007/s11606-013-2752-2

PG 7

WC Health Care Sciences & Services; Medicine, General & Internal

WE Science Citation Index Expanded (SCI-EXPANDED); Social Science Citation Index (SSCI)

SC Health Care Sciences & Services; General & Internal Medicine

GA AK1VS

UT WOS:000338206600025

PM 24557513

OA Green Published, Green Submitted

DA 2024-08-08

ER

PT J

AU Díez-Manglano, J

Yangüela, BD

Calvo, EGA

Díez, EU

López, EM

Sierra, MC

Pinilla, PR

Sanvicente, TO

AF Diez-Manglano, Jesus

de Escalante Yangueela, Begona

Garcia-Arilla Calvo, Ernesto

Ubis Diez, Elena

Munilla Lopez, Eulalia

Clerencia Sierra, Mercedes

Revillo Pinilla, Paz

Omiste Sanvicente, Teresa

CA PLUPAR Study Researchers

TI Differential characteristics in polypathological inpatients in internal

medicine departments and acute geriatric units: The PLUPAR study

SO EUROPEAN JOURNAL OF INTERNAL MEDICINE

LA English

DT Article

DE Polypathological patient; Multimorbidity; Dependence; Cognitive

impairment; Geriatrics; Internal medicine

ID COGNITIVE IMPAIRMENT; OLDER PERSONS; CARE

AB Aim: To determine whether there are any differences between polypathological patients attended in Internal Medicine departments and acute Geriatric units.

Methods: A cross-sectional multicenter study was performed. Polypathological patients admitted to an internal medicine or geriatrics department and attended by investigators consecutively between March 1 and June 30, 2011 were included. Data of age, sex, living in a nursing residence or at home, diagnostic category, use of chronic medication, Charlson, Barthel and Lawton-Brody indexes, Pfeiffer questionnaire, delirium during last admission, need of a caregiver, and having a caregiver were gathered. The need of a caregiver was defined when the Barthel index was < 60 or Pfeiffer questionnaire >= 3 errors.

Results: 471 polypathological patients, 337 from internal medicine and 144 from geriatrics units were included. Geriatrics inpatients were older and more frequently female. Cardiac (62.1% vs 49.6%; p = .01), digestive (8.3% vs 3.0%; p = .04) and oncohematological diseases (30.2% vs 18.8%; p = .01) were more frequent in patients of internal medicine units and neurological (66.2% vs 40.2%; p < .001) and locomotive ones (39.1% vs 20.4%; p < .001) in geriatrics inpatients. Charlson index was higher for internal medicine inpatients [4.0(2.1) vs 3.5(2.1); p = .04). Patients attended in geriatrics scored higher in Pfeiffer questionnaire [5.5(3.7) vs 3.8(3.3); p < .001], and lower in Barthel [38.8(32.5) vs 61.2(34.3); p = .001] and Lawton-Brody indexes [0.9(1.6) vs 3.0(2.9); p < .001], and more frequently needed a caregiver (87.8% vs 53.6%; p < .001) and had it.

Conclusions: There are differences in disease profile and functional and cognitive situation between polypathological patients of internal medicine and geriatrics departments. (C) 2013 European Federation of Internal Medicine. Published by Elsevier B.V. All rights reserved.

C1 [Diez-Manglano, Jesus; Munilla Lopez, Eulalia] Hosp Royo Villanova, Dept Internal Med, Zaragoza 50015, Spain.

[Diez-Manglano, Jesus; de Escalante Yangueela, Begona; Revillo Pinilla, Paz] Inst Aragones Ciencias Salud, Comorbid & Polypathol Aragon Res Grp, Zaragoza, Spain.

[de Escalante Yangueela, Begona] Hosp Clin Univ Lozano Blesa, Dept Internal Med, Zaragoza, Spain.

[Garcia-Arilla Calvo, Ernesto] Hosp Nuestra Senora Gracia, Dept Geriatr, Zaragoza 50003, Spain.

[Ubis Diez, Elena] Hosp Sagrado Corazon, Dept Geriatr, Huesca, Spain.

[Clerencia Sierra, Mercedes] Hosp Univ Miguel Servet, Dept Geriatr, Zaragoza, Spain.

[Revillo Pinilla, Paz] Hosp Univ Miguel Servet, Dept Internal Med, Zaragoza, Spain.

[Omiste Sanvicente, Teresa] Hosp San Jorge, Dept Internal Med, Huesca, Spain.

C3 Lozano Blesa University Clinical Hospital; Miguel Servet University

Hospital; Miguel Servet University Hospital; Hospital Universidad San

Jorge

RP Díez-Manglano, J (corresponding author), Hosp Royo Villanova, Avda San Gregorio 30, Zaragoza 50015, Spain.

EM jdiez@aragon.es; egarciaarilla@salud.aragon.es

RI Díez-Manglano, J/AAC-3198-2020

OI Díez-Manglano, J/0000-0002-3132-2171; Garces Horna,

Vanesa/0000-0002-3485-0789

CR Alarcon M, 1998, Rev Esp Geriatr Gerontol, V33, P178

Ania-Lafuente BJ, 2006, REV ESP GERIATR GERO, V41, P142

Aragonese Statistics Institute, AR BAS DAT

Barnett K, 2012, LANCET, V380, P37, DOI 10.1016/S0140-6736(12)60240-2

Bernabeu-Wittel M, 2011, ARCH GERONTOL GERIAT, V53, P284, DOI 10.1016/j.archger.2010.12.006

Bernabeu-Wittel M, 2011, EUR J INTERN MED, V22, P311, DOI 10.1016/j.ejim.2010.11.012

Bernabeu-Wittel M, 2010, ARCH GERONTOL GERIAT, V51, P185, DOI 10.1016/j.archger.2009.10.006

Boyd CM, 2008, J AM GERIATR SOC, V56, P2171, DOI 10.1111/j.1532-5415.2008.02023.x

Bula C, 2006, Rev Med Suisse, V2, P2540

Buurman BM, 2011, PLOS ONE, V6, DOI 10.1371/journal.pone.0026951

CHARLSON ME, 1987, J CHRON DIS, V40, P373, DOI 10.1016/0021-9681(87)90171-8

de la Iglesia JM, 2001, MED CLIN-BARCELONA, V117, P129

Eurostat, MED AG POP POP 1 JAN

Gómez BJF, 2006, REV CLIN ESP, V206, P534

FOX RA, 1989, CAN MED ASSOC J, V141, P1045

García-Morillo JS, 2005, MED CLIN-BARCELONA, V125, P5, DOI 10.1157/13076399

Inouye SK, 2006, J GEN INTERN MED, V21, P1276, DOI 10.1111/j.1525-1497.2006.00613.x

Inouye SK, 1998, J GEN INTERN MED, V13, P234, DOI 10.1046/j.1525-1497.1998.00073.x

Joray S, 2004, AM J GERIAT PSYCHIAT, V12, P639, DOI 10.1176/appi.ajgp.12.6.639

Landefeld CS, 2003, ANN INTERN MED, V139, P609, DOI 10.7326/0003-4819-139-7-200310070-00034

LAWTON MP, 1969, GERONTOLOGIST, V9, P9, DOI 10.1093/geront/9.1.9

LIBOW LS, 1990, J AM GERIATR SOC, V38, P79, DOI 10.1111/j.1532-5415.1990.tb01603.x

MAHONEY F I, 1965, Md State Med J, V14, P61

Velilla NM, 2012, MED CLIN-BARCELONA, V139, P694, DOI 10.1016/j.medcli.2012.03.028

Ollero-Baturone M., 2007, ATENCION PACIENTE PL

Prados-Torres A, 2012, PLOS ONE, V7, DOI 10.1371/journal.pone.0032190

REUBEN DB, 1993, J AM GERIATR SOC, V41, P444, DOI 10.1111/j.1532-5415.1993.tb06955.x

Rodríguez AS, 2005, MED CLIN-BARCELONA, V125, P12, DOI 10.1157/13076406

Van Den Akker M, 1996, Eur J Gen Pract, V2, P65, DOI [10.3109/13814789609162146, DOI 10.3109/13814789609162146]

Zambrana Garcia JL, 2005, REV CLIN ESP, V205, P413

NR 30

TC 16

Z9 16

U1 0

U2 2

PU ELSEVIER

PI AMSTERDAM

PA RADARWEG 29, 1043 NX AMSTERDAM, NETHERLANDS

SN 0953-6205

EI 1879-0828

J9 EUR J INTERN MED

JI Eur. J. Intern. Med.

PD DEC

PY 2013

VL 24

IS 8

BP 767

EP 771

DI 10.1016/j.ejim.2013.07.010

PG 5

WC Medicine, General & Internal

WE Science Citation Index Expanded (SCI-EXPANDED)

SC General & Internal Medicine

GA 262VF

UT WOS:000327765300019

PM 23938328

DA 2024-08-08

ER

PT J

AU Rubin, CD

Stieglitz, H

Vicioso, B

Kirk, L

AF Rubin, CD

Stieglitz, H

Vicioso, B

Kirk, L

TI Development of geriatrics-oriented faculty in general internal medicine

SO ANNALS OF INTERNAL MEDICINE

LA English

DT Article

ID SOCIETY; MODEL

AB The need for adequate geriatrics training for the physician work-force has been recognized for decades. However, there are not enough academic geriatricians to provide for the educational needs of trainees, and this situation is not expected to change in the future. General internists are often responsible for teaching medical students and internal medicine residents to care for elderly patients in inpatient and ambulatory settings. These academic general internists could play a pivotal role in providing geriatrics instruction.

To characterize what is being done to develop geriatrics-oriented general internal medicine faculty, we identified current practices, "best practices," goals and targets, and barriers to achieving those goals and targets. We reviewed the literature on faculty-development programs for general internal medicine faculty, and we held focus groups and structured interviews with general internal medicine unit chiefs and directors of Geriatric Centers of Excellence at 46 medical schools throughout the United States. We found a need for programs to develop geriatrics-oriented academic general internists. Although general internal medicine faculties seem receptive to further geriatrics training, important obstacles exist. These include inadequate time and resources as well as motivational and attitudinal challenges. We discuss potential solutions for overcoming these barriers and the implications of these solutions for stakeholders.

C1 Univ Texas, SW Med Ctr, Dept Internal Med, Dallas, TX 75390 USA.

C3 University of Texas System; University of Texas Southwestern Medical

Center Dallas; University of Texas Dallas

RP Rubin, CD (corresponding author), Univ Texas, SW Med Ctr, Dept Internal Med, 5323 Harry Hines Blvd, Dallas, TX 75390 USA.

EM craig.rubin@utsouthwestern.edu

CR *BUR HLTH PROF HLT, 2000, GEN PRIM CAR FAC DEV

DANS PE, 1979, NEW ENGL J MED, V300, P228, DOI 10.1056/NEJM197902013000504

EMANUEL LL, 1999, EPEC ED PHYSICIANS E

Fasser C E, 1988, Gerontol Geriatr Educ, V8, P37, DOI 10.1300/J021v08n03_05

Feather J, 1988, Gerontol Geriatr Educ, V8, P165, DOI 10.1300/J021v08n03_14

Fihn SD, 2000, J GEN INTERN MED, V15, P451, DOI 10.1046/j.1525-1497.2000.07001.x

FLEMING M, 1992, ACAD MED, V67, P691, DOI 10.1097/00001888-199210000-00015

Hazzard WR, 1997, J AM GERIATR SOC, V45, P638, DOI 10.1111/j.1532-5415.1997.tb03102.x

*I MED, 1978, AG MED ED

Larson EB, 2001, ANN INTERN MED, V134, P997, DOI 10.7326/0003-4819-134-10-200105150-00013

REUBEN DB, 1993, J AM GERIATR SOC, V41, P560, DOI 10.1111/j.1532-5415.1993.tb01896.x

REUBEN DB, 1991, J AM GERIATR SOC, V39, P799, DOI 10.1111/j.1532-5415.1991.tb02703.x

REUBEN DB, 1994, TRAINING PHYSICIANS

ROBBINS LJ, 1993, J AM GERIATR SOC, V41, P570, DOI 10.1111/j.1532-5415.1993.tb01897.x

RUBIN C, 2001, DEV GERIATRICALLY OR

Sox HC, 2001, AM J MED, V110, P745, DOI 10.1016/S0002-9343(01)00756-2

Swagerty D, 2000, J AM GERIATR SOC, V48, P1513, DOI 10.1111/jgs.2000.48.11.1513

NR 17

TC 29

Z9 29

U1 0

U2 0

PU AMER COLL PHYSICIANS

PI PHILADELPHIA

PA INDEPENDENCE MALL WEST 6TH AND RACE ST, PHILADELPHIA, PA 19106-1572 USA

SN 0003-4819

EI 1539-3704

J9 ANN INTERN MED

JI Ann. Intern. Med.

PD OCT 7

PY 2003

VL 139

IS 7

BP 615

EP 620

DI 10.7326/0003-4819-139-7-200310070-00035

PG 6

WC Medicine, General & Internal

WE Science Citation Index Expanded (SCI-EXPANDED)

SC General & Internal Medicine

GA 729AP

UT WOS:000185748800027

PM 14530245

DA 2024-08-08

ER

PT J

AU Hazzard, WR

AF Hazzard, WR

TI The department of internal medicine: Hub of the academic health center

response to the aging imperative

SO ANNALS OF INTERNAL MEDICINE

LA English

DT Article

AB In the 21st century, geriatrics will increasingly dominate U.S. health care as the median age of the population progressively increases. Academic departments of geriatrics have been created in nations that have already experienced this shift. As an alternative strategy that builds on traditional strengths of academic medicine in the United States, departments of internal medicine should lead a multidepartmental, pan-institutional response to the aging imperative. Recognition of gerontology and geriatric medicine as central to the missions of internal medicine in clinical care, education, and research must be increased. In the process, academic departments of internal medicine will develop a high level of geriatric expertise and will launch many programs that address this challenge. Successful development of geriatric programs will serve as a catalyst to strengthen the integration among and between generalists and subspecialists. This will entail developing optimal sites and systems of geriatric care-at different levels of care and over time-that can enhance the geriatric education of medical students, residents, fellows, and practicing physicians. The study of aging and geriatric health care will also become an integral part of departmental research, in its subspecialty divisions as well as its divisions of general internal medicine and geriatrics. This strategy is urgently recommended as both a challenge and an opportunity for all departments of internal medicine.

C1 Vet Affairs Puget Sound Hlth Care Syst, Seattle, WA 98108 USA.

C3 US Department of Veterans Affairs; Veterans Health Administration (VHA);

Vet Affairs Puget Sound Health Care System

RP Vet Affairs Puget Sound Hlth Care Syst, 1660 S Columbian Way, Seattle, WA 98108 USA.

EM william.hazzard@med.va.gov

CR HAZZARD WR, 1986, AGE AGEING, V15, P307, DOI 10.1093/ageing/15.5.307

HAZZARD WR, 1979, AGE AGEING, V8, P141, DOI 10.1093/ageing/8.3.141

HAZZARD WR, 1994, AM J MED, V97

HAZZARD WR, 1990, AGING NEW DIMENSIONS

NR 4

TC 17

Z9 18

U1 0

U2 0

PU AMER COLL PHYSICIANS

PI PHILADELPHIA

PA INDEPENDENCE MALL WEST 6TH AND RACE ST, PHILADELPHIA, PA 19106-1572 USA

SN 0003-4819

EI 1539-3704

J9 ANN INTERN MED

JI Ann. Intern. Med.

PD AUG 15

PY 2000

VL 133

IS 4

BP 293

EP 296

DI 10.7326/0003-4819-133-4-200008150-00014

PG 4

WC Medicine, General & Internal

WE Science Citation Index Expanded (SCI-EXPANDED)

SC General & Internal Medicine

GA 346NG

UT WOS:000088877500007

PM 10929171

DA 2024-08-08

ER

PT J

AU Warshaw, GA

Bragg, EJ

Thomas, DC

Ho, ML

Brewer, DE

AF Warshaw, Gregg A.

Bragg, Elizabeth J.

Thomas, David C.

Ho, Mona L.

Brewer, David E.

TI Are internal medicine residency programs adequately preparing physicians

to care for the baby boomers? A national survey from the Association of

Directors of Geriatric Academic Programs Status of Geriatrics Workforce

Study

SO JOURNAL OF THE AMERICAN GERIATRICS SOCIETY

LA English

DT Article

DE geriatric medicine education; internal medicine; graduate medical

education

ID EDUCATION

AB Patients aged 65 and older account for 39% of ambulatory visits to internal medicine physicians. This article describes the progress made in training internal medicine residents to care for older Americans. Program directors in internal medicine residency programs accredited by the Accreditation Council for Graduate Medical Education were surveyed in the spring of 2005. Findings from this survey were compared with those from a similar 2002 survey to determine whether any changes had occurred. A 60% response rate was achieved (n = 235). In these 3-year residency training programs, 20 programs (9%) required less than 2 weeks of clinical instruction that was specifically structured to teach geriatric care principles, 48 (21%) at least 2 weeks but less than 4 weeks, 144 (62%) at least 4 weeks but less than 6 weeks, and 21 (9%) required 6 or more weeks. As in 2002, internal medicine residency programs continue to depend on nursing home facilities, geriatric preceptors in nongeriatric clinical ambulatory settings, and outpatient geriatric assessment centers for their geriatrics training. Training was most often offered in a block format. The mean number of physician faculty per residency program dedicated to teaching geriatric medicine was 3.5 full-time equivalents (FTEs) (range 0-50), compared with a mean of 2.2 FTE faculty in 2002 (P <=.001). Internal medicine educators are continuing to improve the training of residents so that, as they become practicing physicians, they will have the knowledge and skills in geriatric medicine to care for older adults.

C1 Univ Cincinnati, Off Geriatr Med, Dept Family Med, Cincinnati, OH 45267 USA.

Univ Cincinnati, Inst Study Hlth, Cincinnati, OH 45267 USA.

Mt Sinai Med Ctr, Dept Med, New York, NY 10029 USA.

Mt Sinai Med Ctr, Dept Rehabil, New York, NY 10029 USA.

Cincinnati Childrens Hosp, Ctr Med, Div Gen & Community Pediat, Cincinnati, OH USA.

C3 University System of Ohio; University of Cincinnati; University System

of Ohio; University of Cincinnati; Icahn School of Medicine at Mount

Sinai; Icahn School of Medicine at Mount Sinai; Cincinnati Children's

Hospital Medical Center

RP Warshaw, GA (corresponding author), Univ Cincinnati, Off Geriatr Med, Dept Family Med, POB 670504,231 Albert Sabin Way, Cincinnati, OH 45267 USA.

EM Gregg.Warshaw@uc.edu

CR *ACC COUNC GRAD ME, 2000, LIST ACC RES PROGR I

*ACCR COUNC GRAD M, PROGR REQ RES ED INT

*APDIM REC GER SUB, BEG WORK SPR M

Bragg EJ, 2006, FAM MED, V38, P258

BRAGG EJ, 2006, ACAD GERIATRIC PROGR

BRAGG EJ, GERIATRICIANS GERIAT

Counsell SR, 1999, J AM GERIATR SOC, V47, P1145, DOI 10.1111/j.1532-5415.1999.tb05242.x

Kansagara DL, 2005, J GEN INTERN MED, V20, P31

Landefeld CS, 2003, ANN INTERN MED, V139, P609, DOI 10.7326/0003-4819-139-7-200310070-00034

Li I, 2003, FAM MED, V35, P35

*NAT CTR HLTH STAT, 2004, TREND TABL 65 OLD PO

REYNOLDS DW, FDN AWARS 12 MILLION

REYNOLDS DW, FDN GERIATRIC TRANIN

Rubin CD, 2003, ANN INTERN MED, V139, P615, DOI 10.7326/0003-4819-139-7-200310070-00035

*SGIM TASK FORC ME, FUT GEN INT MED REP

Thomas DC, 2003, ANN INTERN MED, V139, P628, DOI 10.7326/0003-4819-139-7-200310070-00037

Warshaw GA, 2003, J GEN INTERN MED, V18, P679, DOI 10.1046/j.1525-1497.2003.20906.x

NR 17

TC 38

Z9 40

U1 0

U2 2

PU WILEY

PI HOBOKEN

PA 111 RIVER ST, HOBOKEN 07030-5774, NJ USA

SN 0002-8614

EI 1532-5415

J9 J AM GERIATR SOC

JI J. Am. Geriatr. Soc.

PD OCT

PY 2006

VL 54

IS 10

BP 1603

EP 1609

DI 10.1111/j.1532-5415.2006.00895.x

PG 7

WC Geriatrics & Gerontology; Gerontology

WE Science Citation Index Expanded (SCI-EXPANDED); Social Science Citation Index (SSCI)

SC Geriatrics & Gerontology

GA 096NY

UT WOS:000241385300018

PM 17038081

DA 2024-08-08

ER

PT J

AU Miller, RK

Michener, J

Yang, P

Goldstein, K

Groce-Martin, J

True, G

Johnson, J

AF Miller, Rachel K.

Michener, Jennifer

Yang, Phyllis

Goldstein, Karen

Groce-Martin, Jennine

True, Gala

Johnson, Jerry

TI Effect of a Community-Based Service Learning Experience in Geriatrics on

Internal Medicine Residents and Community Participants

SO JOURNAL OF THE AMERICAN GERIATRICS SOCIETY

LA English

DT Article

DE education; service learning; internal medicine; community

ID HEALTH; EDUCATION; CARE; CURRICULUM; PROGRAMS; IMPROVE

AB Community-based service learning (CBSL) provides an opportunity to teach internal medicine residents the social context of aging and clinical concepts. The objectives of the current study were to demonstrate the feasibility of a CBSL program targeting internal medicine residents and to assess its effect on medical residents and community participants. internal medicine residents participated in a CBSL experience for half a day during ambulatory blocks from 2011 to 2014. Residents attended a senior housing unit or center, delivered a presentation about a geriatric health topic, toured the facility, and received information about local older adult resources. Residents evaluated the experience. Postgraduate Year 3 internal medicine residents (n = 71) delivered 64 sessions. Residents felt that the experience increased their ability to communicate effectively with older adults (mean 3.91 +/- 0.73 on a Likert scale with 5 = strongly agree), increased their knowledge of resources (4.09 +/- 1.01), expanded their knowledge of a health topic pertinent to aging (3.48 +/- 1.09), and contributed to their capacity to evaluate and care for older adults (3.84 +/- 0.67). Free-text responses demonstrated that residents thought that this program would change their practice. Of 815 older adults surveyed from 36 discrete teaching sessions, 461 (56%) thought that the medical residents delivered health information clearly (4.55 +/- 0.88) and that the health topics were relevant (4.26 +/- 0.92). Free-text responses showed that the program helped them understand their health concerns. This CBSL program is a feasible and effective tool for teaching internal medicine residents and older adults.

C1 [Miller, Rachel K.] Univ Penn, Perelman Sch Med, Philadelphia, PA 19104 USA.

[Miller, Rachel K.; Groce-Martin, Jennine; Johnson, Jerry] Univ Penn, Dept Gen Internal Med, Div Geriatr, Philadelphia, PA 19104 USA.

[Michener, Jennifer; Yang, Phyllis] Hosp Univ Penn, 3400 Spruce St, Philadelphia, PA 19104 USA.

[Goldstein, Karen] Duke Univ, Sch Med, Div Gen Med, Durham, NC USA.

[Goldstein, Karen] Durham Vet Affairs Med Ctr, Hlth Serv Res & Dev, Durham, NC USA.

[True, Gala] Southeast Louisiana Vet Hlth Care Syst, New Orleans, LA USA.

C3 University of Pennsylvania; University of Pennsylvania; University of

Pennsylvania; Duke University; US Department of Veterans Affairs;

Veterans Health Administration (VHA); Durham VA Medical Center

RP Miller, RK (corresponding author), 3615 Chestnut St, Philadelphia, PA 19104 USA.

EM rachel.miller@uphs.upenn.edu

OI Goldstein, Karen/0000-0003-4419-5869

FU Donald W. Reynolds Foundation; Health Resources and Services

Administration (HRSA; Geriatric Academic Career Award) [K01HP20495]

FX A Donald W. Reynolds Foundation award provided funding for

implementation of elements of the Senior Community Experience in the

Perelman School of Medicine. Additional funding support came from the

Health Resources and Services Administration (HRSA; Geriatric Academic

Career Award K01HP20495 to Rachel K. Miller).

CR [Anonymous], 2010, UNDERSTANDING MED ED, DOI DOI 10.1002/9781444320282.CH2

[Anonymous], 2008, Retooling for an Aging America: Building the Health Care Workforce

[Anonymous], 2001, SERVICE ED HLTH, DOI DOI 10.1080/13576280110057563

Arora V, 2005, AM J MED, V118, P680, DOI 10.1016/j.amjmed.2005.03.022

Buckner AV, 2010, ACAD MED, V85, P1645, DOI 10.1097/ACM.0b013e3181f08348

Gefter L, 2015, FAM MED, V47, P803

Hunt JB, 2011, ACAD MED, V86, P246, DOI 10.1097/ACM.0b013e3182046481

Karasik RJ, 2007, J AM MED DIR ASSOC, V8, P284, DOI 10.1016/j.jamda.2007.02.012

Landefeld CS, 2003, ANN INTERN MED, V139, P609, DOI 10.7326/0003-4819-139-7-200310070-00034

Maurana CA, 1996, ACAD MED, V71, P425, DOI 10.1097/00001888-199605000-00009

McCrystle SW, 2010, J AM GERIATR SOC, V58, P142, DOI 10.1111/j.1532-5415.2009.02663.x

Muller D, 2010, ACAD MED, V85, P302, DOI 10.1097/ACM.0b013e3181c88434

O'Toole TP, 2005, ACAD MED, V80, P339, DOI 10.1097/00001888-200504000-00006

Saravanan Y, 2011, AM J PREV MED, V41, pS270, DOI 10.1016/j.amepre.2011.06.010

Seifer, 2000, CREATING COMMUNITY R

Seifer SD, 1998, ACAD MED, V73, P273, DOI 10.1097/00001888-199803000-00015

Smith KL, 2013, MED TEACH, V35, pE1139, DOI 10.3109/0142159X.2012.735383

Tartaglia Kimberly M, 2010, J Grad Med Educ, V2, P456, DOI 10.4300/JGME-D-10-00067.1

Thomas DC, 2003, ANN INTERN MED, V139, P628, DOI 10.7326/0003-4819-139-7-200310070-00037

Warshaw GA, 2006, J AM GERIATR SOC, V54, P1603, DOI 10.1111/j.1532-5415.2006.00895.x

Wenger NS, 2007, J AM GERIATR SOC, V55, pS247, DOI 10.1111/j.1532-5415.2007.01328.x

Williams Brent C, 2010, J Grad Med Educ, V2, P373, DOI 10.4300/JGME-D-10-00065.1

Yardley S, 2012, MED TEACH, V34, pE102, DOI 10.3109/0142159X.2012.650741

Young Staci, 2002, Educ Health (Abingdon), V15, P353, DOI 10.1080/1357628021000012796

NR 24

TC 6

Z9 7

U1 0

U2 4

PU WILEY

PI HOBOKEN

PA 111 RIVER ST, HOBOKEN 07030-5774, NJ USA

SN 0002-8614

EI 1532-5415

J9 J AM GERIATR SOC

JI J. Am. Geriatr. Soc.

PD SEP

PY 2017

VL 65

IS 9

BP E130

EP E134

DI 10.1111/jgs.14968

PG 5

WC Geriatrics & Gerontology; Gerontology

WE Science Citation Index Expanded (SCI-EXPANDED); Social Science Citation Index (SSCI)

SC Geriatrics & Gerontology

GA FH3PB

UT WOS:000411060500003

PM 28734046

OA Bronze

DA 2024-08-08

ER

PT J

AU Valero-Ubierna, MD

Benavente-Fernández, A

de Rojas, JP

Moreno-Verdejo, F

López-Gómez, J

Fernández-Ontiveros, S

Chueca-Porcuna, N

García-Marín, C

Jiménez-Moleón, JJ

Rivera-Izquierdo, M

AF Valero-Ubierna, Maria del Carmen

Benavente-Fernandez, Alberto

de Rojas, Javier Perez

Moreno-Verdejo, Fidel

Lopez-Gomez, Jairo

Fernandez-Ontiveros, Sergio

Chueca-Porcuna, Natalia

Garcia-Marin, Cristina

Jimenez-Moleon, Jose Juan

Rivera-Izquierdo, Mario

TI Social and clinical predictors of perianal colonisation by

multidrug-resistant bacteria for geriatric patients in the internal

medicine service

SO INFECTION

LA English

DT Article

DE Internal medicine; Multidrug resistance; Social; Bio-psycho-social;

Colonisation; Predictors

ID FECAL MICROBIOTA TRANSPLANTATION; MULTIPLE IMPUTATION; GUT MICROBIOTA;

MODULATION

AB Background Colonisation by multidrug-resistant (MDR) bacteria is a global health issue. The identification of patients with a higher risk of colonisation is essential. Patients admitted to internal medicine services might represent a vulnerable population with a high risk of colonisation. This study was the first to assess social and clinical variables associated with a higher risk of perianal colonisation by MDR bacteria in a Spanish cohort of patients admitted to internal medicine service.Methods Patients admitted to an internal medicine service during 12 months of recruitment (1 March 2022 to 1 March 2023) were included in the study. Perianal swabs were performed at admission to identify the presence of MDR bacteria. Social and clinical variables were collected following a directed acyclic graph. A cluster analysis was performed to identify clinical profiles of higher risk. Bivariate analyses and multivariable logistic regression models were fitted to identify potential predictors of MDR bacteria colonisation.Results A total of 245 patients, according to the required sample size, were included. Of them, 46 (18.8%) were colonised by MDR bacteria in perianal swabs. Female sex, age > 80 years, dependency on activities of daily living, cognitive deterioration and living in long-term care facilities constituted the highest risk clinical profile. After adjustments, living in long-term care facilities and malnutrition remained the main risk factors identified.Conclusion Patients admitted to internal medicine services presented a high frequency of perianal colonisation by MDR bacteria. Social and clinical variables associated with bio-psycho-social susceptibility were associated with colonisation. Special surveillance is needed in internal medicine services to control the transmission.

C1 [Valero-Ubierna, Maria del Carmen; de Rojas, Javier Perez; Garcia-Marin, Cristina; Jimenez-Moleon, Jose Juan; Rivera-Izquierdo, Mario] Hosp Univ San Cecilio, Serv Prevent Med & Publ Hlth, Ave Invest s-n, Granada 18016, Spain.

[Benavente-Fernandez, Alberto; Moreno-Verdejo, Fidel; Lopez-Gomez, Jairo; Fernandez-Ontiveros, Sergio] Hosp Univ San Cecilio, Serv Internal Med, Granada, Spain.

[Chueca-Porcuna, Natalia] Hosp Univ San Cecilio, Serv Microbiol, Granada, Spain.

[Chueca-Porcuna, Natalia; Jimenez-Moleon, Jose Juan; Rivera-Izquierdo, Mario] Inst Biosanitario Granada Ibs Granada, Granada, Spain.

[Chueca-Porcuna, Natalia] Ctr Biomed Res Network Infect Dis CIBERINFEC, Madrid, Spain.

[Garcia-Marin, Cristina] Hosp Univ La Paz, Serv Prevent Med & Publ Hlth, Madrid, Spain.

[Jimenez-Moleon, Jose Juan; Rivera-Izquierdo, Mario] CIBER Epidemiol & Salud Publ CIBERESP, Madrid, Spain.

C3 Hospital Universitario San Cecilio; Hospital Universitario San Cecilio;

Hospital Universitario San Cecilio; Instituto de Investigacion

Biosanitaria IBS Granada; Hospital Universitario La Paz; CIBER - Centro

de Investigacion Biomedica en Red; CIBERESP

RP Rivera-Izquierdo, M (corresponding author), Hosp Univ San Cecilio, Serv Prevent Med & Publ Hlth, Ave Invest s-n, Granada 18016, Spain.; Rivera-Izquierdo, M (corresponding author), Inst Biosanitario Granada Ibs Granada, Granada, Spain.; Rivera-Izquierdo, M (corresponding author), CIBER Epidemiol & Salud Publ CIBERESP, Madrid, Spain.

EM mariorivera@ugr.es

RI Pérez de Rojas, Javier/GQH-0322-2022

OI Pérez de Rojas, Javier/0000-0003-3225-397X; Benavente Fernandez,

Alberto/0000-0002-0960-6477

CR Andalusian Health Service, CAR PLUR PAT

Bilsen MP, 2022, CURR OPIN GASTROEN, V38, P15, DOI 10.1097/MOG.0000000000000792

Fernández MC, 2022, ANTIMICROB RESIST IN, V11, DOI 10.1186/s13756-022-01200-0

Campos-Madueno EI, 2023, EUR J CLIN MICROBIOL, V42, P229, DOI 10.1007/s10096-023-04548-2

Carvalho-Brugger S, 2023, MED INTENSIVA ENGL E, VS2173-5727, P00057

Dolci G, 2023, MICROORGANISMS, V11, DOI 10.3390/microorganisms11061606

European Society of Clinical Microbiology and Infectious Diseases, 2022, EUR COMM ANT SUSC TE

Gargiullo L, 2019, FRONT MICROBIOL, V10, DOI 10.3389/fmicb.2019.01704

Gonzalez-Montalvo Juan Ignacio, 2020, Rev Esp Geriatr Gerontol, V55, P84, DOI 10.1016/j.regg.2019.10.011

Junta de Andalucia, 2022, SVEA

Macareño-Castro J, 2022, J INFECTION, V84, P749, DOI 10.1016/j.jinf.2022.04.028

Martin EM, 2018, INFECT CONT HOSP EP, V39, P788, DOI 10.1017/ice.2018.93

Martinez JA, EFFICACY PROBIOTIC F

McEwen SA, 2018, ANTIMICROBIAL RESISTANCE IN BACTERIA FROM LIVESTOCK AND COMPANION ANIMALS, P521, DOI [10.1128/9781555819804.ch24, 10.1128/microbiolspec.ARBA-0009-2017]

Merrick B, 2023, INFECT DIS REP, V15, P238, DOI 10.3390/idr15030025

Parker SG, 2018, AGE AGEING, V47, P149, DOI 10.1093/ageing/afx166

Pérez-Nadales E, 2022, BMJ OPEN, V12, DOI 10.1136/bmjopen-2021-058124

Rivera-Izquierdo M, 2021, ANTIBIOTICS-BASEL, V10, DOI 10.3390/antibiotics10010089

Rivera-Izquierdo M, 2020, ANTIBIOTICS-BASEL, V9, DOI 10.3390/antibiotics9060324

Royston P, 2009, STATA J, V9, P466, DOI 10.1177/1536867X0900900308

Sociedad Espanola de Medicina Preventiva, 2022, N32 SOC ESP MED PREV

Spanish Government, 2023, NAT ANT RES PLAN

Tacconelli E, 2019, CLIN MICROBIOL INFEC, V25, P807, DOI 10.1016/j.cmi.2019.01.005

Timler Malgorzata, 2023, Int J Environ Res Public Health, V20, DOI 10.3390/ijerph20031960

van Buuren S, 2007, STAT METHODS MED RES, V16, P219, DOI 10.1177/0962280206074463

Veronese N, 2022, AGE AGEING, V51, DOI 10.1093/ageing/afac104

White IR, 2011, STAT MED, V30, P377, DOI 10.1002/sim.4067

NR 27

TC 0

Z9 0

U1 1

U2 1

PU SPRINGER HEIDELBERG

PI HEIDELBERG

PA TIERGARTENSTRASSE 17, D-69121 HEIDELBERG, GERMANY

SN 0300-8126

EI 1439-0973

J9 INFECTION

JI Infection

PD FEB

PY 2024

VL 52

IS 1

BP 231

EP 241

DI 10.1007/s15010-023-02153-1

EA DEC 2023

PG 11

WC Infectious Diseases

WE Science Citation Index Expanded (SCI-EXPANDED)

SC Infectious Diseases

GA GA4A7

UT WOS:001126684300001

PM 38109027

DA 2024-08-08

ER

PT J

AU BUTLER, RN

AF BUTLER, RN

TI GERIATRICS AND INTERNAL MEDICINE

SO ANNALS OF INTERNAL MEDICINE

LA English

DT Article

RP NIA, BLDG 31, ROOM 5C36, BETHESDA, MD 20214 USA.

CR BEESON PB, 1979, ANN INTERN MED, V90, P262, DOI 10.7326/0003-4819-90-2-262

BUTLER RN, 1977, NEW YORK STATE J MED, V77, P1470

GIBSON RM, 1978, HLTH CARE FINANC DEC

KOVAR MG, 1977, HLTH US 1976 1977

MENDENHALL RC, 1977, USCDRMED1055 U SO CA

MENDENHALL RC, 1978, USCDRMED1085 U SO CA

SPENCE DL, 1968, J AM GERIATR SOC, V16, P976, DOI 10.1111/j.1532-5415.1968.tb02727.x

RES TRAINING OPPORTU

1978, AGING MEDICAL ED

1979, DEV AGING 1978

1978, PROFILE CHRONIC ILLN

1978, JAMA, V240, P976

NR 12

TC 24

Z9 24

U1 0

U2 0

PU AMER COLL PHYSICIANS

PI PHILADELPHIA

PA INDEPENDENCE MALL WEST 6TH AND RACE ST, PHILADELPHIA, PA 19106-1572 USA

SN 0003-4819

EI 1539-3704

J9 ANN INTERN MED

JI Ann. Intern. Med.

PY 1979

VL 91

IS 6

BP 903

EP 908

DI 10.7326/0003-4819-91-6-903

PG 6

WC Medicine, General & Internal

WE Science Citation Index Expanded (SCI-EXPANDED)

SC General & Internal Medicine

GA HY332

UT WOS:A1979HY33200022

PM 517892

DA 2024-08-08

ER

PT J

AU Panaszek, B

Machaj, Z

Bogacka, E

Lindner, K

AF Panaszek, Bernard

Machaj, Zbigniew

Bogacka, Ewa

Lindner, Karolina

TI Chronic disease in the elderly A vital rationale for the revival of

internal medicine

SO POLSKIE ARCHIWUM MEDYCYNY WEWNETRZNEJ-POLISH ARCHIVES OF INTERNAL

MEDICINE

LA English

DT Review

DE chronic disease; the elderly; comorbidities; geriatrics; internal

medicine

ID OBSTRUCTIVE PULMONARY-DISEASE; CARDIOVASCULAR RISK-FACTORS; ANEMIA;

INFLAMMATION; COPD; COMORBIDITY; MARKERS; ATHEROSCLEROSIS; OSTEOPOROSIS;

PREVALENCE

AB The phenomenon of population aging has led to a significant rise in the chronic disease rate compared to other human pathologies. Elderly people are usually affected by >= 2 chronic diseases concomittantly, mainly cardiovascular, pulmonary, and central nervous system diseases, metabolic disturbances and cancer. Chronic comorbidities in elderly patients may worsen their clinical status, making both the diagnosis and treatment more difficult. Meanwhile, contemporary medicine is focused on its sub-specialties, thus turning away from the tradition of great, academic-based, general internal medicine. Clinical practice is dominated by a specific approach to a single disease rather than a patient with comorbidities. Therefore, an accurate diagnosis, ensuring effective treatment in the case of a complex and ambiguous clinical picture, is based on an attempt to combine multiple expert consultations rather than make a holistic evaluation, so characteristic of traditional internal medicine. For that reason, pathophysiology and clinical picture of a chronic disease in the elderly requires the revival of internal medicine, which is also essential to the development of geriatrics.

C1 [Panaszek, Bernard; Machaj, Zbigniew; Bogacka, Ewa; Lindner, Karolina] Univ Med, Dept Internal Dis Geriatr & Allergol, Wroclaw, Poland.

C3 Wroclaw Medical University

RP Panaszek, B (corresponding author), Akad Med Piastow Slaskich, Katedra & Klin Chorob Wewnetrznych, Wybrzeze L Pasteura 1, PL-50367 Wroclaw, Poland.

EM panaszek@alergol.am.wroc.pl

OI Lindner-Pawlowicz, Karolina/0000-0003-1732-1188

CR Agusti A, 2008, COPD, V5, P133, DOI 10.1080/15412550801941349

Agustsson T, 2007, CANCER RES, V67, P5531, DOI 10.1158/0008-5472.CAN-06-4585

Alla F, 2006, EUR J HEART FAIL, V8, P147, DOI 10.1016/j.ejheart.2005.06.008

Andrews NC, 2004, J CLIN INVEST, V113, P1251, DOI 10.1172/JCI200421441

Aniwidyaningsih W, 2008, CURR OPIN CLIN NUTR, V11, P435, DOI 10.1097/MCO.0b013e3283023d37

Argento V, 2008, J AM MED DIR ASSOC, V9, P422, DOI 10.1016/j.jamda.2008.03.002

Barbaro MPF, 2007, INT J IMMUNOPATH PH, V20, P753, DOI 10.1177/039463200702000411

Boyd CM, 2005, JAMA-J AM MED ASSOC, V294, P716, DOI 10.1001/jama.294.6.716

Brandt Nicole J, 2005, Consult Pharm, V20, P976, DOI 10.4140/TCP.n.2005.976

Brown Sue A, 2007, Curr Opin Endocrinol Diabetes Obes, V14, P436, DOI 10.1097/MED.0b013e3282f15419

Caughey GE, 2008, BMC PUBLIC HEALTH, V8, DOI 10.1186/1471-2458-8-221

Chatila Wissam M, 2008, Proc Am Thorac Soc, V5, P549, DOI 10.1513/pats.200709-148ET

de Groot V, 2003, J CLIN EPIDEMIOL, V56, P221, DOI 10.1016/S0895-4356(02)00585-1

Eisenstaedt R, 2006, BLOOD REV, V20, P213, DOI 10.1016/j.blre.2005.12.002

Fabbri LM, 2006, BREATHE, V3, P40, DOI 10.1183/18106838.0301.40

Falk Jeremy A, 2008, Proc Am Thorac Soc, V5, P543, DOI 10.1513/pats.200708-142ET

Frost RJA, 2007, EUR J ENDOCRINOL, V156, P309, DOI 10.1530/EJE-06-0614

GAJEWSKI P, 2007, POL ARCH MED WEWN, V117, P132

Hahn BH, 2008, LUPUS, V17, P368, DOI 10.1177/0961203308089989

Halpin D, 2008, COPD, V5, P187, DOI 10.1080/15412550802093041

Hankey GJ, 2006, JAMA-J AM MED ASSOC, V295, P547, DOI 10.1001/jama.295.5.547

Jawien Jacek, 2008, Pol Arch Med Wewn, V118, P127

Kalantar-Zadeh K, 2008, AM J CARDIOL, V101, p89E, DOI 10.1016/j.amjcard.2008.03.007

Kallio P, 2008, AM J CLIN NUTR, V87, P1497, DOI 10.1093/ajcn/87.5.1497

Kerem M, 2008, WORLD J GASTROENTERO, V14, P3633, DOI 10.3748/wjg.14.3633

Khosla S, 2008, ENDOCR REV, V29, P441, DOI 10.1210/er.2008-0002

Lainscak M, 2008, AM J CARDIOL, V101, p8E, DOI 10.1016/j.amjcard.2008.02.065

Mannino DM, 2006, EUR RESPIR J, V27, P627, DOI 10.1183/09031936.06.00024605

Martinez-Hervas S, 2008, EUR J INTERN MED, V19, P209, DOI 10.1016/j.ejim.2007.09.005

Montagnana Martina, 2008, Recenti Progressi in Medicina, V99, P215

Movahed Mohammad-Reza, 2007, Congest Heart Fail, V13, P78, DOI 10.1111/j.1527-5299.2007.888138.x

Mundy GR, 2007, NUTR REV, V65, pS147, DOI 10.1301/nr.2007.dec.S147-S151

Murphy CL, 2008, HEART FAIL REV, V13, P431, DOI 10.1007/s10741-008-9085-y

Penninx BWJH, 2004, J AM GERIATR SOC, V52, P719, DOI 10.1111/j.1532-5415.2004.52208.x

Prakash J, 2006, INT UROL NEPHROL, V38, P821, DOI 10.1007/s11255-006-9003-y

Rennard SI, 2007, EUR RESPIR REV, V16, P91, DOI 10.1183/09059180.00010502

Sarkar M, 2015, CLIN MED INSIGHTS-CI, V9, P5, DOI 10.4137/CCRPM.S22803

Sharma M, 2008, BASIC CLIN PHARMACOL, V103, P25, DOI 10.1111/j.1742-7843.2008.00215.x

Sin DD, 2006, EUR RESPIR J, V28, P1245, DOI 10.1183/09031936.00133805

Tarmonova L Iu, 2007, Klin Med (Mosk), V85, P26

Van Helvoort HAC, 2006, MED SCI SPORT EXER, V38, P1543, DOI 10.1249/01.mss.0000228331.13123.53

van Weel C, 2006, LANCET, V367, P550, DOI 10.1016/S0140-6736(06)68198-1

Wislowska M, 2008, RHEUMATOL INT, V28, P513, DOI 10.1007/s00296-007-0473-8

Woloshin S, 2008, J NATL CANCER I, V100, P845, DOI 10.1093/jnci/djn124

NR 44

TC 11

Z9 14

U1 0

U2 9

PU MEDYCYNA PRAKTYCZNA SP K SP ZOO

PI KRAKOW

PA REJTANA 2, KRAKOW, 30-510 AP, POLAND

SN 0032-3772

J9 POL ARCH MED WEWN

JI Pol. Arch. Med. Wewn.

PD APR

PY 2009

VL 119

IS 4

BP 248

EP 253

DI 10.20452/pamw.668

PG 6

WC Medicine, General & Internal

WE Science Citation Index Expanded (SCI-EXPANDED)

SC General & Internal Medicine

GA 478NT

UT WOS:000268595700011

PM 19413185

DA 2024-08-08

ER

PT J

AU IRVINE, RE

AF IRVINE, RE

TI GERIATRIC-MEDICINE AND GENERAL INTERNAL MEDICINE

SO JOURNAL OF THE ROYAL COLLEGE OF PHYSICIANS OF LONDON

LA English

DT Article

RP IRVINE, RE (corresponding author), ST HELENS HOSP,DEPT MED ELDERLY,HASTINGS,ENGLAND.

NR 0

TC 12

Z9 13

U1 0

U2 0

PU ROY COLL PHYS LONDON EDITORIAL OFFICE

PI LONDON

PA 11 ST ANDREWS PLACE REGENTS PARK, LONDON, ENGLAND NW1 4LE

SN 0035-8819

J9 J ROY COLL PHYS LOND

JI J. R. Coll. Physicians Lond.

PY 1984

VL 18

IS 1

BP 21

EP 24

PG 4

WC Medicine, General & Internal

WE Science Citation Index Expanded (SCI-EXPANDED)

SC General & Internal Medicine

GA RZ587

UT WOS:A1984RZ58700003

PM 6707981

DA 2024-08-08

ER

PT J

AU Nardi, R

Scanelli, G

Corrao, S

Iori, I

Mathieu, G

Amatrian, RC

AF Nardi, Roberto

Scanelli, Giovanni

Corrao, Salvatore

Iori, Ido

Mathieu, Giovanni

Amatrian, Roberto Cataldi

TI Co-morbidity does not reflect complexity in internal medicine patients

SO EUROPEAN JOURNAL OF INTERNAL MEDICINE

LA English

DT Review

DE co-morbidity; internal medicine; complexity; elderly

ID COMPREHENSIVE GERIATRIC ASSESSMENT; ADMINISTRATIVE DATA; OLDER PATIENTS;

HEALTH-CARE; COMORBIDITY MEASURES; CASE-MIX; MORTALITY; FRAILTY; INDEX;

INFORMATION

AB Internal medicine patients are mostly elderly; they have multiple co-morbidities, which are usually chronic, rather than self-limiting or acute diseases. Neither administrative indicators nor co-morbidity indexes, though validated in elderly patients, are able to completely define these "complex" patients or to allow physicians to correctly "cope" with them. For the complex patients found in internal medicine wards, internists need not only to find the best diagnosis and treatment, but also to apply a complex intervention (i.e., a comprehensive assessment and both continuous and multi-disciplinary care) in order to maintain their health and ability to function and to prevent or delay disability, frailty, and displacement from home and community. The aim of this review is to underscore the differences between the concepts of co-morbidity and complexity, to discuss instruments for their measurement, and to highlight related implications, areas of uncertainty, and the responsibilities of internists in the assessment and management of inpatients of their wards. The conclusion we come to is that it is mandatory to shift from a finance/administrative-based management system to a clinical process model (clinical governance) driven by the quality of the medical outcome and the cost of achieving that outcome. From a "complexity theory" standpoint, patient-centered care and collaboration can be seen as simple rules that guide desirable behaviors in a complex system. By exploring the real complexity of our patients, we exercise the holistic, anthropologic medicine of the person that is internal medicine. (C) 2007 European Federation of Internal Medicine. Published by Elsevier B.V. All rights reserved.

C1 Osped G Dossetti Bazzano, Azienda USL Bologna, UOC Med Interna, Bologna, Italy.

Azienda Osped Univ Ferrara Arcispedale Sant Anna, UOC Med Interna Osped, Modena, Italy.

Univ Palermo, Dipartimento Biomed Med Interna & Specialist, I-90133 Palermo, Italy.

Osped E Agnelli Pinerolo Torino, UOC Med Interna, Turin, Italy.

Univ Salvador, Int Coll Internal Med, Buenos Aires, DF, Argentina.

C3 AUSL di Bologna; University of Ferrara; Arcispedale Sant'Anna;

University of Palermo

RP Nardi, R (corresponding author), Viale Martiri 10 b, I-40053 Bazzano, Italy.

EM r.nardi@auslbosud.emr.it

RI Corrao, Salvatore/AAQ-5768-2020

OI Corrao, Salvatore/0000-0001-5621-1374

CR Alarcón T, 1999, AGE AGEING, V28, P429, DOI 10.1093/ageing/28.5.429

Anderson G., 2002, Chronic conditions: Making the case for ongoing care

[Anonymous], 2005, LANCET, V365, P1440

[Anonymous], 2004, G GERONTOL

BARABASI AL, 2003, LINKED EVERYTHING CO

BARR DP, 1947, ANN INTERN MED, V27, P195, DOI 10.7326/0003-4819-27-2-195

Beers M.H., 2000, The Merck Manual of Geriatrics, V3rd

BELLIS P, 2004, GRUPPO MINERAVA PROG

Bierman AS, 1999, WOMEN HEALTH ISS, V9, P2, DOI 10.1016/S1049-3867(98)00035-8

Bo M, 2003, J AM GERIATR SOC, V51, P529, DOI 10.1046/j.1532-5415.2003.51163.x

Boyd CM, 2005, JAMA-J AM MED ASSOC, V294, P716, DOI 10.1001/jama.294.6.716

BRITT H, 2004, GEN PRACTICE ACTIVIT

*CAN I HLTH INF PA, 2005, WHIC CHR DIS MAN INF

Caplan GA, 2004, J AM GERIATR SOC, V52, P1417, DOI 10.1111/j.1532-5415.2004.52401.x

CHARLSON ME, 1987, J CHRON DIS, V40, P373, DOI 10.1016/0021-9681(87)90171-8

Covinsky KE, 1997, J GEN INTERN MED, V12, P203, DOI 10.1007/s11606-006-5041-5

Crabtree BF, 2001, J FAM PRACTICE, V50, P881

de Groot V, 2003, J CLIN EPIDEMIOL, V56, P221, DOI 10.1016/S0895-4356(02)00585-1

DEYO RA, 1992, J CLIN EPIDEMIOL, V45, P613, DOI 10.1016/0895-4356(92)90133-8

Elixhauser A, 1998, MED CARE, V36, P8, DOI 10.1097/00005650-199801000-00004

*EUR FED INT MED, 2001, VNIT LEK, V47, P416

Evans T, 1987, SOFTWARE QUALITY ASS

Fisher AL, 2005, J AM GERIATR SOC, V53, P2229, DOI 10.1111/j.1532-5415.2005.00510.x

FLUGELMAN MY, 1986, GERONTOLOGY, V32, P272, DOI 10.1159/000212801

Fried LP, 2004, J GERONTOL A-BIOL, V59, P255

Gijsen R, 2001, J CLIN EPIDEMIOL, V54, P661, DOI 10.1016/S0895-4356(00)00363-2

Glouberman S., 2002, Complicated and Complex Systems: What Would Successful Reform of Medicare Look Like?

Gray LK, 2002, J AM GERIATR SOC, V50, P1955, DOI 10.1046/j.1532-5415.2002.50606.x

GREENFIELD S, 1995, MED CARE, V33, pAS47

GREENFIELD S, 1991, ASSESSMENT COMORBIDI, P22

Guralnik JM, 1996, ANN EPIDEMIOL, V6, P376, DOI 10.1016/S1047-2797(96)00060-9

GURALNIK JM, 1989, AGING 80 PREVALENCE

Hammerly M, 2002, AM J MED QUAL, V17, P33, DOI 10.1177/106286060201700107

Harboun M, 2001, REV EPIDEMIOL SANTE, V49, P287

HOFF T, 2003, POWER FRONTLINE WORK

*HONG KONG GER SOC, 2005, BUILD HLTH TOM

Incalzi RA, 1997, J INTERN MED, V242, P291, DOI 10.1046/j.1365-2796.1997.00132.x

John R, 2003, GERONTOLOGIST, V43, P649, DOI 10.1093/geront/43.5.649

Jones DM, 2004, J AM GERIATR SOC, V52, P1929, DOI 10.1111/j.1532-5415.2004.52521.x

Kane R L, 2000, J Aging Soc Policy, V11, P107, DOI 10.1300/J031v11n02_12

Kane Robert L, 2005, J Aging Soc Policy, V17, P1, DOI 10.1300/J031v17n04_01

KAPLAN MH, 1974, J CHRON DIS, V27, P387, DOI 10.1016/0021-9681(74)90017-4

Kucharz Eugene J., 2003, Eur J Intern Med, V14, P272, DOI 10.1016/S0953-6205(03)00066-9

Kurtz CF, 2003, IBM SYST J, V42, P462, DOI 10.1147/sj.423.0462

LINN BS, 1968, J AM GERIATR SOC, V16, P622, DOI 10.1111/j.1532-5415.1968.tb02103.x

Martin CM, 2005, MED J AUSTRALIA, V183, P106, DOI 10.5694/j.1326-5377.2005.tb06943.x

Mitnitski A, 2005, J AM GERIATR SOC, V53, P2184, DOI 10.1111/j.1532-5415.2005.00506.x

Mitnitski Arnold B, 2002, BMC Geriatr, V2, P1

Ouwens M, 2005, INT J QUAL HEALTH C, V17, P141, DOI 10.1093/intqhc/mzi016

Perkins AJ, 2004, J CLIN EPIDEMIOL, V57, P1040, DOI 10.1016/j.jclinepi.2004.03.002

Piccirillo JF, 2004, JAMA-J AM MED ASSOC, V291, P2441, DOI 10.1001/jama.291.20.2441

Pilotto A, 2004, G GERONTOL, V52, P262

Plsek PE, 2001, BRIT MED J, V323, P625, DOI 10.1136/bmj.323.7313.625

Powell H, 2001, J CLIN EPIDEMIOL, V54, P687, DOI 10.1016/S0895-4356(00)00364-4

*PROG FIN MIN SANT, 2001, LIN GUID SULL UT VAL

Quan HD, 2002, MED CARE, V40, P675, DOI 10.1097/00005650-200208000-00007

Quan HD, 2005, MED CARE, V43, P1130, DOI 10.1097/01.mlr.0000182534.19832.83

Rehman H U, 2005, Eur J Intern Med, V16, P385, DOI 10.1016/j.ejim.2005.08.001

Robertson RG, 2004, AM FAM PHYSICIAN, V70, P343

Rockwood BL, 1998, SEM HUM SCI, V12, P189

Rockwood K, 2004, AGE AGEING, V33, P429, DOI 10.1093/ageing/afh153

Rockwood K, 2000, J AM GERIATR SOC, V48, P1080, DOI 10.1111/j.1532-5415.2000.tb04783.x

Roos LL, 1997, HEALTH SERV RES, V32, P229

ROZMAN C, 2005, CURR FUT STAT INT ME

Rozzini R, 2002, AGE AGEING, V31, P277, DOI 10.1093/ageing/31.4.277

Rubenstein LZ, 2004, J GERONTOL A-BIOL, V59, P473

RUBENSTEIN LZ, 1984, NEW ENGL J MED, V311, P1664, DOI 10.1056/NEJM198412273112604

Rubenstein LZ., 1995, Geriatric assessment technology: The state of art

SENIN U, 2004, 49 C NAZ SIGG NOV 4

SENIN U, FRAIL ELDERLY NEW CL

Shwartz M, 1996, MED CARE, V34, P767, DOI 10.1097/00005650-199608000-00005

Sloan KL, 2003, MED CARE, V41, P761, DOI 10.1097/00005650-200306000-00009

SMITH R, 2005, NHS CONCEPTION MODEL

Starfield Barbara, 2003, Ann Fam Med, V1, P8, DOI 10.1370/afm.1

STUCK AE, 1993, LANCET, V342, P1032, DOI 10.1016/0140-6736(93)92884-V

Stukenborg GJ, 2001, MED CARE, V39, P727, DOI 10.1097/00005650-200107000-00009

van den Akker M, 2001, J CLIN EPIDEMIOL, V54, P675, DOI 10.1016/S0895-4356(00)00358-9

Van Den Akker M, 1996, Eur J Gen Pract, V2, P65, DOI [10.3109/13814789609162146, DOI 10.3109/13814789609162146]

VANDERSTRICHT O, 1898, ARCH BIOL, V15, P367

VERBRUGGE LM, 1991, J GERONTOL, V46, pS71, DOI 10.1093/geronj/46.2.S71

VERBRUGGE LM, 1989, MILBANK Q, V67, P450, DOI 10.2307/3350223

Weiner M, 2003, ANN INTERN MED, V139, P430, DOI 10.7326/0003-4819-139-5_Part_2-200309021-00010

Wilson T, 2001, BMJ-BRIT MED J, V323, P685, DOI 10.1136/bmj.323.7314.685

Zhang JX, 2003, J AM GERIATR SOC, V51, P476, DOI 10.1046/j.1532-5415.2003.51155.x

NR 84

TC 86

Z9 99

U1 0

U2 11

PU ELSEVIER SCIENCE BV

PI AMSTERDAM

PA PO BOX 211, 1000 AE AMSTERDAM, NETHERLANDS

SN 0953-6205

EI 1879-0828

J9 EUR J INTERN MED

JI Eur. J. Intern. Med.

PD SEP

PY 2007

VL 18

IS 5

BP 359

EP 368

DI 10.1016/j.ejim.2007.05.002

PG 10

WC Medicine, General & Internal

WE Science Citation Index Expanded (SCI-EXPANDED)

SC General & Internal Medicine

GA 216VW

UT WOS:000249908100003

PM 17693224

OA Green Published

DA 2024-08-08

ER

PT J

AU Díez-Manglano, J

García, JLC

Calvo, EGA

Saínz, AJ

Beguería, EC

Martínez-Alvarez, RM

Tello, EB

Martínez, AC

AF Diez-Manglano, Jesus

Cabrerizo Garcia, Jose Luis

Garcia-Arilla Calvo, Ernesto

Jimeno Sainz, Araceli

Calvo Begueria, Eva

Martinez-Alvarez, Rosa M.

Bejarano Tello, Esperanza

Caudevilla Martinez, Aranzazu

TI External validation of the PROFUND index in polypathological patients

from internal medicine and acute geriatrics departments in Aragn

SO INTERNAL AND EMERGENCY MEDICINE

LA English

DT Article

DE Polypathological patient; Multimorbidity; Mortality; Geriatrics;

Internal medicine

ID MULTIDIMENSIONAL PROGNOSTIC INDEX; OLDER-ADULTS; 1-YEAR MORTALITY;

FEATURES; SCALE; MODEL; TOOL

AB The objective of the study was to validate externally and prospectively the PROFUND index to predict survival of polypathological patients after a year. An observational, prospective and multicenter study was performed. Polypathological patients admitted to an internal medicine or geriatrics department and attended by investigators consecutively between March 1 and June 30, 2011 were included. Data concerning age, gender, comorbidity, Barthel and Lawton-Brody indexes, Pfeiffer questionnaire, socio-familial Gijon scale, delirium, number of drugs and number of admissions during the previous year were gathered for each patient. The PROFUND index was calculated. The follow-up lasted 1 year. A Cox proportional regression model was calculated, and was used to analyze the association of the variables to mortality and C-statistic. 465 polypathological patients, 333 from internal medicine and 132 from geriatrics, were included. One-year mortality is associated with age [hazard ratio (HR) 1.52 95 % CI 1.04-2.12; p = 0.01], presence of neoplasia [HR 2.68 95 % CI 1.71-4.18; p = 0.0001] and dependence for basic activities of daily living [HR 2.34 95 % CI 1.61-3.40; p = 0.0009]. In predicting mortality, the PROFUND index shows good discrimination in patients from internal medicine (C-statistics 0.725 95 % CI 0.670-0.781), but a poor one in those from geriatrics (0.546 95 % CI 0.448-0.644). The PROFUND index is a reliable tool for predicting mortality in internal medicine PP patients.

C1 [Diez-Manglano, Jesus; Martinez-Alvarez, Rosa M.; Bejarano Tello, Esperanza] Hosp Royo Villanova, Dept Internal Med, Zaragoza 50015, Spain.

[Diez-Manglano, Jesus; Jimeno Sainz, Araceli; Caudevilla Martinez, Aranzazu] Aragon Hlth Sci Inst, Res Grp Comorbid & Polypathol Aragon, Zaragoza, Spain.

[Diez-Manglano, Jesus; Martinez-Alvarez, Rosa M.] Univ Zaragoza, Sch Med, Dept Med Dermatol & Psychiat, Zaragoza, Spain.

[Cabrerizo Garcia, Jose Luis] Hosp Def, Dept Internal Med, Zaragoza, Spain.

[Garcia-Arilla Calvo, Ernesto] Hosp Nuestra Senora Gracia, Dept Geriatr, Zaragoza 50003, Spain.

[Jimeno Sainz, Araceli; Caudevilla Martinez, Aranzazu] Hosp Ernest Lluch, Dept Internal Med, Calatayud, Spain.

[Calvo Begueria, Eva] Hosp San Jorge, Dept Internal Med, Huesca, Spain.

C3 University of Zaragoza; Hospital Universidad San Jorge

RP Díez-Manglano, J (corresponding author), Hosp Royo Villanova, Dept Internal Med, Avda San Gregorio 30, Zaragoza 50015, Spain.

EM jdiez@aragon.es; egarciaarilla@salud.aragon.es

RI Díez-Manglano, J/AAC-3198-2020

OI Díez-Manglano, J/0000-0002-3132-2171; Garces Horna,

Vanesa/0000-0002-3485-0789

CR Alarcon M, 1998, Rev Esp Geriatr Gerontol, V33, P178

Amici A, 2008, ARCH GERONTOL GERIAT, V46, P327, DOI 10.1016/j.archger.2007.05.007

Amici A, 2011, ARCH GERONTOL GERIAT, V52, pE60, DOI 10.1016/j.archger.2010.05.015

[Anonymous], 2007, PROCESO ASISTENCIAL

Aragonese Statistics Institute, AR BAS DAT

Bernabeu-Wittel M, 2011, EUR J INTERN MED, V22, P311, DOI 10.1016/j.ejim.2010.11.012

Celli BR, 2004, NEW ENGL J MED, V350, P1005, DOI 10.1056/NEJMoa021322

CHARLSON ME, 1987, J CHRON DIS, V40, P373, DOI 10.1016/0021-9681(87)90171-8

de la Iglesia JM, 2001, MED CLIN-BARCELONA, V117, P129

Díez-Manglano J, 2013, EUR J INTERN MED, V24, P767, DOI 10.1016/j.ejim.2013.07.010

Dramé M, 2008, EUR J EPIDEMIOL, V23, P783, DOI 10.1007/s10654-008-9290-y

García-Morillo JS, 2005, MED CLIN-BARCELONA, V125, P5, DOI 10.1157/13076399

LAWTON MP, 1969, GERONTOLOGIST, V9, P9, DOI 10.1093/geront/9.1.9

Levine SK, 2007, AM J MED, V120, P455, DOI 10.1016/j.amjmed.2006.09.021

Levy WC, 2006, CIRCULATION, V113, P1424, DOI 10.1161/CIRCULATIONAHA.105.584102

MAHONEY F I, 1965, Md State Med J, V14, P61

Pilotto A, 2008, REJUV RES, V11, P151, DOI 10.1089/rej.2007.0569

Pilotto A, 2009, BEST PRACT RES CL GA, V23, P829, DOI 10.1016/j.bpg.2009.10.001

Pilotto A, 2009, J ALZHEIMERS DIS, V18, P191, DOI 10.3233/JAD-2009-1139

Ramírez-Duque N, 2008, REV CLIN ESP, V208, P4, DOI 10.1157/13115000

Sancarlo D, 2012, J NEUROL, V259, P670, DOI 10.1007/s00415-011-6241-4

Walter LC, 2001, JAMA-J AM MED ASSOC, V285, P2987, DOI 10.1001/jama.285.23.2987

Yourman LC, 2012, JAMA-J AM MED ASSOC, V307, P182, DOI 10.1001/jama.2011.1966

NR 23

TC 15

Z9 15

U1 0

U2 3

PU SPRINGER-VERLAG ITALIA SRL

PI MILAN

PA VIA DECEMBRIO, 28, MILAN, 20137, ITALY

SN 1828-0447

EI 1970-9366

J9 INTERN EMERG MED

JI Intern. Emerg. Med.

PD DEC

PY 2015

VL 10

IS 8

BP 915

EP 926

DI 10.1007/s11739-015-1252-2

PG 12

WC Medicine, General & Internal

WE Science Citation Index Expanded (SCI-EXPANDED)

SC General & Internal Medicine

GA CW8US

UT WOS:000365275500005

PM 25986479

DA 2024-08-08

ER

PT J

AU Landefeld, CS

Callahan, CM

Woolard, N

AF Landefeld, CS

Callahan, CM

Woolard, N

TI General internal medicine and geriatrics: Building a foundation to

improve the training of general internists in the care of older adults

SO ANNALS OF INTERNAL MEDICINE

LA English

DT Article

ID PROGRAMS; 21ST-CENTURY; RESIDENTS; FACULTY

AB Internists-"doctors for adults"-provide most of the medical care given to older Americans, especially those with serious chronic disease. Nonetheless, the United States lacks an adequate physician workforce with mastery in caring for older persons and with expertise in building knowledge about how best to provide this care. This supplement aims to strengthen the physician workforce by fostering incremental and sustained improvements in the training of internal medicine residents in the care of older adults and in the development of geriatrics-oriented general internal medicine faculty. It identifies 3 major barriers to these improvements: lack of adequately trained teachers and mentors, the belief that explicit training in geriatrics has little to offer the generalist, and inadequate funding. Three strategies offer particular promise in over-coming these barriers: engaging directors of internal medicine residency programs, funding centers to promote collaboration between teaching and research programs in general internal medicine and geriatrics, and providing substantial incremental funding on the national level to pay for the time required to care for frail older patients and to teach and do research about this care. The barriers and strategies identified in this supplement may also inform efforts to enhance the skills of practicing physicians and improve training and faculty development in family medicine and other disciplines.

C1 Univ Calif San Francisco, San Francisco, CA 94143 USA.

San Francisco VA Med Ctr, San Francisco, CA USA.

Regenstrief Inst Hlth Care, Indianapolis, IN 46202 USA.

Indiana Univ, Indianapolis, IN 46204 USA.

Wake Forest Univ, Sch Med, Winston Salem, NC 27109 USA.

C3 University of California System; University of California San Francisco;

US Department of Veterans Affairs; Veterans Health Administration (VHA);

San Francisco VA Medical Center; Regenstrief Institute Inc; Indiana

University System; Indiana University Indianapolis; Wake Forest

University

RP Univ Calif San Francisco, Box 1265, San Francisco, CA 94143 USA.

EM sethl@medicine.ucsf.edu

FU NIA NIH HHS [K07 AG000912, K-07 AG00912] Funding Source: Medline

CR BURTLER RN, 1975, WHY SURVIVE BEING OL

Hazzard WR, 2001, AM J MED, V110, P507, DOI 10.1016/S0002-9343(01)00685-4

HAZZARD WR, 1994, AM J MED, pS1

*I MED, 1978, AG MED ED REP STUD

*I MED COMM LEAD A, 1987, ACAD GER YEAR 2000

*INT LONG CTR, 2002, NAT CRIS NEED GER FA

JAHNIGEN D, 1994, AM J MED, V97, P43

Kennedy J, 1998, INT J HEALTH SERV, V28, P757, DOI 10.2190/3C1X-TQAE-7UDM-2NWQ

Larson EB, 2001, ANN INTERN MED, V134, P997, DOI 10.7326/0003-4819-134-10-200105150-00013

LIBOW LS, 1990, J AM GERIATR SOC, V38, P79, DOI 10.1111/j.1532-5415.1990.tb01603.x

Medina-Walpole A, 2002, J AM GERIATR SOC, V50, P949, DOI 10.1046/j.1532-5415.2002.50225.x

*NAT CTR HLTH STAT, DAT WAR TRENDS HLTH

REUBEN DB, 1993, J AM GERIATR SOC, V41, P560, DOI 10.1111/j.1532-5415.1993.tb01896.x

REUBEN DB, 1993, J AM GERIATR SOC, V41, P444, DOI 10.1111/j.1532-5415.1993.tb06955.x

Rubin CD, 2003, ANN INTERN MED, V139, P615, DOI 10.7326/0003-4819-139-7-200310070-00035

Simon SR, 2003, ANN INTERN MED, V139, P621, DOI 10.7326/0003-4819-139-7-200310070-00036

Solomon DH, 2002, J AM GERIATR SOC, V50, P5, DOI 10.1046/j.1532-5415.2002.50038.x

Sox HC, 2001, AM J MED, V110, P745, DOI 10.1016/S0002-9343(01)00756-2

Thomas DC, 2003, ANN INTERN MED, V139, P628, DOI 10.7326/0003-4819-139-7-200310070-00037

*US DEP HHS ADM AG, 2001, PROF OLD AM

Warshaw G, 2003, FAM MED, V35, P24

Warshaw G.A., 2002, Geriatric medicine training and practice in the United States at the beginning of the 21st century

Warshaw GA, 2002, JAMA-J AM MED ASSOC, V288, P2313, DOI 10.1001/jama.288.18.2313

NR 23

TC 40

Z9 41

U1 0

U2 0

PU AMER COLL PHYSICIANS

PI PHILADELPHIA

PA INDEPENDENCE MALL WEST 6TH AND RACE ST, PHILADELPHIA, PA 19106-1572 USA

SN 0003-4819

EI 1539-3704

J9 ANN INTERN MED

JI Ann. Intern. Med.

PD OCT 7

PY 2003

VL 139

IS 7

BP 609

EP 614

DI 10.7326/0003-4819-139-7-200310070-00034

PG 6

WC Medicine, General & Internal

WE Science Citation Index Expanded (SCI-EXPANDED)

SC General & Internal Medicine

GA 729AP

UT WOS:000185748800026

PM 14530244

DA 2024-08-08

ER

PT J

AU Wada, T

Harada, T

Ikeda, K

Hiroshige, J

AF Wada, Takahide

Harada, Taku

Ikeda, Keiichiro

Hiroshige, Juichi

TI Spontaneous spinal epidural haematoma due to polypharmacy including

multiple antiplatelet agents

SO POSTGRADUATE MEDICAL JOURNAL

LA English

DT Article

DE Internal medicine; geriatric medicine

C1 [Wada, Takahide] Showa Univ, Koto Toyosu Hosp, Dept Neurol, Tokyo, Japan.

[Harada, Taku; Ikeda, Keiichiro; Hiroshige, Juichi] Showa Univ, Koto Toyosu Hosp, Gen Med, Tokyo, Japan.

[Harada, Taku] Dokkyo Med Univ Hosp, Diagnost & Generalist Med, Mibu, Tochigi, Japan.

C3 Showa University; Showa University; Dokkyo Medical University

RP Harada, T (corresponding author), Showa Med Univ Hosp, Div Gen Med, Shinagawa Ku, 1-5-8 Hatanodai, Tokyo 1425555, Japan.

EM hrdtaku@gmail.com

OI harada, taku/0000-0001-8794-6744

CR Chen YC, 2014, EUR J INTERN MED, V25, P49, DOI 10.1016/j.ejim.2013.10.006

Dimou J, 2010, J CLIN NEUROSCI, V17, P142, DOI 10.1016/j.jocn.2009.03.021

Figueroa Jessica, 2017, J Spine Surg, V3, P58, DOI 10.21037/jss.2017.02.04

Thiele RH, 2008, NEUROCRIT CARE, V9, P242, DOI 10.1007/s12028-008-9083-x

Wang LS, 2012, J ORTHOP SURG-HONG K, V20, P386, DOI 10.1177/230949901202000326

NR 5

TC 0

Z9 0

U1 0

U2 0

PU BMJ PUBLISHING GROUP

PI LONDON

PA BRITISH MED ASSOC HOUSE, TAVISTOCK SQUARE, LONDON WC1H 9JR, ENGLAND

SN 0032-5473

EI 1469-0756

J9 POSTGRAD MED J

JI Postgrad. Med. J.

PD AUG

PY 2021

VL 97

IS 1150

BP 487

EP 488

DI 10.1136/postgradmedj-2020-137755

PG 2

WC Medicine, General & Internal

WE Science Citation Index Expanded (SCI-EXPANDED)

SC General & Internal Medicine

GA WP9FV

UT WOS:000713431500005

PM 32576642

DA 2024-08-08

ER

PT J

AU Maybee, C

Nguyen, NT

Chan, M

Chan, E

Wei, C

Quan, T

Wei, C

Gu, A

Roth, KE

AF Maybee, Camilla

Nguyen, Nam Tran

Chan, Melissa

Chan, Emily

Wei, Chapman

Quan, Theodore

Wei, Chaplin

Gu, Alex

Roth, Katalin E.

TI Assessing the Accessibility of Content for Geriatric Fellowship Programs

SO JOURNAL OF THE AMERICAN GERIATRICS SOCIETY

LA English

DT Article

DE geriatrics; fellowship content; accessibility

ID RESIDENCY; WEBSITES

AB The information available on program websites concerning geriatric fellowships in internal medicine and family medicine is a crucial factor in generating applicants' interest in individual programs. Our study aimed to quantify the accessibility and quality of information available on accredited geriatric (family medicine and internal medicine) fellowship program websites and further analyze the implications of the results obtained. A list of geriatric (family medicine and internal medicine) fellowship programs was analyzed through quantified measures after being verified for accreditation. Certain criteria were evaluated for each of these programs, such as website accessibility and whether critical information was available on online program websites. These criteria were centered on academic, administrative, and application-based factors. Hundred and fifty eight Family Medicine and Internal Medicine geriatric fellowship programs were identified in total, of which only 150 were accredited by the Accreditation Council for Graduate Medical Education and considered for analysis. Of these, 20 (13.33%) programs had website links that were nonfunctional and only 145 programs had websites at all. On programs' websites, information regarding aspects such as contact information-including phone number or email for the program-were lacking. Other information regarding past and current fellows, research, and curriculum were also generally lacking. Geriatric Fellowship websites in Family Medicine and Internal Medicine can gain better traction from those interested in applying for their programs by updating information more often and providing more and better information concerning critical aspects of the programs themselves online.

C1 [Maybee, Camilla; Nguyen, Nam Tran; Chan, Melissa; Chan, Emily; Wei, Chapman; Quan, Theodore; Wei, Chaplin; Roth, Katalin E.] George Washington Univ, Sch Med & Hlth Sci, Dept Med, Div Geriatr & Palliat Med, Washington, DC 20052 USA.

[Gu, Alex] George Washington Univ, Sch Med, Dept Orthopaed Surg, Washington, DC USA.

C3 George Washington University; George Washington University

RP Wei, C (corresponding author), George Washington Univ, Sch Med & Hlth Sci, Dept Med, Div Geriatr & Palliat Med, Washington, DC 20052 USA.

EM cwei@gwu.edu

OI Gu, Alex/0000-0002-4814-0796; Wei, Chapman/0000-0002-2004-5412

CR Accreditation Council for Graduate Medical Education, ACGME DATA RESOURCE

Anderi E, 2020, CUREUS J MED SCIENCE, V12, DOI 10.7759/cureus.9593

[Anonymous], RES DAT SPEC MATCH S

Chu LF, 2011, ANESTH ANALG, V112, P430, DOI 10.1213/ANE.0b013e3182027a94

Daneshpayeh Negin, 2013, J Educ Perioper Med, V15, pE066

Embi PJ, 2003, J MED INTERNET RES, V5, DOI 10.2196/jmir.5.3.e22

Gaeta TJ, 2005, ACAD EMERG MED, V12, P89, DOI 10.1197/j.aem.2004.08.047

Gu A, 2018, J ARTHROPLASTY, V33, P1630, DOI 10.1016/j.arth.2018.01.075

Hu JY, 2016, PLAST SURG-CHIR PLAS, V24, P187, DOI 10.1177/229255031602400301

Huang BY, 2017, J VASC SURG, V66, P1892, DOI 10.1016/j.jvs.2017.08.064

Krebs VE, 2018, J ARTHROPLASTY, V33, P1629, DOI 10.1016/j.arth.2018.02.081

Long Timothy, 2017, J Educ Perioper Med, V19, pE604

Petriceks AH, 2018, GERONTOL GERIATR MED, V4, DOI 10.1177/2333721418777659

Shaath MK, 2020, J AM ACAD ORTHOP SUR, V28, pE1105, DOI 10.5435/JAAOS-D-19-00804

NR 14

TC 2

Z9 2

U1 0

U2 0

PU WILEY

PI HOBOKEN

PA 111 RIVER ST, HOBOKEN 07030-5774, NJ USA

SN 0002-8614

EI 1532-5415

J9 J AM GERIATR SOC

JI J. Am. Geriatr. Soc.

PD JAN

PY 2021

VL 69

IS 1

BP 197

EP 200

DI 10.1111/jgs.16948

EA NOV 2020

PG 4

WC Geriatrics & Gerontology; Gerontology

WE Science Citation Index Expanded (SCI-EXPANDED); Social Science Citation Index (SSCI)

SC Geriatrics & Gerontology

GA PS2FD

UT WOS:000593819400001

PM 33251578

DA 2024-08-08

ER

PT J

AU Di Bari, M

Carreras, G

Giordano, A

Degli Esposti, L

Buda, S

Michelozzi, P

Bernabei, R

Marchionni, N

Balzi, D

AF Di Bari, Mauro

Carreras, Giulia

Giordano, Antonella

Degli Esposti, Luca

Buda, Stefano

Michelozzi, Paola

Bernabei, Roberto

Marchionni, Niccolo

Balzi, Daniela

TI Long-term Survival After Hospital Admission in Older Italians:

Comparison Between Geriatrics and Internal Medicine Across Different

Discharge Diagnoses and Risk Status

SO JOURNALS OF GERONTOLOGY SERIES A-BIOLOGICAL SCIENCES AND MEDICAL

SCIENCES

LA English

DT Article

DE Dynamic Silver Code; Emergency Department; Geriatric model of care

ID EMERGENCY-DEPARTMENT; PROGNOSTIC STRATIFICATION; ADMINISTRATIVE DATA;

ELDERLY-PATIENTS; FRAILTY INDEX; OUTCOMES; VALIDATION; ADULTS; UNIT;

IDENTIFICATION

AB Background: In randomized clinical trials, compared to Internal Medicine, admission to Geriatrics improved clinical outcomes of frail older patients accessing the Emergency Department (ED). Whether this advantage is maintained also in the "real world" is uncertain. We compared long-term survival of patients admitted to Geriatrics or Internal Medicine wards after stratification for background risk and across a variety of discharge diagnoses.

Method: Data were derived from the "Silver Code National Project," an observational study of 180,079 unselected 75+ years old persons, admitted via the ED to Internal Medicine (n = 169,717, 94.2%) or Geriatrics (n = 10,362) wards in Italy. The Dynamic Silver Code (DSC), based on administrative data, was applied to balance for background risk between participants admitted to Geriatrics or Internal Medicine.

Results: One-year mortality was 33.7%, lower in participants discharged from Geriatrics (32.1%) than from Internal Medicine (33.8%; p < .001), and increased progressively across four DSC risk classes (p < .001). Admission to Geriatrics was associated with survival advantage in DSC class II to IV participants, with HR (95% CI) of 0.88 (0.83-0.94), 0.86 (0.80-0.92), and 0.92 (0.86-0.97), respectively. Cerebrovascular diseases, cognitive disorders, and heart failure were the discharge diagnoses with the widest survival benefit from admission to Geriatrics, which was mostly observed in DSC class III.

Conclusions: Admission to Geriatrics may provide long-term survival benefit in subjects who, based on the DSC, may be considered at an intermediate risk. Specific clinical conditions should be considered in the ED to improve selection of patients to be targeted for Geriatrics admission.

C1 [Di Bari, Mauro; Carreras, Giulia; Giordano, Antonella; Marchionni, Niccolo] Univ Florence, Dept Expt & Clin Med, Res Unit Med Aging, Viale G Pieraccini 6, I-50139 Florence, Italy.

[Di Bari, Mauro] Azienda Osped Univ Careggi, Dept Med & Geriatr, Unit Geriatr, Florence, Italy.

[Degli Esposti, Luca; Buda, Stefano] Clicon Hlth Econ & Outcome Res, Ravenna, Italy.

[Michelozzi, Paola] Lazio Reg, Dept Epidemiol, Rome, Italy.

[Bernabei, Roberto] Fdn Policlin Agostino Gemelli IRCCS, Rome, Italy.

[Marchionni, Niccolo] Azienda Osped Univ Careggi, Cardiothorac & Vasc Dept, Florence, Italy.

[Balzi, Daniela] Azienda USL Toscana Ctr, Dept Epidemiol, Florence, Italy.

C3 University of Florence; University of Florence; Azienda Ospedaliero

Universitaria Careggi; University of Florence; Azienda Ospedaliero

Universitaria Careggi

RP Di Bari, M (corresponding author), Univ Florence, Dept Expt & Clin Med, Res Unit Med Aging, Viale G Pieraccini 6, I-50139 Florence, Italy.

EM mauro.dibari@unifi.it

RI carreras, giulia/HPF-0028-2023; DI BARI, MAURO/J-1524-2012; Marchionni,

Niccolò/ABE-3977-2020

OI DI BARI, MAURO/0000-0002-5514-0524; Marchionni,

Niccolò/0000-0002-8101-4695; Carreras, Giulia/0000-0002-1427-637X

FU Centre for Disease Control of the Italian Ministry of Health

FX The "Silver Code National Project" was funded by the Centre for Disease

Control of the Italian Ministry of Health. The funder had no role in

study design, data collection and analysis, decision to publish, or

preparation of the manuscript. All the authors were independent from the

funder, had access to all of the data, and can take responsibility for

the integrity of the data and the accuracy of the data analysis.

CR Albaba M, 2012, MAYO CLIN PROC, V87, P652, DOI 10.1016/j.mayocp.2012.01.020

Aminzadeh F, 2002, ANN EMERG MED, V39, P238, DOI 10.1067/mem.2002.121523

[Anonymous], CLIN CLASS SOFTW CCS

Arnold BF, 2016, JAMA-J AM MED ASSOC, V316, P2597, DOI 10.1001/jama.2016.17700

Asomaning N, 2014, J EMERG NURS, V40, P357, DOI 10.1016/j.jen.2013.08.014

Asplund K, 2000, J AM GERIATR SOC, V48, P1381, DOI 10.1111/j.1532-5415.2000.tb02626.x

Balzi Daniela, 2019, BMJ Open, V9, pe033374, DOI 10.1136/bmjopen-2019-033374

Centro Nazionale per la Prevenzione e il Controllo delle Malattie, MOD INN PRES CAR PAZ MOD INN PRES CAR PAZ

Chiarantini D, 2010, J CARD FAIL, V16, P390, DOI 10.1016/j.cardfail.2010.01.004

Clegg A, 2016, AGE AGEING, V45, P353, DOI 10.1093/ageing/afw039

Corey KM, 2018, PLOS MED, V15, DOI 10.1371/journal.pmed.1002701

Corrao G, 2017, BMJ OPEN, V7, DOI 10.1136/bmjopen-2017-019503

Dagan N, 2017, BMJ-BRIT MED J, V356, DOI 10.1136/bmj.i6755

Davoudi A, 2017, IEEE INT C BIOINF BI, P568, DOI [10.1109/BIBE.2017.00103, 10.1109/BIBE.2017.00014]

Di Bari M, 2014, HEART, V100, P1537, DOI 10.1136/heartjnl-2013-305445

Di Bari M, 2012, J GERONTOL A-BIOL, V67, P544, DOI 10.1093/gerona/glr209

Di Bari M, 2010, J GERONTOL A-BIOL, V65, P159, DOI 10.1093/gerona/glp043

Ekerstad N, 2017, CLIN INTERV AGING, V12, P1, DOI 10.2147/CIA.S124003

Ellis G, 2017, COCHRANE DB SYST REV, DOI 10.1002/14651858.CD006211.pub3

Fracchia S, 2015, J GERONTOL A-BIOL, V70, P223, DOI 10.1093/gerona/glu199

HARRIS RD, 1991, AUST NZ J MED, V21, P230, DOI 10.1111/j.1445-5994.1991.tb00448.x

Hastings SN, 2007, J GEN INTERN MED, V22, P1527, DOI 10.1007/s11606-007-0343-9

Lo Coco D, 2016, VASC HEALTH RISK MAN, V12, P105, DOI 10.2147/VHRM.S75306

Mahajan SM, 2018, EUR J CARDIOVASC NUR, V17, P675, DOI 10.1177/1474515118799059

Quaderni del Ministero della Salute, 2010, CRIT APPR CLIN TECN CRIT APPR CLIN TECN

Reeves GR, 2016, AM J CARDIOL, V117, P1953, DOI 10.1016/j.amjcard.2016.03.046

RUBENSTEIN LZ, 1988, J CLIN EPIDEMIOL, V41, P441, DOI 10.1016/0895-4356(88)90045-5

Saltvedt I, 2002, J AM GERIATR SOC, V50, P792, DOI 10.1046/j.1532-5415.2002.50202.x

Simpson AN, 2018, J COMP EFFECT RES, V7, P293, DOI 10.2217/cer-2017-0058

Stensrud MJ, 2020, JAMA-J AM MED ASSOC, V323, P1401, DOI 10.1001/jama.2020.1267

Tanderup A, 2018, BMJ OPEN, V8, DOI 10.1136/bmjopen-2018-023803

Taylor RA, 2016, ACAD EMERG MED, V23, P269, DOI 10.1111/acem.12876

van Seben R, 2020, J GERONTOL A-BIOL, V75, P1403, DOI 10.1093/gerona/glaa039

Wallace E, 2014, MED CARE, V52, P751, DOI 10.1097/MLR.0000000000000171

Yourman LC, 2012, JAMA-J AM MED ASSOC, V307, P182, DOI 10.1001/jama.2011.1966

NR 35

TC 2

Z9 2

U1 0

U2 2

PU OXFORD UNIV PRESS INC

PI CARY

PA JOURNALS DEPT, 2001 EVANS RD, CARY, NC 27513 USA

SN 1079-5006

EI 1758-535X

J9 J GERONTOL A-BIOL

JI J. Gerontol. Ser. A-Biol. Sci. Med. Sci.

PD JUL

PY 2021

VL 76

IS 7

BP 1333

EP 1339

DI 10.1093/gerona/glaa147

PG 7

WC Geriatrics & Gerontology; Gerontology

WE Science Citation Index Expanded (SCI-EXPANDED); Social Science Citation Index (SSCI)

SC Geriatrics & Gerontology

GA TI4XB

UT WOS:000672804600026

PM 32542343

DA 2024-08-08

ER

PT J

AU NORCINI, JJ

LIPNER, RS

STEEL, K

AF NORCINI, JJ

LIPNER, RS

STEEL, K

TI PERFORMANCE OF CANDIDATES ON THE GERIATRIC-MEDICINE ITEMS OF THE

AMERICAN BOARD OF INTERNAL MEDICINE CERTIFYING EXAMINATION

SO JOURNAL OF THE AMERICAN GERIATRICS SOCIETY

LA English

DT Article

C1 BOSTON UNIV HOSP,MED CTR,EVANS MEM DEPT CLIN RES,BOSTON,MA 02218.

BOSTON UNIV HOSP,MED CTR,DEPT MED,BOSTON,MA 02218.

C3 Boston University; Boston University

RP NORCINI, JJ (corresponding author), AMER BOARD INTERNAL MED,UNIV CITY SCI CTR,3624 MARKET ST,PHILADELPHIA,PA 19104, USA.

OI Norcini, John/0000-0002-8464-4115

CR *I MED, 1978, AG MED ED

1981, ANN INTERN MED, V95, P372

NR 2

TC 2

Z9 2

U1 0

U2 0

PU WILLIAMS & WILKINS

PI BALTIMORE

PA 351 WEST CAMDEN ST, BALTIMORE, MD 21201-2436

SN 0002-8614

J9 J AM GERIATR SOC

JI J. Am. Geriatr. Soc.

PY 1985

VL 33

IS 11

BP 779

EP 780

DI 10.1111/j.1532-5415.1985.tb04190.x

PG 2

WC Geriatrics & Gerontology; Gerontology

WE Social Science Citation Index (SSCI); Science Citation Index Expanded (SCI-EXPANDED)

SC Geriatrics & Gerontology

GA AVD49

UT WOS:A1985AVD4900007

PM 4056274

DA 2024-08-08

ER

PT J

AU Lawlor, EF

Lyttle, CS

Moldwin, E

AF Lawlor, EF

Lyttle, CS

Moldwin, E

TI The state of geriatrics training programs: Findings from the National

Study of Internal Medicine Manpower (NaSIMM)

SO JOURNAL OF THE AMERICAN GERIATRICS SOCIETY

LA English

DT Article

ID PRIMARY-CARE; DISCIPLINE

RP Lawlor, EF (corresponding author), UNIV CHICAGO,CTR HLTH ADM STUDIES,969 E 60TH ST,CHICAGO,IL 60637, USA.

CR Benson J, 1994, AM J MED S4A, V97, p4A

BURTON JR, 1993, J AM GERIATR SOC, V41, P459, DOI 10.1111/j.1532-5415.1993.tb06958.x

ETTINGER WH, 1994, J AM GERIATR SOC, V42, P453, DOI 10.1111/j.1532-5415.1994.tb07498.x

GUPTA KL, 1993, J AM GERIATR SOC, V41, P579, DOI 10.1111/j.1532-5415.1993.tb01907.x

HAZZARD WR, 1992, J AM GERIATR SOC, V40, P1175, DOI 10.1111/j.1532-5415.1992.tb01810.x

*I MED COMM LEAD A, NEW ENGL J MED, V316, P1425

KERIN PB, 1989, GERONTOLOGIST, V29, P606, DOI 10.1093/geront/29.5.606

KRAMER AM, 1992, J AM GERIATR SOC, V40, P1055, DOI 10.1111/j.1532-5415.1992.tb04486.x

LAWLOR EF, 1994, PUBLIC POLICY AGING, V6, P9

LUCHI RJ, 1993, J AM GERIATR SOC, V41, P581, DOI 10.1111/j.1532-5415.1993.tb01912.x

MORELY JE, 1993, J AM GERIATR SOC, V41, P1150

MORRISON L, 1994, J AM GERIATR SOC, V42, P451, DOI 10.1111/j.1532-5415.1994.tb07497.x

REUBEN DB, 1994, J AM GERIATR SOC, V42, P363, DOI 10.1111/j.1532-5415.1994.tb07482.x

REUBEN DB, 1991, J AM GERIATR SOC, V39, P799, DOI 10.1111/j.1532-5415.1991.tb02703.x

TANGELOS E, 1994, J AM GERIATR SOC, V42, P456

NR 15

TC 7

Z9 7

U1 0

U2 1

PU WILLIAMS & WILKINS

PI BALTIMORE

PA 351 WEST CAMDEN ST, BALTIMORE, MD 21201-2436

SN 0002-8614

J9 J AM GERIATR SOC

JI J. Am. Geriatr. Soc.

PD JAN

PY 1997

VL 45

IS 1

BP 108

EP 111

DI 10.1111/j.1532-5415.1997.tb00988.x

PG 4

WC Geriatrics & Gerontology; Gerontology

WE Science Citation Index Expanded (SCI-EXPANDED); Social Science Citation Index (SSCI)

SC Geriatrics & Gerontology

GA WB733

UT WOS:A1997WB73300019

PM 8994498

DA 2024-08-08

ER

PT J

AU TURNBERG, LA

DAME, P

TURNERWARWICK, M

THOMAS, AJ

CARR, D

CLARK, F

DENHAM, MJ

GILMORE, IT

EVANS, JG

MCLELLAN, DL

NELIGAN, PH

MOORESMITH, B

SAUNDERS, KB

STOUT, RW

LONDON, DR

PENTECOST, BL

HOPKINS, AP

MORRIS, J

AF TURNBERG, LA

DAME, P

TURNERWARWICK, M

THOMAS, AJ

CARR, D

CLARK, F

DENHAM, MJ

GILMORE, IT

EVANS, JG

MCLELLAN, DL

NELIGAN, PH

MOORESMITH, B

SAUNDERS, KB

STOUT, RW

LONDON, DR

PENTECOST, BL

HOPKINS, AP

MORRIS, J

TI ENSURING EQUITY AND QUALITY OF CARE FOR ELDERLY PEOPLE - THE INTERFACE

BETWEEN GERIATRIC-MEDICINE AND GENERAL (INTERNAL) MEDICINE

SO JOURNAL OF THE ROYAL COLLEGE OF PHYSICIANS OF LONDON

LA English

DT Article

NR 0

TC 1

Z9 1

U1 0

U2 0

PU ROY COLL PHYS LONDON EDITORIAL OFFICE

PI LONDON

PA 11 ST ANDREWS PLACE REGENTS PARK, LONDON, ENGLAND NW1 4LE

SN 0035-8819

J9 J ROY COLL PHYS LOND

JI J. R. Coll. Physicians Lond.

PD MAY-JUN

PY 1994

VL 28

IS 3

BP 194

EP 196

PG 3

WC Medicine, General & Internal

WE Science Citation Index Expanded (SCI-EXPANDED)

SC General & Internal Medicine

GA NT103

UT WOS:A1994NT10300004

DA 2024-08-08

ER

PT J

AU Thomas, DC

Leipzig, RM

Smith, LG

Dunn, K

Sullivan, G

Callahan, E

AF Thomas, DC

Leipzig, RM

Smith, LG

Dunn, K

Sullivan, G

Callahan, E

TI Improving geriatrics training in internal medicine residency programs:

Best practices and sustainable solutions

SO ANNALS OF INTERNAL MEDICINE

LA English

DT Article

ID FAMILY-PRACTICE RESIDENCY; TEACHING NURSING-HOME; CURRICULUM

RECOMMENDATIONS; ACUTE-CARE; EXPERIENCE; KNOWLEDGE; ROTATION

AB National surveys indicate a need for additional training in geriatrics during internal medicine residencies. This paper describes 1) "best practices" for integrating geriatrics education into internal medicine residency programs, 2) barriers to implementation of these practices, and 3) possible ways to improve geriatrics training for internal medicine residents. These best practices were determined by a systematic review of the literature and through interviews with leaders of 26 residency and geriatrics programs concerned with geriatrics training for residents.

The most successful programs have clinical experiences with 3 key elements: model geriatric care in 1 or more settings (for example, in the hospital or in ambulatory practice), patient care across sites or transitions of care, and interdisciplinary teamwork. Barriers include attitudes, few faculty, need for relationships with nontraditional training sites, and lack of funding. Local solutions include engaging the internal medicine program director to accomplish a mutual goal-for example, by creating a model geriatrics training experience in which residents demonstrate their skill in a new Accreditation Council of Graduate Medical Education competency (such as systems-based practice). National solutions include reaching consensus on the competencies in geriatrics that should be achieved by board-eligible internists. This may mean increasing the number of questions that test geriatrics competency in the certifying and in-training examinations, increasing numbers of faculty members able to teach and model geriatric care, developing "effective medical resident teaching" courses for nonphysician faculty, and lobbying for improved systems of care.

C1 Mt Sinai Sch Med, New York, NY 10029 USA.

NYU, Sch Med, New York, NY USA.

Univ Connecticut, Hlth Sci Ctr, Farmington, CT USA.

C3 Icahn School of Medicine at Mount Sinai; New York University; University

of Connecticut

RP Mt Sinai Sch Med, 1 Gustave Levy Pl,Box 1087, New York, NY 10029 USA.

EM david.thomas@msnyuhealth.org

OI Dunn, Kathel/0000-0002-6967-130X

CR *ACCR COUNC GRAD M, 2003, PROGR REQ RES ED INT

Barbour G L, 1987, Gerontol Geriatr Educ, V7, P29

BARRY PP, 1991, AM J MED, V91, P447, DOI 10.1016/0002-9343(91)90177-Y

Blumenthal D, 2001, JAMA-J AM MED ASSOC, V286, P1027, DOI 10.1001/jama.286.9.1027

CHODOSH J, 1997, GERONTOL GERIATR ED, V18, P3

CLARFIELD AM, 1982, J AM GERIATR SOC, V30, P457, DOI 10.1111/j.1532-5415.1982.tb03384.x

COLENDA CC, 1994, AM J MED, V97, P15

COUNSELL SR, 1994, J AM GERIATR SOC, V42, P1200, DOI 10.1111/j.1532-5415.1994.tb06989.x

COUNSELL SR, 1994, J AM GERIATR SOC, V42, P1193, DOI 10.1111/j.1532-5415.1994.tb06988.x

Counsell SR, 1999, J AM GERIATR SOC, V47, P1145, DOI 10.1111/j.1532-5415.1999.tb05242.x

Degelau J., 1997, Journal of the American Geriatrics Society, V45, pS61

EUBANKS P, 1991, HOSPITALS, V65, P74

EVE SB, 1986, EDUC GERONTOL, V12, P59

*FCIM INT MED CURR, RES GUID CURR DEV

Friend T., 1996, Journal of the American Geriatrics Society, V44, pS65

GARRELL M, 1983, J MED EDUC, V58, P482

JAHNIGEN D, 1994, AM J MED, V97, P43

KATZ PR, 1992, J GEN INTERN MED, V7, P52, DOI 10.1007/BF02599103

KELLY JT, 1977, J FAM PRACTICE, V4, P1103

Koenig H G, 1986, Gerontol Geriatr Educ, V7, P15

Leipzig R.M., 2001, Gerontology and Geriatrics Education, V21, P63

Lindberg MC, 1996, J GEN INTERN MED, V11, P397, DOI 10.1007/BF02600185

Long C C, 1982, Gerontol Geriatr Educ, V2, P219

McNabney M., 1996, Journal of the American Geriatrics Society, V44, pS74

MOON JB, 1990, J FAM PRACTICE, V30, P594

Murden RA, 1996, J GEN INTERN MED, V11, P776, DOI 10.1007/BF02599002

PALMER RM, 1994, J AM GERIATR SOC, V42, P545, DOI 10.1111/j.1532-5415.1994.tb04978.x

REUBEN DB, 1990, ACAD MED, V65, P382, DOI 10.1097/00001888-199006000-00006

Reuben DB, 1997, J GEN INTERN MED, V12, P450, DOI 10.1046/j.1525-1497.1997.00078.x

Reuben DB, 1997, AM J MED, V103, P260

ROBBINS JA, 1983, J MED EDUC, V58, P811

RODRIGUEZ JD, 1998, GERONTOL GERIATR ED, V19, P67

Rubin CD, 2003, ANN INTERN MED, V139, P615, DOI 10.7326/0003-4819-139-7-200310070-00035

Sullivan GM, 1998, J AM GERIATR SOC, V46, P1166, DOI 10.1111/j.1532-5415.1998.tb06660.x

Swamy L, 1997, J AM GERIATR SOC, V45, pP77

WALKER L, 1998, GERONTOL GERIATR ED, V19, P79

WARSHAW G, 2003, IN PRESS J GEN INTER

WEILER PG, 1987, EDUC GERONTOL, V13, P1, DOI 10.1080/0380127870130101

WIELAND D, 1986, JAMA-J AM MED ASSOC, V255, P2622, DOI 10.1001/jama.255.19.2622

1982, JAMA, V247, P2787

NR 40

TC 57

Z9 61

U1 0

U2 4

PU AMER COLL PHYSICIANS

PI PHILADELPHIA

PA INDEPENDENCE MALL WEST 6TH AND RACE ST, PHILADELPHIA, PA 19106-1572 USA

SN 0003-4819

EI 1539-3704

J9 ANN INTERN MED

JI Ann. Intern. Med.

PD OCT 7

PY 2003

VL 139

IS 7

BP 628

EP 634

DI 10.7326/0003-4819-139-7-200310070-00037

PG 7

WC Medicine, General & Internal

WE Science Citation Index Expanded (SCI-EXPANDED)

SC General & Internal Medicine

GA 729AP

UT WOS:000185748800029

PM 14530247

DA 2024-08-08

ER

PT J

AU Hirth, VA

Eleazer, GP

Dever-Bumba, M

AF Hirth, Victor A.

Eleazer, G. Paul

Dever-Bumba, Maureen

TI A step toward solving the geriatrician shortage

SO AMERICAN JOURNAL OF MEDICINE

LA English

DT Article

ID NATIONAL-SURVEY; INTERNAL-MEDICINE; UNITED-STATES; CARE; ASSOCIATION;

FOUNDATION; PHYSICIANS; WORKFORCE; DIRECTORS; EDUCATION

C1 [Hirth, Victor A.; Eleazer, G. Paul; Dever-Bumba, Maureen] Univ S Carolina, Sch Med, Div Geriatr, Columbia, SC 29203 USA.

C3 University of South Carolina System; University of South Carolina

Columbia

RP Hirth, VA (corresponding author), Univ S Carolina, Sch Med, Div Geriatr, 3010 Farrow Rd,Suite 300A, Columbia, SC 29203 USA.

EM victor.hirth@palmettohealth.org

CR *AM GER SOC, 2005, GER MED CLIN IMP AG

[Anonymous], 2004, US INT PROJ AG SEX R

[Anonymous], 2002, MED NEV NEV LAND 10

*ASS AM MED COLL, 2007, MED ED COSTS STUD DE

BEDSINE R, 2005, J AM GERIATR SOC S, V53, pS245

BOND E, 1987, J AM GERIATR SOC, V35, P773

BRAGG E, 2005, TRAINING PRACTICE UP, V3, P1

Bragg EJ, 2006, FAM MED, V38, P258

Brotherton SE, 2006, JAMA-J AM MED ASSOC, V296, P1154, DOI 10.1001/jama.296.9.1154

BUTLER R, 2002, PREPARING AGING NATI

Diachun LL, 2006, J AM GERIATR SOC, V54, P512, DOI 10.1111/j.1532-5415.2005.00610.x

Elon Rebecca D, 2003, J Am Med Dir Assoc, V4, P117, DOI 10.1016/S1525-8610(04)70289-3

GIBBONS CD, 2006, OPPORTUNITIES CHALLE

*I MED, 1978, AGING MED ED

KANE R, 1980, NEW ENGL J MED, V302, P1327, DOI 10.1056/NEJM198006123022402

Kane RL, 2002, J GERONTOL A-BIOL, V57, pM803, DOI 10.1093/gerona/57.12.M803

LaMascus AM, 2005, J AM GERIATR SOC, V53, P343, DOI 10.1111/j.1532-5415.2005.53137.x

Landefeld CS, 2003, ANN INTERN MED, V139, P609, DOI 10.7326/0003-4819-139-7-200310070-00034

Leigh JP, 2002, ARCH INTERN MED, V162, P1577, DOI 10.1001/archinte.162.14.1577

Medina-Walpole A, 2002, J AM GERIATR SOC, V50, P949, DOI 10.1046/j.1532-5415.2002.50225.x

Newton DA, 2003, JAMA-J AM MED ASSOC, V290, P1179, DOI 10.1001/jama.290.9.1179

O'Neil G., 2003, PUBLIC POLICY AGING, V13, P17, DOI [DOI 10.1093/PPAR/13.2.17, 10.1093/ppar/13.2.17]

REUBEN DB, 1993, J AM GERIATR SOC, V41, P560, DOI 10.1111/j.1532-5415.1993.tb01896.x

Reuben DB, 2004, J AM GERIATR SOC, V52, P1384, DOI 10.1111/j.1532-5415.2004.52373.x

REUBEN DB, 1993, J AM GERIATR SOC, V41, P444, DOI 10.1111/j.1532-5415.1993.tb06955.x

SHAH U, 2002, GERONTOL GERIATR ED, V26, P57

Warshaw G.A., 2002, Geriatric medicine training and practice in the United States at the beginning of the 21st century

Warshaw GA, 2003, J AM GERIATR SOC, V51, P1023, DOI 10.1046/j.1365-2389.2003.51319.x

Warshaw GA, 2003, J AM GERIATR SOC, V51, pS338, DOI 10.1046/j.1365-2389.2003.51345.x

Warshaw GA, 2006, J AM GERIATR SOC, V54, P1603, DOI 10.1111/j.1532-5415.2006.00895.x

Weeks WB, 2004, J AM GERIATR SOC, V52, P1940, DOI 10.1111/j.1532-5415.2004.52523.x

Wesley SC, 2005, GERONTOL GERIATR EDU, V25, P13, DOI 10.1300/J021v25n03_02

NR 32

TC 17

Z9 17

U1 0

U2 3

PU ELSEVIER SCIENCE INC

PI NEW YORK

PA 360 PARK AVE SOUTH, NEW YORK, NY 10010-1710 USA

SN 0002-9343

J9 AM J MED

JI Am. J. Med.

PD MAR

PY 2008

VL 121

IS 3

BP 247

EP 251

DI 10.1016/j.amjmed.2007.10.030

PG 5

WC Medicine, General & Internal

WE Science Citation Index Expanded (SCI-EXPANDED)

SC General & Internal Medicine

GA 271YJ

UT WOS:000253824300016

PM 18328311

DA 2024-08-08

ER

PT J

AU Joiner, KA

Haponik, E

High, KP

AF Joiner, KA

Haponik, E

High, KP

TI Integrating geriatrics and subspecialty internal medicine: Results of a

survey on patient care practices, training, attitudes, and research

SO AMERICAN JOURNAL OF MEDICINE

LA English

DT Article

ID INFECTIOUS-DISEASES

C1 Yale Univ, Sch Med, Dept Internal Med, Div Infect Dis, New Haven, CT 06520 USA.

Johns Hopkins Univ, Dept Med, Div Pulm Med, Baltimore, MD 21218 USA.

Wake Forest Univ, Sch Med, Dept Med, Div Infect Dis, Winston Salem, NC 27109 USA.

C3 Yale University; Johns Hopkins University; Wake Forest University

RP Joiner, KA (corresponding author), Yale Univ, Sch Med, Dept Internal Med, Div Infect Dis, New Haven, CT 06520 USA.

RI Joiner, Keith/AAH-1059-2021

OI Joiner, Keith/0000-0003-3062-823X

CR Angus DC, 2000, JAMA-J AM MED ASSOC, V284, P2762, DOI 10.1001/jama.284.21.2762

Applegate WB, 1997, J AM GERIATR SOC, V45, P641, DOI 10.1111/j.1532-5415.1997.tb03103.x

Ely EW, 2001, CRIT CARE MED, V29, P1481

Hazzard WR, 1997, J AM GERIATR SOC, V45, P638, DOI 10.1111/j.1532-5415.1997.tb03102.x

High KP, 1999, CLIN INFECT DIS, V28, P708, DOI 10.1086/515217

High KP, 1999, CLIN INFECT DIS, V28, P753, DOI 10.1086/515211

Inouye SK, 1999, NEW ENGL J MED, V340, P669, DOI 10.1056/NEJM199903043400901

Kennedy B J, 1997, J Cancer Educ, V12, P139

NR 8

TC 3

Z9 3

U1 0

U2 1

PU EXCERPTA MEDICA INC

PI NEW YORK

PA 650 AVENUE OF THE AMERICAS, NEW YORK, NY 10011 USA

SN 0002-9343

J9 AM J MED

JI Am. J. Med.

PD FEB 15

PY 2002

VL 112

IS 3

BP 249

EP 254

AR PII S0002-9343(01)01125-1

DI 10.1016/S0002-9343(01)01125-1

PG 6

WC Medicine, General & Internal

WE Science Citation Index Expanded (SCI-EXPANDED)

SC General & Internal Medicine

GA 534TD

UT WOS:000174602100022

PM 11893362

DA 2024-08-08

ER

PT J

AU Hazzard, WR

Woolard, N

Regenstreif, DI

AF Hazzard, WR

Woolard, N

Regenstreif, DI

TI Internal medicine: At the nexus of the health care system in responding

to the demographic imperative of an aging population

SO AMERICAN JOURNAL OF MEDICINE

LA English

DT Article

ID GERIATRICS

CR FRIES JF, 1980, NEW ENGL J MED, V303, P130, DOI 10.1056/NEJM198007173030304

Hazzard WR, 1997, J AM GERIATR SOC, V45, P638, DOI 10.1111/j.1532-5415.1997.tb03102.x

HAZZARD WR, 1994, AM J MED, V97, pR1

HAZZARD WR, IN PRESS ANN INTERN

MANTON KG, 1995, NEW ENGL J MED, V333, P1232, DOI 10.1056/NEJM199511023331824

ROWE JW, 1987, SCIENCE, V237, P143, DOI 10.1126/science.3299702

Rowe JW, 1997, GERONTOLOGIST, V37, P433, DOI 10.1093/geront/37.4.433

Solomon DH, 2000, J AM GERIATR SOC, V48, P702, DOI 10.1111/j.1532-5415.2000.tb04734.x

NR 8

TC 13

Z9 15

U1 1

U2 2

PU ELSEVIER SCIENCE INC

PI NEW YORK

PA STE 800, 230 PARK AVE, NEW YORK, NY 10169 USA

SN 0002-9343

EI 1555-7162

J9 AM J MED

JI Am. J. Med.

PD APR 15

PY 2001

VL 110

IS 6

BP 507

EP 513

DI 10.1016/S0002-9343(01)00685-4

PG 7

WC Medicine, General & Internal

WE Science Citation Index Expanded (SCI-EXPANDED)

SC General & Internal Medicine

GA 425BD

UT WOS:000168267700020

PM 11331067

DA 2024-08-08

ER

PT J

AU Horn, L

Tzanetos, K

Thorpe, K

Straus, SE

AF Horn, Leora

Tzanetos, Katina

Thorpe, Kevin

Straus, Sharon E.

TI Factors associated with the subspecialty choices of internal medicine

residents in Canada

SO BMC MEDICAL EDUCATION

LA English

DT Article

AB Background: Currently, there are more residents enrolled in cardiology training programs in Canada than in immunology, pharmacology, rheumatology, infectious diseases, geriatrics and endocrinology combined. There is no published data regarding the proportion of Canadian internal medicine residents applying to the various subspecialties, or the factors that residents consider important when deciding which subspecialty to pursue. To address the concern about physician imbalances in internal medicine subspecialties, we need to examine the factors that motivate residents when making career decisions.

Methods: In this two-phase study, Canadian internal medicine residents participating in the post graduate year 4 (PGY4) subspecialty match were invited to participate in a web-based survey and focus group discussions. The focus group discussions were based on issues identified from the survey results. Analysis of focus group transcripts grew on grounded theory.

Results: 110 PGY3 residents participating in the PGY4 subspecialty match from 10 participating Canadian universities participated in the web-based survey (54% response rate). 22 residents from 3 different training programs participated in 4 focus groups held across Canada. Our study found that residents are choosing careers that provide intellectual stimulation, are consistent with their personality, and that provide a challenge in diagnosis. From our focus group discussions it appears that lifestyle, role models, mentorship and the experience of the resident with the specialty appear to be equally important in career decisions. Males are more likely to choose procedure based specialties and are more concerned with the reputation of the specialty as well as the anticipated salary. In contrast, residents choosing non-procedure based specialties are more concerned with issues related to lifestyle, including work-related stress, work hours and time for leisure as well as the patient populations they are treating.

Conclusion: This study suggests that internal medicine trainees, and particularly males, are increasingly choosing procedure-based specialties while non-procedure based specialties, and in particular general internal medicine, are losing appeal. We need to implement strategies to ensure positive rotation experiences, exposure to role models, improved lifestyle and job satisfaction as well as payment schedules that are equitable between disciplines in order to attract residents to less popular career choices.

C1 [Straus, Sharon E.] Univ Calgary, Dept Med, Calgary, AB T2N 2T9, Canada.

[Horn, Leora] Vanderbilt Univ, Dept Med Oncol, Nashville, TN 37232 USA.

[Tzanetos, Katina] Univ Toronto, Toronto Gen Hosp, Dept Med, Toronto, ON M5G 2C4, Canada.

[Thorpe, Kevin] Li Ka Shing Knowledge Inst, Keenan Res Ctr, Toronto, ON M5B 1W8, Canada.

[Thorpe, Kevin] Univ Toronto, Fac Med, Dept Publ Hlth Sci, Toronto, ON M5T 3M7, Canada.

C3 University of Calgary; Vanderbilt University; University of Toronto;

University Health Network Toronto; Toronto General Hospital; University

of Toronto; Li Ka Shing Knowledge Institute; University of Toronto

RP Straus, SE (corresponding author), Univ Calgary, Dept Med, Room 1103 S Tower,1403 29th St NW, Calgary, AB T2N 2T9, Canada.

EM l.horn@utoronto.ca; katina.tzanetos@uhn.on.ca; kevin.thorpe@utoronto.ca;

sharon.straus@utoronto.ca

FU Royal College of Physician and Surgeons of Canada; Tier 2 Canada

Research Chair; Alberta Heritage Foundation for Medical Research

FX This research was funded by a grant from the Royal College of Physician

and Surgeons of Canada.; Sharon Straus is supported by a Tier 2 Canada

Research Chair and by a Health Scholar Award from the Alberta Heritage

Foundation for Medical Research

CR ANDERSEN RM, 1992, ANN INTERN MED, V117, P243, DOI 10.7326/0003-4819-117-3-243

BABBOTT D, 1991, ANN INTERN MED, V114, P16, DOI 10.7326/0003-4819-114-1-16

Baker MZ, 1998, MED EDUC, V32, P527

Basco WT, 2001, ACAD MED, V76, P380, DOI 10.1097/00001888-200104000-00017

Benya RV, 2000, AM J GASTROENTEROL, V95, P777

Buddeberg-Fischer B, 2006, BMC HEALTH SERV RES, V6, DOI 10.1186/1472-6963-6-137

Canadian Society of Internal Medicine, 2005, CAR FULL DEF PLAN GE

DeWitt DE, 1998, J GEN INTERN MED, V13, P257, DOI 10.1046/j.1525-1497.1998.00076.x

Dillman D. A., 2000, Mail and internet surveys: The tailored design method

Dodson Thomas F, 2005, Curr Surg, V62, P128, DOI 10.1016/j.cursur.2004.07.009

Doherty MJ, 2002, NEUROLOGY, V58, P495, DOI 10.1212/WNL.58.3.495

Dorsey ER, 2003, JAMA-J AM MED ASSOC, V290, P1173, DOI 10.1001/jama.290.9.1173

Fincher RE, 2004, NEW ENGL J MED, V351, P630, DOI 10.1056/NEJMp048054

Ghali WA, 2006, J GEN INTERN MED, V21, P197, DOI 10.1111/j.1525-1497.2005.00289.x

GONG H, 1978, J MED EDUC, V53, P921

Hanly JG, 2001, J RHEUMATOL, V28, P1944

Harvey A, 2005, CAN MED ASSOC J, V172, P737, DOI 10.1503/cmaj.1040146

Jolly P., 2004, AAMC Report

KASSEBAUM DG, 1993, ACAD MED, V68, P866, DOI 10.1097/00001888-199311000-00018

KLIPPERAURBACH Y, 1995, MED HYPOTHESES, V45, P486, DOI 10.1016/0306-9877(95)90228-7

Kwong JC, 2002, CAN MED ASSOC J, V166, P1023

LIEU TA, 1989, ACAD MED, V64, P622, DOI 10.1097/00001888-198910000-00015

Lingard L, 2007, QUALITATIVE RES MED

LINZER M, 1994, J GEN INTERN MED, V9, pS14, DOI 10.1007/BF02598114

MARTINI CJM, 1994, JAMA-J AM MED ASSOC, V272, P661, DOI 10.1001/jama.272.9.661

Mayer KL, 2001, J SURG RES, V98, P71, DOI 10.1006/jsre.2001.6143

Newton DA, 2005, ACAD MED, V80, P809, DOI 10.1097/00001888-200509000-00005

NK D, 2000, HDB QUALITATIVE

RAMSDELL JW, 1983, J MED EDUC, V58, P547

Rosenthal MP, 1996, ACAD MED, V71, P675, DOI 10.1097/00001888-199606000-00024

Ross GS, 2005, ANN INTERN MED, V143, P693, DOI 10.7326/0003-4819-143-9-200511010-00021

Sambunjak D, 2006, JAMA-J AM MED ASSOC, V296, P1103, DOI 10.1001/jama.296.9.1103

Straus SE, 2006, J GEN INTERN MED, V21, P1222, DOI 10.1111/j.1525-1497.2006.00599.x

Teichman JMH, 2005, CAN J SURG, V48, P27

Thornton J, 2003, HEALTH ECON, V12, P67, DOI 10.1002/hec.682

Whittaker LD, 2006, AM J SURG, V191, P320, DOI 10.1016/j.amjsurg.2005.10.029

Williams GC, 1997, SOC SCI MED, V45, P1705, DOI 10.1016/S0277-9536(97)00103-2

Wright S, 1997, J GEN INTERN MED, V12, P53, DOI 10.1046/j.1525-1497.1997.12109.x

NR 38

TC 49

Z9 49

U1 0

U2 6

PU BIOMED CENTRAL LTD

PI LONDON

PA 236 GRAYS INN RD, FLOOR 6, LONDON WC1X 8HL, ENGLAND

SN 1472-6920

J9 BMC MED EDUC

JI BMC Med. Educ.

PY 2008

VL 8

AR 37

DI 10.1186/1472-6920-8-37

PG 8

WC Education & Educational Research; Education, Scientific Disciplines

WE Science Citation Index Expanded (SCI-EXPANDED); Social Science Citation Index (SSCI)

SC Education & Educational Research

GA V14LE

UT WOS:000207735100037

OA gold, Green Published

DA 2024-08-08

ER

PT J

AU Thomas, KG

West, CP

Popkave, C

Bellini, LM

Weinberger, SE

Kolars, JC

Kogan, JR

AF Thomas, Kris G.

West, Colin P.

Popkave, Carol

Bellini, Lisa M.

Weinberger, Steven E.

Kolars, Joseph C.

Kogan, Jennifer R.

TI Alternative Approaches to Ambulatory Training: Internal Medicine

Residents' and Program Directors' Perspectives

SO JOURNAL OF GENERAL INTERNAL MEDICINE

LA English

DT Article

DE medical education-graduate; ambulatory care; curriculum/program

evaluation; medical student and residency education

ID CARE; CONTINUITY; SATISFACTION; INPATIENT; EDUCATION; PHYSICIAN; TIME

AB Internal medicine ambulatory training redesign, including recommendations to increase ambulatory training, is a focus of national discussion. Residents' and program directors' perceptions about ambulatory training models are unknown.

To describe internal medicine residents' and program directors' perceptions regarding ambulatory training duration, alternative ambulatory training models, and factors important for ambulatory education.

National cohort study.

Internal medicine residents (N = 14,941) and program directors (N = 222) who completed the 2007 Internal Medicine In-Training Examination (IM-ITE) Residents Questionnaire or Program Directors Survey, representing 389 US residency programs.

A total of 58.4% of program directors and 43.7% of residents preferred one-third or more training time in outpatient settings. Resident preferences for one-third or more outpatient training increased with higher levels of training (48.3% PGY3), female sex (52.7%), primary care program enrollment (64.8%), and anticipated outpatient-focused career, such as geriatrics. Most program directors (77.3%) and residents (58.4%) preferred training models containing weekly clinic. Although residents and program directors reported problems with competing inpatient-outpatient responsibilities (74.9% and 88.1%, respectively) and felt that absence of conflict with inpatient responsibilities is important for good outpatient training (69.4% and 74.2%, respectively), only 41.6% of residents and 22.7% of program directors supported models eliminating ambulatory sessions during inpatient rotations.

Residents' and program directors' preferences for outpatient training differ from recommendations for increased ambulatory training. Discordance was observed between reported problems with conflicting inpatient-outpatient responsibilities and preferences for models maintaining longitudinal clinic during inpatient rotations. Further study regarding benefits and barriers of ambulatory redesign is needed.

C1 [Thomas, Kris G.; West, Colin P.; Kolars, Joseph C.] Mayo Clin, Coll Med, Rochester, MN USA.

[Popkave, Carol; Weinberger, Steven E.] Amer Coll Physicians, Philadelphia, PA USA.

[Bellini, Lisa M.; Kogan, Jennifer R.] Univ Penn Hlth Syst, Philadelphia, PA 19104 USA.

C3 Mayo Clinic; American College of Physicians; University of Pennsylvania

RP Kogan, JR (corresponding author), Univ Penn Hlth Syst, 3701 Market St,Suite 640, Philadelphia, PA 19104 USA.

EM jennifer.kogan@uphs.upenn.edu

OI Kolars, Joseph/0000-0002-2603-188X

CR *ACGME, ACGME NUMB ACCR PROG

*ACGME, ACGME PROGR REQ RES

*AM COLL PHYS, JOINT PRINC PAT CTR

[Anonymous], PROGRAM REQUIREMENTS

Arora V, 2005, AM J MED, V118, P680, DOI 10.1016/j.amjmed.2005.03.022

Bharel M, 2003, J GEN INTERN MED, V18, P288, DOI 10.1046/j.1525-1497.2003.20712.x

BLANKFIELD RP, 1990, J FAM PRACTICE, V31, P69

BLUMENTHAL D, 2001, JAMA-J AM MED ASSOC, V286, P1127

Bowen JL, 2005, J GEN INTERN MED, V20, P1181, DOI 10.1111/j.1525-1497.2005.0248.x

Charap MH, 2005, AM J MED, V118, P1042, DOI 10.1016/j.amjmed.2005.06.009

CROASDALE M, REDESIGNING RESIDENC

Darer JD, 2004, ACAD MED, V79, P541, DOI 10.1097/00001888-200406000-00009

Feddock CA, 2005, EVAL HEALTH PROF, V28, P390, DOI 10.1177/0163278705281070

Fitzgibbons JP, 2006, ANN INTERN MED, V144, P920, DOI 10.7326/0003-4819-144-12-200606200-00010

GARIBALDI RA, 1994, ANN INTERN MED, V121, P117, DOI 10.7326/0003-4819-121-2-199407150-00008

Garibaldi RA, 2002, ANN INTERN MED, V137, P505, DOI 10.7326/0003-4819-137-6-200209170-00011

Green LA, 2001, NEW ENGL J MED, V344, P2021, DOI 10.1056/NEJM200106283442611

HODGE RH, 1979, J MED EDUC, V54, P638

Holmboe ES, 2005, J GEN INTERN MED, V20, P1165, DOI 10.1111/j.1525-1497.2005.0249.x

Huddle TS, 2008, ACAD MED, V83, P910, DOI 10.1097/ACM.0b013e3181850a92

IRBY DM, 1995, ACAD MED, V70, P898, DOI 10.1097/00001888-199510000-00014

Keirns Carla C, 2008, Acad Med, V83, P498, DOI 10.1097/ACM.0b013e31816be3ab

LINN LS, 1985, MED CARE, V23, P1171, DOI 10.1097/00005650-198510000-00006

McBurney PG, 2004, PEDIATRICS, V114, P1023, DOI 10.1542/peds.2003-0280-L

Meyers FJ, 2007, ACAD MED, V82, P1211, DOI 10.1097/ACM.0b013e318159d010

Randall C S, 1997, Fam Med, V29, P730

Saint S, 2003, J GEN INTERN MED, V18, P725, DOI 10.1046/j.1525-1497.2003.20637.x

Salerno SM, 2007, TEACH LEARN MED, V19, P30, DOI 10.1207/s15328015tlm1901_6

Sisson SD, 2007, J GEN INTERN MED, V22, P1704, DOI 10.1007/s11606-007-0412-0

Smith CS, 1997, ACAD MED, V72, P32

Warm EJ, 2008, J GEN INTERN MED, V23, P921, DOI 10.1007/s11606-008-0588-y

Weinberger SE, 2006, ANN INTERN MED, V144, P927, DOI 10.7326/0003-4819-144-12-200606200-00124

Wiest FC, 2002, JAMA-J AM MED ASSOC, V288, P2609, DOI 10.1001/jama.288.20.2609

NR 33

TC 20

Z9 20

U1 0

U2 1

PU SPRINGER

PI NEW YORK

PA 233 SPRING ST, NEW YORK, NY 10013 USA

SN 0884-8734

EI 1525-1497

J9 J GEN INTERN MED

JI J. Gen. Intern. Med.

PD AUG

PY 2009

VL 24

IS 8

BP 904

EP 910

DI 10.1007/s11606-009-1015-8

PG 7

WC Health Care Sciences & Services; Medicine, General & Internal

WE Science Citation Index Expanded (SCI-EXPANDED)

SC Health Care Sciences & Services; General & Internal Medicine

GA 471PC

UT WOS:000268069500003

PM 19475458

OA Green Published

DA 2024-08-08

ER

PT J

AU Sahin, S

Timur, O

Alp, A

Oven, B

Duman, S

Akcicek, F

AF Sahin, S.

Timur, O.

Alp, A.

Oven, B.

Duman, S.

Akcicek, F.

TI Assessment of geriatric patients at the internal medicine intensive care

unit (IMICU)

SO EUROPEAN GERIATRIC MEDICINE

LA English

DT Article

DE Geriatrics; Intensive care unit; Elderly; Internal medicine

ID PATIENTS AGED 80; ACUTE-RENAL-FAILURE; QUALITY-OF-LIFE;

ELDERLY-PATIENTS; BLOOD-TRANSFUSION; CRITICALLY-ILL; MORTALITY; OLDER;

ICU; ADMISSION

AB Background: The aim of this study was to evaluate the general characteristics of geriatric patients hospitalized at the internal medicine intensive care unit (IMICU).

Methods: All patients aged above 65 years and admitted to the intensive care unit of the internal medicine department at Ege University Hospital October 2010 to February 2011 were included in the study. Files of the patients were accessed retrospectively and the following information was recorded: age, gender, main cause of intensive care admission, number of chronic diseases, laboratory results, drug use and other therapeutic procedures, length of stay, outcomes in terms of survival and mortality. Secondary outcomes were defined to compare patients admitted to the intensive care unit from the emergency ward with those transferred from the internal medicine wards.

Results: The total number of patients admitted to the internal medicine intensive care unit during the study period was 357, of whom 171(47.99%) were aged above 65 years, a mean age of 74 +/- 6.6 years without any statistically significant difference between gender (P = 0.859). Days of hospitalization at the intensive care were 15.7 +/- 4.8, in patient mortality was found to be 17.7%, The mean number of chronic diseases per patient was 2.2 +/- 1.3. and the most frequent chronic diseases were arterial hypertension 57%. The most frequent pathology of the patients was found to be renal dysfunction 38.6% (n = 66), followed by anaemia 38.0% (n = 65) and liver dysfunction 11.7% (n = 20). The ratio of haemodialysis (HD) was found as 11.7%. When the patients were assessed in terms of invasive interventions by grouping them into those admitted from the emergency unit and those transferring from the internal medicine departments, it was seen that foley, intravenous catheterization, nasogastric tube insertion and HD were significantly higher for the patients admitted from the emergency unit. Mortality rate was found to be significantly higher for same group (44% versus 17, P < 0.001).

Conclusion: The proportion of elderly in the intensive care unit is increasing. In the present study renal dysfunction is the most common organ failure. Infection seems to be one of the common problems in IMICU. Organ failure and invasive intervention needs are high in elderly patient admitted from the emergency ward to intensive care. (c) 2012 Elsevier Masson SAS and European Union Geriatric Medicine Society. All rights reserved.

C1 [Sahin, S.; Akcicek, F.] Ege Univ, Div Geriatr Med, Dept Internal Med, Bornova, Turkey.

[Timur, O.; Oven, B.; Duman, S.] Ege Univ, Dept Internal Med, Bornova, Turkey.

[Alp, A.] Adnan Menderes Univ, Div Nephrol, Dept Internal Med, Aydin, Turkey.

C3 Ege University; Ege University; Adnan Menderes University

RP Sahin, S (corresponding author), Ege Univ, Div Geriatr Med, Dept Internal Med, Bornova, Turkey.

EM drsevnaz@hotmail.com

RI Alp, Alper/AAK-2766-2020

OI Alp, Alper/0000-0002-2864-361X

CR Azoulay É, 2001, CRIT CARE MED, V29, P2132, DOI 10.1097/00003246-200111000-00014

Bo M, 2003, J AM GERIATR SOC, V51, P529, DOI 10.1046/j.1532-5415.2003.51163.x

Boumendil A, 2005, J AM GERIATR SOC, V53, P88, DOI 10.1111/j.1532-5415.2005.53016.x

Brunner-Ziegler S, 2007, WIEN KLIN WOCHENSCHR, V119, P14, DOI 10.1007/s00508-007-0771-x

Chant C, 2006, CRIT CARE, V10, P2

Chronopoulos A, 2010, INTENS CARE MED, V36, P1454, DOI 10.1007/s00134-010-1957-7

Corwin HL, 2004, CRIT CARE MED, V32, P39, DOI 10.1097/01.CCM.0000104112.34142.79

de Mendonça A, 2000, INTENS CARE MED, V26, P915, DOI 10.1007/s001340051281

de Rooij SE, 2005, CRIT CARE, V9, pR307, DOI 10.1186/cc3536

Escher M, 2004, BMJ-BRIT MED J, P329

Fuchs L, 2012, INTENS CARE MED, V38, P1654, DOI 10.1007/s00134-012-2629-6

Inouye SK, 2007, J AM GERIATR SOC, V55, P780, DOI 10.1111/j.1532-5415.2007.01156.x

Joannidis M, 2009, INTENS CARE MED, V35, P1692, DOI 10.1007/s00134-009-1530-4

KNAUS WA, 1981, CRIT CARE MED, V9, P591, DOI 10.1097/00003246-198108000-00008

Lerolle N, 2010, CRIT CARE MED, V38, P59, DOI 10.1097/CCM.0b013e3181b088ec

Mark PE, 2006, CRIT CARE MED, V34, pS176, DOI 10.1097/01.CCM.0000232624.14883.9A

MAYEROAKES SA, 1991, J AM GERIATR SOC, V39, P862, DOI 10.1111/j.1532-5415.1991.tb04452.x

Nalbant S, 2001, TURK J GERIATR, V4, P135

NICOLAS F, 1987, INTENS CARE MED, V13, P9, DOI 10.1007/BF00263549

Pascual J, 1998, J AM GERIATR SOC, V46, P721, DOI 10.1111/j.1532-5415.1998.tb03807.x

Pisani Margaret A, 2009, J Intensive Care Med, V24, P83, DOI 10.1177/0885066608329942

Roch A, 2011, CRIT CARE, V15, DOI 10.1186/cc9984

Somme D, 2003, INTENS CARE MED, V29, P2137, DOI 10.1007/s00134-003-1929-2

Tabah A, 2010, CRIT CARE, V14, DOI 10.1186/cc8231

Udekwu P, 2001, J AM COLL SURGEONS, V193, P245, DOI 10.1016/S1072-7515(01)00994-2

Ulger Z, 2006, YOGUN BAKIM DERGISI, V6, P94

Uysal N, 2010, TURKISH J MED SURGL, V1, P1

Walsh TS, 2006, INTENS CARE MED, V32, P100, DOI 10.1007/s00134-005-2855-2

Nguyen YL, 2011, ANN INTENSIVE CARE, V1, DOI 10.1186/2110-5820-1-29

NR 29

TC 1

Z9 2

U1 31

U2 34

PU SPRINGER

PI NEW YORK

PA 233 SPRING ST, NEW YORK, NY 10013 USA

SN 1878-7649

EI 1878-7657

J9 EUR GERIATR MED

JI Eur. Geriatr. Med.

PD FEB

PY 2013

VL 4

IS 1

BP 20

EP 24

DI 10.1016/j.eurger.2012.10.005

PG 5

WC Geriatrics & Gerontology

WE Science Citation Index Expanded (SCI-EXPANDED); Social Science Citation Index (SSCI)

SC Geriatrics & Gerontology

GA 287SA

UT WOS:000329561300005

DA 2024-08-08

ER

PT J

AU Nardi, R

Scanelli, G

Borioni, D

Grandi, M

Sacchetti, C

Parenti, M

Fiorino, S

Iori, I

Donato, C

Agostinelli, P

Cipollini, F

Pelliccia, G

Centurioni, R

Pontoriero, L

AF Nardi, Roberto

Scanelli, Giovanni

Borioni, Daniele

Grandi, Marco

Sacchetti, Carla

Parenti, Mario

Fiorino, Sirio

Iori, Ido

Di Donato, Carlo

Agostinelli, Paolo

Cipollini, Francesco

Pelliccia, Gennaro

Centurioni, Riccardo

Pontoriero, Laura

TI The assessment of complexity in internal medicine patients. The FADOI

Medicomplex study

SO EUROPEAN JOURNAL OF INTERNAL MEDICINE

LA English

DT Article

DE co-morbidity; internal medicine; complexity; elderly

ID ILLNESS RATING-SCALE; ADMINISTRATIVE DATA; COMORBIDITY; DISEASE; BURDEN

AB Background: Although the number of elderly people is progressively increasing in the world, old and very old patients have been under-represented and understudied in trials evaluating the efficacy of chronic illness management models. The usual hospital indicators and practice guidelines do not consider the effects of complexity - co-morbidity, social support, functional and cognitive status, patient adherence to therapy, risk of adverse drug reactions - in these subjects. The aim of this observational, multi-centric cohort study was to carefully assess factors contributing to the complexity of care for patients admitted to internal medicine wards. This was done by evaluating the severity of disease and degree of stability at admission, co-morbidity, age-related impairments, and the need for discharge planning plus post-discharge support.

Methods: A total of 3 86 patients from 11 internal medicine wards in Emilia-Romagna and Marche, Italy, enrolled in a given week were evaluated. At admission, the following variables were recorded: demographic characteristics, medical history, global clinical-social prognostic evaluation, co-morbidity, severity of illness, presence of shock or hemodynamic instability, coma, and frequencies and causes of unscheduled hospital re-admission.

Results: Cancer, congestive heart failure, pneumonia, stroke, and chronic obstructive pulmonary disease were the most frequent primary diagnoses. The complexity of our case study was characterized by several concomitant diseases. Over 50% of the patients were considered severe or more than severe, and over 20% extremely severe, with very high co-morbidity indices and illness severity scores. Some 55% of our patients were in need of partial or total care; 10% had some speech impairment, and 63% needed in-home health care after hospital discharge.

Conclusions: The increasing numbers of elderly patients admitted to internal medicine departments suggests the need for a chronic illness management model, integrating gerontological and geriatric care to improve outcomes. For effective care, future protocols need to take a multidimensional, interdisciplinary approach to these patients and to develop a coordinated, integrated plan for treatment and long-term follow-up. (c) 2007 European Federation of Internal Medicine. Published by Elsevier B.V. All rights reserved.

C1 Osp G Dossetti, UO Med Interna, I-40053 Bologna, Italy.

Osped Maggiore Bologna, Bologna, Italy.

Azienda Osped Univ Arcispedale Sant Anna, Ferrara, Italy.

Osped Sassuolo, Sassuolo, Italy.

Osped Rimini, Rimini, Italy.

Osped Budrio, Budrio, Italy.

Azienda Osped Arcipedale S Maria Nuova Reggio Emi, Reggio Emilia, Italy.

Osped Vignola, Vignola, Italy.

Osped Jesi, Jesi, Italy.

Osped Amandola Ascoli Piceno, Ascoli Piceno, Italy.

Osped Fermo, Fermo, Italy.

Osped Civitanova Marche, Civitanova Marche, Italy.

Osped Lamezia Terme, Lamezia Terme, Italy.

C3 AUSL di Bologna; University of Ferrara; Arcispedale Sant'Anna; Hospital

of Rimini

RP Nardi, R (corresponding author), Osp G Dossetti, UO Med Interna, Viale Martiri 10-b, I-40053 Bologna, Italy.

EM r.nardi@auslbosud.emr.it

CR Elixhauser A, 1998, MED CARE, V36, P8, DOI 10.1097/00005650-199801000-00004

FLUGELMAN MY, 1986, GERONTOLOGY, V32, P272, DOI 10.1159/000212801

Hazzard WR, 2000, ANN INTERN MED, V133, P293, DOI 10.7326/0003-4819-133-4-200008150-00014

John R, 2003, GERONTOLOGIST, V43, P649, DOI 10.1093/geront/43.5.649

Kügler C, 2003, INT ANGIOL, V22, P290

Landi F, 2004, J CLIN EPIDEMIOL, V57, P832, DOI 10.1016/j.jclinepi.2004.01.013

LINN BS, 1968, J AM GERIATR SOC, V16, P622, DOI 10.1111/j.1532-5415.1968.tb02103.x

MILLER MD, 1992, PSYCHIAT RES, V41, P237, DOI 10.1016/0165-1781(92)90005-N

Miller MD., 1991, MANUAL GUIDELINES SC

Monti M A, 2001, Ann Ital Med Int, V16, P38

Naylor MD, 2004, J AM GERIATR SOC, V52, P675, DOI 10.1111/j.1532-5415.2004.52202.x

PARMELEE PA, 1995, J AM GERIATR SOC, V43, P130, DOI 10.1111/j.1532-5415.1995.tb06377.x

Pettina G., 2005, G. Ital. Med. Interna, V4, P64

Powell H, 2001, J CLIN EPIDEMIOL, V54, P687, DOI 10.1016/S0895-4356(00)00364-4

Quan HD, 2002, MED CARE, V40, P675, DOI 10.1097/00005650-200208000-00007

NR 15

TC 38

Z9 38

U1 0

U2 4

PU ELSEVIER SCIENCE BV

PI AMSTERDAM

PA PO BOX 211, 1000 AE AMSTERDAM, NETHERLANDS

SN 0953-6205

J9 EUR J INTERN MED

JI Eur. J. Intern. Med.

PD JUL

PY 2007

VL 18

IS 4

BP 283

EP 287

DI 10.1016/j.ejim.2006.12.006

PG 5

WC Medicine, General & Internal

WE Science Citation Index Expanded (SCI-EXPANDED)

SC General & Internal Medicine

GA 194TR

UT WOS:000248367200004

PM 17574101

DA 2024-08-08

ER

PT J

AU Hazzard, WR

Woolard, N

Regenstreif, DI

AF Hazzard, WR

Woolard, N

Regenstreif, DI

TI Integrating geriatrics into the subspecialties of internal medicine: The

Hartford Foundation American Geriatrics Society Wake Forest University

Bowman Gray School of Medicine Initiative

SO JOURNAL OF THE AMERICAN GERIATRICS SOCIETY

LA English

DT Article

RP Hazzard, WR (corresponding author), WAKE FOREST UNIV,BOWMAN GRAY SCH MED,DEPT INTERNAL MED,MED CTR BLVD,WINSTON SALEM,NC 27157, USA.

CR Hazzard W R, 1993, N C Med J, V54, P161

NR 1

TC 22

Z9 22

U1 0

U2 0

PU WILLIAMS & WILKINS

PI BALTIMORE

PA 351 WEST CAMDEN ST, BALTIMORE, MD 21201-2436

SN 0002-8614

J9 J AM GERIATR SOC

JI J. Am. Geriatr. Soc.

PD MAY

PY 1997

VL 45

IS 5

BP 638

EP 640

DI 10.1111/j.1532-5415.1997.tb03102.x

PG 3

WC Geriatrics & Gerontology; Gerontology

WE Science Citation Index Expanded (SCI-EXPANDED); Social Science Citation Index (SSCI)

SC Geriatrics & Gerontology

GA WZ009

UT WOS:A1997WZ00900019

PM 9158591

OA Bronze

DA 2024-08-08

ER

PT J

AU Steinsdóttir, HR

Sigurosson, MI

Björnsson, ES

Jónsdóttir, F

AF Steinsdottir, Helga Rut

Sigurosson, Martin I.

Bjornsson, Einar Stefan

Jonsdottir, Freyja

TI The incidence and prevalence of proton pump inhibitor usage among

internal medicine patients after hospital admission: A retrospective

cohort study

SO EUROPEAN JOURNAL OF CLINICAL PHARMACOLOGY

LA English

DT Article

DE Proton pump inhibitors; Hospital admission; Polypharmacy; Inappropriate

prescribing; Drug usage

ID HEALTH-CARE; DISCHARGE; PPIS

AB BackgroundThe use of proton pump inhibitors (PPIs) has increased over the past decades. One potential gateway into new PPI use is following a hospital admission. The study aimed to examine the incidence of new PPI usage following admission to internal medicine services and the ratio of new persistent users.MethodsA retrospective descriptive study was conducted among all adults who had been admitted to internal medicine wards at the National University Hospital of Iceland from 2010-2020. Data was obtained from the Icelandic Internal Medicine Database. The proportion of patients who started treatment with PPI within 3 months of discharge (new users) and the proportion of patients who continued to use it after 3 months (persistent users) were examined.ResultsAmong 85.942 admissions during the study period, 7238 (15.6%) became new users, and of those 4942 (68%) were new persistent users. The incidence of new PPI use was highest for patients discharged from gastroenterology (32.2%), hematology (31.8%), and oncology (29.2%). Patients with new PPI use more commonly had a history of malignancy (19.5%) and liver disease (22.7%) and more commonly were admitted to the ICU during their hospitalization. The highest ratio of persistent usage was among patients discharged from geriatric medicine (84%).ConclusionOne in every six patients admitted to internal medicine wards filled out a prescription for PPI within 3 months from discharge, and a large proportion of them became persistent users. The high rate of new PPI users from oncology and hematology is noteworthy and requires further research.

C1 [Steinsdottir, Helga Rut; Jonsdottir, Freyja] Landspitali Natl Univ, Clin Pharm Serv, Hosp Iceland, Reykjavik, Iceland.

[Sigurosson, Martin I.; Bjornsson, Einar Stefan] Univ Iceland, Fac Med, Reykjavik, Iceland.

[Sigurosson, Martin I.] Landspitali Natl Univ, Div Anesthesia & Intens Care Med, Hosp Iceland, Reykjavik, Iceland.

[Bjornsson, Einar Stefan] Landspitali Natl Univ, Dept Internal Med, Div Gastroenterol, Hosp Iceland, Reykjavik, Iceland.

[Jonsdottir, Freyja] Univ Iceland, Pharmaceut Sci, Reykjavik, Iceland.

C3 University of Iceland; University of Iceland

RP Steinsdóttir, HR (corresponding author), Landspitali Natl Univ, Clin Pharm Serv, Hosp Iceland, Reykjavik, Iceland.

EM helgarst@landspitali.is

OI Steinsdottir, Helga Rut/0009-0004-2939-6640

FU Icelandic Centre for Research

FX No Statement Available

CR Balzano A, 2019, DIGEST LIVER DIS, V51, P43, DOI 10.1016/j.dld.2018.06.015

Batuwitage BT, 2007, POSTGRAD MED J, V83, P66, DOI 10.1136/pgmj.2006.051151

Cameron KA, 2010, J WOMENS HEALTH, V19, P1643, DOI 10.1089/jwh.2009.1701

Collet JP, 2021, EUR HEART J, V42, P1289, DOI 10.1093/eurheartj/ehaa575

DA Peura BR., 2011, P T, V36, P434

Daniels B, 2020, THER ADV GASTROENTER, V13, DOI 10.1177/1756284820913743

Elinardottir S, 2020, LYFJAGAGNAGRUNNUR LA

Excellence NIfHa C, 2014, GASTR OES REFL DIS D

Fukuba N, 2020, CUREUS J MED SCIENCE, V12, DOI 10.7759/cureus.7267

Gawron AJ, 2015, GASTROENT RES PRACT, V2015, DOI 10.1155/2015/689531

Hálfdánarson OÖ, 2018, THER ADV GASTROENTER, V11, DOI 10.1177/1756284818777943

Helgadottir H, 2019, INT J MOL SCI, V20, DOI 10.3390/ijms20215469

Jaynes M, 2018, THER ADV DRUG SAF, V10, DOI 10.1177/2042098618809927

Johnson DA, 2017, DRUGS, V77, P547, DOI 10.1007/s40265-017-0712-6

Katz PO, 2013, AM J GASTROENTEROL, V108, P308, DOI 10.1038/ajg.2012.444

Kristjansdottir TR, 2021, HASKOLI ISLANDS, P7

Laine L, 2016, AM J GASTROENTEROL, V111, P913, DOI 10.1038/ajg.2016.156

Lanas A, 2016, AM J GASTROENTEROL, V111, P1085, DOI 10.1038/ajg.2016.166

Landlaeknis E., LYFJATOLFRAEOI GANGV

Liu YJ, 2020, BMJ OPEN, V10, DOI 10.1136/bmjopen-2020-040473

Lodato F, 2016, EUR J INTERN MED, V30, P31, DOI 10.1016/j.ejim.2016.01.025

McCaleb RV, 2016, ANN PHARMACOTHER, V50, P541, DOI 10.1177/1060028016644469

Ng TP, 2014, J AM MED DIR ASSOC, V15, P635, DOI 10.1016/j.jamda.2014.03.008

NICE NIfHaCE, 2014, GASTR OES REFL DIS D

Numico G, 2017, CRIT REV ONCOL HEMAT, V111, P144, DOI 10.1016/j.critrevonc.2017.01.014

Pottegård A, 2016, THER ADV GASTROENTER, V9, P671, DOI 10.1177/1756283X16650156

Ramírez E, 2010, CURR CLIN PHARMACOL, V5, P288, DOI 10.2174/157488410793352067

Robinson M, 2003, DRUGS, V63, P2739, DOI 10.2165/00003495-200363240-00004

Roffman CE, 2016, J PHYSIOTHER, V62, P171, DOI 10.1016/j.jphys.2016.05.008

Sattayalertyanyong O, 2020, INT J CLIN PHARM-NET, V42, P174, DOI 10.1007/s11096-019-00955-8

Scarpignato C, 2016, BMC MED, V14, DOI 10.1186/s12916-016-0718-z

Simmonds SJ, 2014, AGE AGEING, V43, P653, DOI 10.1093/ageing/afu020

Song J, 2006, J WOMENS HEALTH, V15, P1205, DOI 10.1089/jwh.2006.15.1205

Torres-Bondia F, 2022, BMC PUBLIC HEALTH, V22, DOI 10.1186/s12889-022-13217-6

Wahking RA., 2018, OUTCOMES PHARMACIST, V53, P59

Waldum HL, 2010, SCAND J GASTROENTERO, V45, P389, DOI 10.3109/00365520903477348

Weisz G., 2015, PROTON PUMP INHIBITO, V8

NR 37

TC 0

Z9 0

U1 0

U2 0

PU SPRINGER HEIDELBERG

PI HEIDELBERG

PA TIERGARTENSTRASSE 17, D-69121 HEIDELBERG, GERMANY

SN 0031-6970

EI 1432-1041

J9 EUR J CLIN PHARMACOL

JI Eur. J. Clin. Pharmacol.

PD FEB

PY 2024

VL 80

IS 2

BP 273

EP 281

DI 10.1007/s00228-023-03607-z

EA DEC 2023

PG 9

WC Pharmacology & Pharmacy

WE Science Citation Index Expanded (SCI-EXPANDED)

SC Pharmacology & Pharmacy

GA GX8Q6

UT WOS:001129215000001

PM 38105298

DA 2024-08-08

ER

PT J

AU Bernabeu-Wittel, M

Para, O

Voicehovska, J

Gómez-Huelgas, R

Václavík, J

Battegay, E

Holecki, M

van Munster, BC

AF Bernabeu-Wittel, M.

Para, O.

Voicehovska, J.

Gomez-Huelgas, R.

Vaclavik, J.

Battegay, E.

Holecki, M.

van Munster, B. C.

CA EFIM Multimorbidity Working Grp

TI Competences of internal medicine specialists for the management of

patients with multimorbidity. EFIM multimorbidity working group position

paper

SO EUROPEAN JOURNAL OF INTERNAL MEDICINE

LA English

DT Article

DE Multimorbidity; Competences; Integrated care; Internal medicine

ID MULTIPLE CHRONIC CONDITIONS; INFORMED DECISION-MAKING; HEALTH-CARE;

MEDICATION ADHERENCE; FUNCTIONAL DECLINE; PROGNOSTIC INDEX; CHRONIC

DISEASES; PALLIATIVE CARE; CENTERED CARE; OLDER-PEOPLE

AB Patients with multimorbidity increasingly impact healthcare systems, both in primary care and in hospitals. This is particularly true in Internal Medicine. This population associates with higher mortality rates, polypharmacy, hospital readmissions, post-discharge syndrome, anxiety, depression, accelerated age-related functional decline, and development of geriatric syndromes, amongst others. Internists and Hospitalists, in one of their roles as Generalists, are increasingly asked to attend to these patients, both in their own Departments as well as in surgical areas. The management of polypathology and multimorbidity, however, is often complex, and requires specific clinical skills and corresponding experience. In addition, patients' needs, health-care environment, and routines have changed, so emerging and re-emerging specific competences and approaches are required to offer the best coordinated, continuous, and comprehensive integrated care to these populations, to achieve optimal health outcomes and satisfaction of patients, their relatives, and staff. This position paper proposes a set of emerging and re-emerging competences for internal medicine specialists, which are needed to optimally address multimorbidity now and in the future.

C1 [Bernabeu-Wittel, M.] Univ Seville, Hosp Univ Virgen Rocio, Dept Med, Internal Med Dept, Seville, Spain.

[Para, O.] Azienda Osped Univ Careggi, Florence, Italy.

[Voicehovska, J.] Riga Stradins Univ, Riga East Univ Hosp, Internal Dis Dept, Nephrol & Renal replacement therapy Clin, Riga, Latvia.

[Gomez-Huelgas, R.] Univ Malaga, Hosp Univ Reg Malaga, Internal Med Dept, Dept Med, Malaga, Spain.

[Vaclavik, J.] Univ Hosp Ostrava, Dept Internal Med & Cardiol, Ostrava, Czech Republic.

[Vaclavik, J.] Ostrava Univ, Fac Med, Ostrava, Czech Republic.

[Battegay, E.] Univ Zurich, Int Ctr Multimorbid & Complex ICMC, Zurich, Switzerland.

[Battegay, E.] Univ Hosp Basel, Dept Psychosomat Med, Basel, Switzerland.

[Battegay, E.] Merian Iselin Klin, Basel, Switzerland.

[Holecki, M.] Med Univ Silesiaia, Dept Internal Autoimmune & Metab Dis, Katowice, Poland.

[van Munster, B. C.] Univ Groningen, Univ Med Ctr Groningen, Dept Geriatr Med, Groningen, Netherlands.

C3 University of Sevilla; Virgen del Rocio University Hospital; University

of Florence; Azienda Ospedaliero Universitaria Careggi; Riga East

University Hospital; Riga Stradins University; Universidad de Malaga;

University Hospital Ostrava; University of Ostrava; University of

Zurich; University of Basel; Medical University Silesia; University of

Groningen

RP Bernabeu-Wittel, M (corresponding author), Univ Seville, Hosp Univ Virgen Rocio, Dept Med, Internal Med Dept, Seville, Spain.

OI Holecki, Michal/0000-0002-3289-1050

CR Afshar S, 2015, BMC PUBLIC HEALTH, V15, DOI 10.1186/s12889-015-2008-7

Albarqouni L, 2018, JAMA NETW OPEN, V1, DOI 10.1001/jamanetworkopen.2018.0281

[Anonymous], REDUCING MEDICATIONS

[Anonymous], HLTH EXPECT

[Anonymous], PERSONALISED CARE

[Anonymous], SHARED DECISION MAKI

Barbato A, 2022, J EVAL CLIN PRACT, V28, P371, DOI 10.1111/jep.13674

Barnett K, 2012, LANCET, V380, P37, DOI 10.1016/S0140-6736(12)60240-2

Bernabeu-Wittel M, 2022, EUR J INTERN MED, V100, P130, DOI 10.1016/j.ejim.2022.02.008

Bernabeu-Wittel M, 2017, REV CLIN ESP, V217, P410, DOI 10.1016/j.rce.2017.01.011

Bernabeu-Wittel M, 2011, ARCH GERONTOL GERIAT, V53, P284, DOI 10.1016/j.archger.2010.12.006

Bernabeu-Wittel M, 2011, EUR J INTERN MED, V22, P311, DOI 10.1016/j.ejim.2010.11.012

Bernabeu-Wittel M, 2010, ARCH GERONTOL GERIAT, V51, P185, DOI 10.1016/j.archger.2009.10.006

Bernabeu-Wittel M, 2014, J PAIN SYMPTOM MANAG, V47, P551, DOI 10.1016/j.jpainsymman.2013.04.011

Bernabeu-Wittel M, 2013, J AM GERIATR SOC, V61, P475, DOI 10.1111/jgs.12142

Bernabeu-Wittelt M, 2012, INT J GERONTOL, V6, P68, DOI 10.1016/j.ijge.2011.09.038

Braddock CH, 1997, J GEN INTERN MED, V12, P339, DOI 10.1007/s11606-006-5081-x

Braddock CH, 1999, JAMA-J AM MED ASSOC, V282, P2313, DOI 10.1001/jama.282.24.2313

Brown MT, 2016, AM J MED SCI, V351, P387, DOI 10.1016/j.amjms.2016.01.010

Bunn F, 2018, BMC GERIATR, V18, DOI 10.1186/s12877-018-0853-9

Carey IM, 2013, J CLIN EPIDEMIOL, V66, P436, DOI 10.1016/j.jclinepi.2012.10.012

Piñeiro-Fernández JC, 2022, INTERN EMERG MED, V17, P789, DOI 10.1007/s11739-021-02876-9

Coleman K, 2009, HEALTH AFFAIR, V28, P75, DOI 10.1377/hlthaff.28.1.75

Conn VS, 2017, PREV MED, V99, P269, DOI 10.1016/j.ypmed.2017.03.008

Corrao G, 2017, BMJ OPEN, V7, DOI 10.1136/bmjopen-2017-019503

Coulter A, 2015, COCHRANE DB SYST REV, DOI 10.1002/14651858.CD010523.pub2

Cranston M, 2013, EUR J INTERN MED, V24, P627, DOI 10.1016/j.ejim.2013.08.005

de Jonghe A, 2014, J PSYCHOSOM RES, V76, P193, DOI 10.1016/j.jpsychores.2013.12.007

Dellasega C, 2012, DIABETES RES CLIN PR, V95, P37, DOI 10.1016/j.diabres.2011.08.011

Dharmarajan K, 2013, JAMA-J AM MED ASSOC, V309, P355, DOI 10.1001/jama.2012.216476

Di Angelantonio E, 2015, JAMA-J AM MED ASSOC, V314, P52, DOI 10.1001/jama.2015.7008

Donzé J, 2013, BMJ-BRIT MED J, V347, DOI 10.1136/bmj.f7171

DuGoff EH, 2014, MED CARE, V52, P688, DOI 10.1097/MLR.0000000000000166

Eamer G, 2018, COCHRANE DB SYST REV, DOI 10.1002/14651858.CD012485.pub2

Ellis G, 2017, COCHRANE DB SYST REV, DOI 10.1002/14651858.CD006211.pub3

Elwyn G, 2014, ANN FAM MED, V12, P270, DOI 10.1370/afm.1615

Elwyn G, 2012, J GEN INTERN MED, V27, P1361, DOI 10.1007/s11606-012-2077-6

Elwyn G, 2010, BMJ-BRIT MED J, V341, DOI 10.1136/bmj.c5146

European Board of Internal Medicine, 2016, EUR CURR INT MED

European Board of Internal Medicine, US

Fatima S, 2021, PAK J MED SCI, V37, P2008, DOI 10.12669/pjms.37.7.3575

Moyano AF, 2017, REV CLIN ESP, V217, P351, DOI 10.1016/j.rce.2017.03.003

Foguet-Boreu Q, 2014, BMC FAM PRACT, V15, DOI 10.1186/1471-2296-15-55

Franchi C, 2022, INT J ENV RES PUB HE, V19, DOI 10.3390/ijerph19095692

Fulton Maryann M, 2005, J Am Acad Nurse Pract, V17, P123, DOI 10.1111/j.1041-2972.2005.0020.x

Galindo-Ocaña J, 2010, REV CLIN ESP, V210, P270, DOI 10.1016/j.rce.2009.12.008

Gibbins J, 2010, PALLIATIVE MED, V24, P299, DOI 10.1177/0269216309356029

Hajat Cother, 2018, Prev Med Rep, V12, P284, DOI 10.1016/j.pmedr.2018.10.008

Hanlon Peter, 2018, BJGP Open, V2, pbjgpopen18X101445, DOI 10.3399/bjgpopen18X101445

Hanlon P, 2018, LANCET PUBLIC HEALTH, V3, pE323, DOI 10.1016/S2468-2667(18)30091-4

Ho IS, 2021, LANCET PUBLIC HEALTH, V6, P587, DOI [10.1016/S2468-2667(21), DOI 10.1016/S2468-2667(21)]

Huibers CJA, 2022, EUR GERIATR MED, V13, P541, DOI 10.1007/s41999-022-00633-5

Janssen F, 2021, ELIFE, V10, DOI 10.7554/eLife.66590

Johnston MC, 2019, EUR J PUBLIC HEALTH, V29, P182, DOI 10.1093/eurpub/cky098

Kernick D, 2017, BRIT J GEN PRACT, V67, P235, DOI 10.3399/bjgp17X690857

Krumholz HM, 2013, NEW ENGL J MED, V368, P100, DOI 10.1056/NEJMp1212324

Kuipers SJ, 2021, INT J ENV RES PUB HE, V18, DOI 10.3390/ijerph18116057

Kwan JL, 2013, ANN INTERN MED, V158, P397, DOI 10.7326/0003-4819-158-5-201303051-00006

Lee SJ, 2006, JAMA-J AM MED ASSOC, V295, P801, DOI 10.1001/jama.295.7.801

Leiva-Fernández F, 2020, MECH AGEING DEV, V192, DOI 10.1016/j.mad.2020.111354

Maguire S, 2015, EDUC PRIM CARE, V26, P410, DOI 10.1080/14739879.2015.1101848

Makovski TT, 2019, AGEING RES REV, V53, DOI 10.1016/j.arr.2019.04.005

Mangin D, 2019, BMJ OPEN, V9, DOI 10.1136/bmjopen-2018-023731

Marengoni A, 2008, AM J PUBLIC HEALTH, V98, P1198, DOI 10.2105/AJPH.2007.121137

Marengoni A, 2011, AGEING RES REV, V10, P430, DOI 10.1016/j.arr.2011.03.003

Markun S, 2014, PLOS ONE, V9, DOI 10.1371/journal.pone.0110309

Mauksch LB, 2008, ARCH INTERN MED, V168, P1387, DOI 10.1001/archinte.168.13.1387

McNab D, 2018, BMJ QUAL SAF, V27, P308, DOI 10.1136/bmjqs-2017-007087

Melchiorre MG, 2020, BIOMED RES INT-UK, V2020, DOI 10.1155/2020/9025326

Molist-Brunet N, 2022, BMC GERIATR, V22, DOI 10.1186/s12877-022-03107-2

Muth C, 2019, J INTERN MED, V285, P272, DOI 10.1111/joim.12842

N'Goran Alexandra A, 2018, BMJ Open, V8, pe018281, DOI 10.1136/bmjopen-2017-018281

Nagel G, 2008, BMC PUBLIC HEALTH, V8, DOI 10.1186/1471-2458-8-384

Nobili Alessandro, 2011, J Comorb, V1, P28

O'Mahony D, 2015, AGE AGEING, V44, P213, DOI 10.1093/ageing/afu145

Ollero-Baturone M., PERSONALIZED ACTION

Onder G, 2015, EUR J INTERN MED, V26, P157, DOI 10.1016/j.ejim.2015.02.020

Payne RA, 2014, EUR J CLIN PHARMACOL, V70, P575, DOI 10.1007/s00228-013-1639-9

Pazan F, 2021, EUR GERIATR MED, V12, P443, DOI 10.1007/s41999-021-00479-3

Pefoyo AJK, 2015, BMC PUBLIC HEALTH, V15, DOI 10.1186/s12889-015-1733-2

Pilotto A, 2017, J AM MED DIR ASSOC, V18, DOI 10.1016/j.jamda.2016.11.004

Poblador-Plou B, 2014, PLOS ONE, V9, DOI 10.1371/journal.pone.0100375

Prados-Torres A, 2014, J CLIN EPIDEMIOL, V67, P254, DOI 10.1016/j.jclinepi.2013.09.021

Qato DM, 2016, JAMA INTERN MED, V176, P473, DOI 10.1001/jamainternmed.2015.8581

Quinn KL, 2020, JAMA-J AM MED ASSOC, V324, P1439, DOI 10.1001/jama.2020.14205

Rathert C., 2012, Medical Care Research Review

Rodríguez-Pérez A, 2019, EUR J HOSP PHARM, V26, P334, DOI 10.1136/ejhpharm-2017-001476

Rodríguez-Pérez A, 2017, GERIATR GERONTOL INT, V17, P2200, DOI 10.1111/ggi.13062

Santoro A, 2021, AGEING RES REV, V71, DOI 10.1016/j.arr.2021.101422

Savitz LA, 2021, HEALTH SERV RES, V56, P980, DOI 10.1111/1475-6773.13774

Schiotz Michaela L, 2016, J Comorb, V6, P95, DOI 10.15256/joc.2016.6.81

Schneider F, 2012, SWISS MED WKLY, V142, DOI 10.4414/smw.2012.13533

Schneider KM, 2009, HEALTH QUAL LIFE OUT, V7, DOI 10.1186/1477-7525-7-82

Silina V, 2018, EUR J GEN PRACT, V24, P112, DOI 10.1080/13814788.2018.1429594

Stacey Dawn, 2017, Cochrane Database Syst Rev, V4, pCD001431, DOI [10.1002/14651858.CD001431.pub3, 10.1002/14651858.CD001431.pub4, 10.1002/14651858.CD001431.pub5]

Stegmann ME, 2019, MATURITAS, V128, P49, DOI 10.1016/j.maturitas.2019.07.022

Stirland LE, 2020, BMJ-BRIT MED J, V368, DOI 10.1136/bmj.m160

Thienemann F, 2020, CARDIOVASC DIAGN THE, V10, P376, DOI 10.21037/cdt.2019.09.09

Tonelli MR, 2019, J EVAL CLIN PRACT, V25, P1057, DOI 10.1111/jep.13260

Turrillas P, 2019, J PAIN SYMPTOM MANAG, V57, P156, DOI 10.1016/j.jpainsymman.2018.09.020

Tziraki C, 2020, PRIM HEALTH CARE RES, V21, DOI 10.1017/S1463423620000328

VALLEJO MI, 2021, REV CLIN ESP BARC, V221, P347

van de Glind EMM, 2014, J AM GERIATR SOC, V62, P1133, DOI 10.1111/jgs.12830

van den Akker M, 1998, J CLIN EPIDEMIOL, V51, P367, DOI 10.1016/S0895-4356(97)00306-5

Van Grootven B, 2017, AGE AGEING, V46, P903, DOI 10.1093/ageing/afx051

van Munster BC, 2018, J EVAL CLIN PRACT, V24, P254, DOI 10.1111/jep.12738

van Seben R, 2020, J GERONTOL A-BIOL, V75, P1403, DOI 10.1093/gerona/glaa039

van Weert JCM, 2016, BMC MED INFORM DECIS, V16, DOI 10.1186/s12911-016-0281-8

von Gunten CF, 2000, JAMA-J AM MED ASSOC, V284, P3051, DOI 10.1001/jama.284.23.3051

Willadsen TG, 2016, SCAND J PRIM HEALTH, V34, P112, DOI 10.3109/02813432.2016.1153242

Wittel MB., 2022, J Biomed Res Environ Sci, V3, P1321

Xu XL, 2017, AGEING RES REV, V37, P53, DOI 10.1016/j.arr.2017.05.003

Zhou PL, 2018, BMC PUBLIC HEALTH, V18, DOI 10.1186/s12889-018-5408-7

Zissimopoulos JM, 2018, J GERONTOL B-PSYCHOL, V73, pS38, DOI 10.1093/geronb/gbx147

NR 114

TC 2

Z9 2

U1 3

U2 9

PU ELSEVIER

PI AMSTERDAM

PA RADARWEG 29, 1043 NX AMSTERDAM, NETHERLANDS

SN 0953-6205

EI 1879-0828

J9 EUR J INTERN MED

JI Eur. J. Intern. Med.

PD MAR

PY 2023

VL 109

BP 97

EP 106

DI 10.1016/j.ejim.2023.01.011

EA FEB 2023

PG 10

WC Medicine, General & Internal

WE Science Citation Index Expanded (SCI-EXPANDED)

SC General & Internal Medicine

GA 9X3QM

UT WOS:000949686800001

PM 36653235

OA Bronze, Green Published

DA 2024-08-08

ER

PT J

AU Blomaard, LC

Lucke, JA

de Gelder, J

Anten, S

Alsma, J

Schuit, SCE

Gussekloo, J

de Groot, B

Mooijaart, SP

AF Blomaard, L. C.

Lucke, J. A.

de Gelder, J.

Anten, S.

Alsma, J.

Schuit, S. C. E.

Gussekloo, J.

de Groot, B.

Mooijaart, S. P.

TI The APOP screener and clinical outcomes in older hospitalised internal

medicine patients

SO NETHERLANDS JOURNAL OF MEDICINE

LA English

DT Article

DE Frailty; geriatric emergency medicine; internal medicine; older people;

risk stratification

ID EMERGENCY-DEPARTMENT; FUNCTIONAL DECLINE; INDEX; FRAILTY; ADULTS

AB Background: Acutely hospitalised older patients with indications related to internal medicine have high risks of adverse outcomes. We investigated whether risk stratification using the Acutely Presenting Older Patient (APOP) screening tool associates with clinical outcomes in this patient group.

Methods: Patients aged >= 70 years who visited the Emergency Department (ED) and were acutely hospitalised for internal medicine were followed prospectively. The APOP screener assesses demographics, physical and cognitive function at ED presentation, and predicts 3-month mortality and functional decline in the older ED population. Patients with a predicted risk >= 45% were considered 'high risk'. Clinical outcome was hospital length of stay (LOS), and adverse outcomes were mortality and functional decline, 3 and 12 months after hospitalisation.

Results: We included 319 patients, with a median age of 80 (IQR 74-85) years, of whom 94 (29.5%) were categorised as 'high risk' by the APOP screener. These patients had a longer hospital LOS compared to 'low risk' patients (5 (IQR 3-10) vs. 3 (IQR 1-7) days, respectively; p = 0.006). At 3 months, adverse outcomes were more frequent in 'high risk' patients compared to 'low risk' patients (59.6% vs. 34.7%, respectively; p < 0.001). At 12 months, adverse outcomes (67.0% vs. 46.2%, respectively; p = 0.001) and mortality (48.9% vs. 28.0%, respectively; p < 0.001) were greater in 'high risk' compared to 'low risk' patients.

Conclusion: The APOP screener identifies acutely hospitalised internal medicine patients at high risk for poor short and long-term outcomes. Early risk stratification at admission could aid in individualised treatment decisions to optimise outcomes for older patients.

C1 [Blomaard, L. C.; de Gelder, J.; Gussekloo, J.; Mooijaart, S. P.] Leiden Univ, Med Ctr, Dept Internal Med, Sect Gerontol & Geriatr, Leiden, Netherlands.

[Lucke, J. A.; de Groot, B.] Leiden Univ, Med Ctr, Dept Emergency Med, Leiden, Netherlands.

[Lucke, J. A.] Spaarne Hosp, Dept Emergency Med, Haarlem, Netherlands.

[de Gelder, J.; Gussekloo, J.] Leiden Univ, Dept Publ Hlth & Primary Care, Med Ctr, Leiden, Netherlands.

[Anten, S.] Alrijne Hosp, Dept Internal Med, Sect Acute Care, Leiderdorp, Netherlands.

[Alsma, J.; Schuit, S. C. E.] Erasmus MC, Dept Internal Med, Rotterdam, Netherlands.

[Mooijaart, S. P.] IEMO, Inst Evidence Based Med Old Age, Leiden, Netherlands.

C3 Leiden University; Leiden University Medical Center (LUMC); Leiden

University - Excl LUMC; Leiden University; Leiden University Medical

Center (LUMC); Leiden University - Excl LUMC; Spaarne Hospital; Leiden

University; Leiden University Medical Center (LUMC); Leiden University -

Excl LUMC; Erasmus University Rotterdam; Erasmus MC

RP Blomaard, LC (corresponding author), Leiden Univ, Med Ctr, Dept Internal Med, Sect Gerontol & Geriatr, Leiden, Netherlands.

EM l.c.blomaard@lumc.nl

RI Alsma, Jelmer/O-7165-2019; Gussekloo, Jacobijn/ABE-3879-2021; Mooijaart,

Simon P./A-5306-2008

OI Alsma, Jelmer/0000-0002-2808-1514; Gussekloo,

Jacobijn/0000-0001-7186-8278; Mooijaart, Simon P./0000-0003-3106-3568;

Schuit, Stephanie/0000-0003-1200-9961

FU Dutch Ministry of Health, Welfare and Sport; Netherlands Organisation

for Health Research and Development (ZonMw) [62700.3001, 62700.4001]

FX The Institute for Evidence-Based Medicine in Old Age (IEMO) is supported

by the Dutch Ministry of Health, Welfare and Sport and by the

Netherlands Organisation for Health Research and Development (ZonMw

project number 62700.3001 and 62700.4001).

CR Admi H, 2015, RAMBAM MAIMONIDES ME, V6, DOI 10.5041/RMMJ.10201

[Anonymous], 1997, MANCHESTER TRIAGE GR

Basic D, 2015, J AGING HEALTH, V27, P670, DOI 10.1177/0898264314558202

Boyd CM, 2008, J AM GERIATR SOC, V56, P2171, DOI 10.1111/j.1532-5415.2008.02023.x

Buurman BM, 2016, JAMA INTERN MED, V176, P302, DOI 10.1001/jamainternmed.2015.8042

Buurman BM, 2012, PLOS ONE, V7, DOI 10.1371/journal.pone.0029621

Buurman BM, 2011, PLOS ONE, V6, DOI 10.1371/journal.pone.0026951

Buurman BM, 2008, J GEN INTERN MED, V23, P1883, DOI 10.1007/s11606-008-0741-7

Carpenter CR, 2015, ACAD EMERG MED, V22, P1, DOI 10.1111/acem.12569

Covinsky KE, 2003, J AM GERIATR SOC, V51, P451, DOI 10.1046/j.1532-5415.2003.51152.x

Davies S, 2020, INT J GERIATR PSYCH, V35, P53, DOI 10.1002/gps.5214

de Gelder J, 2018, EXP GERONTOL, V110, P253, DOI 10.1016/j.exger.2018.06.015

de Gelder J, 2017, NETH J MED, V75, P379

de Gelder J, 2016, NETH J MED, V74, P342

Ellis G, 2011, COCHRANE DB SYST REV, DOI 10.1002/14651858.CD006211.pub2

Evans SJ, 2014, AGE AGEING, V43, P127, DOI 10.1093/ageing/aft156

INOUYE SK, 1993, J GEN INTERN MED, V8, P645, DOI 10.1007/BF02598279

Jorgensen R, 2017, EUR J INTERN MED, V45, P71, DOI 10.1016/j.ejim.2017.09.036

KATZ S, 1963, JAMA-J AM MED ASSOC, V185, P914, DOI 10.1001/jama.1963.03060120024016

KATZMAN R, 1983, AM J PSYCHIAT, V140, P734

Meyer GS, 2012, BMJ QUAL SAF, V21, P964, DOI 10.1136/bmjqs-2012-001081

Parker SG, 2017, AGE AGEING, V46, P713, DOI 10.1093/ageing/afx104

Piers RD, 2013, PATIENT EDUC COUNS, V90, P323, DOI 10.1016/j.pec.2011.07.008

Theou O, 2018, BMC GERIATR, V18, DOI 10.1186/s12877-018-0823-2

van Deudekom FJ, 2017, PLOS ONE, V12, DOI [10.1371/.journal.pone.0174053, 10.1371/journal.pone.0174053]

van Seben R, 2019, J AM MED DIR ASSOC, V20, P152, DOI 10.1016/j.jamda.2018.08.003

NR 26

TC 10

Z9 10

U1 0

U2 0

PU VAN ZUIDEN COMMUNICATIONS

PI ALPHEN AAN DE RIJN

PA HENRY DUNANTWEG 40A, 2402 NR ALPHEN AAN DE RIJN, NETHERLANDS

SN 0300-2977

EI 1872-9061

J9 NETH J MED

JI Neth. J. Med.

PD FEB

PY 2020

VL 78

IS 1

BP 25

EP 33

PG 9

WC Medicine, General & Internal

WE Science Citation Index Expanded (SCI-EXPANDED); Social Science Citation Index (SSCI)

SC General & Internal Medicine

GA KM1NE

UT WOS:000513885300004

PM 32043475

DA 2024-08-08

ER

PT J

AU High, KP

Clayton, CP

Woolard, N

Regenstreif, DI

Lundebjerg, N

Hazzard, WR

AF High, KP

Clayton, CP

Woolard, N

Regenstreif, DI

Lundebjerg, N

Hazzard, WR

TI Expanding geriatrics into subspecialty internal medicine healthcare,

research and education: Caring for older adults and the T. Franklin

Williams Career Development Awards

SO AMERICAN JOURNAL OF MEDICINE

LA English

DT Article

C1 Wake Forest Univ, Sch Med, Dept Med, Winston Salem, NC 27109 USA.

Amer Geriatr Soc, Profess Educ Project, New York, NY USA.

Amer Geriatr Soc, Special Project, New York, NY USA.

Dept Vet Affairs Puget Sound Hlth Care Syst, Geriatr Program, Seattle, WA USA.

Dept Vet Affairs Puget Sound Hlth Care Syst, Extended Care Program, Seattle, WA USA.

C3 Wake Forest University

RP High, KP (corresponding author), Wake Forest Univ, Sch Med, Dept Med, Winston Salem, NC 27109 USA.

OI Clayton, Charles/0000-0003-1945-0477

CR Angus DC, 2000, JAMA-J AM MED ASSOC, V284, P2762, DOI 10.1001/jama.284.21.2762

Costa ST, 2001, AM J MED, V111, P674, DOI 10.1016/S0002-9343(01)01021-X

Hazzard WR, 1997, J AM GERIATR SOC, V45, P638, DOI 10.1111/j.1532-5415.1997.tb03102.x

Joiner KA, 2002, AM J MED, V112, P249, DOI 10.1016/S0002-9343(01)01125-1

*POP DIV DEP EC SO, AG WORLDS POP

Solomon DH, 2000, J AM GERIATR SOC, V48, P702, DOI 10.1111/j.1532-5415.2000.tb04734.x

NR 6

TC 12

Z9 13

U1 0

U2 0

PU EXCERPTA MEDICA INC

PI NEW YORK

PA 650 AVENUE OF THE AMERICAS, NEW YORK, NY 10011 USA

SN 0002-9343

J9 AM J MED

JI Am. J. Med.

PD OCT 15

PY 2002

VL 113

IS 6

BP 533

EP 536

AR PII S0002-9343(02)01348-7

DI 10.1016/S0002-9343(02)01348-7

PG 4

WC Medicine, General & Internal

WE Science Citation Index Expanded (SCI-EXPANDED)

SC General & Internal Medicine

GA 613TY

UT WOS:000179148600020

PM 12427511

DA 2024-08-08

ER

PT J

AU Shaffer, SL

Day, HD

AF Shaffer, Stacey L.

Day, Hollis D.

TI Systematic Outpatient Screening for the Elderly: Care of the Vulnerable

Elderly Practice Improvement Module to Assess Resident Care of Older

Adults

SO JOURNAL OF THE AMERICAN GERIATRICS SOCIETY

LA English

DT Article

DE medical education-graduate; geriatrics; ambulatory care

ID COMPETENCE

AB OBJECTIVES: To determine the feasibility of using the American Board of Internal Medicine Care of the Vulnerable Elderly Practice Improvement Module (CoVE PIM) in an internal medicine residency program and to assess aggregate resident documentation of geriatric screening in continuity clinics.

DESIGN: Needs assessment chart review for single-site pre-/postintervention study.

SETTING: Internal medicine resident primary care continuity clinics.

PARTICIPANTS: Thirty-seven postgraduate year (PGY)-1 and PGY-2 internal medicine residents.

MEASUREMENTS: Completion rate and time of CoVE PIM chart review, CoVE PIM user difficulty, and aggregate percentage of charts documenting geriatric screening measures.

RESULTS: Sixty-five percent of residents completed the CoVE PIM in an average of 47 minutes (range 30-90 minutes); 72% of resident surveys rated the CoVE PIM as easy to use. Residents demonstrated very good documentation of chronic medical conditions, smoking status, height, weight, and blood pressure and poor documentation of falls and fall risk, hearing assessment, postural hypotension, balance, rigidity, bradykinesia, home safety assessment, seat belt counseling, code status, and surrogate decision-maker.

CONCLUSION: The CoVE PIM can be used to assess aggregate resident performance of geriatric screening measures. In resident clinics, general adult screening performed by nurses is well documented, whereas geriatric-specific screening performed by physicians is poorly documented. J Am Geriatr Soc 58:2173-2177, 2010.

C1 [Day, Hollis D.] Univ Pittsburgh, Div Gen Internal Med, Pittsburgh, PA 15261 USA.

[Shaffer, Stacey L.] Univ Pittsburgh, Div Geriatr Med, Pittsburgh, PA 15261 USA.

[Shaffer, Stacey L.; Day, Hollis D.] Dept Vet Affairs Med Ctr, Pittsburgh, PA USA.

C3 Pennsylvania Commonwealth System of Higher Education (PCSHE); University

of Pittsburgh; Pennsylvania Commonwealth System of Higher Education

(PCSHE); University of Pittsburgh

RP Day, HD (corresponding author), Univ Pittsburgh, Div Gen Internal Med, M211 Scaife Hall,3550 Terrace St, Pittsburgh, PA 15261 USA.

EM dayh@upmc.edu

OI Day, Hollis/0000-0001-8817-5160

FU ABIM

FX This material is the result of work supported with resources and the use

of facilities at the Veterans Affairs Pittsburgh Healthcare System,

Pittsburgh, Pennsylvania. The ABIM provided funding through the

provision of the CoVE PIM at no charge, and Frank Kroboth, the residency

program director, provided funding through provision of the educational

incentive. The John A. Hartford Foundation supported the statistician.

CR ACGME, ACGME common program requirements

Bragg EJ, 2005, ACAD MED, V80, P279, DOI 10.1097/00001888-200503000-00014

Frohna JG, 2004, TEACH LEARN MED, V16, P77, DOI 10.1207/s15328015tlm1601_16

MILLER GE, 1990, ACAD MED, V65, pS63, DOI 10.1097/00001888-199009000-00045

Thomas DC, 2003, ANN INTERN MED, V139, P628, DOI 10.7326/0003-4819-139-7-200310070-00037

NR 5

TC 5

Z9 6

U1 0

U2 6

PU WILEY

PI HOBOKEN

PA 111 RIVER ST, HOBOKEN 07030-5774, NJ USA

SN 0002-8614

EI 1532-5415

J9 J AM GERIATR SOC

JI J. Am. Geriatr. Soc.

PD NOV

PY 2010

VL 58

IS 11

BP 2173

EP 2177

DI 10.1111/j.1532-5415.2010.03134.x

PG 5

WC Geriatrics & Gerontology; Gerontology

WE Science Citation Index Expanded (SCI-EXPANDED); Social Science Citation Index (SSCI)

SC Geriatrics & Gerontology

GA 676WT

UT WOS:000283950100018

PM 21039370

DA 2024-08-08

ER

PT J

AU Ceylan, S

Oytun, MG

Bas, AO

Balci, C

Halil, MG

Cankurtaran, M

Dogu, BB

AF Ceylan, Serdar

Guner Oytun, Merve

Okyar Bas, Arzu

Balci, Cafer

Halil, Meltem Gulhan

Cankurtaran, Mustafa

Dogu, Burcu Balam

TI How does hospitalization affect the frailty status of geriatric

patients? Prospective study from internal medicine wards of a university

hospital

SO CHRONIC ILLNESS

LA English

DT Article; Early Access

DE Frailty; internal medicine; hospitalization; Clinical Frailty Scale;

readmission

ID OLDER-ADULTS

AB Objectives: Frailty is a dynamic process. Frailty in the baseline, discharge, and post-discharge are important in the management of patients. We aimed to see how hospitalization affects frailty and to evaluate its effects on health outcomes.Methods: It was conducted with patients aged 65 and over who were hospitalized in the internal medicine wards of a university hospital. Frailty was evaluated by Clinical Frailty Scale within the first 24 h of hospitalization, within 24 h before discharge, and at third months after discharge.Results: Ninety-six (57.8%) of patients at baseline, 79 (50.6%) at discharge, and 68 (47.9%) at 3 months were frail. According to baseline, 12 (7.7%) patients changed from frail to non-frail at discharge, while 4 (2.6%) patients became frail (p = 0.08). According to the baseline, 18 (12.5%) patients went from frail to non-frail at 3 months, while 7 (4.9%) patients turned frail (p = 0.04). In regression analysis, living with frailty at discharge and low education level increased re-hospitalization. Five or more are considered living with frailty.Discussion: Hospitalization may have positive effects on frailty in older adult patients hospitalized in internal medicine wards, the main effect is seen to be more significant in the post-discharge follow-up.

C1 [Ceylan, Serdar; Guner Oytun, Merve; Okyar Bas, Arzu; Balci, Cafer; Halil, Meltem Gulhan; Cankurtaran, Mustafa; Dogu, Burcu Balam] Hacettepe Univ, Fac Med, Dept Internal Med, Div Geriatr, Ankara, Turkiye.

[Ceylan, Serdar] Hacettepe Univ, Fac Med, Dept Internal Med, Div Geriatr, TR-06230 Ankara, Turkiye.

C3 Hacettepe University; Hacettepe University

RP Ceylan, S (corresponding author), Hacettepe Univ, Fac Med, Dept Internal Med, Div Geriatr, TR-06230 Ankara, Turkiye.

EM serdarceyla@gmail.com

RI Güner, Merve/ISA-1949-2023; HALIL, MELTEM GULHAN/I-9038-2013; Ceylan,

Serdar/AAD-7086-2021; BAŞ, Arzu OKYAR/AAE-6709-2022

OI Güner, Merve/0000-0002-7417-5415; Ceylan, Serdar/0000-0001-5885-4023;

BAŞ, Arzu OKYAR/0000-0002-1518-5939

FU We wish to thank the staff working in the IMWs of Hacettepe University

Hospitals.; Hacettepe University Hospitals

FX We wish to thank the staff working in the IMWs of Hacettepe University

Hospitals.

CR Abel GA, 2018, BLOOD, V131, P515, DOI 10.1182/blood-2017-09-746420

Andela RM, 2010, INT J NURS PRACT, V16, P14, DOI 10.1111/j.1440-172X.2009.01807.x

Arik G, 2015, ARCH GERONTOL GERIAT, V61, P344, DOI 10.1016/j.archger.2015.08.019

Beard JR, 2016, LANCET, V387, P2145, DOI 10.1016/S0140-6736(15)00516-4

Bolayir B, 2019, NUTR CLIN PRACT, V34, P297, DOI 10.1002/ncp.10082

Church S, 2020, BMC GERIATR, V20, DOI 10.1186/s12877-020-01801-7

Courtwright AM, 2019, CLIN TRANSPLANT, V33, DOI 10.1111/ctr.13694

Fried LP, 2001, J GERONTOL A-BIOL, V56, pM146, DOI 10.1093/gerona/56.3.M146

Gilbert T, 2018, LANCET, V391, P1775, DOI 10.1016/S0140-6736(18)30668-8

Hao QK, 2019, SCI REP-UK, V9, DOI 10.1038/s41598-018-38072-7

Hartley P, 2017, GERIATR GERONTOL INT, V17, P1063, DOI 10.1111/ggi.12827

Hoogendijk EO, 2019, LANCET, V394, P1365, DOI 10.1016/S0140-6736(19)31786-6

Isik EI, 2020, ANN GERIATR MED RES, V24, P35

Jiao J, 2020, BMC GERIATR, V20, DOI 10.1186/s12877-020-1496-1

Johnston MC, 2019, EUR J PUBLIC HEALTH, V29, P182, DOI 10.1093/eurpub/cky098

Kahlon S, 2015, CAN MED ASSOC J, V187, P799, DOI 10.1503/cmaj.150100

Landre B, 2019, J NUTR HEALTH AGING, V23, P466, DOI 10.1007/s12603-019-1186-x

Lang PO, 2009, GERONTOLOGY, V55, P539, DOI 10.1159/000211949

Liang YD, 2019, CLIN INTERV AGING, V14, P2249, DOI 10.2147/CIA.S225149

Ligthart-Melis GC, 2020, J AM MED DIR ASSOC, V21, P1216, DOI 10.1016/j.jamda.2020.03.006

McAdams-DeMarco MA, 2015, J AM GERIATR SOC, V63, P2152, DOI 10.1111/jgs.13657

McCoy KL, 2022, SURGERY, V171, P718, DOI 10.1016/j.surg.2021.10.055

Miguelena-Hycka J, 2022, ARCH GERONTOL GERIAT, V98, DOI 10.1016/j.archger.2021.104568

Özsürekci C, 2020, ACTA CLIN BELG, V75, P200, DOI 10.1080/17843286.2019.1597457

Pazan F, 2021, EUR GERIATR MED, V12, P443, DOI 10.1007/s41999-021-00479-3

Richards SM, 2019, PLOS ONE, V14, DOI 10.1371/journal.pone.0209499

Rockwood K, 2005, CAN MED ASSOC J, V173, P489, DOI 10.1503/cmaj.050051

Strain WD, 2021, DIABETES THER, V12, P1227, DOI 10.1007/s13300-021-01035-9

Vetrano DL, 2018, BMJ OPEN, V8, DOI 10.1136/bmjopen-2018-024406

Walston J, 2006, J AM GERIATR SOC, V54, P991, DOI 10.1111/j.1532-5415.2006.00745.x

World Health Organization, 2016, WHO CLIN CONS HLTH A

NR 31

TC 0

Z9 0

U1 1

U2 5

PU SAGE PUBLICATIONS LTD

PI LONDON

PA 1 OLIVERS YARD, 55 CITY ROAD, LONDON EC1Y 1SP, ENGLAND

SN 1742-3953

EI 1745-9206

J9 CHRONIC ILLN

JI Chronic Illn.

PD 2023 OCT 16

PY 2023

DI 10.1177/17423953231209461

EA OCT 2023

PG 10

WC Health Care Sciences & Services; Public, Environmental & Occupational

Health; Medicine, General & Internal

WE Science Citation Index Expanded (SCI-EXPANDED); Social Science Citation Index (SSCI)

SC Health Care Sciences & Services; Public, Environmental & Occupational

Health; General & Internal Medicine

GA U8CT3

UT WOS:001087033900001

PM 37844580

DA 2024-08-08

ER

PT J

AU Díez-Manglano, J

Palazón-Fraile, C

Díez-Massó, F

Martínez-Alvarez, R

Del Corral-Beamonte, E

Carreño-Borrego, P

Pueyo-Tejedor, P

Gomes-Martín, J

AF Diez-Manglano, Jesus

Palazon-Fraile, Claudia

Diez-Masso, Fabiola

Martinez-Alvarez, Rosa

Del Corral-Beamonte, Esther

Carreno-Borrego, Pilar

Pueyo-Tejedor, Pilar

Gomes-Martin, Javier

TI Factors Associated With Onset of Delirium Among Internal Medicine

Inpatients in Spain

SO NURSING RESEARCH

LA English

DT Article

DE delirium; geriatrics; hospitalization; nursing

ID ELDERLY-PATIENTS; INSTITUTIONALIZATION; SYMPTOMS; SYSTEM

AB Background: Delirium increases mortality and length of stay among hospital inpatients. Little is known about the incidence of delirium among inpatients receiving care in internal medicine nursing units in Spain.

Objectives: The aim of this study was to estimate frequency of delirium onset among internal medicine inpatients and identify factors associated with delirium onset using nursing records and administrative databases.

Methods: Retrospective cohort study of 744 patients hospitalized in an internal medicine department in October 2010 and January, May, and October 2011. Data concerning occurrence of delirium, age, gender, living in a nursing residence, Barthel Index of activities of daily living, Norton scale for pressure ulcer risk, intravenous fluid therapy, urinary catheterization, presence of pressure ulcers, major diagnostic category at discharge, length of stay, and mean weight in the diagnosis-related group were gathered for each patient. Backward stepwise logistic regression was used to identify factors associated with onset of delirium.

Results: Ninety-seven (13%) patients experienced delirium. Factors associated with delirium were age (OR = 1.03, 95% CI [1.01, 1.06]), Barthel Index (OR = 0.99. 95% CI [0.98, 0.99]), and urinary catheterization (OR = 2.00, 95% CI [1.19, 3.68]).

Conclusion: Increased age and presence of a urinary catheter were associated with increased onset of delirium, whereas higher levels of independence in activities of daily living were protective.

C1 [Diez-Manglano, Jesus; Palazon-Fraile, Claudia; Diez-Masso, Fabiola; Martinez-Alvarez, Rosa; Del Corral-Beamonte, Esther; Carreno-Borrego, Pilar; Pueyo-Tejedor, Pilar; Gomes-Martin, Javier] Hosp Royo Villanova, Dept Internal Med, Zaragoza 50015, Spain.

[Diez-Manglano, Jesus] Aragon Hlth Sci Inst, Res Grp Comorbid & Polypathol, Zaragoza, Spain.

RP Díez-Manglano, J (corresponding author), Hosp Royo Villanova, Avda San Gregorio 30, Zaragoza 50015, Spain.

EM jdiez@aragon.es

RI Díez-Manglano, J/AAC-3198-2020

OI Díez-Manglano, J/0000-0002-3132-2171; Garces Horna,

Vanesa/0000-0002-3485-0789

CR Martín RB, 2009, REV CLIN ESP, V209, P459, DOI 10.1016/S0014-2565(09)72630-X

Bernabeu-Wittel M, 2010, ARCH GERONTOL GERIAT, V51, P185, DOI 10.1016/j.archger.2009.10.006

Bourdel-Marchasson I, 2004, J GERONTOL A-BIOL, V59, P350

CAMERON DJ, 1987, J AM GERIATR SOC, V35, P1007, DOI 10.1111/j.1532-5415.1987.tb04004.x

Casademont J., 2011, MED CLIN, V138, P289

Chrispal Anugrah, 2010, J Assoc Physicians India, V58, P15

de Teran CMD, 2006, REV CLIN ESP, V206, P4, DOI 10.1157/13084760

Everett G, 2007, J GEN INTERN MED, V22, P662, DOI 10.1007/s11606-007-0148-x

Gokula RRM, 2004, AM J INFECT CONTROL, V32, P196, DOI 10.1016/j.ajic.2003.08.007

GUSTAFSON Y, 1991, J AM GERIATR SOC, V39, P760, DOI 10.1111/j.1532-5415.1991.tb02697.x

Inouye SK, 2001, ARCH INTERN MED, V161, P2467, DOI 10.1001/archinte.161.20.2467

Inouye SK, 1999, NEW ENGL J MED, V340, P669, DOI 10.1056/NEJM199903043400901

Inouye SK, 2003, ARCH INTERN MED, V163, P958, DOI 10.1001/archinte.163.8.958

Inouye SK, 1996, JAMA-J AM MED ASSOC, V275, P852, DOI 10.1001/jama.275.11.852

INOUYE SK, 1990, ANN INTERN MED, V113, P941, DOI 10.7326/0003-4819-113-12-941

Inouye SK, 1998, J GEN INTERN MED, V13, P234, DOI 10.1046/j.1525-1497.1998.00073.x

LAZARO L, 1995, MED CLIN-BARCELONA, V104, P329

Leslie DL, 2005, ARCH INTERN MED, V165, P1657, DOI 10.1001/archinte.165.14.1657

MAHONEY F I, 1965, Md State Med J, V14, P61

McCusker J, 2004, J AM GERIATR SOC, V52, P1744, DOI 10.1111/j.1532-5415.2004.52471.x

McCusker J, 2003, J AM GERIATR SOC, V51, P1539, DOI 10.1046/j.1532-5415.2003.51509.x

McCusker J, 2002, ARCH INTERN MED, V162, P457, DOI 10.1001/archinte.162.4.457

Moller JT, 1998, LANCET, V351, P857, DOI 10.1016/S0140-6736(97)07382-0

Norton D., 1979, INVESTIGATION GERIAT, V3

Robertsson A., 2002, Delirium in old age, P9

Siddiqi N, 2006, AGE AGEING, V35, P350, DOI 10.1093/ageing/afl005

Stevens LE, 1998, AUST NZ J PSYCHIAT, V32, P805, DOI 10.3109/00048679809073869

van Rompaey B, 2009, CRIT CARE, V13, DOI 10.1186/cc7892

Witlox J, 2010, JAMA-J AM MED ASSOC, V304, P443, DOI 10.1001/jama.2010.1013

NR 29

TC 11

Z9 14

U1 0

U2 4

PU LIPPINCOTT WILLIAMS & WILKINS

PI PHILADELPHIA

PA TWO COMMERCE SQ, 2001 MARKET ST, PHILADELPHIA, PA 19103 USA

SN 0029-6562

EI 1538-9847

J9 NURS RES

JI Nurs. Res.

PD NOV-DEC

PY 2013

VL 62

IS 6

BP 445

EP 449

DI 10.1097/NNR.0000000000000004

PG 5

WC Nursing

WE Science Citation Index Expanded (SCI-EXPANDED); Social Science Citation Index (SSCI)

SC Nursing

GA 251QG

UT WOS:000326944400011

PM 24165221

OA Bronze

DA 2024-08-08

ER

PT J

AU Coni, N

AF Coni, N

TI A rude awakening: post-take ward round in the department of medicine for

the elderly

SO JOURNAL OF THE ROYAL SOCIETY OF MEDICINE

LA English

DT Article

AB This paper describes the post-take ward round of a department of medicine for the elderly (DME), to portray the nature of the medical admissions and their immediate management. The data concern the patients seen by one consultant in 28 such ward rounds during the last four months of 1997, in a teaching hospital where the DME is separate from the department of general internal medicine.

254 patients were seen, 107 men and 147 women, with an average age of 82.4 years (range 73-102). The decisions taken included diagnosis, further investigations, treatment, referral, discharge, and resuscitation status. Very few admissions were judged inappropriate, particularly among the majority referred by general practitioners. 101 patients were thought suitable for transfer to the department of general internal medicine, 109 definitely unsuitable.

These findings support the view that, if medical beds are to be freed, the initiative must come from facilitating discharge rather than curtailing admission. Generalists are needed to sort and manage these patients. In the UK, these will often be general internal medicine consultant geriatricians, while the younger patients are seen by consultants practising general internal medicine in addition to one of the specialties. Sizeable numbers of these consultants are needed if the post-take ward round is to be efficient and not conflict with their fixed commitments.

C1 Addenbrookes Hosp, Cambridge CB2 2QQ, England.

C3 Cambridge University Hospitals NHS Foundation Trust; Addenbrooke's

Hospital; University of Cambridge

RP Coni, N (corresponding author), Addenbrookes Hosp, Hills Rd, Cambridge CB2 2QQ, England.

CR *AUD COMM, 1997, COM AG

Capewell S, 1996, BRIT MED J, V312, P991

Hampton JR, 1998, J ROY COLL PHYS LOND, V32, P39

Houghton A, 1996, J ROY COLL PHYS LOND, V30, P551

Kafetz K, 1995, J ROY SOC MED, V88, P629

*ROYAL COLL PHYS N, 1997, TACKL NHS EM ADM

Smith HE, 1997, J ROY COLL PHYS LOND, V31, P527

NR 7

TC 1

Z9 1

U1 0

U2 4

PU ROYAL SOC MEDICINE PRESS LTD

PI LONDON

PA 1 WIMPOLE STREET, LONDON W1M 8AE, ENGLAND

SN 0141-0768

J9 J ROY SOC MED

JI J. R. Soc. Med.

PD SEP

PY 1998

VL 91

IS 9

BP 471

EP 474

DI 10.1177/014107689809100905

PG 4

WC Medicine, General & Internal

WE Science Citation Index Expanded (SCI-EXPANDED)

SC General & Internal Medicine

GA V2661

UT WOS:000165538200005

PM 9849517

OA Green Published, Bronze

DA 2024-08-08

ER

PT J

AU Litvin, CB

Davis, KS

Moran, WP

Iverson, PJ

Zhao, YM

Zapka, J

AF Litvin, Cara B.

Davis, Kimberly S.

Moran, William P.

Iverson, Patty J.

Zhao, Yumin

Zapka, Jane

TI The Use of Clinical Decision-Support Tools to Facilitate Geriatric

Education

SO JOURNAL OF THE AMERICAN GERIATRICS SOCIETY

LA English

DT Article

DE ambulatory care; clinical decision support; electronic health records;

geriatrics; medical education

ID MEDICAL-STUDENTS; QUALITY; RECORDS; SYSTEMS; CARE

AB Innovative methods are needed to incorporate effective geriatric education into internal medicine residency programs. The purpose of this report is to describe the development and use of clinical decision-support (CDS) tools to facilitate geriatric education and improve the care delivered to older adults in an academic internal medicine residency ambulatory care clinic. Starting in 2009, CDS tools were implemented as a major strategy of an initiative to improve resident physician clinical competencies in geriatrics and improve the quality of care and quality of life of older adults. These tools, designed to improve resident assessment and action for each of three educational modules (falls, vision, and dementia) were embedded within the ambulatory electronic medical record (EMR) and provided a method of point-of-care training to residents caring for older adults. One hundred internal medicine residents supervised by 17 general internal medicine faculty members participated. Data regarding CDS use and associated outcomes were recorded and extracted from the ambulatory clinic EMR. Residents screened between 67% and 88% of eligible patients using CDS algorithms; rates of additional assessment and referral or further examination reflected the prevalence of the condition in the patient population. Although further development may be necessary, CDS tools are a promising modality to supplement geriatric postgraduate education while simultaneously improving patient care.

C1 [Litvin, Cara B.] Med Univ S Carolina, Div Gen Internal Med & Geriatr, Dept Med, Charleston, SC 29425 USA.

[Zapka, Jane] Med Univ S Carolina, Div Gen Biostat & Epidemiol, Dept Med, Charleston, SC 29425 USA.

C3 Medical University of South Carolina; Medical University of South

Carolina

RP Litvin, CB (corresponding author), Med Univ S Carolina, Div Gen Internal Med & Geriatr, Dept Med, 135 Rutledge Ave,Suite 820, Charleston, SC 29425 USA.

EM litvincb@musc.edu

FU Donald W. Reynolds Foundation

FX This study was funded by the Donald W. Reynolds Foundation Aging and

Quality of Life program.

CR [Anonymous], 2008, Retooling for an aging America: Building the Healthcare Workforce

[Anonymous], 2009, AHRQ PUBLICATION

Arora V, 2005, AM J MED, V118, P680, DOI 10.1016/j.amjmed.2005.03.022

Blumenthal D, 2010, NEW ENGL J MED, V363, P501, DOI 10.1056/NEJMp1006114

Blumenthal D, 2009, NEW ENGL J MED, V360, P1477, DOI 10.1056/NEJMp0901592

Borson S, 2000, INT J GERIATR PSYCH, V15, P1021, DOI 10.1002/1099-1166(200011)15:11<1021::AID-GPS234>3.0.CO;2-6

Bryan Cathy, 2008, Inform Prim Care, V16, P79

Caton C, 2011, J AM GERIATR SOC, V59, P1941, DOI 10.1111/j.1532-5415.2011.03555.x

Drickamer MA, 2006, J GEN INTERN MED, V21, P1230, DOI 10.1111/j.1525-1497.2006.00585.x

Garg AX, 2005, JAMA-J AM MED ASSOC, V293, P1223, DOI 10.1001/jama.293.10.1223

Kawamoto K, 2005, BMJ-BRIT MED J, V330, P765, DOI 10.1136/bmj.38398.500764.8F

Keenan CR, 2006, ACAD PSYCHIATR, V30, P522, DOI 10.1176/appi.ap.30.6.522

Kho A, 2006, J GEN INTERN MED, V21, P531, DOI 10.1111/j.1525-1497.2006.00444.x

Leung GM, 2003, BMJ-BRIT MED J, V327, P1090, DOI 10.1136/bmj.327.7423.1090

Moran WP, 2012, ACAD MED IN PRESS

PODSIADLO D, 1991, J AM GERIATR SOC, V39, P142, DOI 10.1111/j.1532-5415.1991.tb01616.x

Riggio JM, 2009, ACAD MED, V84, P1719, DOI 10.1097/ACM.0b013e3181bf51d6

Sittig DF, 2008, J BIOMED INFORM, V41, P387, DOI 10.1016/j.jbi.2007.09.003

Warshaw GA, 2007, J AM GERIATR SOC, V55, P2075, DOI 10.1111/j.1532-5415.2007.01519.x

Wenger NS, 2003, ANN INTERN MED, V139, P740, DOI 10.7326/0003-4819-139-9-200311040-00008

Wenger NS, 2001, ANN INTERN MED, V135, P677, DOI 10.7326/0003-4819-135-8_Part_2-200110161-00006

NR 21

TC 14

Z9 16

U1 0

U2 9

PU WILEY-BLACKWELL

PI HOBOKEN

PA 111 RIVER ST, HOBOKEN 07030-5774, NJ USA

SN 0002-8614

J9 J AM GERIATR SOC

JI J. Am. Geriatr. Soc.

PD JUN

PY 2012

VL 60

IS 6

BP 1145

EP 1149

DI 10.1111/j.1532-5415.2012.03960.x

PG 5

WC Geriatrics & Gerontology; Gerontology

WE Science Citation Index Expanded (SCI-EXPANDED); Social Science Citation Index (SSCI)

SC Geriatrics & Gerontology

GA 957AW

UT WOS:000305131900022

PM 22642270

DA 2024-08-08

ER

PT J

AU Chen, XL

Shi, HN

Chang, JQ

Guo, WJ

Yang, YH

Wang, Y

Pan, L

AF Chen, Xiaolan

Shi, Hongning

Chang, Jiaqi

Guo, Wenjia

Yang, Yuanhua

Wang, Yong

Pan, Lei

TI External Validation of the Risk Assessment Model of Venous

Thromboembolism in Multicenter Internal Medicine Inpatients

SO CLINICAL AND APPLIED THROMBOSIS-HEMOSTASIS

LA English

DT Article

DE venous thromboembolism; risk assessment model; internal medicine

inpatients; multicenter; external validation

ID INTENSIVE-CARE-UNIT; CANCER-PATIENTS; VTE; PROPHYLAXIS; THROMBOSIS;

CAPRINI; DERIVATION

AB To external validate the risk assessment model (RAM) of venous thromboembolism (VTE) in multicenter internal medicine inpatients. We prospectively collected 595 internal medical patients (310 with VTE patients, 285 non-VTE patients) were from Beijing Shijitan Hospital, Beijing Chaoyang Hospital, and the respiratory department of Beijing Tsinghua Changgeng Hospital from January 2022 to December 2022 for multicenter external validation. The prediction ability of Caprini RAM, Padua RAM, The International Medical Prevention Registry on Venous Thromboembolism (IMPROVE) RAM, and Shijitan (SJT) RAM were compared. This study included a total of 595 internal medicine inpatients, including 242 (40.67%) in the respiratory department, 17 (2.86%) in the respiratory intensive care unit, 49 (8.24%) in the neurology department, 34 (5.71%) in the intensive care unit, 26 (4.37%) in the geriatric department, 22 (3.70%) in the emergency department, 71 (11.93%) in the nephrology department, 63 (10.59%) in the cardiology department, 24 (4.03%) in the hematology department, 6 (1.01%) in the traditional Chinese medicine department, 9 (1.51%) cases in the rheumatology department, 7 (1.18%) in the endocrinology department, 14 (2.35%) in the oncology department, and 11 (1.85%) in the gastroenterology department. Multivariate logistic regression analysis showed that among internal medicine inpatients, age > 60 years old, heart failure, nephrotic syndrome, tumors, history of VTE, and elevated D-dimer were significantly correlated with the occurrence of VTE (P < .05). The incidence of VTE increases with the increase of D-dimer. It was found that the effectiveness of SJT RAM (AUC = 0.80 +/- 0.03) was better than Caprini RAM (AUC = 0.74 +/- 0.03), Padua RAM (AUC = 0.72 +/- 0.03) and IMPROVE RAM (AUC = 0.52 +/- 0.03) (P < .05). The sensitivity and Yoden index of SJT RAM were higher than those of Caprini RAM, Pauda RAM, and IMPROVE RAM (P < .05), but specificity was not significantly different between the 4 models (P > .05). The SJT RAM derived from general hospitalized Chinese patients has effective and better predictive ability for internal medicine inpatients at risk of VTE.

C1 [Chen, Xiaolan; Wang, Yong; Pan, Lei] Capital Med Univ, Beijing Shijitan Hosp, Dept Resp & Crit Care Med, Beijing 100038, Peoples R China.

[Shi, Hongning] People Hosp Puer, Dept Hepatobiliary & Pancreat Surg, Puer, Peoples R China.

[Chang, Jiaqi] Nankai Univ, Sch Stat & Data Sci, Tianjin, Peoples R China.

[Guo, Wenjia] Beijing Tsinghua Changgung Hosp, Beijing, Peoples R China.

[Yang, Yuanhua] Capital Med Univ, Beijing Chaoyang Hosp, Dept Resp & Crit Care Med, Beijing 100020, Peoples R China.

[Wang, Yong] Beijing Red Cross Blood Ctr, Inst Blood Transfus, Beijing 100088, Peoples R China.

C3 Capital Medical University; Nankai University; Capital Medical

University; Chinese Academy of Medical Sciences - Peking Union Medical

College; Institute of Blood Transfusion - CAMS

RP Wang, Y; Pan, L (corresponding author), Capital Med Univ, Beijing Shijitan Hosp, Dept Resp & Crit Care Med, Beijing 100038, Peoples R China.; Yang, YH (corresponding author), Capital Med Univ, Beijing Chaoyang Hosp, Dept Resp & Crit Care Med, Beijing 100020, Peoples R China.; Wang, Y (corresponding author), Beijing Red Cross Blood Ctr, Inst Blood Transfus, Beijing 100088, Peoples R China.

EM yyh1031@sina.com; wangyong7096@aliyun.com; panlei@bjsjth.cn

FU Beijing Municipal Administration of Hospitals Incubating Program

FX No Statement Available

CR Abukhalil AD, 2022, VASC HEALTH RISK MAN, V18, P701, DOI 10.2147/VHRM.S382050

Arpaia GG, 2020, INTERN EMERG MED, V15, P997, DOI 10.1007/s11739-019-02264-4

Asmamaw M, 2022, SAGE OPEN MED, V10, DOI 10.1177/20503121221079488

Chen X., 2023, Clin Appl Thromb Hemost, V1, P1

Chen XL, 2021, ANN PALLIAT MED, V10, P4453, DOI 10.21037/apm-21-464

Darzi AJ, 2020, BLOOD ADV, V4, P4929, DOI 10.1182/bloodadvances.2020002482

Darzi AJ, 2020, BLOOD, V135, P1788, DOI 10.1182/blood.2019003603

de Bastos M, 2016, J THROMB THROMBOLYS, V41, P628, DOI 10.1007/s11239-015-1277-4

Greco S, 2021, EUR REV MED PHARMACO, V25, P2123, DOI 10.26355/eurrev_202102_25118

Hu YH, 2020, INTERACT CARDIOV TH, V31, P454, DOI 10.1093/icvts/ivaa137

Kandagatla P, 2019, J THROMB THROMBOLYS, V47, P566, DOI 10.1007/s11239-018-01801-w

Klen J, 2022, LIFE-BASEL, V12, DOI 10.3390/life12111843

Lavon O, 2022, SCI REP-UK, V12, DOI 10.1038/s41598-022-10209-9

Liu XH, 2016, INTERACT CARDIOV TH, V23, P538, DOI 10.1093/icvts/ivw158

Luo X Y, 2017, Zhonghua Yi Xue Za Zhi, V97, P1875, DOI 10.3760/cma.j.issn.0376-2491.2017.24.007

Peng H, 2020, J BURN CARE RES, V41, P113, DOI 10.1093/jbcr/irz121

Rothberg MB, 2022, THROMB HAEMOSTASIS, V122, P1231, DOI 10.1055/a-1698-6506

Schünemann HJ, 2018, BLOOD ADV, V2, P3198, DOI 10.1182/bloodadvances.2018022954

Shang MM, 2020, J THROMB THROMBOLYS, V50, P446, DOI 10.1007/s11239-020-02038-2

Shi J, 2019, J OBSTET GYNAECOL RE, V45, P657, DOI 10.1111/jog.13832

Song CF, 2019, EUR J CARDIO-THORAC, V55, P455, DOI 10.1093/ejcts/ezy323

Spyropoulos AC, 2021, RES PRACT THROMB HAE, V5, P296, DOI 10.1002/rth2.12486

Spyropoulos AC, 2011, CHEST, V140, P706, DOI 10.1378/chest.10-1944

Tafur AJ., 2016, Am J Med, V129

Wehmeyer A, 2022, SAMJ S AFR MED J, V112, P117, DOI 10.7196/SAMJ.2022.v112i2.16040

Wu ZQ, 2020, TRANSL ANDROL UROL, V9, P1904, DOI 10.21037/tau-20-320

Xu JX, 2018, J BUON, V23, P248

Zhai ZG, 2019, CHEST, V155, P114, DOI 10.1016/j.chest.2018.09.020

Zhang L, 2020, CIRCULATION, V142, P114, DOI 10.1161/CIRCULATIONAHA.120.046702

Zhou HX, 2018, J ATHEROSCLER THROMB, V25, P1091, DOI 10.5551/jat.43653

NR 30

TC 0

Z9 0

U1 0

U2 0

PU SAGE PUBLICATIONS INC

PI THOUSAND OAKS

PA 2455 TELLER RD, THOUSAND OAKS, CA 91320 USA

SN 1076-0296

EI 1938-2723

J9 CLIN APPL THROMB-HEM

JI Clin. Appl. Thromb.-Hemost.

PY 2024

VL 30

AR 10760296241247205

DI 10.1177/10760296241247205

PG 8

WC Hematology; Peripheral Vascular Disease

WE Science Citation Index Expanded (SCI-EXPANDED)

SC Hematology; Cardiovascular System & Cardiology

GA OC5R3

UT WOS:001205078400001

PM 38632943

OA gold, Green Published

DA 2024-08-08

ER

PT J

AU Baum, EE

Nelson, KM

AF Baum, Elizabeth E.

Nelson, Karl M.

TI The effect of a 12-month longitudinal long-term care rotation on

knowledge and attitudes of internal medicine residents about geriatrics

SO JOURNAL OF THE AMERICAN MEDICAL DIRECTORS ASSOCIATION

LA English

DT Article

DE geriatric; education; nursing home

ID OLDER PERSONS; STUDENTS; FELLOWS

AB Objective: To determine if participation in a 12-month longitudinal long-term care (LTC) rotation resulted in improved knowledge and attitudes about geriatrics.

Design: Longitudinal study with paired measurements.

Setting: A community LTC facility and a University-affiliated, community-based internal medicine residency program.

Participants: Sixty-seven internal medicine residents who participated in the rotation from 1997 through 2004.

ntervention: The internal medicine residents attended nursing home (NH) rounds one half day per month for 1 year, during which time they participated in a case-based interactive lecture on a core geriatric topic and rounded on their assigned patients.

Measurements: Knowledge was assessed using a 70-item test. Attitudes were evaluated with a 28-item, 5-point Likert scale (1 = least positive, 5 = most positive).

Results: The percent correct responses on geriatric knowledge pretest was 47% (95% CI = 45.2% to 48.8%) and on the posttest it was 57.5% (95% Cl = 55.3% to 59.6%) (t = 8.180, df = 67, P <.001). The pretest total attitude score was 3.6 (95% Cl = 3.6 to 3.7), with a posttest score of 3.7 (95% Cl = 3.7 to 3.8) (P <.001). The difference in this total was accounted for mainly by the significant changes in the attitude subscales in educational preparation (pretest 3.6 [95% CI = 3.5 to 3.8]; posttest 3.8 [95% CI = 3.7 to 3.9] [P < .001]), general attitudes (pretest 4.0 [95% Cl = 3.9 to 4.1]; posttest 4.2 [95 % CI = 4.0 to 4.3] [P = .006]), and therapeutic potential (pretest 3.7 [95% Cl = 3.5 to 3.8]; posttest 3.8 [95% CI = 3.7 to 3.9] [P = .048]).

Conclusion: A longitudinal LTC rotation is an efficient and effective way to systematically provide internal medicine residents their core knowledge and experience in geriatrics.

C1 Mercy Med Ctr, Canton, OH 44708 USA.

Aultman Hlth Fdn, Dept Grad Med Educ & Res, Canton, OH USA.

Northeastern Ohio Univ Coll Med & Pharm, Affiliated Hosp, Canton, OH USA.

C3 University System of Ohio; Northeast Ohio Medical University (NEOMED)

RP Baum, EE (corresponding author), Mercy Med Ctr, 1320 Mercy Dr NW, Canton, OH 44708 USA.

EM elizabeth.baum@csauh.com

CR [Anonymous], 2001, PROF OLD AM

Blumenthal D, 2001, JAMA-J AM MED ASSOC, V286, P1027, DOI 10.1001/jama.286.9.1027

DOBBS EL, 2002, CORE CURRICULUM GERI, V3

Ehrlich AR, 2003, J AM GERIATR SOC, V51, pS196

Fleming KC, 2003, MAYO CLIN PROC, V78, P1026

Hazzard WR, 2003, ANN INTERN MED, V139, P597, DOI 10.7326/0003-4819-139-7-200310070-00014

Irby DM, 2003, J GEN INTERN MED, V18, P370, DOI 10.1046/j.1525-1497.2003.21049.x

KEMPER P, 1991, NEW ENGL J MED, V324, P595, DOI 10.1056/NEJM199102283240905

Kishimoto M, 2005, J AM GERIATR SOC, V53, P99, DOI 10.1111/j.1532-5415.2005.53018.x

Lee M, 2005, J AM GERIATR SOC, V53, P489, DOI 10.1111/j.1532-5415.2005.53170.x

Lindberg MC, 1996, J GEN INTERN MED, V11, P397, DOI 10.1007/BF02600185

MAXWELL AJ, 1980, J AM GERIATR SOC, V28, P341, DOI 10.1111/j.1532-5415.1980.tb01095.x

PERROTTA P, 1981, J MED EDUC, V56, P478

RBERSON PK, 1995, FAM MED, V27, P671

REUBEN DB, 1995, J AM GERIATR SOC, V43, P1430, DOI 10.1111/j.1532-5415.1995.tb06626.x

Reuben DB, 1998, J AM GERIATR SOC, V46, P1425, DOI 10.1111/j.1532-5415.1998.tb06012.x

Thomas DC, 2003, ANN INTERN MED, V139, P628, DOI 10.7326/0003-4819-139-7-200310070-00037

NR 17

TC 12

Z9 12

U1 0

U2 4

PU ELSEVIER SCIENCE INC

PI NEW YORK

PA 360 PARK AVE SOUTH, NEW YORK, NY 10010-1710 USA

SN 1525-8610

J9 J AM MED DIR ASSOC

JI J. Am. Med. Dir. Assoc.

PD FEB

PY 2007

VL 8

IS 2

BP 105

EP 109

DI 10.1016/j.jamda.2006.05.009

PG 5

WC Geriatrics & Gerontology

WE Science Citation Index Expanded (SCI-EXPANDED)

SC Geriatrics & Gerontology

GA 138RX

UT WOS:000244381100008

PM 17289540

DA 2024-08-08

ER

PT J

AU Anani, S

Goldhaber, G

Brom, A

Lasman, N

Turpashvili, N

Shenhav-saltzman, G

Avaky, C

Negru, L

Agbaria, M

Ariam, S

Portal, D

Wasserstrum, Y

Segal, G

AF Anani, Sapir

Goldhaber, Gal

Brom, Adi

Lasman, Nir

Turpashvili, Natia

Shenhav-saltzman, Gilat

Avaky, Chen

Negru, Liat

Agbaria, Muhamad

Ariam, Sigalit

Portal, Doron

Wasserstrum, Yishay

Segal, Gad

TI Frailty and Sarcopenia Assessment upon Hospital Admission to Internal

Medicine Predicts Length of Hospital Stay and Re-Admission: A

Prospective Study of 980 Patients

SO JOURNAL OF CLINICAL MEDICINE

LA English

DT Article

DE sarcopenia; frailty; ALT; MAMC; frail questionnaire; internal medicine

ID ALL-CAUSE MORTALITY; ALANINE AMINOTRANSFERASE; OUTCOMES; CIRCUMFERENCE;

DISEASE; HEALTH; INDEX; MASS

AB Background: Frailty and sarcopenia are associated with frequent hospitalizations and poor clinical outcomes in geriatric patients. Ascertaining this association for younger patients hospitalized in internal medicine departments could help better prognosticate patients in the realm of internal medicine. Methods: During a 1-year prospective study in an internal medicine department, we evaluated patients upon admission for sarcopenia and frailty. We used the FRAIL questionnaire, blood alanine-amino transferase (ALT) activity, and mid-arm muscle circumference (MAMC) measurements. Results: We recruited 980 consecutive patients upon hospital admission (median age 72 years (IQR 65-79); 56.8% males). According to the FRAIL questionnaire, 106 (10.8%) patients were robust, 368 (37.5%) pre-frail, and 506 (51.7%) were frail. The median ALT value was 19IU/L (IQR 14-28). The median MAMC value was 27.8 (IQR 25.7-30.2). Patients with low ALT activity level (<17IU/L) were frailer according to their FRAIL score (3 (IQR 2-4) vs. 2 (IQR 1-3);p< 0.001). Higher MAMC values were associated with higher ALT activity, both representing robustness. The rate of 30 days readmission in the whole cohort was 17.4%. Frail patients, according to the FRAIL score (FS), had a higher risk for 30 days readmission (for FS > 2, HR = 1.99; 95CI = 1.29-3.08;p= 0.002). Frail patients, according to low ALT activity, also had a significantly higher risk for 30 days readmission (HR = 2.22; 95CI = 1.26-3.91;p= 0.006). After excluding patients whose length of stay (LOS) was >= 10 days, 252 (27.5%) stayed in-hospital for 4 days or longer. Frail patients according to FS had a higher risk for LOS >= 4 days (for FS > 2, HR = 1.87; 95CI = 1.39-2.52;p< 0.001). Frail patients, according to low ALT activity, were also at higher risk for LOS >= 4 days (HR = 1.87; 95CI = 1.39-2.52;p< 0.001). MAMC values were not correlated with patients' LOS or risk for re-admission. Conclusion: Frailty and sarcopenia upon admission to internal medicine departments are associated with longer hospitalization and increased risk for re-admission.

C1 [Anani, Sapir; Goldhaber, Gal; Brom, Adi; Lasman, Nir; Turpashvili, Natia; Shenhav-saltzman, Gilat; Avaky, Chen; Negru, Liat; Agbaria, Muhamad; Ariam, Sigalit; Wasserstrum, Yishay; Segal, Gad] Tel Aviv Univ, Sackler Fac Med, Chaim Sheba Med Ctr, Internal Med Dept T, POB 39040, IL-6997801 Tel Aviv, Israel.

[Portal, Doron] Tel Aviv Univ, Dept Internal Med, Sackler Fac Med, IL-6997801 Tel Aviv, Israel.

C3 Chaim Sheba Medical Center; Tel Aviv University; Tel Aviv University

RP Segal, G (corresponding author), Tel Aviv Univ, Sackler Fac Med, Chaim Sheba Med Ctr, Internal Med Dept T, POB 39040, IL-6997801 Tel Aviv, Israel.

EM sapir.anani@sheba.health.gov.il; gal.goldhaber@sheba.health.gov.il;

adi.brom@sheba.health.gov.il; nir.lasman@sheba.health.gov.il;

natia.turpashvili@sheba.health.gov.il;

gilat.shenhavzaltsman@sheba.health.gov.il;

chen.avaky@sheba.health.gov.il; Liat.negru@sheba.health.gov.il;

muhamad.agbaria@sheba.health.gov.il; sigalit.ariam@sheba.health.gov.il;

doron.portal@sheba.health.gov.il;

yishay.wasserstrum@sheba.health.gov.il; Gad.segal@sheba.health.gov.il

CR Atkins JL, 2014, J AM GERIATR SOC, V62, P253, DOI 10.1111/jgs.12652

Blomaard LC, 2020, NETH J MED, V78, P25

Buckinx F, 2018, J AM MED DIR ASSOC, V19, P18, DOI 10.1016/j.jamda.2017.06.014

Carnevale V, 2018, J AM MED DIR ASSOC, V19, P793, DOI 10.1016/j.jamda.2018.05.016

Cruz-Jentoft AJ, 2010, AGE AGEING, V39, P412, DOI 10.1093/ageing/afq034

Dieleman JL, 2020, JAMA-J AM MED ASSOC, V323, P863, DOI 10.1001/jama.2020.0734

FRISANCHO AR, 1981, AM J CLIN NUTR, V34, P2540, DOI 10.1093/ajcn/34.11.2540

Gentene AJ, 2021, J PHARM PRACT, V34, P110, DOI 10.1177/0897190019889440

Gringauz I, 2018, INT J REHABIL RES, V41, P41, DOI 10.1097/MRR.0000000000000258

Ibrahim AM, 2020, BMJ EVID-BASED MED, V25, P166, DOI 10.1136/bmjebm-2019-111271

Irina G, 2018, J CLIN MED, V7, DOI 10.3390/jcm7110386

Karapinar-Çarkt F, 2019, BMC HEALTH SERV RES, V19, DOI 10.1186/s12913-019-4617-9

Kim WR, 2008, HEPATOLOGY, V47, P1363, DOI 10.1002/hep.22109

Kogan M, 2018, J EXERC SCI FIT, V16, P1, DOI 10.1016/j.jesf.2017.11.002

Lasman N, 2020, BMC PULM MED, V20, DOI 10.1186/s12890-020-1169-z

Le Couteur DG, 2010, J GERONTOL A-BIOL, V65, P712, DOI 10.1093/gerona/glq082

Lembeck MA, 2019, BMC HEALTH SERV RES, V19, DOI 10.1186/s12913-019-4528-9

Luben RN, 2020, BMC GERIATR, V20, DOI 10.1186/s12877-020-01573-0

Marty E, 2017, BONE, V105, P276, DOI 10.1016/j.bone.2017.09.008

Morley JE, 2012, J NUTR HEALTH AGING, V16, P601, DOI 10.1007/s12603-012-0084-2

Nall RW, 2020, AM J MED, V133, pE260, DOI 10.1016/j.amjmed.2019.10.040

Nishikawa H, 2020, DIAGNOSTICS, V10, DOI 10.3390/diagnostics10060433

Noori N, 2010, CLIN J AM SOC NEPHRO, V5, P2258, DOI 10.2215/CJN.02080310

Peltz-Sinvani N, 2016, J GEN INTERN MED, V31, P209, DOI 10.1007/s11606-015-3480-6

Portal D, 2019, CANCER MANAG RES, V11, P2579, DOI 10.2147/CMAR.S195869

Ramati Erez, 2015, Harefuah, V154, P89

Ramaty E, 2014, EUR J INTERN MED, V25, P919, DOI 10.1016/j.ejim.2014.10.019

Senior JR, 2012, CLIN PHARMACOL THER, V92, P332, DOI 10.1038/clpt.2012.108

Singh M, 2014, EUR HEART J, V35, P1726, DOI 10.1093/eurheartj/ehu197

Sunkara PR, 2020, BMJ QUAL SAF, V29, P569, DOI 10.1136/bmjqs-2019-009936

Sykora Daniel, 2020, Crit Care Explor, V2, pe0103, DOI 10.1097/CCE.0000000000000103

Tosato M, 2017, AGING CLIN EXP RES, V29, P19, DOI 10.1007/s40520-016-0717-0

Vespasiani-Gentilucci U, 2018, J GERONTOL A-BIOL, V73, P925, DOI 10.1093/gerona/glx126

Wijnhoven HAH, 2010, J GERONTOL A-BIOL, V65, P1107, DOI 10.1093/gerona/glq100

Williams KH, 2016, METABOLISM, V65, P783, DOI 10.1016/j.metabol.2015.12.008

Wu LW, 2017, PLOS ONE, V12, DOI 10.1371/journal.pone.0171707

NR 36

TC 20

Z9 20

U1 0

U2 5

PU MDPI

PI BASEL

PA ST ALBAN-ANLAGE 66, CH-4052 BASEL, SWITZERLAND

EI 2077-0383

J9 J CLIN MED

JI J. Clin. Med.

PD AUG

PY 2020

VL 9

IS 8

AR 2659

DI 10.3390/jcm9082659

PG 12

WC Medicine, General & Internal

WE Science Citation Index Expanded (SCI-EXPANDED); Social Science Citation Index (SSCI)

SC General & Internal Medicine

GA NH5FD

UT WOS:000564694800001

PM 32824484

OA Green Published, gold

DA 2024-08-08

ER

PT J

AU Justo, D

Schwartz, N

Dvorkin, E

Gringauz, I

Groutz, A

AF Justo, Dan

Schwartz, Natalia

Dvorkin, Eliyahu

Gringauz, Irina

Groutz, Asnat

TI Asymptomatic urinary retention in elderly women upon admission to the

Internal Medicine department: A prospective study

SO NEUROUROLOGY AND URODYNAMICS

LA English

DT Article

DE elderly women; post-void residual urine; urinary retention; voiding

dysfunction

ID GERIATRIC VOIDING DYSFUNCTION; STRUCTURAL BASIS; RESIDUAL URINE;

DETRUSOR; MEN

AB AimTo assess the incidence and associated risk factors of asymptomatic urinary retention in elderly women upon admission to the Internal Medicine department.

MethodsTwo hundred and two consecutive elderly women (mean age 84.45.7 years) who were admitted to four Internal Medicine departments at a tertiary medical center were prospectively enrolled. All patients underwent post-void residual urine (PVR) measurements on the morning following the admission day. The measurements were undertaken by using a portable ultrasound bladder scan. Asymptomatic urinary retention was defined as PVR200ml without lower urinary tract symptoms, or abdominal pain, in two consecutive measurements.

ResultsAsymptomatic urinary retention was diagnosed in 29 (14.4%) women (mean PVR: 353.1 +/- 155.2ml; range: 200-712ml). The mean age, prevalence of chronic diseases, and the use of opioid and antimuscarinic drugs were similar in women with versus without asymptomatic urinary retention. A binary logistic regression analysis showed that asymptomatic urinary retention was significantly and independently associated with low mobility, measured by the functional independence measure (FIM) scale (odds ratio=0.7, 95% confidence interval 0.6-0.9, P=0.026), and hypothyroidism (odds ratio=2.4, 95% confidence interval 1.0-5.8, P=0.049). Among 174 (86.1%) patients in whom thyroid-stimulating hormone (TSH) serum levels were measured, a statistically significant correlation was demonstrated between TSH values and PVR measurements.

ConclusionsAsymptomatic urinary retention in elderly women upon admission to the Internal Medicine department is not infrequent and is independently associated with hypothyroidism and low mobility. PVR measurements should, therefore, be considered in all women with a low level of mobility and/or hypothyroidism upon admission to the Internal Medicine department. Neurourol. Urodynam. 36:794-797, 2017. (c) 2016 Wiley Periodicals, Inc.

C1 [Justo, Dan; Dvorkin, Eliyahu] Sheba Med Ctr, Dept Internal Med & Geriatr D, Tel Hashomer, Israel.

[Justo, Dan; Groutz, Asnat] Tel Aviv Univ, Sackler Sch Med, Tel Aviv, Israel.

[Schwartz, Natalia] Sheba Med Ctr, Dept Internal Med & Geriatr C, Tel Hashomer, Israel.

[Gringauz, Irina] Sheba Med Ctr, Dept Internal Med T, Tel Hashomer, Israel.

[Groutz, Asnat] Sourasky Med Ctr, Lis Matern Hosp, Urogynecol, Tel Aviv, Israel.

C3 Chaim Sheba Medical Center; Tel Aviv University; Chaim Sheba Medical

Center; Chaim Sheba Medical Center; Tel Aviv University; Tel Aviv

Sourasky Medical Center

RP Groutz, A (corresponding author), Tel Aviv Sourasky Med Ctr, Lis Matern Hosp, Urogynecol, 6 Weizman St, IL-6423906 Tel Aviv, Israel.

EM agroutz@yahoo.com

CR Asimakopoulos AD, 2016, NEUROUROL URODYNAM, V35, P55, DOI 10.1002/nau.22671

ELBADAWI A, 1993, J UROLOGY, V150, P1650, DOI 10.1016/S0022-5347(17)35866-4

ELBADAWI A, 1993, J UROLOGY, V150, P1657, DOI 10.1016/S0022-5347(17)35867-6

ELBADAWI A, 1993, J UROLOGY, V150, P1668, DOI 10.1016/S0022-5347(17)35868-8

GROSSHANS C, 1993, J AM GERIATR SOC, V41, P633, DOI 10.1111/j.1532-5415.1993.tb06736.x

HANSEN MV, 1988, SCAND J UROL NEPHROL, V22, P351, DOI 10.3109/00365598809180814

Kaplan SA, 2008, J UROLOGY, V180, P47, DOI 10.1016/j.juro.2008.03.027

Kong KH, 2000, ARCH PHYS MED REHAB, V81, P1464, DOI 10.1053/apmr.2000.9630

Lin ATL, 1997, J UROLOGY, V157, P1990, DOI 10.1016/S0022-5347(01)64916-4

Madersbacher S, 1998, UROLOGY, V51, P206, DOI 10.1016/S0090-4295(97)00616-X

NATHAN AW, 1982, BRIT MED J, V285, P477, DOI 10.1136/bmj.285.6340.477

Pedersen Hanne, 2010, Ugeskr Laeger, V172, P1512

Rodrigo C, 2011, THYROID RES, V4, DOI 10.1186/1756-6614-4-7

Shimoni Z, 2015, AM J MED, V128, P77, DOI 10.1016/j.amjmed.2014.08.018

Tobu S, 2014, GERIATR GERONTOL INT, V14, P636, DOI 10.1111/ggi.12150

NR 15

TC 11

Z9 12

U1 0

U2 4

PU WILEY

PI HOBOKEN

PA 111 RIVER ST, HOBOKEN 07030-5774, NJ USA

SN 0733-2467

EI 1520-6777

J9 NEUROUROL URODYNAM

JI Neurourol. Urodyn.

PD MAR

PY 2017

VL 36

IS 3

BP 794

EP 797

DI 10.1002/nau.23029

PG 4

WC Urology & Nephrology

WE Science Citation Index Expanded (SCI-EXPANDED)

SC Urology & Nephrology

GA EP6PO

UT WOS:000397501600046

PM 27176656

DA 2024-08-08

ER

PT J

AU Li, QZ

Zhao, YL

Chen, Y

Yue, JR

Xiong, Y

AF Li, Qinzheng

Zhao, Yanli

Chen, Yu

Yue, Jirong

Xiong, Yan

TI Developing a machine learning model to identify delirium risk in

geriatric internal medicine inpatients

SO EUROPEAN GERIATRIC MEDICINE

LA English

DT Article

DE Delirium; Elderly; Internal medicine; Machine learning; Predictive model

ID OLDER-PEOPLE; VALIDATION; EVENTS; SAMPLE; SCALE

AB Key summary pointsAim To develop a machine learning model that predicts delirium risk in geriatric internal medicine inpatients. Findings Depression, cognitive impairment, types of drugs, nutritional status, and activity of daily life (ADL) were important predictors of delirium. A machine learning model constructed from the above predictors achieved satisfactory discriminative ability. Message This machine learning model may allow more precise targeting of delirium prevention.

Purpose To develop a machine learning model that predicts delirium risk in geriatric internal medicine inpatients. Methods A prospective cohort study of internal medicine wards in a tertiary care hospital in China. Blinded observers assessed delirium using the Confusion Assessment Method (CAM). The data set was randomly divided into a training set (70%) and a test set (30%). The model was trained on the training set using the decision tree and the five-fold cross-validation, and then the model performance was evaluated on the test set. Under-sampling was used to address the class imbalance. The discriminatory power of the model was measured by the area under the receiver operating characteristic curve (AUC) and F1 score. The data set comprised 740 patients from March 2016 to January 2017. Results The training set included 518 patients; the median (IQR) age was 84 (79-87) years; 364 (70.3%) were men; 71 (13.7%) with delirium. The test set included 222 patients; the median (IQR) age was 84.5 (79-87) years; 163 (73.4%) were men; 30 (13.5%) with delirium. In total, the data set included 740 hospital admissions with a median (IQR) age of 84 (79-87) years, 527 (71.2%) were men, and 101 (13.6%) with delirium. From 32 potential predictors, we included five variables in the predictive model: depression, cognitive impairment, types of drugs, nutritional status, and activity of daily life (ADL). The mean AUC on the training set was 0.967, the AUC and F1 score on the test set was 0.950 and 0.810, respectively. The model achieved 93.3% sensitivity, 94.3% specificity, 71.8% positive predictive value, 98.9% negative predictive value, and 94.1% accuracy on the test set. Conclusion This machine learning model may allow more precise targeting of delirium prevention and could support clinical decision making in geriatric internal medicine wards.

C1 [Li, Qinzheng; Xiong, Yan] Sichuan Univ, Sch Mech Engn, Chengdu, Peoples R China.

[Zhao, Yanli; Yue, Jirong] Sichuan Univ, West China Hosp, Dept Geriatr, Chengdu, Peoples R China.

[Chen, Yu] Sichuan Univ, Dept Appl Mech, Chengdu, Peoples R China.

[Chen, Yu] Sichuan Univ, Med Big Data Ctr, Chengdu, Peoples R China.

C3 Sichuan University; Sichuan University; Sichuan University; Sichuan

University

RP Xiong, Y (corresponding author), Sichuan Univ, Sch Mech Engn, Chengdu, Peoples R China.; Yue, JR (corresponding author), Sichuan Univ, West China Hosp, Dept Geriatr, Chengdu, Peoples R China.

EM yuejirong11@hotmail.com; xy@scu.edu.cn

RI zhao, yan/GWC-9174-2022; , 月月/GMX-1381-2022

OI Yue, Jirong/0000-0002-3730-779X

FU National Key R&D Program of China [2020YFC2005600]; Project of Sichuan

Chengdu Science and Technology Bureau [2019-YF05-00622-SN]

FX We thank the National Key R&D Program of China (2020YFC2005600) and

Project of Sichuan Chengdu Science and Technology Bureau

(2019-YF05-00622-SN).

CR Ahmed S, 2014, AGE AGEING, V43, P326, DOI 10.1093/ageing/afu022

Martinez JA, 2012, BMJ OPEN, V2, DOI 10.1136/bmjopen-2012-001599

Batista G. E. A. P. A., 2004, SIGKDD Explorations: Newsletter of the Special Interest Group (SIG) on Knowledge Discovery Data Mining, V6, P20, DOI [10.1145/1007730.1007735, DOI 10.1145/1007730.1007735]

Carrasco MP, 2014, AGE AGEING, V43, P346, DOI 10.1093/ageing/aft141

Carrière I, 2009, ARCH INTERN MED, V169, P1317, DOI 10.1001/archinternmed.2009.229

Chawla NV, 2002, J ARTIF INTELL RES, V16, P321, DOI 10.1613/jair.953

Corradi JP, 2018, J MED SYST, V42, DOI 10.1007/s10916-018-1109-0

Davoudi A, 2017, IEEE INT C BIOINF BI, P568, DOI [10.1109/BIBE.2017.00103, 10.1109/BIBE.2017.00014]

Douglas VC, 2013, J HOSP MED, V8, P493, DOI 10.1002/jhm.2062

FP M, 2011, PSYCHIATR POL, V45, P211

García S, 2016, KNOWL-BASED SYST, V98, P1, DOI 10.1016/j.knosys.2015.12.006

Glass J, 2005, BMJ-BRIT MED J, V331, P1169, DOI 10.1136/bmj.38623.768588.47

Hein C, 2014, J AM MED DIR ASSOC, V15, DOI 10.1016/j.jamda.2014.08.012

Hercus C, 2020, BMC HEALTH SERV RES, V20, DOI 10.1186/s12913-020-5005-1

Hicks CL, 2001, PAIN, V93, P173, DOI 10.1016/S0304-3959(01)00314-1

Inouye SK, 2007, ARCH INTERN MED, V167, P1406, DOI 10.1001/archinte.167.13.1406

Inouye SK, 2014, LANCET, V383, P911, DOI 10.1016/S0140-6736(13)60688-1

INOUYE SK, 1990, ANN INTERN MED, V113, P941, DOI 10.7326/0003-4819-113-12-941

Isfandiaty Ratih, 2012, Acta Med Indones, V44, P290

Kalisvaart I, 2014, J GERIATR ONCOL, V5, pS48, DOI [10.1016/J.JGO.2014.09.082, DOI 10.1016/J.JGO.2014.09.082]

Liao L, 2003, J AM COLL CARDIOL, V42, P851, DOI 10.1016/S0735-1097(03)00836-2

MacLullich AMJ, 2017, NEW ENGL J MED, V45, P46, DOI [10.1016/J.MPMED.2016.10.006, DOI 10.1016/J.MPMED.2016.10.006]

McSherry D, 1999, KNOWL-BASED SYST, V12, P269, DOI 10.1016/S0950-7051(99)00024-6

NABEEL H, 2019, JMIR MED INF, V7

Nyunt MSZ, 2009, AGING MENT HEALTH, V13, P376, DOI 10.1080/13607860902861027

O'Sullivan R, 2014, LANCET PSYCHIAT, V1, P303, DOI 10.1016/S2215-0366(14)70281-0

Oh ES, 2017, JAMA-J AM MED ASSOC, V318, P1161, DOI 10.1001/jama.2017.12067

Oh J, 2018, PHYSIOL MEAS, V39, DOI 10.1088/1361-6579/aaab07

Peduzzi P, 1995, J CLIN EPIDEMIOL, V48, P1503, DOI 10.1016/0895-4356(95)00048-8

Peduzzi P, 1996, J CLIN EPIDEMIOL, V49, P1373, DOI 10.1016/S0895-4356(96)00236-3

PFEIFFER E, 1975, J AM GERIATR SOC, V23, P433, DOI 10.1111/j.1532-5415.1975.tb00927.x

PRAAGMAN J, 1985, EUR J OPER RES, V19, P144, DOI 10.1016/0377-2217(85)90321-2

Quinlan Ross., 1992, C4.5: Programs for Machine Learning

Racine AM, 2021, J GEN INTERN MED, V36, P265, DOI 10.1007/s11606-020-06238-7

Rubenstein LZ, 2001, J GERONTOL A-BIOL, V56, pM366, DOI 10.1093/gerona/56.6.M366

SHAH S, 1989, J CLIN EPIDEMIOL, V42, P703, DOI 10.1016/0895-4356(89)90065-6

Sokolova M, 2009, INFORM PROCESS MANAG, V45, P427, DOI 10.1016/j.ipm.2009.03.002

Stahlmann R, 2010, DRUG AGING, V27, P193, DOI 10.2165/11531490-000000000-00000

Viktil KK, 2007, BRIT J CLIN PHARMACO, V63, P187, DOI 10.1111/j.1365-2125.2006.02744.x

Vittinghoff E, 2007, AM J EPIDEMIOL, V165, P710, DOI 10.1093/aje/kwk052

Waljee AK, 2010, AM J GASTROENTEROL, V105, P1224, DOI 10.1038/ajg.2010.173

Wasikowski M, 2010, IEEE T KNOWL DATA EN, V22, P1388, DOI 10.1109/TKDE.2009.187

Wong A, 2018, JAMA NETW OPEN, V1, DOI 10.1001/jamanetworkopen.2018.1018

NR 43

TC 6

Z9 6

U1 1

U2 10

PU SPRINGER

PI NEW YORK

PA ONE NEW YORK PLAZA, SUITE 4600, NEW YORK, NY, UNITED STATES

SN 1878-7649

EI 1878-7657

J9 EUR GERIATR MED

JI Eur. Geriatr. Med.

PD FEB

PY 2022

VL 13

IS 1

BP 173

EP 183

DI 10.1007/s41999-021-00562-9

EA SEP 2021

PG 11

WC Geriatrics & Gerontology

WE Science Citation Index Expanded (SCI-EXPANDED); Social Science Citation Index (SSCI)

SC Geriatrics & Gerontology

GA ZE3YL

UT WOS:000698339900001

PM 34553310

DA 2024-08-08

ER

PT J

AU Snow, V

Beck, D

Budnitz, T

Miller, DC

Potter, J

Wears, RL

Weiss, KB

Williams, MV

AF Snow, Vincenza

Beck, Dennis

Budnitz, Tina

Miller, Doriane C.

Potter, Jane

Wears, Robert L.

Weiss, Kevin B.

Williams, Mark V.

TI Transitions of Care Consensus Policy Statement: American College of

Physicians, Society of General Internal Medicine, Society of Hospital

Medicine, American Geriatrics Society, American College of Emergency

Physicians, and Society for Academic Emergency Medicine

SO JOURNAL OF HOSPITAL MEDICINE

LA English

DT Article

DE care standardization; continuity of care transition and discharge

planning; patient safety

ID PATIENT SAFETY; ADVERSE EVENTS; QUALITY

AB The American College of Physicians, Society of Hospital Medicine, and Society of General Internal Medicine convened a multi-stakeholder consensus conference in July 2007 to address the quality gaps in the transitions between inpatient and outpatient settings and to develop consensus standards for these transitions. Over 30 organizations sent representatives to the Transitions of Care Consensus Conference. Participating organizations included medical specialty societies from internal medicine as well as family medicine and pediatrics, governmental agencies such as the Agency for Healthcare Research and Quality and the Centers for Medicare and Medicaid Services, performance measure developers such as the National Committee for Quality Assurance and the American Medical Association Physician Consortium on Performance Improvement, nurse associations such as the Visiting Nurse Associations of America and Home Care and Hospice, pharmacist groups, and patient groups such as the Institute for Family-Centered Care. The Transitions of Care Consensus Conference made recommendations for standards concerning the transitions between inpatient and outpatient settings for future implementation. The American College of Physicians, Society of Hospital Medicine, Society of General Internal Medicine, American Geriatric Society, American College of Emergency Physicians, and Society for Academic Emergency Medicine all endorsed this document. Journal of Hospital Medicine 2009;4:364-370. (C) 2009 Society of Hospital Medicine.

C1 [Snow, Vincenza] Amer Coll Physicians, Philadelphia, PA 19106 USA.

[Beck, Dennis] Beacon Med Serv, Aurora, CO USA.

[Budnitz, Tina] Soc Hosp Med, Philadelphia, PA USA.

[Miller, Doriane C.] Univ Chicago, Med Ctr, Chicago, IL 60637 USA.

[Potter, Jane] Univ Nebraska, Lincoln, NE USA.

[Wears, Robert L.] Univ Florida, Gainesville, FL USA.

[Weiss, Kevin B.] Amer Board Med Specialties, Chicago, IL USA.

[Williams, Mark V.] Northwestern Univ, Chicago, IL 60611 USA.

C3 American College of Physicians; University of Chicago; University of

Chicago Medical Center; University of Nebraska System; University of

Nebraska Lincoln; State University System of Florida; University of

Florida; Northwestern University

RP Snow, V (corresponding author), Amer Coll Physicians, 190 N Independence Mall W, Philadelphia, PA 19106 USA.

EM vincenza@acponline.org

FU Novo Nordisk; Agency for Healthcare Research and Quality

FX The Transitions of Care Consensus Conference was funded by an

unrestricted educational grant from Novo Nordisk as part of the American

College of Physicians Diabetes Initiative and by the Agency for

Healthcare Research and Quality. The funders had no input into the

planning, structure, content, participants, or outcomes of the

conference. This article is also being published by the Journal of

General Internal Medicine [Vincenza Snow, Dennis Beck, Tina Budnitz,

Doriane C. Miller, Jane Potter, Robert L. Wears, Kevin B. Weiss, Mark V

Williams. Transitions of Care Consensus Policy Statement American

College of Physic i a ns- Society of General Internal Medicine- Society

of Hospital Medicine -American Geriatrics Society-American College of

Emergency Physicians -Society of Academic Emergency Medicine]. J Gen

Intern Med 2009 (online in advance of print).

CR *AM BOARD INT MED, STEPP PLAT IN PRESS

*AM COLL PHYS, 2006, ADV MED HOM PATCTR P

Babbott SF, 2007, J GEN INTERN MED, V22, P400, DOI 10.1007/s11606-006-0082-3

Behara R., 2005, ADV PATIENT SAFETY, V2, P309

Coleman EA, 2005, ARCH INTERN MED, V165, P1842, DOI 10.1001/archinte.165.16.1842

Coleman EA, 2005, MED CARE, V43, P246, DOI 10.1097/00005650-200503000-00007

Coleman EA, 2004, ANN INTERN MED, V141, P533, DOI 10.7326/0003-4819-141-7-200410050-00009

Coleman Eric A, 2002, Int J Integr Care, V2, pe02

Cooper J B, 1989, J Clin Anesth, V1, P228, DOI 10.1016/0952-8180(89)90047-0

COOPER JB, 1982, ANESTHESIOLOGY, V56, P456, DOI 10.1097/00000542-198206000-00010

Feldman JA, 2003, ACAD EMERG MED, V10, P910

Forster AJ, 2003, ANN INTERN MED, V138, P161, DOI 10.7326/0003-4819-138-3-200302040-00007

Kripalani S, 2007, JAMA-J AM MED ASSOC, V297, P831, DOI 10.1001/jama.297.8.831

NAWAR EW, 2007, ADV DATA VITAL HLTH, V386

Roy CL, 2005, ANN INTERN MED, V143, P121, DOI 10.7326/0003-4819-143-2-200507190-00011

Sia CJ, 2002, PEDIATRICS, V110, P184

Starfield B, 2004, PEDIATRICS, V113, P1493

Tsilimingras D, 2008, JT COMM J QUAL PATIE, V34, P85, DOI 10.1016/S1553-7250(08)34011-2

vom Eigen KA, 1999, MED CARE, V37, P33, DOI 10.1097/00005650-199901000-00006

Wears R.L., 2003, P HUMAN FACTORS ERGO, P1420

WEARS RL, 2004, P P HUM FACT ERG SOC, P1625

NR 21

TC 128

Z9 129

U1 0

U2 15

PU FRONTLINE MEDICAL COMMUNICATIONS

PI THE WOODLANDS

PA WRIGHTS MEDIA, 2407 TIMBERLOCH PLACE, SUITE B, THE WOODLANDS, TX 77386

USA

SN 1553-5592

EI 1553-5606

J9 J HOSP MED

JI J. Hosp. Med.

PD JUL-AUG

PY 2009

VL 4

IS 6

BP 364

EP 370

DI 10.1002/jhm.510

PG 7

WC Medicine, General & Internal

WE Science Citation Index Expanded (SCI-EXPANDED)

SC General & Internal Medicine

GA 484SD

UT WOS:000269066800006

PM 19479781

OA hybrid

DA 2024-08-08

ER

PT J

AU Sonnenblick, M

Raveh, D

Gratch, L

Yinnon, A

AF Sonnenblick, M.

Raveh, D.

Gratch, L.

Yinnon, A.

TI Clinical and demographic characteristics of elderly patients

hospitalised in an internal medicine department in Israel

SO INTERNATIONAL JOURNAL OF CLINICAL PRACTICE

LA English

DT Article

ID FUNCTIONAL OUTCOMES; CONTROLLED-TRIAL; GERIATRIC EVALUATION; OLDER;

IMPROVE; CARE; INTERVENTION; INPATIENT; EDUCATION; UNIT

AB The number of elderly patients hospitalised in acute medical wards is increasing rapidly. It is important to understand the demographic and clinical characteristics of these patients so as to plan appropriate resource allocation and geriatric training programmes. The aim of the study is to describe the demographic and clinical characteristics of elderly patients admitted to an internal medicine department. During a 3-months prospective study, 779 patients over the age of 65 with acute illness admitted to internal medicine wards. The mean age of the patients was 80 +/- 8 years; 277 (36%) were defined as dependent. The latter had significantly more moderate or severe dementia, higher APACH II scores, lower serum albumin levels, needed more mechanical ventilation, a higher mortality rate and more prolonged admissions (for all variables p < 0.001). Infectious disease was the main indication for admission in the dependent patients while in the independent patients it was cardiac disease (54 vs. 29% and 17 vs. 45%, respectively, p < 0.001). In-hospital mortality was 11%. Functional capacity during hospitalisation declined significantly in all patients but more in those over the age of 85. Of those patients discharged from hospital, mental deterioration during the hospitalisation was observed in only 3%. Our study supports the concept of the introduction of specialised geriatric facilities within the general hospital framework, including the geriatric training and education of all residents in internal medicine.

C1 Ben Gurion Univ Negev, Fac Hlth Sci, Shaare Zedek Med Ctr, Dept Geriatr, IL-91031 Jerusalem, Israel.

Ben Gurion Univ Negev, Fac Hlth Sci, Shaare Zedek Med Ctr, Infect Dis Unit, IL-91031 Jerusalem, Israel.

Ben Gurion Univ Negev, Fac Hlth Sci, Shaare Zedek Med Ctr, Dept Med, IL-91031 Jerusalem, Israel.

C3 Hebrew University of Jerusalem; Shaare Zedek Medical Center; Ben Gurion

University; Ben Gurion University; Hebrew University of Jerusalem;

Shaare Zedek Medical Center; Hebrew University of Jerusalem; Shaare

Zedek Medical Center; Ben Gurion University

RP Sonnenblick, M (corresponding author), Ben Gurion Univ Negev, Fac Hlth Sci, Shaare Zedek Med Ctr, Dept Geriatr, POB 3235, IL-91031 Jerusalem, Israel.

EM son@szmc.org.il

RI Raveh, Dina/A-7166-2012

CR Asplund K, 2000, J AM GERIATR SOC, V48, P1381, DOI 10.1111/j.1532-5415.2000.tb02626.x

BOEHM R, 1994, ISRAEL J MED SCI, V30, P125

Cohen HJ, 2002, NEW ENGL J MED, V346, P905, DOI 10.1056/NEJMsa010285

Counsell SR, 2000, J AM GERIATR SOC, V48, P1572, DOI 10.1111/j.1532-5415.2000.tb03866.x

Elon RD, 2004, ANN INTERN MED, V140, P1061, DOI 10.7326/0003-4819-140-12-200406150-00025

Ferrara AM, 2004, DRUG AGING, V21, P167, DOI 10.2165/00002512-200421030-00003

FISHER R, 2002, GERIATRIA S14, V1, P18

Glantz S.A., 1992, PRIMER BIOSTATISTICS

HAKLAI Z, 2001, HLTH ISRAEL SELECTED

Hanlon JT, 1996, AM J MED, V100, P428, DOI 10.1016/S0002-9343(97)89519-8

Landefeld CS, 2003, ANN INTERN MED, V139, P609, DOI 10.7326/0003-4819-139-7-200310070-00034

LANDEFELD CS, 1995, NEW ENGL J MED, V332, P1338, DOI 10.1056/NEJM199505183322006

Lynn J, 1997, ANN INTERN MED, V126, P97, DOI 10.7326/0003-4819-126-2-199701150-00001

Raveh D, 2005, J EVAL CLIN PRACT, V11, P33, DOI 10.1111/j.1365-2753.2004.00492.x

RUBENSTEIN LZ, 1984, NEW ENGL J MED, V311, P1664, DOI 10.1056/NEJM198412273112604

Sager MA, 1996, ARCH INTERN MED, V156, P645, DOI 10.1001/archinte.156.6.645

SCHROEDER SA, 1986, ANN INTERN MED, V104, P554, DOI 10.7326/0003-4819-104-4-554

SNEDECOR GW, 1980, STATISTICAL METHODS, P206

STEINER JF, 1987, AM J MED, V83, P331, DOI 10.1016/0002-9343(87)90705-4

Thomas DC, 2003, ANN INTERN MED, V139, P628, DOI 10.7326/0003-4819-139-7-200310070-00037

Warshaw GA, 2003, J GEN INTERN MED, V18, P679, DOI 10.1046/j.1525-1497.2003.20906.x

NATL STUDY GRADUATE

NR 22

TC 23

Z9 29

U1 0

U2 3

PU WILEY

PI HOBOKEN

PA 111 RIVER ST, HOBOKEN 07030-5774, NJ USA

SN 1368-5031

EI 1742-1241

J9 INT J CLIN PRACT

JI Int. J. Clin. Pract.

PD FEB

PY 2007

VL 61

IS 2

BP 247

EP 254

DI 10.1111/j.1742-1241.2006.00925.x

PG 8

WC Medicine, General & Internal; Pharmacology & Pharmacy

WE Science Citation Index Expanded (SCI-EXPANDED)

SC General & Internal Medicine; Pharmacology & Pharmacy

GA 131KU

UT WOS:000243868500016

PM 17263711

DA 2024-08-08

ER

PT J

AU Kabelka, L

AF Kabelka, Ladislav

TI Complex decision making in patients with dementia in an internal

medicine department

SO ANNALS OF PALLIATIVE MEDICINE

LA English

DT Article

DE Dementia syndrome; life expectancy; palliative care; internal medicine;

geriatrics; care plan; previously expressed wishes; polymorbidity

AB With the increase of polymorbidity, extending life expectancy and improving treatment options for chronic diseases, the care for dementia is moving into other areas of medicine. The length and quality of life with advanced dementia is directly dependent on the quality of medical and nursing care, early detection and treatment of complications, nutritional support and palliative care plan. Significant is also the support for family carers. The key coordinators of care for patients with dementia are general practitioners (GPs), geriatricians, psychiatrists, and an increasingly important role play internists. Case reports of patients admitted to an internal medicine department. Description of clinical experiences with caring on patients with dementia. In the internal departments of regional hospitals, there is a room for adjustment of the care plan, for comprehensive assessment of the patient and for making crucial decisions regarding nutrition, treatment of chronic diseases, consideration of previously expressed wishes in the context of the patient condition, and potential prognostic indicators. This assessment must result in a comprehensive documentation and communication with patients, and in the case of advanced dementia with their family members. The general internal medicine is very often the first place where the patient has a chance to hear about indication for palliative care. Without the availability of a multidisciplinary assessment, good communication and documentation, it is unrealistic to expect that the hospital would provide comprehensive care for patients with dementia.

C1 [Kabelka, Ladislav] Masaryk Mem Inst Brno, Czech Soc Palliat Med, Brno, Czech Republic.

C3 Masaryk Memorial Cancer Institute

RP Kabelka, L (corresponding author), Masaryk Mem Inst Brno, Czech Soc Palliat Med, Brno, Czech Republic.

EM ladislav.kabelka.mobilni@gmail.com

CR Chai E., 2014, Geriatric Palliative Care

Fick D, 2012, J AM GERIATR SOC, V60, P616, DOI 10.1111/j.1532-5415.2012.03923.x

Kabelka L., 2017, GERIATRICKA PALIATIV

NR 3

TC 2

Z9 2

U1 3

U2 16

PU AME PUBLISHING COMPANY

PI SHATIN

PA FLAT-RM C 16F, KINGS WING PLAZA 1, NO 3 KWAN ST, SHATIN, HONG KONG

00000, PEOPLES R CHINA

SN 2224-5820

EI 2224-5839

J9 ANN PALLIAT MED

JI Ann. Pallliat. Med.

PD OCT

PY 2017

VL 6

IS 4

BP 399

EP 404

DI 10.21037/apm.2017.06.21

PG 6

WC Health Care Sciences & Services

WE Science Citation Index Expanded (SCI-EXPANDED)

SC Health Care Sciences & Services

GA FC5TF

UT WOS:000406903900014

PM 28754053

DA 2024-08-08

ER

PT J

AU Matter, CA

Speice, JA

McCann, R

Mendelson, DA

McCormick, K

Friedman, S

Medina-Walpole, A

Clark, NS

AF Matter, CA

Speice, JA

McCann, R

Mendelson, DA

McCormick, K

Friedman, S

Medina-Walpole, A

Clark, NS

TI Hospital to home: Improving internal medicine residents' understanding

of the needs of older persons after a hospital stay

SO ACADEMIC MEDICINE

LA English

DT Article

AB Physicians-in-training discharge many older patients from the hospital, but few have any knowledge of what happens to the patients they send home, of how discharge plans are applied, or of the difficulties patients and their families face. The authors describe a pilot program, Hospital to Home, at the University of Rochester School of Medicine and Dentistry's internal medicine residency program, which uses home visits as an educational tool in geriatrics training. The program was begun in July 2001, and 23 residents have participated. Home visits expose residents in their first-year geriatrics rotation to the elements and outcomes of discharge planning and create a heightened awareness of the needs of older persons recently discharged from the hospital. The home visits are videotaped, and the residents present a videoconference based on the visits, which are attended by internal medicine residents, family medicine residents, and medical students. The authors describe the three-part Hospital to Home program, three vignettes that highlight learning experiences, and the residents' feedback about the experience and the use of audiovisual recording for education.

C1 Vet Adm Outpatient Clin, Rochester, NY 14620 USA.

Univ Rochester, Sch Med & Dent, Rochester, NY 14627 USA.

C3 University of Rochester

RP Vet Adm Outpatient Clin, 465 Westfall Rd, Rochester, NY 14620 USA.

EM colleen.matter2@med.va.gov

RI Mendelson, Daniel Ari/AFM-8461-2022

OI Mendelson, Daniel Ari/0000-0002-3777-3686

FU NIMH NIH HHS [T32 MH020061] Funding Source: Medline

CR BECKMAN HB, 1994, J GEN INTERN MED, V9, P517, DOI 10.1007/BF02599224

CREDITOR MC, 1993, ANN INTERN MED, V118, P219, DOI 10.7326/0003-4819-118-3-199302010-00011

Leff B, 1998, NEW ENGL J MED, V338, P1467

Mahoney JE, 2000, J GEN INTERN MED, V15, P611, DOI 10.1046/j.1525-1497.2000.06139.x

Naylor MD, 1999, JAMA-J AM MED ASSOC, V281, P613, DOI 10.1001/jama.281.7.613

Sager MA, 1996, ARCH INTERN MED, V156, P645, DOI 10.1001/archinte.156.6.645

Stoltz CM, 2001, ACAD MED, V76, P181, DOI 10.1097/00001888-200102000-00020

NR 7

TC 29

Z9 29

U1 0

U2 3

PU LIPPINCOTT WILLIAMS & WILKINS

PI PHILADELPHIA

PA TWO COMMERCE SQ, 2001 MARKET ST, PHILADELPHIA, PA 19103 USA

SN 1040-2446

EI 1938-808X

J9 ACAD MED

JI Acad. Med.

PD AUG

PY 2003

VL 78

IS 8

BP 793

EP 797

DI 10.1097/00001888-200308000-00009

PG 5

WC Education, Scientific Disciplines; Health Care Sciences & Services

WE Science Citation Index Expanded (SCI-EXPANDED)

SC Education & Educational Research; Health Care Sciences & Services

GA 801BD

UT WOS:000220070500008

PM 12915369

OA Bronze

DA 2024-08-08

ER

PT J

AU Gorski, S

Piotrowicz, K

Rewiuk, K

Halicka, M

Kalwak, W

Rybak, P

Grodzicki, T

AF Gorski, Stanislaw

Piotrowicz, Karolina

Rewiuk, Krzysztof

Halicka, Monika

Kalwak, Weronika

Rybak, Paulina

Grodzicki, Tomasz

TI Nonpharmacological Interventions Targeted at Delirium Risk Factors,

Delivered by Trained Volunteers (Medical and Psychology Students),

Reduced Need for Antipsychotic Medications and the Length of Hospital

Stay in Aged Patients Admitted to an Acute Internal Medicine Ward: Pilot

Study

SO BIOMED RESEARCH INTERNATIONAL

LA English

DT Article

ID ELDER LIFE PROGRAM; HIP FRACTURE; CARE; IMPROVE

AB Purpose. Effectiveness of nonpharmacological multicomponent prevention delivered by trained volunteers (medical and psychology students), targeted at delirium risk factors in geriatric inpatients, was assessed at an internal medicine ward in Poland. Patients and Methods. Participants were recruited to intervention and control groups at the internal medicine ward (inclusion criteria: age >= 75, acute medical condition, basic orientation, and logical contact on admission; exclusion criteria: life expectancy < 24 hours, surgical hospitalization, isolation due to infectious disease, and discharge to other medical wards). Every day trained volunteers delivered a multicomponent standardized intervention targeted at risk factors of in-hospital complications to the intervention group. The control group, selected using a retrospective individual matching strategy (1 : 1 ratio, regarding age, gender, and time of hospitalization), received standard care. Outcome Measures. Hospitalization time, deaths, falls, delirium episodes, and antipsychotic prescriptions were assessed retrospectively from medical documentation. Results. 130 patients (38.4% males) participated in the study, with 65 in the intervention group. Antipsychotic medications were initiated less frequently in the intervention group compared to the control group. There was a trend towards a shorter hospitalization time and a not statistically significant decrease in deaths in the intervention group. Conclusion. Nonpharmacological multicomponent intervention targeted at delirium risk factors effectively reduced length of hospitalization and need for initiating antipsychotic treatment in elderly patients at the internal medicine ward.

C1 [Gorski, Stanislaw] Jagiellonian Univ, Dept Med Educ, Coll Med, Krakow, Poland.

[Piotrowicz, Karolina; Rewiuk, Krzysztof; Grodzicki, Tomasz] Jagiellonian Univ, Dept Internal Med & Gerontol, Coll Med, Krakow, Poland.

[Halicka, Monika; Rybak, Paulina] Jagiellonian Univ, Inst Psychol, Krakow, Poland.

[Kalwak, Weronika] Jagiellonian Univ, Inst Psychol, Dept Hlth Psychol, Krakow, Poland.

C3 Jagiellonian University; Collegium Medicum Jagiellonian University;

Jagiellonian University; Collegium Medicum Jagiellonian University;

Jagiellonian University; Jagiellonian University

RP Gorski, S (corresponding author), Jagiellonian Univ, Dept Med Educ, Coll Med, Krakow, Poland.

EM stanislaw.gorski@uj.edu.pl

RI Piotrowicz, Karolina/AGZ-0543-2022; Grodzicki, Tomasz/K-4451-2012

OI Piotrowicz, Karolina/0000-0002-4760-8588; Rewiuk,

Krzysztof/0000-0001-6451-5730; Halicka, Monika/0000-0001-6283-9352;

Grodzicki, Tomasz/0000-0003-0159-4915

FU Faculty of Medicine of Jagiellonian University Medical College (Leading

National Research Centre)

FX Publication was supported by the Faculty of Medicine of Jagiellonian

University Medical College (Leading National Research Centre 2012-2017).

CR Abraha I, 2015, PLOS ONE, V10, DOI 10.1371/journal.pone.0123090

[Anonymous], 2012, REV CHILENA TERAPIA

Bo Mario, 2009, Am J Geriatr Psychiatry, V17, P760

Buurman BM, 2011, PLOS ONE, V6, DOI 10.1371/journal.pone.0026951

Caplan GA, 2007, INTERN MED J, V37, P95, DOI 10.1111/j.1445-5994.2007.01265.x

CHARLSON M, 1994, J CLIN EPIDEMIOL, V47, P1245, DOI 10.1016/0895-4356(94)90129-5

European Delirium Association, 2014, BMC Med, V12, P141, DOI 10.1186/s12916-014-0141-2

Godfrey M, 2013, BMC HEALTH SERV RES, V13, DOI 10.1186/1472-6963-13-341

Hshieh TT, 2015, JAMA INTERN MED, V175, P512, DOI 10.1001/jamainternmed.2014.7779

Inouye SK, 2014, LANCET, V383, P911, DOI 10.1016/S0140-6736(13)60688-1

Inouye SK, 1999, NEW ENGL J MED, V340, P669, DOI 10.1056/NEJM199903043400901

Inouye SK, 2000, J AM GERIATR SOC, V48, P1697

Klich-Raczka A, 2009, GERONTOLOGIA POLSKA, V17, P32

Leslie DL, 2005, ARCH INTERN MED, V165, P1657, DOI 10.1001/archinte.165.14.1657

Lundström M, 2007, AGING CLIN EXP RES, V19, P178

Marcantonio ER, 2001, J AM GERIATR SOC, V49, P516, DOI 10.1046/j.1532-5415.2001.49108.x

Martinez F, 2015, AGE AGEING, V44, P196, DOI 10.1093/ageing/afu173

Piotrowicz K, 2015, EXP GERONTOL, V72, P45, DOI 10.1016/j.exger.2015.09.007

Pitkala KH, 2005, DEMENT GERIATR COGN, V19, P158, DOI 10.1159/000082888

Rapp C G, 2001, J Gerontol Nurs, V27, P21

Reston JT, 2013, ANN INTERN MED, V158, P375, DOI 10.7326/0003-4819-158-5-201303051-00003

Stenvall M, 2007, OSTEOPOROSIS INT, V18, P167, DOI 10.1007/s00198-006-0226-7

Steunenberg B, 2016, GERIATR NURS, V37, P458, DOI 10.1016/j.gerinurse.2016.06.014

Martinez FT, 2012, AGE AGEING, V41, P629, DOI 10.1093/ageing/afs060

van Zyl LT, 2003, CAN J PSYCHIAT, V48, P555, DOI 10.1177/070674370304800807

Vidán M, 2005, J AM GERIATR SOC, V53, P1476, DOI 10.1111/j.1532-5415.2005.53466.x

Vidán MT, 2009, J AM GERIATR SOC, V57, P2029, DOI 10.1111/j.1532-5415.2009.02485.x

NR 27

TC 27

Z9 27

U1 0

U2 15

PU HINDAWI LTD

PI LONDON

PA ADAM HOUSE, 3RD FLR, 1 FITZROY SQ, LONDON, W1T 5HF, ENGLAND

SN 2314-6133

EI 2314-6141

J9 BIOMED RES INT

JI Biomed Res. Int.

PY 2017

VL 2017

AR 1297164

DI 10.1155/2017/1297164

PG 8

WC Biotechnology & Applied Microbiology; Medicine, Research & Experimental

WE Science Citation Index Expanded (SCI-EXPANDED); Social Science Citation Index (SSCI)

SC Biotechnology & Applied Microbiology; Research & Experimental Medicine

GA EK6IH

UT WOS:000394027800001

PM 28164113

OA Green Published, gold

DA 2024-08-08

ER

PT J

AU BIRO, FM

SIEGEL, DM

PARKER, RM

GILLMAN, MW

AF BIRO, FM

SIEGEL, DM

PARKER, RM

GILLMAN, MW

TI A COMPARISON OF SELF-PERCEIVED CLINICAL COMPETENCES IN PRIMARY-CARE

RESIDENCY GRADUATES

SO PEDIATRIC RESEARCH

LA English

DT Article

ID COMBINED INTERNAL-MEDICINE; FUTURE PRIMARY CARE; TRAINING-PROGRAMS;

STANDARDIZED PATIENTS; FAMILY PHYSICIANS; PEDIATRICS; MERGER;

21ST-CENTURY; SPECIALTY; TIME

AB One hundred seventy-eight graduates from four primary care residency training programs with common hospital sites (medicine)pediatrics, 72; family medicine, 29; pediatrics, 35; and internal medicine, 42) responded to a mailed survey questionnaire regarding distribution of professional time and self-perceived clinical competencies. Most of the internists, family physicians, and internist/pediatricians (MED/PED) were in primary care, and 57% of the pediatricians were subspecialists. Respondents rated each of 24 clinical vignettes as to their level of comfort in managing the patient problems presented. MED/PED and pediatricians responded similarly to all the infant, child, and adolescent cases. Family physicians were less comfortable in managing the complicated neonatal situations but more comfortable with adolescent health care than the MED/PED or pediatricians. MED/PED reported greater comfort than family physicians in complex internal medicine issues, but less than internists in intensive care and geriatric consultation. Significant differences in reported competency existed among these primary care practitioners despite substantially overlapping training backgrounds.

C1 UNIV CINCINNATI,COLL MED,DEPT PEDIAT,CINCINNATI,OH 45229.

UNIV ROCHESTER,SCH MED & DENT,ROCHESTER,NY 14621.

EMORY UNIV,SCH MED,DEPT INTERNAL MED,ATLANTA,GA 30345.

BOSTON UNIV,SCH MED,BOSTON,MA 02130.

C3 University System of Ohio; University of Cincinnati; University of

Rochester; Emory University; Boston University

RI Parker, Ruth/S-4583-2017

FU NIOSH CDC HHS [OH-964] Funding Source: Medline

CR AINSWORTH MA, 1991, JAMA-J AM MED ASSOC, V266, P1390, DOI 10.1001/jama.266.10.1390

BIRO FM, 1990, ACAD MED, V65, P266, DOI 10.1097/00001888-199004000-00010

CHANEY EJ, 1989, J FAM PRACTICE, V29, P600

CHARNEY E, 1975, J MED EDUC, V50, P137

CHRISTIANSEN RG, 1986, JAMA-J AM MED ASSOC, V255, P2628, DOI 10.1001/jama.255.19.2628

COHEN R, 1991, ACAD MED, V66, P545, DOI 10.1097/00001888-199109000-00012

COLWILL JM, 1986, JAMA-J AM MED ASSOC, V255, P2643, DOI 10.1001/jama.255.19.2643

FERRARI ND, 1989, PEDIATRICS, V84, P94

FRANKS P, 1986, MED CARE, V24, P941, DOI 10.1097/00005650-198610000-00007

FRIEDMAN RH, 1986, JAMA-J AM MED ASSOC, V255, P2644, DOI 10.1001/jama.255.19.2644

GEYMAN JP, 1986, JAMA-J AM MED ASSOC, V255, P2631, DOI 10.1001/jama.255.19.2631

GOLDENBERG K, 1989, ACAD MED, V64, P519, DOI 10.1097/00001888-198909000-00009

GREGANTI MA, 1986, J MED EDUC, V61, P883

HAINER BL, 1988, JAMA-J AM MED ASSOC, V260, P354, DOI 10.1001/jama.260.3.354

JOORABCHI B, 1991, AM J DIS CHILD, V145, P757

KARPF M, 1986, ANN INTERN MED, V104, P567, DOI 10.7326/0003-4819-104-4-567

LANGSLEY DG, 1991, JAMA-J AM MED ASSOC, V266, P977, DOI 10.1001/jama.266.7.977

MIDDELKAMP JN, 1989, PEDIATRICS, V84, P181

NORCINI JJ, 1989, JAMA-J AM MED ASSOC, V262, P2402, DOI 10.1001/jama.262.17.2402

PERKOFF GT, 1989, J FAM PRACTICE, V29, P185

PETERSDORF RG, 1975, NEW ENGL J MED, V293, P326, DOI 10.1056/NEJM197508142930704

ROGERS DE, 1985, ANN INTERN MED, V102, P702, DOI 10.7326/0003-4819-102-5-702

SCHERGER JE, 1989, J FAM PRACTICE, V29, P189

SCHROEDER SA, 1986, ANN INTERN MED, V104, P554, DOI 10.7326/0003-4819-104-4-554

SHUMWAY JM, 1987, J GEN INTERN MED, V2, P377, DOI 10.1007/BF02596361

SIEGEL DM, 1988, AM J DIS CHILD, V142, P1104, DOI 10.1001/archpedi.1988.02150100098036

SMILKSTEIN G, 1989, J FAM PRACTICE, V29, P600

STILLMAN P, 1991, ANN INTERN MED, V114, P393, DOI 10.7326/0003-4819-114-5-393

STILLMAN PL, 1987, ARCH INTERN MED, V147, P1049, DOI 10.1001/archinte.147.6.1049

STRELNICK AH, 1988, ANN INTERN MED, V109, P324, DOI 10.7326/0003-4819-109-4-324

TAMBLYN RM, 1991, MED EDUC, V25, P100, DOI 10.1111/j.1365-2923.1991.tb00035.x

WOOLLISCROFT JO, 1984, J MED EDUC, V59, P799

1978, FUTURE PEDIATRIC ED

NR 33

TC 12

Z9 12

U1 0

U2 1

PU WILLIAMS & WILKINS

PI BALTIMORE

PA 351 WEST CAMDEN ST, BALTIMORE, MD 21201-2436

SN 0031-3998

J9 PEDIATR RES

JI Pediatr. Res.

PD NOV

PY 1993

VL 34

IS 5

BP 555

EP 559

DI 10.1203/00006450-199311000-00001

PG 5

WC Pediatrics

WE Science Citation Index Expanded (SCI-EXPANDED)

SC Pediatrics

GA MD795

UT WOS:A1993MD79500001

PM 8284089

OA Bronze

DA 2024-08-08

ER

PT J

AU Meydan, C

Haklai, Z

Gordon, B

Mendlovic, J

Afek, A

AF Meydan, Chanan

Haklai, Ziona

Gordon, Barak

Mendlovic, Joseph

Afek, Arnon

TI Managing the increasing shortage of acute care hospital beds in Israel

SO JOURNAL OF EVALUATION IN CLINICAL PRACTICE

LA English

DT Article

DE health policy; public health

AB Rationale, aims and objectivesIsrael's healthcare system has been facing increasing hospital bed shortage over the last few decades. Community-based services and shortening length of stay have helped to ease this problem, but hospitals continue to suffer from serious overload and saturation. The objective of this study is to present hospitalization trends in Israel's internal medicine departments.

MethodsThe data is based on the National Hospital Discharges database (NHDR) in the Israeli Health Ministry, pertaining to hospitalizations in all internal medicine departments nationwide between 2000 and 2012.

ResultsTotal yearly hospitalization days, representing healthcare burden, had increased by 4.2% during the study period, driven mainly by the most advanced age groups. The rate of total hospitalization days per 100,000 people for all the age groups has decreased by 17.6%, but the oldest patient group had a modest reduction in comparison (7.5%). The parameter of age correlated with length of stay and readmission rates, and neither decreased during the surveyed years.

ConclusionsThese results demonstrated that the healthcare burden on acute internal medicine services has been reduced mostly for middle-aged populations but only modestly for elderly populations. The length of hospital stay and the readmission rates have reached and maintained a plateau in recent years, regardless of age. The findings of this study call for planning specific to elderly populations in light of changing demographics. Possible directions may include renewed emphasis on internal medicine and geriatric medicine, and efforts to shorten hospitalization time by extended utilization of multidisciplinary primary care.

C1 [Meydan, Chanan] Tel Hashomer Med Ctr, Ramat Gan, Israel.

[Haklai, Ziona] Minist Hlth, Hlth Informat Div, Jerusalem, Israel.

[Gordon, Barak] Israeli Def Forces, Med Corps, Ramat Gan, Israel.

[Meydan, Chanan; Gordon, Barak] Tel Aviv Univ, Sackler Fac Med, IL-6997801 Tel Aviv, Israel.

[Mendlovic, Joseph] Minist Hlth, Hlth Adm, Jerusalem, Israel.

[Mendlovic, Joseph] Shaare Zedek Med Ctr, Jerusalem, Israel.

[Afek, Arnon] Minist Hlth, Jerusalem, Israel.

[Afek, Arnon] Tel Aviv Univ, Sackler Fac Med, New York Program, IL-6997801 Tel Aviv, Israel.

C3 Tel Aviv University; Hebrew University of Jerusalem; Shaare Zedek

Medical Center; Tel Aviv University

RP Meydan, C (corresponding author), Tel Aviv Univ, Sackler Fac Med, POB 39040, IL-6997801 Tel Aviv, Israel.

EM chananme@post.tau.ac.il

RI Gordon, Barak/L-6139-2018

OI Meydan, Chanan/0000-0002-8824-6309

CR [Anonymous], 2013, OECD EC SURV ISR 201

[Anonymous], 2012, OECD REV HLTH CAR QU

[Anonymous], PROJ ISR POP UNT 203

[Anonymous], 2013, OECD HLTH STAT 2013

[Anonymous], 2011, BURD OUT OF POCK HLT, DOI DOI 10.1787/HEALTH_GLANCE-2011-54-EN

[Anonymous], ELD ISR 2003 STAT AB

Haklai Z, 2013, HOSPITALIZATION INTE

Health Information Division Israeli Ministry of Health, 2012, INP I DAY CAR UN I 1, P77

Ministry of Health DoHI, 2011, SEL HLTH ALL IND

OECD, 2013, Health at a Glance 2013: OECD Indicators, DOI [DOI 10.1787/HEALTH_GLANCE-2013-EN, 10.1787/eag-2013-en]

Paliel A., 2012, LONG RANGE POPULATIO

Peleg S., 2012, NATL EXPENDITURE HLT

Rosen B, 2011, INT J QUAL HEALTH C, V23, P15, DOI 10.1093/intqhc/mzq065

Shepperd S, 2010, COCHRANE DB SYST REV, DOI 10.1002/14651858.CD000313.pub3

NR 14

TC 11

Z9 11

U1 0

U2 8

PU WILEY-BLACKWELL

PI HOBOKEN

PA 111 RIVER ST, HOBOKEN 07030-5774, NJ USA

SN 1356-1294

EI 1365-2753

J9 J EVAL CLIN PRACT

JI J. Eval. Clin. Pract.

PD FEB

PY 2015

VL 21

IS 1

BP 79

EP 84

DI 10.1111/jep.12246

PG 6

WC Health Care Sciences & Services; Medical Informatics; Medicine, General

& Internal

WE Science Citation Index Expanded (SCI-EXPANDED)

SC Health Care Sciences & Services; Medical Informatics; General & Internal

Medicine

GA CC6AV

UT WOS:000350447800012

PM 25312266

DA 2024-08-08

ER

PT J

AU Coyle, A

Bhatia, S

Arnaldy, AR

Wang, KE

Lindenberger, EC

Fishman, M

AF Coyle, Andrew

Bhatia, Sonica

Reyes Arnaldy, Amy

Wang, Katherine

Lindenberger, Elizabeth C.

Fishman, Mary

TI Advance care planning clinic: A structured clinical experience for

internal medicine residents

SO JOURNAL OF THE AMERICAN GERIATRICS SOCIETY

LA English

DT Article

DE advance care planning; ambulatory education; geriatrics education;

graduate medical education; internal medicine residency education

ID DIRECTIVES; END; COMMUNICATION; COMPLETION

AB Background Advance care planning (ACP) is an important step to provide medical care consistent with patients' preferences and values. Nationally, rates of ACP completion are low, and internal medicine residency clinics face additional barriers. To address this need, we implemented an ACP clinic for internal medicine residents. Methods An ACP clinical experience was created for PGY2 residents beginning in 2018, with 6 total sessions, consisting of consolidated didactics, protected time to identify, outreach, and schedule patients, and two half days of dedicated ACP visits. Residents were surveyed before (end of PGY1) and after (end of PGY2) the intervention. The preceding residency class, serving as a historic control, only received the curriculum and were surveyed at the end of their PGY2 year. Electronic medical record (EMR) data was accessed to track ACP documentation. Results The overall survey response rate was 124/134 (93%). Comparing the intervention cohort before and after the intervention, there was a significant increase in self-assessed confidence in completing ACP (2.1/4.0 vs 3.5/4.0, p < 0.01). Comparing the intervention and historic cohorts (end of PGY2), the intervention was associated with improved confidence in ability to complete ACP for their patients (3.5/4.0 vs 2.7/4.0, p < 0.01). The historic control had no increase in ACP documentation rates over time, while the intervention cohort had a 13.9% absolute increase in ACP documentation for their patients over the course of residency (p < 0.01). Conclusion The creation of an ACP-specific clinical experience, in conjunction with existing curricula, resulted in significant improvements in knowledge, self-assessed skills and behavior, and EMR documentation.

C1 [Coyle, Andrew; Fishman, Mary] Icahn Sch Med Mt Sinai, Dept Med, 17 East 102nd St,7th Floor Area A, New York, NY 10029 USA.

[Bhatia, Sonica; Wang, Katherine; Lindenberger, Elizabeth C.] Icahn Sch Med Mt Sinai, Brookdale Dept Geriatr & Palliat Med, New York, NY USA.

[Reyes Arnaldy, Amy] AdventHlth Med Grp, Tavares, FL USA.

[Lindenberger, Elizabeth C.] James J Peters Vet Affairs Med Ctr, Geriatr Res Educ & Clin Ctr, Bronx, NY USA.

C3 Icahn School of Medicine at Mount Sinai; Icahn School of Medicine at

Mount Sinai; US Department of Veterans Affairs; Veterans Health

Administration (VHA); James J. Peters VA Medical Center; Geriatric

Research Education & Clinical Center

RP Coyle, A (corresponding author), Icahn Sch Med Mt Sinai, Dept Med, 17 East 102nd St,7th Floor Area A, New York, NY 10029 USA.

EM andrew.coyle@mountsinai.org

CR Alderman JS, 2008, AM J HOSP PALLIAT ME, V25, P190, DOI 10.1177/1049909108315301

Alexander SC, 2006, ACAD MED, V81, P1008, DOI 10.1097/01.ACM.0000242580.83851.ad

Allen Sarah Leatherman, 2015, J Grad Med Educ, V7, P91, DOI 10.4300/JGME-D-14-00145.1

Berns SH, 2017, J PALLIAT MED, V20, P1345, DOI 10.1089/jpm.2016.0273

Buss Mary Kathleen, 2005, J Palliat Med, V8, P363, DOI 10.1089/jpm.2005.8.363

Chan D, 2016, J PALLIAT MED, V19, P734, DOI 10.1089/jpm.2015.0313

Chang C, 2018, J AM GERIATR SOC, V66, P420, DOI 10.1111/jgs.15152

Chang C, 2017, GERONTOL GERIATR EDU, V38, P271, DOI 10.1080/02701960.2015.1031897

Colbert Colleen Y, 2010, J Grad Med Educ, V2, P278, DOI 10.4300/JGME-D-10-00003.1

Coyle Andrew, 2020, J Grad Med Educ, V12, P478, DOI 10.4300/JGME-D-19-00909.1

Deep KS, 2007, J PALLIAT MED, V10, P712, DOI 10.1089/jpm.2006.0220

GORDON GH, 1991, ARCH INTERN MED, V151, P567, DOI 10.1001/archinte.151.3.567

Knowles M.S., 1980, MODERN PRACTICE ADUL, V2nd

Nassikas NJ, 2020, AM J HOSP PALLIAT ME, V37, P185, DOI 10.1177/1049909119872757

Rao JK, 2014, AM J PREV MED, V46, P65, DOI 10.1016/j.amepre.2013.09.008

Rhodes RL, 2015, AM J HOSP PALLIAT ME, V32, P262, DOI 10.1177/1049909113517163

Silveira MJ, 2014, J AM GERIATR SOC, V62, P706, DOI 10.1111/jgs.12736

Sulmasy DP, 1996, J GEN INTERN MED, V11, P657, DOI 10.1007/BF02600156

Tung EE, 2014, AM J HOSP PALLIAT ME, V31, P275, DOI 10.1177/1049909113485636

VITALtalk, QUICK GUID

Warshaw GA, 2006, J AM GERIATR SOC, V54, P1603, DOI 10.1111/j.1532-5415.2006.00895.x

Yadav KN, 2017, HEALTH AFFAIR, V36, P1244, DOI 10.1377/hlthaff.2017.0175

Yardley S, 2012, MED TEACH, V34, P161, DOI 10.3109/0142159X.2012.643264

Yuen JK, 2020, J PAIN SYMPTOM MANAG, V59, P1, DOI 10.1016/j.jpainsymman.2019.09.001

NR 24

TC 1

Z9 2

U1 0

U2 1

PU WILEY

PI HOBOKEN

PA 111 RIVER ST, HOBOKEN 07030-5774, NJ USA

SN 0002-8614

EI 1532-5415

J9 J AM GERIATR SOC

JI J. Am. Geriatr. Soc.

PD OCT

PY 2021

VL 69

IS 10

BP 2931

EP 2938

DI 10.1111/jgs.17411

EA AUG 2021

PG 8

WC Geriatrics & Gerontology; Gerontology

WE Science Citation Index Expanded (SCI-EXPANDED); Social Science Citation Index (SSCI)

SC Geriatrics & Gerontology

GA WC7LL

UT WOS:000683419800001

PM 34374990

DA 2024-08-08

ER

PT J

AU Martín-Escalante, MD

Quirós-López, R

Martos-Pérez, F

Olalla-Sierra, J

Rivas-Ruiz, F

Aguilar-García, JA

Jiménez-Puente, A

García-Alegría, J

AF Martin-Escalante, M. D.

Quiros-Lopez, R.

Martos-Perez, F.

Olalla-Sierra, J.

Rivas-Ruiz, F.

Aguilar-Garcia, J. A.

Jimenez-Puente, A.

Garcia-Alegria, J.

TI Validation of the PROFUND index to predict early post-hospital discharge

mortality

SO QJM-AN INTERNATIONAL JOURNAL OF MEDICINE

LA English

DT Article

ID MULTIDIMENSIONAL PROGNOSTIC INDEX; COMPREHENSIVE GERIATRIC ASSESSMENT;

LONG-TERM MORTALITY; INTERNAL-MEDICINE; ELDERLY-PATIENTS; CO-MORBIDITY;

CARE; DEPARTMENTS; FEATURES

AB Background: The PROFUND index (PI) is a prognostic scale for polypathological patients at 12months. The objective of the study was to validate the PI as a predictor of 1-year mortality in a current cohort of polypathological patients and analyse its prognostic usefulness in the short-term (1month and 3months) after discharge from Internal Medicine.

Design: We conducted a prospective observational study and all polypathological patients discharged from an Internal Medicine Department between 01 March 2016 and 28 February 2017 were enrolled.

Methods: The variables recorded for each patient were age, sex, diseases and diagnostic categories defining patients as polypathological patients, PI at discharge, number of hospital admissions, length of stay, vital status at 1 year, and date and place of death if applicable. Follow-up lasted 1 year from the time of enrolment.

Results: Six hundred and ten polypathological patients were enrolled. Mortality was 41% and the patients who died were older, their length of stay was longer and their PI was higher compared with those who survived. The discrimination of the PI for predicting mortality was good, with a C-statistic of 0.718 [95% confidence interval (CI) 0.67-0.76]. In addition, a subgroup of patients with early mortality after discharge was identified, with a C-statistic of 0.74 (95% CI 0.67-0.80) at 30days and 0.73 (95% CI 0.68-0.78) at 90days.

Conclusions: The PI is a valid tool for predicting early and 1-year mortality in polypathological patients after discharge from Internal Medicine.

C1 [Martin-Escalante, M. D.; Quiros-Lopez, R.; Martos-Perez, F.; Olalla-Sierra, J.; Aguilar-Garcia, J. A.; Garcia-Alegria, J.] Hosp Costa del Sol, Internal Med Dept, Malaga, Spain.

[Quiros-Lopez, R.; Rivas-Ruiz, F.; Jimenez-Puente, A.; Garcia-Alegria, J.] Res Network Hlth Serv Chron Dis REDISSEC, Malaga, Spain.

[Rivas-Ruiz, F.] Hosp Costa del Sol, Res Unit, Malaga, Spain.

[Jimenez-Puente, A.] Hosp Costa del Sol, Assessment Unit, Malaga, Spain.

RP Martín-Escalante, MD (corresponding author), Hosp Costa del Sol, Internal Med Dept, Malaga, Spain.

EM mmartinescalante@gmail.com

RI Rivas-Ruiz, Francisco/I-2814-2017; Martos, Francisco/ABB-5430-2021

OI Rivas-Ruiz, Francisco/0000-0002-8894-0501; Martos,

Francisco/0000-0003-3660-2743; Jimenez-Puente,

Alberto/0000-0003-3250-4863; Martin Escalante, Maria

Dolores/0000-0003-1932-8682; Raul, Quiros Lopez/0000-0002-2802-2477

CR [Anonymous], 2011, OECD 50 ANN C

[Anonymous], 2007, PROCESO ASISTENCIAL

Barnett K, 2012, LANCET, V380, P37, DOI 10.1016/S0140-6736(12)60240-2

Bernabeu-Wittel M, 2016, EUR J INTERN MED, V36, P20, DOI 10.1016/j.ejim.2016.07.022

Bernabeu-Wittel M, 2011, ARCH GERONTOL GERIAT, V53, P284, DOI 10.1016/j.archger.2010.12.006

Bernabeu-Wittel M, 2011, EUR J INTERN MED, V22, P311, DOI 10.1016/j.ejim.2010.11.012

Bernabeu-Wittel M, 2013, J AM GERIATR SOC, V61, P475, DOI 10.1111/jgs.12142

CHARLSON ME, 1987, J CHRON DIS, V40, P373, DOI 10.1016/0021-9681(87)90171-8

Díez-Manglano J, 2016, MED CLIN-BARCELONA, V147, P238, DOI 10.1016/j.medcli.2016.06.003

Díez-Manglano J, 2015, INTERN EMERG MED, V10, P915, DOI 10.1007/s11739-015-1252-2

García-Morillo JS, 2005, MED CLIN-BARCELONA, V125, P5, DOI 10.1157/13076399

Krumholz HM, 2013, NEW ENGL J MED, V368, P100, DOI 10.1056/NEJMp1212324

LINN BS, 1968, J AM GERIATR SOC, V16, P622, DOI 10.1111/j.1532-5415.1968.tb02103.x

López-Garrido MA, 2017, REV CLIN ESP, V217, P87, DOI 10.1016/j.rce.2016.10.007

Miró O, 2009, REV ESP CARDIOL, V62, P757, DOI 10.1016/S0300-8932(09)71689-6

Pasina L, 2018, ARCH GERONTOL GERIAT, V74, P169, DOI 10.1016/j.archger.2017.10.016

Pilotto A, 2008, REJUV RES, V11, P151, DOI 10.1089/rej.2007.0569

Pilotto A, 2009, J ALZHEIMERS DIS, V18, P191, DOI 10.3233/JAD-2009-1139

Pilotto A, 2009, J GERONTOL A-BIOL, V64, P880, DOI 10.1093/gerona/glp031

Ramírez-Duque N, 2008, REV CLIN ESP, V208, P4, DOI 10.1157/13115000

Suárez-Dono J, 2016, EUR J INTERN MED, V36, P25, DOI 10.1016/j.ejim.2016.08.002

Zafrir B, 2010, ISR MED ASSOC J, V12, P10

Gaviria AZ, 2016, REV CLIN ESP, V216, P175, DOI 10.1016/j.rce.2016.01.002

Zekry D, 2010, REJUV RES, V13, P675, DOI 10.1089/rej.2010.1037

NR 24

TC 2

Z9 2

U1 0

U2 0

PU OXFORD UNIV PRESS

PI OXFORD

PA GREAT CLARENDON ST, OXFORD OX2 6DP, ENGLAND

SN 1460-2725

EI 1460-2393

J9 QJM-INT J MED

JI QJM-An Int. J. Med.

PD NOV

PY 2019

VL 112

IS 11

BP 854

EP 860

DI 10.1093/qjmed/hcz179

PG 7

WC Medicine, General & Internal

WE Science Citation Index Expanded (SCI-EXPANDED)

SC General & Internal Medicine

GA JP3LH

UT WOS:000498168500006

PM 31297526

OA Bronze

DA 2024-08-08

ER

PT J

AU Ross, K

Lynn, L

Foley, KT

Barczi, SR

Widera, E

Parks, S

Luz, C

Colburn, JL

Leff, B

AF Ross, Kathryn

Lynn, Lorna

Foley, Kevin T.

Barczi, Steven R.

Widera, Eric

Parks, Susan

Luz, Clare

Colburn, Jessica L.

Leff, Bruce

TI Fellowship-trained physicians who let their geriatric medicine

certification lapse: A national survey

SO JOURNAL OF THE AMERICAN GERIATRICS SOCIETY

LA English

DT Article

DE board certification; geriatric medicine; maintenance of certification;

workforce development

AB BackgroundOnly 62.6% of fellowship-trained and American Board of Internal Medicine (ABIM)-certified geriatricians maintain their specialty certification in geriatric medicine, the lowest rate among all internal medicine subspecialties and the only subspecialty in which physicians maintain their internal medicine certification at higher rates than their specialty certification. This study aims to better understand underlying issues related to the low rate of maintaining geriatric medicine certification in order to inform geriatric workforce development strategies.MethodsEighteen-item online survey of internists who completed a geriatric medicine fellowship, earned initial ABIM certification in geriatric medicine between 1999 and 2009, and maintained certification in internal medicine (and/or another specialty but not geriatric medicine). Survey domains: demographics, issues related to maintaining geriatric medicine certification, professional identity, and current professional duties.Results153/723 eligible completed surveys (21.5% response). Top reasons for not maintaining geriatric medicine certification were time (56%), cost of maintenance of certification (MOC) (45%), low Medicare reimbursement for geriatricians' work (32%), and no employer requirement to maintain geriatric medicine certification (31%). Though not maintaining geriatric medicine certification, 68% reported engaging in professional activities related to geriatric medicine. Reflecting on career decisions, 56% would again complete geriatric medicine fellowship, 21% would not, and 23% were unsure. 54% considered recertifying in geriatric medicine. 49% reported flexible MOC assessment options would increase likelihood of maintaining certification.ConclusionsThe value proposition of geriatric medicine certification needs strengthening. Geriatric medicine leaders must develop strategies and tactics to reduce attrition of geriatricians by enhancing the value of geriatric medicine expertise to key stakeholders.

See related Commentary by

C1 [Ross, Kathryn; Lynn, Lorna; Leff, Bruce] Amer Board Internal Med, 510 Walnut St,Suite 1700, Philadelphia, PA 19106 USA.

[Foley, Kevin T.; Luz, Clare] Michigan State Univ, Coll Osteopath Med, Dept Family & Community Med, E Lansing, MI USA.

[Barczi, Steven R.] Univ Wisconsin, Div Geriatr, Madison, WI USA.

[Barczi, Steven R.] Wm S Middleton Vet Affairs, Div Geriatr & Gerontol, Geriatr Res Educ & Clin Ctr, Madison, WI USA.

[Widera, Eric] Univ Calif San Francisco, Div Geriatr, San Francisco, CA USA.

[Parks, Susan] Thomas Jefferson Univ, Div Geriatr Med, Philadelphia, PA USA.

[Colburn, Jessica L.; Leff, Bruce] Johns Hopkins Univ, Div Geriatr Med, Sch Med, Baltimore, MD USA.

C3 American Board of Internal Medicine; Michigan State University; Michigan

State University College of Osteopathic Medicine; University of

Wisconsin System; University of Wisconsin Madison; Geriatric Research

Education & Clinical Center; University of California System; University

of California San Francisco; Jefferson University; Johns Hopkins

University

RP Ross, K (corresponding author), Amer Board Internal Med, 510 Walnut St,Suite 1700, Philadelphia, PA 19106 USA.

EM kross@abim.org

OI Widera, Eric/0000-0003-2296-2852

CR Administration HRS, 2017, NATL REGIONAL PROJEC

American Board of Internal Medicine, CENTR DAT REP

American Board of Internal Medicine, MAINT CERT REQ

[Anonymous], 2008, Committee on the Future Health Care Workforce for Older Americans. Retooling for an Aging America: Building the Health Care Workforce

Gurwitz JH, 2023, JAMA-J AM MED ASSOC, V330, P693, DOI 10.1001/jama.2023.11110

Hirth VA, 2008, AM J MED, V121, P247, DOI 10.1016/j.amjmed.2007.10.030

ihi, AG FRIENDL HLTH SYST

Snyder RA., HLTH AFFAIRS 0414

Tinetti M, 2016, J AM GERIATR SOC, V64, P1400, DOI 10.1111/jgs.14181

NR 9

TC 2

Z9 2

U1 0

U2 0

PU WILEY

PI HOBOKEN

PA 111 RIVER ST, HOBOKEN 07030-5774, NJ USA

SN 0002-8614

EI 1532-5415

J9 J AM GERIATR SOC

JI J. Am. Geriatr. Soc.

PD APR

PY 2024

VL 72

IS 4

BP 1177

EP 1182

DI 10.1111/jgs.18781

EA JAN 2024

PG 6

WC Geriatrics & Gerontology; Gerontology

WE Science Citation Index Expanded (SCI-EXPANDED); Social Science Citation Index (SSCI)

SC Geriatrics & Gerontology

GA NS2I7

UT WOS:001144990100001

PM 38243369

DA 2024-08-08

ER

PT J

AU Demir, MV

Tamer, A

Cinemre, H

Uslan, I

Yaylaci, S

Erkorkmaz, U

AF Demir, M. V.

Tamer, A.

Cinemre, H.

Uslan, I.

Yaylaci, S.

Erkorkmaz, U.

TI Nutritional status and laboratory parameters among internal medicine

inpatients

SO NIGERIAN JOURNAL OF CLINICAL PRACTICE

LA English

DT Article

DE Internal medicine inpatients; malnutrition related laboratory

parameters; nutritional status

ID MALNUTRITION; PREVALENCE; MORTALITY; HISTORY; RISK

AB Background: Malnutrition is a clinical state resulting in prolonged hospital stay, increase in severity of infections and poor wound healing.

Aims: Our aim was to investigate the prevalence and etiologic factors of malnutrition in medical inpatients.

Study Design: A total of 290 consecutively admitted internal medicine patients from February to May 2012 were included. On admission, demographic data, anthropometric measurements, laboratory parameters and nutritional screening test results were recorded.

Methods: Nutritional risk score-2002 for patients under 65 years old, mini nutritional assessment for older patients and subjective global assessment (SGA) tests performed. Relation of demographic characteristics, laboratory parameters, weight and body mass index (BMI) with nutritional status were evaluated.

Results: Mean age was 61 +/- 17 years; 145 patients were male. Among 160 patients < 65 years old, 34 were in malnutrition (21%), 41 (26%) were under risk of malnutrition and 85 (53%) were normal. When they were divided into three groups according to SGA, we found significant difference in hemoglobin, low density lipoprotein (LDL), high density lipoprotein, cholesterol, triglyceride, albumin and protein, weight and BMI. Among 130 patients over 65 years old, 47 patients (37%) were in malnutrition, 41 (31%) were under risk of malnutrition and 42 (32%) were normal. There was significant difference in LDL, cholesterol, albumin, protein, weight and BMI between three groups; each 1 g/dl decrease in serum albumin and age older than 65 years old increased malnutrition risk 5.21 and 1.97 times, respectively.

Conclusion: Malnutrition risk is high among internal medicine inpatients and risk seems to be higher among older patients. Nutritional screening of geriatric patients, close follow-up and providing earlier health care would contribute rehabilitation of chronic diseases and decrease re-admissions.

C1 [Demir, M. V.] Malatya State Hosp, Dept Internal Med, TR-44100 Malatya, Turkey.

[Tamer, A.; Cinemre, H.; Uslan, I.] Sakarya Univ, Dept Internal Med, Fac Med, Sakarya, Turkey.

[Erkorkmaz, U.] Sakarya Univ, Dept Biostat, Fac Med, Sakarya, Turkey.

[Yaylaci, S.] Rize Findikli State Hosp, Dept Internal Med, Rize, Turkey.

C3 Malatya State Hospital; Sakarya University; Sakarya University; Rize

Findikli Region Goiter Research & Treatment Center

RP Demir, MV (corresponding author), Malatya State Hosp, Dept Internal Med, TR-44100 Malatya, Turkey.

EM mvolkandemir@gmail.com

RI uslan, ihsan/ABC-2530-2020; TAMER, ALİ/HTO-2299-2023; Erkorkmaz,

Ünal/AAA-4241-2022; Yaylacı, Selcuk/ABF-7883-2022; Cinemre,

Hakan/AAD-7506-2021

OI uslan, ihsan/0000-0002-8890-8026; TAMER, ALİ/0000-0003-2005-0737;

Erkorkmaz, Ünal/0000-0002-8497-4704; Yaylacı,

Selcuk/0000-0002-6768-7973;

CR [Anonymous], 2005, J AM DIET ASSOC, V105, P1955, DOI 10.1016/j.jada.2005.10.004

Barone L, 2003, J Nutr Health Aging, V7, P13

Bauer JM, 2008, NUTR CLIN PRACT, V23, P388, DOI 10.1177/0884533608321132

BISTRIAN BR, 1976, JAMA-J AM MED ASSOC, V235, P1567, DOI 10.1001/jama.235.15.1567

Covinsky KE, 1999, J AM GERIATR SOC, V47, P532, DOI 10.1111/j.1532-5415.1999.tb02566.x

Crogan NL, 2003, J GERONTOL A-BIOL, V58, P159

DETSKY AS, 1987, JPEN-PARENTER ENTER, V11, P8, DOI 10.1177/014860718701100108

Guigoz Y, 2002, CLIN GERIATR MED, V18, P737, DOI 10.1016/S0749-0690(02)00059-9

KNAUS WA, 1991, CHEST, V100, P1619, DOI 10.1378/chest.100.6.1619

Kondrup J, 2003, CLIN NUTR, V22, P415, DOI 10.1016/S0261-5614(03)00098-0

Korfali G, 2009, CLIN NUTR, V28, P533, DOI 10.1016/j.clnu.2009.04.015

Lamb CA, 2009, BRIT J NUTR, V102, P571, DOI 10.1017/S0007114509236038

Landi F, 2000, ARCH INTERN MED, V160, P2641, DOI 10.1001/archinte.160.17.2641

Mitrache C, 2001, ANN HEMATOL, V80, P295, DOI 10.1007/s002770100287

Nursal TZ, 2005, NUTRITION, V21, P659, DOI 10.1016/j.nut.2004.10.016

Olmos MMN, 2005, EUR J CLIN NUTR, V59, P938, DOI 10.1038/sj.ejcn.1602157

Omran ML, 2000, NUTRITION, V16, P50, DOI 10.1016/S0899-9007(99)00224-5

PAYETTE H, 1990, AM J CLIN NUTR, V52, P927, DOI 10.1093/ajcn/52.5.927

RAYNAUDSIMON A, 2009, EUROPEAN E J CLIN NU, V4, pE86

ROTHSCHILD MA, 1972, NEW ENGL J MED, V286, P748, DOI 10.1056/NEJM197204062861404

Segall L, 2008, Rev Med Chir Soc Med Nat Iasi, V112, P343

Seiler WO, 2001, NUTRITION, V17, P496, DOI 10.1016/S0899-9007(01)00558-5

Sullivan DH, 2001, J GERONTOL A-BIOL, V56, pM71, DOI 10.1093/gerona/56.2.M71

Sungurtekin H, 2003, TURK ANESTEZIYOLOJI, V31, P368

NR 24

TC 9

Z9 10

U1 0

U2 11

PU MEDKNOW PUBLICATIONS & MEDIA PVT LTD

PI MUMBAI

PA B-9, KANARA BUSINESS CENTRE, OFF LINK RD, GHAKTOPAR-E, MUMBAI, 400075,

INDIA

SN 1119-3077

J9 NIGER J CLIN PRACT

JI Niger. J. Clin. Pract.

PD NOV-DEC

PY 2015

VL 18

IS 6

BP 757

EP 761

DI 10.4103/1119-3077.158145

PG 5

WC Medicine, General & Internal

WE Science Citation Index Expanded (SCI-EXPANDED)

SC General & Internal Medicine

GA DM2GF

UT WOS:000376164400009

PM 26289513

DA 2024-08-08

ER

PT J

AU Retornaz, F

Seux, V

Sourial, N

Braud, AC

Monette, J

Bergman, H

Soubeyrand, J

AF Retornaz, Frederique

Seux, Valerie

Sourial, Nadia

Braud, Anne-Chantal

Monette, Johanne

Bergman, Howard

Soubeyrand, Jacques

TI Comparison of the health and functional status between older inpatients

with and without cancer admitted to a geriatric/internal medicine unit

SO JOURNALS OF GERONTOLOGY SERIES A-BIOLOGICAL SCIENCES AND MEDICAL

SCIENCES

LA English

DT Article

ID ELDERLY-PATIENTS; INSTRUMENTAL ACTIVITIES; PERFORMANCE STATUS; ONCOLOGY;

MORTALITY; CHEMOTHERAPY; COMORBIDITY; PREVALENCE; DISABILITY; DEPRESSION

AB Background. Cancer is predominantly a disease in the population aged 65 years and older. Previous studies have suggested that older cancers patients seen in oncology departments are healthy with few comorbidities. Relatively little is known about the health and functional status of older cancer inpatients, especially outside oncology units. The purpose of this study is to compare the health and functional status of older cancer and noncancer inpatients admitted to a geriatric/ internal medicine unit.

Methods. A retrospective chart review was conducted on inpatients 65 years old and older, who had been hospitalized during a period of 2 years in the geriatric/internal medicine unit. The health and functional status of 144 inpatients with active cancer was compared to that of 682 inpatients without active cancer. Eight domains were compared: functional status, comorbidity, medication, nutritional status, neurosensory deficits, cognition, mood, and mobility. The hospitalization measures (length of stay, death, need for palliative care) were also compared.

Results. We found that inpatients with active cancer were younger, had less comorbidity and less cognitive impairment, but were more depressed and at greater risk for malnutrition than patients without cancer. These two groups were similar in terms of functional status, neurosensory deficit, and mobility. Cancer patients had a significantly shorter length of stay, required more palliative care, and were more likely to die during hospitalization.

Conclusion. These findings indicate that older cancer patients admitted to a geriatric/internal medicine unit present with multiple active geriatric problems, have characteristics distinct from those of traditional geriatric patients, and require specific care and management.

C1 [Retornaz, Frederique; Seux, Valerie; Sourial, Nadia; Braud, Anne-Chantal; Monette, Johanne; Bergman, Howard; Soubeyrand, Jacques] Ctr Hosp Univ St Marguerite, Dept Internal Med & Geriatr, Marseille, France.

C3 Aix-Marseille Universite

RP Retornaz, F (corresponding author), McGill Univ, Jewish Gen Hosp, Div Geriatr Med, 3755 Cote St Catherine, Montreal, PQ H3T 1E2, Canada.

EM frederique.retomaz@mail.mcgill.ca

CR Argiles J M, 2005, Eur J Oncol Nurs, V9 Suppl 2, pS39, DOI 10.1016/j.ejon.2005.09.006

Balducci L, 2000, CRIT REV ONCOL HEMAT, V35, P147, DOI 10.1016/S1040-8428(00)00089-5

BARBERGERGATEAU P, 1993, AGE AGEING, V22, P457, DOI 10.1093/ageing/22.6.457

BENJAMINI Y, 1995, J R STAT SOC B, V57, P289, DOI 10.1111/j.2517-6161.1995.tb02031.x

Cacciatore F, 2004, AGING CLIN EXP RES, V16, P382

Clement JP, 1997, ENCEPHALE, V23, P91

Covinsky KE, 1999, ANN INTERN MED, V130, P563, DOI 10.7326/0003-4819-130-7-199904060-00004

Donini L M, 2003, J Nutr Health Aging, V7, P282

Extermann M, 2005, CRIT REV ONCOL HEMAT, V55, P241, DOI 10.1016/j.critrevonc.2005.06.003

Extermann M, 2004, CRIT REV ONCOL HEMAT, V49, P69, DOI 10.1016/S1040-8428(03)00099-4

Extermann M, 2002, EUR J CANCER, V38, P1466, DOI 10.1016/S0959-8049(02)00090-4

Ferrucci L, 2003, CRIT REV ONCOL HEMAT, V46, P127, DOI 10.1016/S1040-8428(02)00177-4

Flood KL, 2006, J CLIN ONCOL, V24, P2298, DOI 10.1200/JCO.2005.02.8514

FOLSTEIN MF, 1975, J PSYCHIAT RES, V12, P189, DOI 10.1016/0022-3956(75)90026-6

Frazier Susan C, 2005, J Gerontol Nurs, V31, P4

Garman KS, 2004, CRIT REV ONCOL HEMAT, V51, P241, DOI 10.1016/j.critrevonc.2004.03.002

Given B, 2001, NURS RES, V50, P222, DOI 10.1097/00006199-200107000-00006

Ingram SS, 2002, J CLIN ONCOL, V20, P770, DOI 10.1200/JCO.20.3.770

Inouye SK, 1998, JAMA-J AM MED ASSOC, V279, P1187, DOI 10.1001/jama.279.15.1187

KATZ S, 1983, J AM GERIATR SOC, V31, P721, DOI 10.1111/j.1532-5415.1983.tb03391.x

Lunney JR, 2003, JAMA-J AM MED ASSOC, V289, P2387, DOI 10.1001/jama.289.18.2387

Maione P, 2005, J CLIN ONCOL, V23, P6865, DOI 10.1200/JCO.2005.02.527

Passik SD, 1998, J CLIN ONCOL, V16, P1594, DOI 10.1200/JCO.1998.16.4.1594

Repetto L, 1998, CANCER, V82, P760, DOI 10.1002/(SICI)1097-0142(19980215)82:4<760::AID-CNCR20>3.0.CO;2-V

Repetto L, 2002, J CLIN ONCOL, V20, P494, DOI 10.1200/JCO.2002.20.2.494

Reuben DB, 1998, J AM GERIATR SOC, V46, P1008, DOI 10.1111/j.1532-5415.1998.tb02758.x

Roche R J, 1997, Cancer Pract, V5, P81

Rubenstein LZ, 2001, J GERONTOL A-BIOL, V56, pM366, DOI 10.1093/gerona/56.6.M366

Serraino D, 2001, CRIT REV ONCOL HEMAT, V39, P269, DOI 10.1016/S1040-8428(00)00130-X

SILVERMAN M, 1995, J AM GERIATR SOC, V43, P733, DOI 10.1111/j.1532-5415.1995.tb07041.x

Solomon D, 2003, J AM GERIATR SOC, V51, P1490, DOI 10.1046/j.1532-5415.2003.51471.x

STUCK AE, 1993, LANCET, V342, P1032, DOI 10.1016/0140-6736(93)92884-V

Teno J M, 2001, J Palliat Med, V4, P457, DOI 10.1089/109662101753381593

Vellas BJ, 1997, J AM GERIATR SOC, V45, P735, DOI 10.1111/j.1532-5415.1997.tb01479.x

Wolfson C, 2001, NEW ENGL J MED, V344, P1111, DOI 10.1056/NEJM200104123441501

Yancik R, 2000, HEMATOL ONCOL CLIN N, V14, P17, DOI 10.1016/S0889-8588(05)70275-6

Yancik R, 2001, JAMA-J AM MED ASSOC, V285, P885, DOI 10.1001/jama.285.7.885

NR 37

TC 31

Z9 33

U1 0

U2 3

PU OXFORD UNIV PRESS INC

PI CARY

PA JOURNALS DEPT, 2001 EVANS RD, CARY, NC 27513 USA

SN 1079-5006

EI 1758-535X

J9 J GERONTOL A-BIOL

JI J. Gerontol. Ser. A-Biol. Sci. Med. Sci.

PD AUG

PY 2007

VL 62

IS 8

BP 917

EP 922

DI 10.1093/gerona/62.8.917

PG 6

WC Geriatrics & Gerontology; Gerontology

WE Science Citation Index Expanded (SCI-EXPANDED); Social Science Citation Index (SSCI)

SC Geriatrics & Gerontology

GA 272AD

UT WOS:000253828900016

PM 17702885

OA Bronze

DA 2024-08-08

ER

PT J

AU Reid, MC

Tinetti, ME

Brown, CJ

Concato, J

AF Reid, MC

Tinetti, ME

Brown, CJ

Concato, J

TI Physician awareness of alcohol use disorders among older patients

SO JOURNAL OF GENERAL INTERNAL MEDICINE

LA English

DT Article

DE alcohol use disorders; older patients; screening

ID PRIMARY-CARE PATIENTS; SCREENING QUESTIONNAIRES; CAGE QUESTIONNAIRE;

ELDERLY VETERANS; DRINKING; ABUSE; ADMISSION; DRINKERS; PEOPLE

AB OBJECTIVES: To determine primary care physicians' awareness of, and screening practices for, alcohol use disorders (AUDs) among older patients.

DESIGN: Cross-sectional telephone survey of a national sample of primary care physicians.

PARTICIPANTS: Physicians randomly sampled from the Masterfile database of the American Medical Association and stratified by specialty as family practice physicians, internal medicine physicians, and either family practice or internal medicine physicians with geriatric certification.

MAIN RESULTS: A total of 171 physicians were contacted: 155 (91%) agreed to participate, and responses were analyzed from 150 (50 family practice, 50 internal medicine, 50 with geriatric certification). The median prevalence estimate of AUDs among older patients was 5% for each group of physicians. In contrast to published prevalence rates of AUDs ranging from 5% to 23%, 38% of physicians reported prevalence estimates of less than 5%, and 5% cited estimates of at least 25%. Compared with the other groups, the physicians with geriatric certification were more likely to report no regular screening (42% vs 20% for family practice vs 18% for internal medicine, p = .01), while younger (<40 years) and middle-aged physicians (40-55 years) reported higher annual screening rates relative to older physicians (>55 years) (77% vs 60% vs 44% respectively, p = .03). Among physicians who regularly screened (n = 110), 100% asked quantity-frequency questions, 39% also used the CAGE questions, and 15% also cited use of biochemical markers.

CONCLUSIONS: Primary care physicians may "underdetect" AUDs among older patients. The development of age-specific screening methods and physician education may facilitate detection of older patients with (or at risk for) these disorders.

C1 W Haven VA Med Ctr, Clin Epidemiol Unit, VA Connecticut Healthcare Syst, West Haven, CT 06516 USA.

C3 US Department of Veterans Affairs; Veterans Health Administration (VHA);

VA Connecticut Healthcare System

RP W Haven VA Med Ctr, Clin Epidemiol Unit, VA Connecticut Healthcare Syst, 111 GIM,950 Campbell Ave, West Haven, CT 06516 USA.

RI Rao, Rahul/P-7158-2014

OI Reid, Cary/0000-0001-8117-662X; Brown, Cynthia J/0000-0002-1629-5507

CR ADAMS WL, 1995, J AM GERIATR SOC, V43, P1021, DOI 10.1111/j.1532-5415.1995.tb05567.x

Adams WL, 1996, JAMA-J AM MED ASSOC, V276, P1964

ADAMS WL, 1992, J AM GERIATR SOC, V40, P1236, DOI 10.1111/j.1532-5415.1992.tb03649.x

[Anonymous], 1996, Guide to Clinical Preventive Services, V2nd

[Anonymous], 1990, Broadening the Base of Treatment for Alcohol Problems, DOI DOI 10.17226/1341

[Anonymous], NIH PUBLICATION

BERNADT MW, 1982, LANCET, V1, P325

BOOTH BM, 1992, ALCOHOL CLIN EXP RES, V16, P1029, DOI 10.1111/j.1530-0277.1992.tb00694.x

BUCHSBAUM DG, 1992, J AM GERIATR SOC, V40, P662, DOI 10.1111/j.1532-5415.1992.tb01956.x

CALLAHAN CM, 1995, J AM GERIATR SOC, V43, P1378, DOI 10.1111/j.1532-5415.1995.tb06617.x

CURTIS JR, 1989, J AM GERIATR SOC, V37, P310, DOI 10.1111/j.1532-5415.1989.tb05496.x

EWING JA, 1984, JAMA-J AM MED ASSOC, V252, P1905, DOI 10.1001/jama.252.14.1905

Fleming MF, 1997, JAMA-J AM MED ASSOC, V277, P1039, DOI 10.1001/jama.277.13.1039

FULOP G, 1993, J AM GERIATR SOC, V41, P737, DOI 10.1111/j.1532-5415.1993.tb07463.x

GRAHAM K, 1986, J STUD ALCOHOL, V47, P322, DOI 10.15288/jsa.1986.47.322

*I HLTH POL, 1993, SUBST AB NAT NUMB 1

JONES TV, 1993, J GEN INTERN MED, V8, P674, DOI 10.1007/BF02598284

Macneil P. D., 1994, Journal of the American Geriatrics Society, V42, pSA7

MAGRUDERHABIB K, 1986, HOSP COMMUNITY PSYCH, V37, P1251

MANGION DM, 1992, AGE AGEING, V21, P362, DOI 10.1093/ageing/21.5.362

MIHAS AA, 1992, AM J MED SCI, V303, P415, DOI 10.1097/00000441-199206000-00014

Mittal VA, 2011, PSYCHIAT RES, V189, P158, DOI 10.1016/j.psychres.2011.06.006

MORAN MB, 1990, J GEN INTERN MED, V5, P361, DOI 10.1007/BF02600408

Morton JL, 1996, AM J MED, V101, P153, DOI 10.1016/S0002-9343(96)80069-6

NAIK PC, 1994, BRIT MED J, V308, P248, DOI 10.1136/bmj.308.6923.248

SANWAN N, 1997, J AM GERIATR SOC, V45, pS5

SELZER ML, 1972, AM J PSYCHIAT, P1653

SIMON A, 1968, GERIATRICS, V23, P125

Wilk AI, 1997, J GEN INTERN MED, V12, P274

World Health Organization, 1992, The ICD10 classification of mental and behavioural disorders: Clinical descriptions and diagnostic guidelines

NR 30

TC 35

Z9 35

U1 0

U2 3

PU SPRINGER

PI NEW YORK

PA ONE NEW YORK PLAZA, SUITE 4600, NEW YORK, NY, UNITED STATES

SN 0884-8734

EI 1525-1497

J9 J GEN INTERN MED

JI J. Gen. Intern. Med.

PD NOV

PY 1998

VL 13

IS 11

BP 729

EP 734

DI 10.1046/j.1525-1497.1998.00223.x

PG 6

WC Health Care Sciences & Services; Medicine, General & Internal

WE Science Citation Index Expanded (SCI-EXPANDED); Social Science Citation Index (SSCI)

SC Health Care Sciences & Services; General & Internal Medicine

GA 137QM

UT WOS:000076927000001

PM 9824517

OA Green Published

DA 2024-08-08

ER

PT J

AU Gogol, M

AF Gogol, M.

TI Choosing Wisely

SO ZEITSCHRIFT FUR GERONTOLOGIE UND GERIATRIE

LA English

DT Article

DE American Board of Internal Medicine (ABIM); Germany; Geriatrics; Health

care system; Cost

ID AMERICAN GERIATRICS SOCIETY; HEALTH-CARE; UNEXPLAINED VARIATION;

DECISION-MAKING; NURSES ROLE; PHYSICIANS; RESIDENTS; COSTS;

DISSEMINATION; MEDICATIONS

AB In 2011 the American Board of Internal Medicine (ABIM) started the Choosing Wisely campaign.

The goal was to establish top 5 lists by the medical societies to reduce diagnostic and therapeutic procedures which are not necessary or are potentially harmful, and thereby lower health care costs. The lists contributed by the American Geriatric Society and the American Medical Director Association in 2013 will be discussed.

At first glance, the idea seems simple, but numerous questions remain. Transferring this process to Germany appears theoretically possible, but various aspects of the health care system should be taken into consideration.

C1 Krankenhaus Lindenbrunn, Klin Geriatrie, D-31863 Coppenbrugge, Germany.

RP Gogol, M (corresponding author), Krankenhaus Lindenbrunn, Klin Geriatrie, Lindenbrunn 1, D-31863 Coppenbrugge, Germany.

EM gogol@krankenhaus-lindenbrunn.de

CR Boyd CM, 2005, JAMA-J AM MED ASSOC, V294, P716, DOI 10.1001/jama.294.6.716

Briesacher BA, 2013, JAMA-J AM MED ASSOC, V309, P440, DOI 10.1001/jama.2012.211266

Brody H, 2012, NEW ENGL J MED, V366, P1949, DOI 10.1056/NEJMp1203365

Brook RH, 2011, JAMA-J AM MED ASSOC, V306, P650, DOI 10.1001/jama.2011.1136

Cassel CK, 2012, JAMA-J AM MED ASSOC, V307, P1801, DOI 10.1001/jama.2012.476

Chen Y, 2010, ARCH INTERN MED, V170, P89, DOI 10.1001/archinternmed.2009.469

Dorsey ER, 2013, NEUROLOGY, V81, P946, DOI 10.1212/WNL.0b013e3182905037

Fick DM, 2013, J GERONTOL NURS, V39, P4, DOI 10.3928/00989134-20130417-01

Fowler FJ, 2013, JAMA INTERN MED, V173, P1215, DOI 10.1001/jamainternmed.2013.6172

Frist WH, 2005, NEW ENGL J MED, V352, P267, DOI 10.1056/NEJMsa045011

Fuso L, 2011, TUMORI J, V97, P551, DOI 10.1700/989.10710

Ickowicz E, 2012, J AM GERIATR SOC, V60, P1957, DOI 10.1111/j.1532-5415.2012.04187.x

Kitzman DW, 2010, JAMA-J AM MED ASSOC, V304, P1950, DOI 10.1001/jama.2010.1592

Lee YY, 2013, MED J AUSTRALIA, V199, P348, DOI 10.5694/mja13.10279

Lemay CA, 2013, J AM MED DIR ASSOC, V14, P895, DOI 10.1016/j.jamda.2013.08.009

Lipkin M, 2013, JAMA INTERN MED, V173, P1204, DOI 10.1001/jamainternmed.2013.6248

Lopez RP, 2010, ARCH INTERN MED, V170, P83, DOI 10.1001/archinternmed.2009.467

Maguire A, 2013, J AM GERIATR SOC, V61, P215, DOI 10.1111/jgs.12101

Moriates C, 2013, JAMA INTERN MED, V173, P308, DOI 10.1001/jamainternmed.2013.2286

OECD, 2013, Health at a Glance 2013: OECD Indicators, DOI [DOI 10.1787/HEALTH_GLANCE-2013-EN, 10.1787/eag-2013-en]

Resnick B, 2013, GERIATR NURS, V34, P179, DOI 10.1016/j.gerinurse.2013.04.009

Rosenbaum L, 2013, NEW ENGL J MED, V368, P959, DOI 10.1056/NEJMms1301576

Rosenbaum L, 2012, NEW ENGL J MED, V367, P99, DOI 10.1056/NEJMp1205634

Samuel MJ, 2013, J AM GERIATR SOC, V61, P622, DOI 10.1111/jgs.12226

Sheffield KM, 2013, JAMA INTERN MED, V173, P542, DOI 10.1001/jamainternmed.2013.2912

Smith AK, 2013, JAMA INTERN MED, V173, P1241, DOI 10.1001/jamainternmed.2013.6053

Smith CD, 2012, ANN INTERN MED, V157, P284, DOI 10.7326/0003-4819-157-4-201208210-00496

Tak HJ, 2013, JAMA INTERN MED, V173, P1195, DOI 10.1001/jamainternmed.2013.6048

Tena JM, 2010, JAMA-J AM MED ASSOC, V303, P544

Tinetti ME, 2004, NEW ENGL J MED, V351, P2870, DOI 10.1056/NEJMsb042458

Vance J, 2013, J AM MED DIR ASSOC, V14, P639, DOI 10.1016/j.jamda.2013.07.004

NR 31

TC 1

Z9 1

U1 0

U2 6

PU SPRINGER HEIDELBERG

PI HEIDELBERG

PA TIERGARTENSTRASSE 17, D-69121 HEIDELBERG, GERMANY

SN 0948-6704

EI 1435-1269

J9 Z GERONTOL GERIATR

JI Z. Gerontol. Geriatr.

PD JAN

PY 2014

VL 47

IS 1

BP 23

EP 26

DI 10.1007/s00391-013-0594-5

PG 4

WC Geriatrics & Gerontology; Gerontology

WE Science Citation Index Expanded (SCI-EXPANDED); Social Science Citation Index (SSCI)

SC Geriatrics & Gerontology

GA 293MW

UT WOS:000329976900005

PM 24352408

DA 2024-08-08

ER

PT J

AU Blanc, AL

Spasojevic, S

Leszek, A

Théodoloz, M

Bonnabry, P

Fumeaux, T

Schaad, N

AF Blanc, A. -L.

Spasojevic, S.

Leszek, A.

Theodoloz, M.

Bonnabry, P.

Fumeaux, T.

Schaad, N.

TI A comparison of two tools to screen potentially inappropriate medication

in internal medicine patients

SO JOURNAL OF CLINICAL PHARMACY AND THERAPEUTICS

LA English

DT Article

DE internal medicine patients; medication review; PIM-Check; potentially

inappropriate medication; STOPP; START

ID DRUG-RELATED PROBLEMS; STOPP/START CRITERIA; ELDERLY-PATIENTS; OLDER

PATIENTS; PRESCRIBING CRITERIA; EXPLICIT CRITERIA; CONSENSUS PANEL;

POPULATION; PEOPLE; PRESCRIPTIONS

AB What is knownPotentially inappropriate medication (PIM) is an important issue for inpatient management; it has been associated with safety problems, such as increases in adverse drugs events, and with longer hospital stays and higher healthcare costs.

ObjectiveTo compare two PIM-screening toolsSTOPP/START and PIM-Checkapplied to internal medicine patients. A second objective was to compare the use of PIMs in readmitted and non-readmitted patients.

MethodA retrospective observational study, in the general internal medicine ward of a Swiss non-university hospital. We analysed a random sample of 50 patients, hospitalized in 2013, whose readmission within 30days of discharge had been potentially preventable, and compared them to a sample of 50 sex- and age-matched patients who were not readmitted. PIMs were screened using the STOPP/START tool, developed for geriatric patients, and the PIM-Check tool, developed for internal medicine patients. The time needed to perform each patient's analysis was measured. A clinical pharmacist counted and evaluated each PIM detected, based on its clinical relevance to the individual patient's case. The rates of screened and validated PIMs involving readmitted and non-readmitted patients were compared.

ResultsAcross the whole population, PIM-Check and STOPP/START detected 1348 and 537 PIMs, respectively, representing 13.5 and 5.4 PIMs/patient. Screening time was substantially shorter with PIM-Check than with STOPP/START (4 vs 10minutes, respectively). The clinical pharmacist judged that 45% and 42% of the PIMs detected using PIM-Check and STOPP/START, respectively, were clinically relevant to individual patients' cases. No significant differences in the rates of detected and clinically relevant PIM were found between readmitted and non-readmitted patients.

What is new and conclusionInternal medicine patients are frequently prescribed PIMs. PIM-Check's PIM detection rate was three times higher than STOPP/START's, and its screening time was shorter thanks to its electronic interface. Nearly half of the PIMs detected were judged to be non-clinically relevant, however, potentially overalerting the prescriber. These tools can, nevertheless, be considered useful in daily practice. Furthermore, the relevance of any PIM detected by these tools should always be carefully evaluated within the clinical context surrounding the individual patient.

C1 [Blanc, A. -L.; Bonnabry, P.] Geneva Univ Hosp, Pharm, Geneva, Switzerland.

[Blanc, A. -L.; Schaad, N.] Pharm Interhosp Cote, Morges, Switzerland.

[Blanc, A. -L.; Spasojevic, S.; Bonnabry, P.] Univ Geneva, Sch Pharmaceut Sci, Univ Lausanne, Geneva, Switzerland.

[Leszek, A.; Fumeaux, T.] Grp Hosp Ouest Leman, Nyon, Switzerland.

[Theodoloz, M.] Ensemble Hosp Cote, Morges, Switzerland.

[Fumeaux, T.; Schaad, N.] Univ Geneva, Fac Med, Geneva, Switzerland.

C3 University of Geneva; University of Lausanne; University of Geneva;

University of Geneva

RP Blanc, AL (corresponding author), Geneva Univ Hosp, Pharm, Geneva, Switzerland.

EM Annelaure.blanc@phel.ch

RI Fumeaux, Thierry/J-1537-2019; Anne-Laure, Blanc/Y-1701-2019

OI Fumeaux, Thierry/0000-0002-7427-6902;

FU Swiss Association of Public Health Administration and Hospital

Pharmacists GSASA

FX Anne-Laure Blanc, as a PhD student, was supported by the annual national

research grant from the Swiss Association of Public Health

Administration and Hospital Pharmacists GSASA, 2013. The GSASA had no

role in the design and conduct of this study, in the data analysis and

interpretation of results, nor in the preparation of the manuscript

CR Ahmad A, 2014, PATIENT PREFER ADHER, V8, P155, DOI 10.2147/PPA.S48357

ANQ, 2012, EV PSEUD MES REH POT

Anrys P, 2016, AGE AGEING, V45, P590, DOI 10.1093/ageing/afw114

Barry PJ, 2006, J CLIN PHARM THER, V31, P617, DOI 10.1111/j.1365-2710.2006.00783.x

BEERS MH, 1991, ARCH INTERN MED, V151, P1825

Cahir C, 2010, BRIT J CLIN PHARMACO, V69, P543, DOI 10.1111/j.1365-2125.2010.03628.x

Chang CB, 2011, BRIT J CLIN PHARMACO, V72, P482, DOI 10.1111/j.1365-2125.2011.04010.x

Chang CB, 2010, DRUG AGING, V27, P947, DOI 10.2165/11584850-000000000-00000

CHARLSON ME, 1987, J CHRON DIS, V40, P373, DOI 10.1016/0021-9681(87)90171-8

Cooper JA, 2016, EUR J CLIN PHARMACOL, V72, P583, DOI 10.1007/s00228-015-2003-z

Curtain CM, 2013, DRUG AGING, V30, P935, DOI 10.1007/s40266-013-0116-6

Dalleur O, 2012, DRUG AGING, V29, P829, DOI 10.1007/s40266-012-0016-1

Desnoyer A, 2016, PRESSE MED, V45, P957, DOI 10.1016/j.lpm.2016.06.033

Endres HG, 2016, PLOS ONE, V11, DOI 10.1371/journal.pone.0146811

Gallagher P, 2008, INT J CLIN PHARM TH, V46, P72

Gallagher PF, 2011, CLIN PHARMACOL THER, V89, P845, DOI 10.1038/clpt.2011.44

Gallagher P, 2011, EUR J CLIN PHARMACOL, V67, P1175, DOI 10.1007/s00228-011-1061-0

Gillespie U, 2013, PLOS ONE, V8, DOI 10.1371/journal.pone.0062401

Guignard B, 2015, EUR J INTERN MED, V26, P399, DOI 10.1016/j.ejim.2015.05.012

Hagstrom K, 2015, J AM GERIATR SOC, V63, P185, DOI 10.1111/jgs.13229

Halfon P, 2002, J CLIN EPIDEMIOL, V55, P573, DOI 10.1016/S0895-4356(01)00521-2

Halfon P, 2006, MED CARE, V44, P972, DOI 10.1097/01.mlr.0000228002.43688.c2

Hill-Taylor B, 2013, J CLIN PHARM THER, V38, P360, DOI 10.1111/jcpt.12059

Holt S, 2010, DTSCH ARZTEBL INT, V107, P543, DOI 10.3238/arztebl.2010.0543

Hudhra K, 2016, J EVAL CLIN PRACT, V22, P707, DOI 10.1111/jep.12521

Lang PO, 2015, NEUROL PSYCHIAT GERI, V2015, P323

Laroche ML, 2007, EUR J CLIN PHARMACOL, V63, P725, DOI 10.1007/s00228-007-0324-2

Mansur N, 2009, ANN PHARMACOTHER, V43, P177, DOI 10.1345/aph.1L461

McLeod PJ, 1997, CAN MED ASSOC J, V156, P385

O'Connor MN, 2012, DRUG AGING, V29, P437, DOI 10.2165/11632610-000000000-00000

O'Mahony D, 2008, AGE AGEING, V37, P138, DOI 10.1093/ageing/Afm189

O'Mahony D, 2015, AGE AGEING, V44, P213, DOI 10.1093/ageing/afu145

Reich O, 2014, PLOS ONE, V9, DOI 10.1371/journal.pone.0105425

Rognstad S, 2009, SCAND J PRIM HEALTH, V27, P153, DOI 10.1080/02813430902992215

Ryan C, 2013, INT J CLIN PHARM-NET, V35, P230, DOI 10.1007/s11096-012-9733-0

Ryan C, 2009, BRIT J CLIN PHARMACO, V68, P936, DOI 10.1111/j.1365-2125.2009.03531.x

San-José A, 2014, EUR J INTERN MED, V25, P710, DOI 10.1016/j.ejim.2014.07.011

Sehgal Vishal, 2013, J Family Med Prim Care, V2, P194, DOI 10.4103/2249-4863.117423

Spinewine A, 2007, LANCET, V370, P173, DOI 10.1016/S0140-6736(07)61091-5

Stafford AC, 2011, J CLIN PHARM THER, V36, P33, DOI 10.1111/j.1365-2710.2009.01151.x

Viktil KK, 2004, ANN PHARMACOTHER, V38, P942, DOI 10.1345/aph.1D531

NR 41

TC 9

Z9 9

U1 0

U2 5

PU WILEY

PI HOBOKEN

PA 111 RIVER ST, HOBOKEN 07030-5774, NJ USA

SN 0269-4727

EI 1365-2710

J9 J CLIN PHARM THER

JI J. Clin. Pharm. Ther.

PD APR

PY 2018

VL 43

IS 2

BP 232

EP 239

DI 10.1111/jcpt.12638

PG 8

WC Pharmacology & Pharmacy

WE Science Citation Index Expanded (SCI-EXPANDED)

SC Pharmacology & Pharmacy

GA FY1TN

UT WOS:000426596200010

PM 28990244

OA gold

DA 2024-08-08

ER

PT J

AU Bynum, DL

Wilson, LA

Ong, T

Callahan, KE

Dalton, T

Ohuabunwa, U

AF Bynum, Debra L.

Wilson, Lindsay A.

Ong, Thuan

Callahan, Kathryn E.

Dalton, Thomas

Ohuabunwa, Ugochi

TI Meeting American Geriatrics Society Competencies: Are Residents Meeting

Expectations for Quality Care of Older Adults?

SO JOURNAL OF THE AMERICAN GERIATRICS SOCIETY

LA English

DT Article

DE resident education; geriatric competencies

ID INTERNAL-MEDICINE-RESIDENTS; KNOWLEDGE; AWARENESS; PROGRAMS; STUDENTS;

HAZARDS

AB In order to determine how often internal medicine and family medicine residents performed specific actions related to the geriatric competencies established by the American Geriatrics Society (AGS) when caring for older hospitalized adults, a cross-sectional anonymous survey of residents at the University of North Carolina, University of Washington, Wake Forest University, Duke University, and Emory University was undertaken. Data on frequency of self-reported behaviors were analyzed, with comparisons made for different levels of training, institution, and program. A total of 375 residents responded for an overall response rate of 48%. Residents reported that they often do not demonstrate all of the AGS recommended core competencies when caring for older adults in the hospital setting. Residents report more frequently performing activities that are routinely integrated into hospital systems such as reviewing medication lists, working with an interdisciplinary team, evaluating for inappropriate bladder catheters, and evaluating for pressure ulcers. There were no consistent differences between institutions and only minor differences noted between Family Medicine and Internal Medicine residents. Operationalizing core competencies by integrating them into hospital systems' quality process indicators may prompt more consistent high-quality care and ensure systems support residents' competence.

C1 [Bynum, Debra L.; Wilson, Lindsay A.] Univ N Carolina, Div Geriatr Med, Ctr Aging & Hlth, Chapel Hill, NC 27599 USA.

[Ong, Thuan] Univ Washington, Harborview Med Ctr, Div Gerontol & Geriatr Med, Seattle, WA 98104 USA.

[Callahan, Kathryn E.] Wake Forest Sch Med, Dept Internal Med, Sect Gerontol & Geriatr Med, Sticht Ctr Aging, Winston Salem, NC USA.

[Dalton, Thomas] Duke Univ, Med Ctr, Dept Med, Div Geriatr, Durham, NC 27710 USA.

[Ohuabunwa, Ugochi] Emory Univ, Sch Med, Div Gen Med & Geriatr, Atlanta, GA USA.

C3 University of North Carolina; University of North Carolina Chapel Hill;

Harborview Medical Center; University of Washington; University of

Washington Seattle; Wake Forest University; Duke University; Emory

University

RP Bynum, DL (corresponding author), Univ N Carolina, 101 Manning Dr,126 MacNider Bldg, Chapel Hill, NC 27599 USA.

EM bynumdl@med.unc.edu

RI Dalton, Thomas/V-7714-2019

FU NIA NIH HHS [L30 AG043161] Funding Source: Medline

CR Adams AS, 1999, INT J QUAL HEALTH C, V11, P187, DOI 10.1093/intqhc/11.3.187

[Anonymous], 2015, National patient safety goals

Boustani M, 2010, J HOSP MED, V5, P69, DOI 10.1002/jhm.589

Bragg EJ, 2005, ACAD MED, V80, P279, DOI 10.1097/00001888-200503000-00014

Drickamer MA, 2006, J GEN INTERN MED, V21, P1230, DOI 10.1111/j.1525-1497.2006.00585.x

Duthie E H Jr, 1983, Gerontol Geriatr Educ, V3, P233

Fernandez HM, 2008, ARCH INTERN MED, V168, P390, DOI 10.1001/archinternmed.2007.87

Fineberg Harvey V, 2008, Medscape J Med, V10, P188

Jellinek SP, 2008, AM J GERIATR PHARMAC, V6, P82, DOI 10.1016/j.amjopharm.2008.06.003

Lindberg MC, 1996, J GEN INTERN MED, V11, P397, DOI 10.1007/BF02600185

Litvin CB, 2012, J AM GERIATR SOC, V60, P1145, DOI 10.1111/j.1532-5415.2012.03960.x

Moran WP, 2012, ACAD MED, V87, P635, DOI 10.1097/ACM.0b013e31824d4a10

Oyler J, 2008, J GEN INTERN MED, V23, P927, DOI 10.1007/s11606-008-0549-5

Oyler J, 2011, J GEN INTERN MED, V26, P221, DOI 10.1007/s11606-010-1547-y

Podrazik PM, 2008, J HOSP MED, V3, P384, DOI 10.1002/jhm.348

Smith KL, 2012, J HOSP MED, V7, P148, DOI 10.1002/jhm.945

Thomas DC, 2003, ANN INTERN MED, V139, P628, DOI 10.7326/0003-4819-139-7-200310070-00037

Vinci LM, 2014, AM J MED QUAL, V29, P277, DOI 10.1177/1062860613498264

Warshaw GA, 2006, J AM GERIATR SOC, V54, P1603, DOI 10.1111/j.1532-5415.2006.00895.x

Wilkerson LM, 2014, J AM GERIATR SOC, V62, P727, DOI 10.1111/jgs.12733

Williams Brent C, 2010, J Grad Med Educ, V2, P373, DOI 10.4300/JGME-D-10-00065.1

NR 21

TC 5

Z9 5

U1 1

U2 8

PU WILEY

PI HOBOKEN

PA 111 RIVER ST, HOBOKEN 07030-5774, NJ USA

SN 0002-8614

EI 1532-5415

J9 J AM GERIATR SOC

JI J. Am. Geriatr. Soc.

PD SEP

PY 2015

VL 63

IS 9

BP 1918

EP 1923

DI 10.1111/jgs.13598

PG 6

WC Geriatrics & Gerontology; Gerontology

WE Science Citation Index Expanded (SCI-EXPANDED); Social Science Citation Index (SSCI)

SC Geriatrics & Gerontology

GA CU8PI

UT WOS:000363804800026

PM 26313811

OA Green Accepted

DA 2024-08-08

ER

PT J

AU Egloff, HM

West, CP

Wang, AT

Lowe, KM

Varayil, JE

Beckman, TJ

Sawatsky, A

AF Egloff, Heidi M.

West, Colin P.

Wang, Amy T.

Lowe, Katie M.

Varayil, Jithinraj Edakkanambeth

Beckman, Thomas J.

Sawatsky, Adam P.

TI Publication Rates of Abstracts Presented at the Society of General

Internal Medicine Annual Meeting

SO JOURNAL OF GENERAL INTERNAL MEDICINE

LA English

DT Article

DE general internal medicine; abstracts; publication rates; impact factor;

publication bias

ID FULL PUBLICATION; BIAS; ASSOCIATION; PUBLISH; IMPACT; FUTURE; FATE

AB accepted at scientific meetings are often not subsequently published. Data on publication rates are largely from subspecialty and surgical studies.

The aims of this study were to 1) determine publication rates of abstracts presented at a general internal medicine meeting; 2) describe research activity among academic general internists; 3) identify factors associated with publication and with the impact factor of the journal of publication; and 4) evaluate for publication bias.

Retrospective cohort study.

All scientific abstracts presented at the Society of General Internal Medicine 2009 Annual Meeting.

Publication rates were determined by searching for full-text publications in MEDLINE. Data were abstracted regarding authors' institution, research topic category, number of study sites, sample size, study design, statistical significance (p value and confidence interval) in abstract and publication, journal of publication, publication date, and journal impact factor.

Of the 578 abstracts analyzed, 274 (47.4%) were subsequently published as a full article in a peer-reviewed journal indexed in MEDLINE. In a multivariable model adjusting for institution site, research topic, number of study sites, study design, sample size, and abstract results, publication rates for academic general internists were highest in the areas of medical education (52.5%, OR 5.05, 95% CI 1.57-17.25, reference group Veterans Affairs (VA)-based research, publication rate 36.7%), mental health/substance use (67.7%, OR 4.16, 95% CI 1.39-13.06), and aging/geriatrics/end of life (65.7%, OR 3.31, 95% CI 1.15-9.94, p = 0.01 across topics). Publication rates were higher for multicenter studies than single-institution studies (52.4% vs. 40.4%, OR 1.66, 95% CI 1.10-2.52, p = 0.04 across categories). Randomized controlled trials had higher publication rates than other study designs (66.7% vs. 45.9%, OR 2.72, 95% CI 1.30-5.94, p = 0.03 across study designs). Studies with positive results did not predict higher publication rates than negative studies (OR 0.89, 95% CI 0.6-1.31, p = 0.21).

This study demonstrated that 47.4% of abstracts presented at a general internal medicine national conference were subsequently published in a peer-reviewed journal indexed in MEDLINE.

C1 [Egloff, Heidi M.] Univ Michigan Hlth Syst, Div Hematol Oncol, Ann Arbor, MI USA.

[West, Colin P.; Beckman, Thomas J.; Sawatsky, Adam P.] Mayo Clin, Div Gen Internal Med, Rochester, MN 55905 USA.

[West, Colin P.] Mayo Clin, Div Biomed Stat & Informat, Rochester, MN USA.

[Wang, Amy T.] Harbor UCLA Med Ctr, Div Gen Internal Med, Torrance, CA 90509 USA.

[Lowe, Katie M.] Mayo Clin, Dept Med, Rochester, MN USA.

[Varayil, Jithinraj Edakkanambeth] Univ Illinois, Dept Family & Community Med, Chicago, IL USA.

C3 University of Michigan System; University of Michigan; Mayo Clinic; Mayo

Clinic; University of California System; University of California Los

Angeles; University of California Los Angeles Medical Center; Mayo

Clinic; University of Illinois System; University of Illinois Chicago;

University of Illinois Chicago Hospital

RP Sawatsky, A (corresponding author), Mayo Clin, Div Gen Internal Med, Rochester, MN 55905 USA.

EM sawatsky.adam@mayo.edu

CR Armstrong K, 2013, J GEN INTERN MED, V28, P845, DOI 10.1007/s11606-013-2334-3

Callaham ML, 1998, JAMA-J AM MED ASSOC, V280, P254, DOI 10.1001/jama.280.3.254

De Angelis C, 2004, ANN INTERN MED, V141, P477, DOI 10.7326/0003-4819-141-6-200409210-00109

de Meijer VE, 2016, AM J SURG, V211, P166, DOI 10.1016/j.amjsurg.2015.06.017

DEBELLEFEUILLE C, 1992, ANN ONCOL, V3, P187, DOI 10.1093/oxfordjournals.annonc.a058147

DICKERSIN K, 1992, JAMA-J AM MED ASSOC, V267, P374, DOI 10.1001/jama.267.3.374

DICKERSIN K, 1990, JAMA-J AM MED ASSOC, V263, P1385, DOI 10.1001/jama.263.10.1385

EASTERBROOK PJ, 1991, LANCET, V337, P867, DOI 10.1016/0140-6736(91)90201-Y

Eloubeidi MA, 2001, GASTROINTEST ENDOSC, V53, P275, DOI 10.1067/mge.2001.113383

Hopewell S, 2003, INTERN MED J, V33, P192, DOI 10.1046/j.1445-5994.2003.00353.x

Koene H R, 1994, Ned Tijdschr Geneeskd, V138, P1868

Krzyzanowska MK, 2003, JAMA-J AM MED ASSOC, V290, P495, DOI 10.1001/jama.290.4.495

Larson EB, 2004, J GEN INTERN MED, V19, P69, DOI 10.1111/j.1525-1497.2004.31337.x

Levinson W, 2002, JAMA-J AM MED ASSOC, V288, P2045, DOI 10.1001/jama.288.16.2045

Patsopoulos NA, 2005, JAMA-J AM MED ASSOC, V293, P2362, DOI 10.1001/jama.293.19.2362

Pollart SM, 2015, ACAD MED, V90, P365, DOI 10.1097/ACM.0000000000000458

Sawatsky AP, 2015, J GEN INTERN MED, V30, P1172, DOI 10.1007/s11606-015-3269-7

Scherer RW, 2007, COCHRANE DB SYST REV, DOI 10.1002/14651858.MR000005.pub4

SCHERER RW, 1994, JAMA-J AM MED ASSOC, V272, P158, DOI 10.1001/jama.272.2.158

Sprague S, 2003, J BONE JOINT SURG AM, V85A, P158, DOI 10.2106/00004623-200301000-00024

Sridharan L, 2009, ARCH INTERN MED, V169, P1022, DOI 10.1001/archinternmed.2009.100

von Elm Erik, 2003, BMC Med Res Methodol, V3, P12

Weber EJ, 1998, JAMA-J AM MED ASSOC, V280, P257, DOI 10.1001/jama.280.3.257

WEINTRAUB WH, 1987, J PEDIATR SURG, V22, P11, DOI 10.1016/S0022-3468(87)80005-2

NR 24

TC 18

Z9 19

U1 0

U2 11

PU SPRINGER

PI NEW YORK

PA 233 SPRING ST, NEW YORK, NY 10013 USA

SN 0884-8734

EI 1525-1497

J9 J GEN INTERN MED

JI J. Gen. Intern. Med.

PD JUN

PY 2017

VL 32

IS 6

BP 673

EP 678

DI 10.1007/s11606-017-3990-5

PG 6

WC Health Care Sciences & Services; Medicine, General & Internal

WE Science Citation Index Expanded (SCI-EXPANDED)

SC Health Care Sciences & Services; General & Internal Medicine

GA EV8XQ

UT WOS:000402068300017

PM 28138874

OA Bronze, Green Published

DA 2024-08-08

ER

PT J

AU Mozzini, C

Cominacini, L

Casadei, A

Schiavone, C

Soresi, M

AF Mozzini, Chiara

Cominacini, Luciano

Casadei, Alder

Schiavone, Cosima

Soresi, Maurizio

TI Ultrasonography in Heart Failure: A Story that Matters

SO CURRENT PROBLEMS IN CARDIOLOGY

LA English

DT Review

ID ANKLE-BRACHIAL INDEX; INFERIOR VENA-CAVA; ABDOMINAL AORTIC-ANEURYSMS;

INTIMA-MEDIA THICKNESS; LUNG ULTRASOUND; CARDIOVASCULAR-DISEASE;

MYOCARDIAL-INFARCTION; ATHEROSCLEROSIS RISK; PULMONARY CONGESTION;

ARTERIAL COMPLIANCE

AB Heart failure (HF) is a clinical syndrome caused by structural and/or functional cardiac abnormalities, resulting in a reduced cardiac output and/or elevated intracardiac pressures at rest or during stress. It is the leading cause of hospitalization in Internal Medicine departments. This article aims at reviewing evidence of the importance of ultrasound in HF both for hospitalized patients and in the follow-up. Ultrasound may be used as a recovery monitoring instrument at the bedside and also as a global cardiovascular assessment tool for these patients. HF represents an exciting opportunity to create an integrative ultrasound approach in Internal Medicine and/or Geriatric departments. The authors plan a five-step ultrasound examination to evaluate and monitor HF patients during hospitalization and follow-up. They call this examination: the "ABCDE" score. It includes the evaluations of A, the ankle-brachial index, B, the B-lines, C, the carotid intima media thickness, D, the diameter of the abdominal aorta and of the inferior cava vein and E, the echocardiographic assessment of the ejection fraction. This score may represent an integrative ultrasound approach in Internal Medicine and/or Geriatric departments.

C1 [Schiavone, Cosima] Univ G dAnnunzio, Appl Med Tech Sci, Chieti, Italy.

[Schiavone, Cosima; Soresi, Maurizio] SIUMB, Ultrasound Soc, Chieti, Italy.

[Mozzini, Chiara] Univ Verona, Sect Internal Med, Med Doctor Act, Verona, Italy.

[Casadei, Alder] SIUMB, Ultrasound Soc South Tyrol, Verona, Italy.

[Cominacini, Luciano] Univ Verona, Sch Med, Internal Med, Verona, Italy.

[Cominacini, Luciano] Univ Hosp Verona, Internal Med Unit, Verona, Italy.

[Soresi, Maurizio] Univ Palermo, Internal Med, Palermo, Italy.

C3 G d'Annunzio University of Chieti-Pescara; University of Verona;

University of Verona; University of Verona; Azienda Ospedaliera

Universitaria Integrata Verona; University of Palermo

RP Mozzini, C (corresponding author), Univ Verona, Sect Internal Med, Med Doctor Act, Verona, Italy.

RI mozzini, chiara/K-9570-2015; Soresi, Maurizio/AAY-2045-2020; mozzini,

chiara/AAW-3028-2021

OI Soresi, Maurizio/0000-0001-7850-555X; mozzini,

chiara/0000-0002-4414-6576

CR Alahdab F, 2015, J VASC SURG, V61, p42S, DOI 10.1016/j.jvs.2014.12.008

Ang SH, 2012, CURR CARDIOL REV, V8, P123, DOI 10.2174/157340312801784907

Benetos A, 1997, J HYPERTENS, V15, pS89, DOI 10.1097/00004872-199715022-00009

Brennan JM, 2007, J AM SOC ECHOCARDIOG, V20, P857, DOI 10.1016/j.echo.2007.01.005

Ciccarese F, 2015, RESPIRATION, V90, P56, DOI 10.1159/000430994

Cogliati C, 2016, INT J CARDIOL, V218, P104, DOI 10.1016/j.ijcard.2016.05.010

Cuspidi C, 2001, BLOOD PRESSURE, V10, P142, DOI 10.1080/080370501753182352

Dokainish H, 2011, EUR J ECHOCARDIOGR, V12, P857, DOI 10.1093/ejechocard/jer157

Effoe VS, 2014, J AM HEART ASSOC, V3, DOI 10.1161/JAHA.114.000797

Fernandes VRS, 2006, J AM COLL CARDIOL, V47, P2420, DOI 10.1016/j.jacc.2005.12.075

Finn AV, 2010, ARTERIOSCL THROM VAS, V30, P177, DOI 10.1161/ATVBAHA.108.173609

Forsdahl SH, 2010, INT J EPIDEMIOL, V39, P225, DOI 10.1093/ije/dyp320

Fowkes FGR, 2008, JAMA-J AM MED ASSOC, V300, P197, DOI 10.1001/jama.300.2.197

GARGANI L, 2014, CARDIOVASC ULTRASOUN, V12

Gargani L, 2011, CARDIOVASC ULTRASOUN, V9, DOI 10.1186/1476-7120-9-6

Gundersen GH, 2016, HEART, V102, P29, DOI 10.1136/heartjnl-2015-307798

Gupta DK, 2014, JACC-HEART FAIL, V2, P447, DOI 10.1016/j.jchf.2014.05.008

Halkar Meghana, 2013, Curr Heart Fail Rep, V10, P450, DOI 10.1007/s11897-013-0165-5

Hendry PB, 2016, CARDIOL RES, V7, P110, DOI 10.14740/cr473w

Hodis HN, 1998, ANN INTERN MED, V128, P262, DOI 10.7326/0003-4819-128-4-199802150-00002

LAGE SG, 1994, AM J CARDIOL, V74, P691, DOI 10.1016/0002-9149(94)90311-5

Leong KTG, 2017, AM J CARDIOL, V119, P1428, DOI 10.1016/j.amjcard.2017.01.026

Lewis EF, 2003, J AM COLL CARDIOL, V42, P1446, DOI 10.1016/S0735-1097(03)01057-X

Lichtenstein DA, 2008, CHEST, V134, P117, DOI 10.1378/chest.07-2800

Loehr LR, 2008, AM J CARDIOL, V101, P1016, DOI 10.1016/j.amjcard.2007.11.061

Lorenz MW, 2007, CIRCULATION, V115, P459, DOI 10.1161/CIRCULATIONAHA.106.628875

Maggioni AP, 2010, EUR J HEART FAIL, V12, P1076, DOI 10.1093/eurjhf/hfq154

Martindale JL, 2013, EUR J EMERG MED, V20, P356, DOI 10.1097/MEJ.0b013e32835c2b88

Marwick TH, 2010, JACC-CARDIOVASC IMAG, V3, P429, DOI 10.1016/j.jcmg.2010.02.002

Miglioranza MH, 2013, JACC-CARDIOVASC IMAG, V6, P1141, DOI 10.1016/j.jcmg.2013.08.004

Miura T, 2017, PLOS ONE, V12, DOI 10.1371/journal.pone.0177609

MORENO FL, 1984, AM J CARDIOL, V53, P579, DOI 10.1016/0002-9149(84)90034-1

Mozzini C, 2018, INTERN EMERG MED, V13, P27, DOI 10.1007/s11739-017-1738-1

Newman AB, 1999, ARTERIOSCL THROM VAS, V19, P538, DOI 10.1161/01.ATV.19.3.538

Nishimura H, 2017, HEART VESSELS, V32, P295, DOI 10.1007/s00380-016-0873-3

O'Leary DH, 1999, NEW ENGL J MED, V340, P14, DOI 10.1056/NEJM199901073400103

Parrinello G, 2004, J HUM HYPERTENS, V18, P201, DOI 10.1038/sj.jhh.1001653

Paulus WJ, 2007, EUR HEART J, V28, P2539, DOI 10.1093/eurheartj/ehm037

Platz E, 2016, EUR HEART J, V37, P1244, DOI 10.1093/eurheartj/ehv745

Ponikowski P, 2016, EUR HEART J, V37, P2129, DOI 10.1093/eurheartj/ehw128

Qazi S, 2017, CIRC-CARDIOVASC IMAG, V10, DOI 10.1161/CIRCIMAGING.117.006776

Rea G, 2015, RESPIRATION, V90, P522, DOI 10.1159/000441010

Sakaguchi T, 2017, J CARDIOL DEC, V26, DOI [10.1016/j.jjcc.2017.11.01, DOI 10.1016/J.JJCC.2017.11.01]

Sako H, 2017, CARDIOL RES, V8, P339, DOI 10.14740/cr640w

Scrutinio D, 2014, J HEART LUNG TRANSPL, V33, P404, DOI 10.1016/j.healun.2013.12.005

Scrutinio D, 2013, INT J CARDIOL, V168, P2120, DOI 10.1016/j.ijcard.2013.01.005

Sohrabi S, 2014, BRIT J SURG, V101, P1238, DOI 10.1002/bjs.9567

Soldati G, 2017, J ULTRASOUND, V20, P91, DOI 10.1007/s40477-017-0244-7

Solomon SD, 2005, CIRCULATION, V111, P3411, DOI 10.1161/CIRCULATIONAHA.104.508093

Spencer KT, 2013, J AM SOC ECHOCARDIOG, V26, P567, DOI 10.1016/j.echo.2013.04.001

Sperandeo M, 2016, EUR J HEART FAIL, V18, P214, DOI 10.1002/ejhf.451

Sperandeo M, 2014, RADIOL MED, V119, P729, DOI 10.1007/s11547-014-0385-0

Suzuki T, 2018, HEART FAIL CLIN, V14, pIX, DOI 10.1016/j.hfc.2017.08.012

Trovato GM, 2016, WORLD J CARDIOL, V8, P566, DOI 10.4330/wjc.v8.i10.566

Trovato GM, 2015, RESPIRATION, V90, P85, DOI 10.1159/000375316

Trovato GM, 2015, RESPIRATION, V89, P175, DOI 10.1159/000369037

Trovato GM, 2013, INTENS CARE MED, V39, P1662, DOI 10.1007/s00134-013-2965-1

Trovato GM, 2013, AM J RESP CRIT CARE, V187, P780, DOI 10.1164/ajrccm.187.7.780

Tsao CW, 2015, J AM HEART ASSOC, V4, DOI 10.1161/JAHA.115.002189

Volpicelli G, 2012, INTENS CARE MED, V38, P577, DOI 10.1007/s00134-012-2513-4

White C, 2007, NEW ENGL J MED, V356, P1241, DOI 10.1056/NEJMcp064483

Wilkins JT, 2012, AM J HYPERTENS, V25, P535, DOI 10.1038/ajh.2012.13

Yavasi Ö, 2014, AM J EMERG MED, V32, P403, DOI 10.1016/j.ajem.2013.12.046

Zanforlin A, 2014, JACC-CARDIOVASC IMAG, V7, P635, DOI 10.1016/j.jcmg.2013.12.018

NR 64

TC 5

Z9 6

U1 0

U2 11

PU MOSBY-ELSEVIER

PI NEW YORK

PA 360 PARK AVENUE SOUTH, NEW YORK, NY 10010-1710 USA

SN 0146-2806

EI 1535-6280

J9 CURR PROB CARDIOLOGY

JI Curr. Probl. Cardiol.

PD APR

PY 2019

VL 44

IS 4

BP 114

EP 136

DI 10.1016/j.cpcardiol.2018.05.003

PG 23

WC Cardiac & Cardiovascular Systems

WE Science Citation Index Expanded (SCI-EXPANDED)

SC Cardiovascular System & Cardiology

GA HK6LI

UT WOS:000458090700001

PM 30172551

DA 2024-08-08

ER

PT J

AU Orso, F

Pratesi, A

Herbst, A

Baroncini, AC

Bacci, F

Ciuti, G

Berni, A

Tozzetti, C

Nozzoli, C

Pignone, AM

Poggesi, L

Gabbani, L

Di Bari, M

Fattirolli, F

Milli, M

Ungar, A

Marchionni, N

Baldasseroni, S

AF Orso, Francesco

Pratesi, Alessandra

Herbst, Andrea

Baroncini, Anna Chiara

Bacci, Francesca

Ciuti, Gabriele

Berni, Andrea

Tozzetti, Camilla

Nozzoli, Carlo

Pignone, Alberto Moggi

Poggesi, Loredana

Gabbani, Luciano

Di Bari, Mauro

Fattirolli, Francesco

Milli, Massimo

Ungar, Andrea

Marchionni, Niccolo

Baldasseroni, Samuele

TI Acute heart failure in the elderly: setting related differences in

clinical features and management

SO JOURNAL OF GERIATRIC CARDIOLOGY

LA English

DT Article

ID ESC; EPIDEMIOLOGY; OUTCOMES; MORTALITY; PROGRAM; HFA; AGE

AB BACKGROUND Administrative data show that acute heart failure (HF) patients are older than those enrolled in clinical registries and frequently admitted to non-cardiological settings of care. The purpose of this study was to describe clinical characteristics of old patients hospitalised for acute HF in Cardiology, Internal Medicine or Geriatrics wards.

METHODS Data came from ATHENA (AcuTe Heart failurE in advaNced Age) registry which included elderly patients (= 65 years) admitted to the above mentioned settings of care from December 1, 2014 to December 1, 2015.

RESULTS We enrolled 396 patients, 15.4% assigned to Cardiology, 69.7% to Internal Medicine, and 14.9% to a Geriatrics ward. Mean age was 83.5 +/- 7.6 years (51.8% of patients = 85 years) and was higher in patients admitted to Geriatrics ( P < 0.001); more than half were females. Medical treatments did not differ significantly among settings of care (in a context of a low prescription rate of renin-angiotensin-aldosterone system inhibitors) whereas significant differences were observed in comorbidity patterns and management guidelines recommendation adherence for decongestion evaluation with comparison of weight and N-terminal pro-B-type natriuretic peptide levels on admission and at discharge (both P = 0.035 and P < 0.001), echocardiographic evaluation ( P < 0.001) and follow-up visits planning ( P < 0.001), all higher in Cardiology. Mean in-hospital length of stay was 9 +/- 5.9 days, significantly higher in Geriatrics (13.7 +/- 6.5 days) and Cardiology (9.9 +/- 6.7 days) compared to Internal Medicine (8 +/- 5.2 days), P < 0.001. In-hospital mortality was 9.3%, resulting higher in Geriatrics (18.6%) and Cardiology (16.4%) than Internal Medicine (5.8%), P = 0.001.

CONCLUSIONS In elderly patients hospitalised for acute HF, clinical characteristics and management differ significantly according to the setting of admission.

C1 [Orso, Francesco; Herbst, Andrea; Baroncini, Anna Chiara; Di Bari, Mauro; Ungar, Andrea; Baldasseroni, Samuele] Azienda Osped Univ Careggi, Div Geriatr Med, Heart Failure Clin, Florence, Italy.

[Orso, Francesco; Herbst, Andrea; Baroncini, Anna Chiara; Di Bari, Mauro; Ungar, Andrea; Baldasseroni, Samuele] Azienda Osped Univ Careggi, Intens Care Unit, Florence, Italy.

[Pratesi, Alessandra; Di Bari, Mauro; Fattirolli, Francesco; Ungar, Andrea; Marchionni, Niccolo] Univ Florence, Dept Expt & Clin Med, Florence, Italy.

[Bacci, Francesca; Nozzoli, Carlo] Azienda Osped Univ Careggi, Div Internal Med 1, Florence, Italy.

[Ciuti, Gabriele; Pignone, Alberto Moggi] Azienda Osped Univ Careggi, Div Internal Med 4, Florence, Italy.

[Berni, Andrea; Tozzetti, Camilla; Poggesi, Loredana] Azienda Osped Univ Careggi, Div Internal Med 3, Florence, Italy.

[Gabbani, Luciano] Azienda Osped Univ Careggi, Div Med, Geriatr Dept, Florence, Italy.

[Fattirolli, Francesco; Marchionni, Niccolo] Azienda Osped Univ Careggi, Dept Cardiothoracovasc Med, Florence, Italy.

[Milli, Massimo] Santa Maria Nuova Hosp, Div Cardiol, Florence, Italy.

C3 University of Florence; Azienda Ospedaliero Universitaria Careggi;

University of Florence; Azienda Ospedaliero Universitaria Careggi;

University of Florence; University of Florence; Azienda Ospedaliero

Universitaria Careggi; University of Florence; Azienda Ospedaliero

Universitaria Careggi; University of Florence; Azienda Ospedaliero

Universitaria Careggi; University of Florence; Azienda Ospedaliero

Universitaria Careggi; University of Florence; Azienda Ospedaliero

Universitaria Careggi; IRCCS Arcispedale S. Maria Nuova

RP Orso, F (corresponding author), Azienda Osped Univ Careggi, Div Geriatr Med, Heart Failure Clin, Florence, Italy.

EM orsof@aou-careggi.toscana.it

RI nozzoli, carlo/AAW-9197-2020; Pignone, Alberto Moggi/IAR-6927-2023;

Orso, Francesco/AAS-7483-2020

OI Pignone, Alberto Moggi/0000-0001-5828-6206;

CR Abraham WT, 2008, J AM COLL CARDIOL, V52, P347, DOI 10.1016/j.jacc.2008.04.028

Berthelot E., 2018, Health Care Curr Rev., V6, P2

Cannon JA, 2017, J CARD FAIL, V23, P464, DOI 10.1016/j.cardfail.2017.04.007

Chioncel O, 2017, EUR J HEART FAIL, V19, P1574, DOI 10.1002/ejhf.813

Chioncel O, 2017, EUR J HEART FAIL, V19, P1242, DOI 10.1002/ejhf.890

Dharmarajan K, 2017, HEART FAIL CLIN, V13, P417, DOI 10.1016/j.hfc.2017.02.001

Dharmarajan K, 2013, JAMA-J AM MED ASSOC, V309, P355, DOI 10.1001/jama.2012.216476

Di Lenarda A, 2003, AM HEART J, V146, DOI 10.1016/S0002-8703(03)00315-6

Dunlay Shannon M, 2014, Curr Heart Fail Rep, V11, P404, DOI 10.1007/s11897-014-0220-x

Fonarow GC, 2009, AM J CARDIOL, V104, P107, DOI 10.1016/j.amjcard.2009.02.057

Howell EH, 2017, SAGE OPEN MED, V5, DOI 10.1177/2050312117700301

Johnell K, 2007, DRUG SAFETY, V30, P911, DOI 10.2165/00002018-200730100-00009

Kapoor JR, 2016, JACC-HEART FAIL, V4, P464, DOI 10.1016/j.jchf.2016.02.017

Komajda M, 2009, EUR HEART J, V30, P478, DOI 10.1093/eurheartj/ehn539

Levey AS, 2009, ANN INTERN MED, V150, P604, DOI 10.7326/0003-4819-150-9-200905050-00006

Maggioni AP, 2016, EUR J HEART FAIL, V18, P402, DOI 10.1002/ejhf.471

Maggioni AP, 2013, EUR J HEART FAIL, V15, P808, DOI 10.1093/eurjhf/hft050

McMurray JJV, 2012, EUR J HEART FAIL, V14, P803, DOI 10.1093/eurjhf/hfs105

Oliva F, 2012, EUR J HEART FAIL, V14, P1208, DOI 10.1093/eurjhf/hfs117

RICKHAM PP, 1964, BRIT MED J, V2, P173

Roger VL, 2013, CIRC RES, V113, P646, DOI 10.1161/CIRCRESAHA.113.300268

Rutledge T, 2006, J AM COLL CARDIOL, V48, P1527, DOI 10.1016/j.jacc.2006.06.055

Senni M, 2014, INT J CARDIOL, V173, P163, DOI 10.1016/j.ijcard.2014.02.018

Targher G, 2017, EUR J HEART FAIL, V19, P54, DOI 10.1002/ejhf.679

Teixeira A, 2015, EUR J HEART FAIL, V17, P1114, DOI 10.1002/ejhf.330

Van Spall HGC, 2007, JAMA-J AM MED ASSOC, V297, P1233, DOI 10.1001/jama.297.11.1233

Ziaeian B, 2016, NAT REV CARDIOL, V13, P368, DOI 10.1038/nrcardio.2016.25

NR 27

TC 3

Z9 3

U1 0

U2 5

PU SCIENCE PRESS

PI BEIJING

PA 16 DONGHUANGCHENGGEN NORTH ST, BEIJING 100717, PEOPLES R CHINA

SN 1671-5411

J9 J GERIATR CARDIOL

JI J. Geriatr. Cardiol.

PY 2021

VL 18

IS 6

BP 407

EP 415

DI 10.11909/j.issn.1671-5411.2021.06.003

PG 9

WC Cardiac & Cardiovascular Systems; Geriatrics & Gerontology

WE Science Citation Index Expanded (SCI-EXPANDED)

SC Cardiovascular System & Cardiology; Geriatrics & Gerontology

GA UK6VD

UT WOS:000692104500001

PM 34220970

DA 2024-08-08

ER

PT J

AU Day, H

Eckstrom, E

Lee, S

Wald, H

Counsell, S

Rich, E

AF Day, Hollis

Eckstrom, Elizabeth

Lee, Sei

Wald, Heidi

Counsell, Steven

Rich, Eugene

TI Optimizing Health for Complex Adults in Primary Care: Current Challenges

and a Way Forward

SO JOURNAL OF GENERAL INTERNAL MEDICINE

LA English

DT Article

DE implementation research; geriatrics; primary care

ID RANDOMIZED-CONTROLLED-TRIAL; IMPLEMENTATION SCIENCE; ACADEMIC MEDICINE;

RESEARCH AGENDA; GERIATRIC CARE

AB As the population ages, the quantity and complexity of comorbidities only increases in the primary care setting. Health systems strive to improve quality of care and enhance cost savings, but current administrative and payment systems do not easily support the implementation of existing evidence and best practices for multimorbid adults in most primary care offices. This perspectives piece sets forth a research agenda in the area of implementation science at the intersection of geriatrics and general internal medicine. We challenge academic medical centers, medical societies, journals, and funders to actively value and support investigation in this area as much as traditional research pathways. (C) Society of General Internal Medicine 2014

C1 [Day, Hollis] Univ Pittsburgh Med Ctr, Div Gen Internal Med, Pittsburgh, PA 15261 USA.

[Eckstrom, Elizabeth] Oregon Hlth & Sci Univ, Div Gen Internal Med & Geriatr, Portland, OR 97201 USA.

[Lee, Sei] Univ Calif San Francisco, Div Geriatr, San Francisco, CA 94143 USA.

[Wald, Heidi] Univ Colorado, Div Hlth Care Policy & Res, Boulder, CO 80309 USA.

[Counsell, Steven] Indiana Univ Sch Med, Div Gen Internal Med & Geriatr, Indianapolis, IN 46202 USA.

[Rich, Eugene] Math Policy Res, Washington, DC USA.

C3 Pennsylvania Commonwealth System of Higher Education (PCSHE); University

of Pittsburgh; Oregon Health & Science University; University of

California System; University of California San Francisco; University of

Colorado System; University of Colorado Boulder; Indiana University

System; Indiana University Bloomington; Mathematica

RP Day, H (corresponding author), Univ Pittsburgh Med Ctr, Div Gen Internal Med, 3550 Terrace St,M211 Scaife Hall, Pittsburgh, PA 15261 USA.

EM dayh@upmc.edu

OI Day, Hollis/0000-0001-8817-5160

FU Association of Subspecialty Professors for the Society of General

Internal Medicine Geriatrics Task Force

FX This paper was funded through a grant obtained from the Association of

Subspecialty Professors for the Society of General Internal Medicine

Geriatrics Task Force.

CR Beswick AD, 2008, LANCET, V371, P725, DOI 10.1016/S0140-6736(08)60342-6

Bonham AC, 2010, HEALTH AFFAIR, V29, P1901, DOI 10.1377/hlthaff.2010.0790

Bonham AC, 2010, ACAD MED, V85, P1551, DOI 10.1097/ACM.0b013e3181effdbc

Boult C, 2011, ARCH INTERN MED, V171, P460, DOI 10.1001/archinternmed.2010.540

Bynum JPW, 2004, J AM GERIATR SOC, V52, P187, DOI 10.1111/j.1532-5415.2004.52054.x

California Health Advocates, UND MED ANN WELLB VI

Center for Health Systems Research and Analysis University of Wisconsin, ACT ASS PACE ENR CHA

Cigolle CT, 2011, J GEN INTERN MED, V26, P272, DOI 10.1007/s11606-010-1510-y

Counsell SR, 2007, JAMA-J AM MED ASSOC, V298, P2623, DOI 10.1001/jama.298.22.2623

Eng C, 1997, J AM GERIATR SOC, V45, P223, DOI 10.1111/j.1532-5415.1997.tb04513.x

Fried LP, 2008, J AM GERIATR SOC, V56, P1791, DOI 10.1111/j.1532-5415.2008.01939.x

Institute of Medicine of the National Academies, CHALL SUCC RED HLTH

Jagosh J, 2012, MILBANK Q, V90, P311, DOI 10.1111/j.1468-0009.2012.00665.x

Madon T, 2007, SCIENCE, V318, P1728, DOI 10.1126/science.1150009

Selby JV, 2012, JAMA-J AM MED ASSOC, V307, P1583, DOI 10.1001/jama.2012.500

Stange KC, 2010, J GEN INTERN MED, V25, P601, DOI 10.1007/s11606-010-1291-3

UCSF Department of Medicine Quality and Safety Programs, SYST INN QUAL IMPR P

NR 17

TC 4

Z9 4

U1 0

U2 5

PU SPRINGER

PI NEW YORK

PA 233 SPRING ST, NEW YORK, NY 10013 USA

SN 0884-8734

EI 1525-1497

J9 J GEN INTERN MED

JI J. Gen. Intern. Med.

PD JUN

PY 2014

VL 29

IS 6

BP 911

EP 914

DI 10.1007/s11606-013-2749-x

PG 4

WC Health Care Sciences & Services; Medicine, General & Internal

WE Science Citation Index Expanded (SCI-EXPANDED); Social Science Citation Index (SSCI)

SC Health Care Sciences & Services; General & Internal Medicine

GA AK1VS

UT WOS:000338206600020

PM 24557512

OA Green Published

DA 2024-08-08

ER

PT J

AU Zamir, D

Zamir, M

Reitblat, T

Zeev, W

Polishchuk, I

AF Zamir, Doron

Zamir, Mariana

Reitblat, Tatiana

Zeev, Weiler

Polishchuk, Ilia

TI Readmissions to hospital within 30 days of discharge from the internal

medicine wards in southern Israel

SO EUROPEAN JOURNAL OF INTERNAL MEDICINE

LA English

DT Article

DE early rehospitalization; risk factors; veteran Israelis

ID CONGESTIVE-HEART-FAILURE; EARLY REHOSPITALIZATION; GERIATRIC-PATIENTS

AB Introduction: Hospital readmission within a short time of discharge is a common phenomenon in internal medicine. Although there are a few reports of intensive care and surgical readmission rates there is almost no information available on the readmission rates of internal medicine patients.

Aims: To explore the medical patient readmission rate within 30 days of discharge from a medical center in southern Israel, and determine how it may be related to age, gender, diagnoses and other factors.

Methods: Chart review of all 2469 patients admitted to the departments of internal medicine of Barzilai Health Center between May 1st and July 15th 2002. A randomized control group of 87 patients was gathered from patients admitted to hospital during the study period but who were not readmitted within 30 days of discharge.

Results: 124 patients (5%) were readmitted. They were significantly older than controls (mean age 68.7 +/- 14.8 vs. 59.3 +/- 16.3 years), (p<0.0003), 21% being over 80 years of age (compared with 9% of controls, p<0.05) and had a lower average Functional Independence Measure (FIM). The index hospitalization of readmitted patients was significantly longer than the hospitalization of control patients (6.1 +/- 5.5 vs. 4.1 +/- 3.8 days), (p<0.04). Most patients (85%) were readmitted for the same medical reason that prompted the original hospitalization. The 15% readmitted for other reasons were older (74.9 +/- 12.4 vs. 67.6 +/- 15.0 years) (p<0.001) and had a lower Functional Independence Measure (mean FIM 4.6 +/- 2.2 vs. 5.7 +/- 1.9), (p<0.05). New immigrants (mean age 71.1 +/- 12.9 years) had significantly lower readmission rate compared to the general population (3.2% vs. 4.98%), (p<0.05). Veteran Israelis, on the other hand, had a higher readmission rate than the general population (6.8% vs. 4.98%) (p<0.001).

Conclusion: Only 5% of patients required readmission. The original hospitalization period of readmitted patients was significantly longer than controls and most readmissions were for the original medical problem for which they were readmitted originally or because of a low functional status. Hospitalized new immigrants although being older in age are less likely to require readmission, while veteran Israelis are more likely to be readmitted. (C) 2005 European Federation of Internal Medicine. Published by Elsevier B.V. All rights reserved.

C1 Barzilai Govt Hosp, Dept Internal Med D, IL-78306 Ashqelon, Israel.

Barzilai Govt Hosp, Dept Internal Med D, Ashqelon, Israel.

Barzilai Govt Hosp, Resp Inst, Ashqelon, Israel.

Ben Gurion Univ Negev, Fac Hlth Sci, Beer Sheva, Israel.

C3 Ben Gurion University; Barzilai Medical Center; Ben Gurion University;

Barzilai Medical Center; Ben Gurion University; Barzilai Medical Center;

Ben Gurion University

RP Zamir, D (corresponding author), Barzilai Govt Hosp, Dept Internal Med D, IL-78306 Ashqelon, Israel.

EM ZAMIR@BAPZI.HEALTH.GOV.IL

OI Reitblat, Tatiana/0000-0002-7197-641X

CR BOYDELL KM, 1991, CAN J PSYCHIAT, V36, P743

Brook U., 1999, Harefuah, V136, P931

*DEP RES INF, 2002, STAT REP BARZ MED CT

Di Iorio A, 1998, AGING CLIN EXP RES, V10, P339, DOI 10.1007/BF03339797

GRANGER CV, 1990, ARCH PHYS MED REHAB, V71, P870

GREEN JH, 1988, HOSP COMMUNITY PSYCH, V39, P963

HARRISON ML, 1995, CAN MED ASSOC J, V153, P745

JOYCE PR, 1985, PSYCHOL MED, P521

Martín RB, 2000, REV CLIN ESP, V200, P252

MARTINEZ JLA, 2001, AN MED INTERN, V18, P248

NIKOLAUS T, 1992, DEUT MED WOCHENSCHR, V117, P403, DOI 10.1055/s-2008-1062325

Philbin EF, 1997, AM J MANAG C, V3, P1285

RICH MW, 1988, AM J PUBLIC HEALTH, V78, P680, DOI 10.2105/AJPH.78.6.680

Scheier MF, 1999, ARCH INTERN MED, V159, P829, DOI 10.1001/archinte.159.8.829

Schwartzberg J G, 1982, Home Health Care Serv Q, V3, P25

Spisak V, 2000, Vnitr Lek, V46, P452

NR 16

TC 16

Z9 21

U1 1

U2 4

PU ELSEVIER SCIENCE BV

PI AMSTERDAM

PA PO BOX 211, 1000 AE AMSTERDAM, NETHERLANDS

SN 0953-6205

J9 EUR J INTERN MED

JI Eur. J. Intern. Med.

PD JAN

PY 2006

VL 17

IS 1

BP 20

EP 23

DI 10.1016/j.ejim.2005.10.004

PG 4

WC Medicine, General & Internal

WE Science Citation Index Expanded (SCI-EXPANDED)

SC General & Internal Medicine

GA 117EC

UT WOS:000242854500004

PM 16378880

DA 2024-08-08

ER

PT J

AU Schwendimann, R

Bühler, H

De Geest, S

Milisen, K

AF Schwendimann, Rene

Buehler, Hugo

De Geest, Sabina

Milisen, Koen

TI Characteristics of Hospital Inpatient Falls across Clinical Departments

SO GERONTOLOGY

LA English

DT Article

DE Inpatient falls; Hospitals; Characteristics of hospital falls; Hospital

departments

ID PREDICTIVE MODEL; RISK-FACTORS; PATIENT; PREVENTION

AB Background: Hospital inpatient falls are common and may lead to injuries and prolonged hospitalization. Although hospital studies have reported overall fall rates and injuries associated with falls, few have addressed population characteristics and circumstances of falls across clinical departments within a hospital setting. Objective: To determine inpatient fall rates in an urban public hospital and to explore associated characteristics across clinical departments. Methods: The study was conducted in a 300-bed urban public hospital in Switzerland from 1999 to 2003. Patient data and data from the hospital's standardized fall reporting system on hospital inpatients' first falls, along with associated characteristics, across the departments of internal medicine, geriatrics and surgery, were analyzed. Descriptive statistics and statistical tests: chi(2) and ANOVA tests with multiple comparisons tests (post-hoc analysis) were used. Results: Over this 5-year period, 34,972 patients were hospitalized (female 53.6%; mean age 67.3 +/- 19.3 years; mean length of stay 11.9 +/- 13.2 days) including 2,512 patients (7.5%) who experienced at least one fall during their hospitalization (geriatrics 24.8%; internal medicine 8.8%; surgery 1.9%). The fall rates per 1,000 patient (adjusted for age) days differed significantly between all of the departments (geriatrics 10.7; internal medicine 9.6; surgery: 3.2) (p < 0.001). Overall, 30.1% of the patients who fell experienced minor injuries and 5.1% major injuries. In geriatrics, fall-related circumstances such as transferring were more common (40.4%) than in medicine (33%) or surgery (30.4%) (p < 0.001), whereas falling out of bed was rarer (16.4%) (surgery 27.1%; internal medicine 20.5%) (p < 0.001). In addition, the prevalence of risk factors among patients who fell varied significantly among clinical departments, except for impaired cognition and narcotic use. Conclusion: In the hospital studied, inpatient falls are significantly more common in departments of geriatrics and internal medicine than in surgical departments. Fall rates, related injuries and circumstances of inpatient falls varied significantly among clinical departments, probably due to differences in patient characteristics. When monitoring falls, hospitals should therefore consider differences in characteristics associated with patient falls across clinical departments. High priorities should be allocated in view of identifying patients at risk of falling and implementing fall prevention strategies and interventions. Copyright (C) 2008 S. Karger AG, Basel

C1 [Schwendimann, Rene] Univ Basel, Fak Med, Inst Pflegewissensch, CH-4056 Basel, Switzerland.

[Schwendimann, Rene; Buehler, Hugo] Stadtspital Waid, Zurich, Switzerland.

[De Geest, Sabina; Milisen, Koen] Katholieke Univ Leuven, Ctr Hlth Serv & Nursing Res, Louvain, Belgium.

[Milisen, Koen] Univ Hosp Leuven, Dept Geriatr Med, Louvain, Belgium.

C3 University of Basel; KU Leuven; KU Leuven; University Hospital Leuven

RP Schwendimann, R (corresponding author), Univ Basel, Fak Med, Inst Pflegewissensch, Bernoullistr 28, CH-4056 Basel, Switzerland.

EM rene.schwendimann@unibas.ch

RI De Geest, Sabina M/F-7724-2010

OI DE GEEST, SABINA/0000-0001-6596-7237; Milisen, Koen/0000-0001-9230-1246

CR Aisen M L, 1994, J Am Paraplegia Soc, V17, P179

Alcée D, 2000, J NURS CARE QUAL, V14, P43, DOI 10.1097/00001786-200004000-00006

Ash K L, 1998, J Gerontol Nurs, V24, P7

Enloe M., 2005, Journal of Patient Safety, V1, P208, DOI DOI 10.1097/01.jps.0000195776.91068.93

GAEBLER S, 1993, J ADV NURS, V18, P1895, DOI 10.1046/j.1365-2648.1993.18121895.x

Goodwin M B, 1993, Aust Clin Rev, V13, P141

Halfon P, 2001, J CLIN EPIDEMIOL, V54, P1258, DOI 10.1016/S0895-4356(01)00406-1

Heinze C, 2004, SCHWESTER PFLEGER, V43, P46

HENDRICH A, 1995, APPL NURS RES, V8, P129, DOI 10.1016/S0897-1897(95)80592-3

Hitcho EB, 2004, J GEN INTERN MED, V19, P732, DOI 10.1111/j.1525-1497.2004.30387.x

HOLDENERMASCHER.E, 1999, SCHWEIZ MED WOCHENSC, V129, pS12

Kerzman H, 2004, J ADV NURS, V47, P223, DOI 10.1111/j.1365-2648.2004.03080.x

Krause Tom, 2005, Pflege, V18, P39, DOI 10.1024/1012-5302.18.1.39

MORGAN VR, 1985, AM J PUBLIC HEALTH, V75, P775, DOI 10.2105/AJPH.75.7.775

Morse J M, 1988, QRB Qual Rev Bull, V14, P369

MORSE JM, 1987, GERONTOLOGIST, V27, P516, DOI 10.1093/geront/27.4.516

Myers Helen, 2003, Int J Nurs Pract, V9, P223, DOI 10.1046/j.1440-172X.2003.00430.x

Nadkarni JB, 2005, GERONTOLOGY, V51, P329, DOI 10.1159/000086370

Oliver D, 2004, AGE AGEING, V33, P122, DOI 10.1093/ageing/afh017

ROHDE JM, 1990, INFECT CONT HOSP EP, V11, P521

Sari ABA, 2007, BMJ-BRIT MED J, V334, P79, DOI 10.1136/bmj.39031.507153.AE

Schwendimann R, 1998, Pflege, V11, P335

SEHESTED P, 1977, GERIATRICS, V32, P101

SPECHTLEIBLE N, 2002, EUR J GERIATR, V4, P131

SUTTON JC, 1994, BRIT J CLIN PRACT, V48, P63

Tutuarima JA, 1997, STROKE, V28, P297, DOI 10.1161/01.STR.28.2.297

Vassallo M, 2000, GERONTOLOGY, V46, P158, DOI 10.1159/000022152

Vassallo M, 2002, GERONTOLOGY, V48, P147, DOI 10.1159/000052833

von Renteln-Kruse W, 2004, Z GERONTOL GERIATR, V37, P9, DOI 10.1007/s00391-004-0204-7

NR 29

TC 116

Z9 126

U1 0

U2 13

PU KARGER

PI BASEL

PA ALLSCHWILERSTRASSE 10, CH-4009 BASEL, SWITZERLAND

SN 0304-324X

EI 1423-0003

J9 GERONTOLOGY

JI Gerontology

PY 2008

VL 54

IS 6

BP 342

EP 348

DI 10.1159/000129954

PG 7

WC Geriatrics & Gerontology

WE Science Citation Index Expanded (SCI-EXPANDED); Social Science Citation Index (SSCI)

SC Geriatrics & Gerontology

GA 372HE

UT WOS:000260891900002

PM 18460867

DA 2024-08-08

ER

PT J

AU Strom, C

Mollerup, TK

Kromberg, LS

Rasmussen, LS

Schmidt, TA

AF Strom, Camilla

Mollerup, Talie Khadem

Kromberg, Laurits Schou

Rasmussen, Lars Simon

Schmidt, Thomas Andersen

TI Hospitalisation in an emergency department short-stay unit compared to

an internal medicine department is associated with fewer complications

in older patients - an observational study

SO SCANDINAVIAN JOURNAL OF TRAUMA RESUSCITATION & EMERGENCY MEDICINE

LA English

DT Article

DE Emergency department short-stay units; Adverse events; Geriatric

emergency medicine; Elderly patients; Accelerated care; Alternative

hospitalisation strategies

ID PREVENTABLE ADVERSE EVENTS; ELDERLY-PATIENTS; IATROGENIC ILLNESS;

COLORECTAL SURGERY; CARE; ADMISSIONS; OUTCOMES

AB Background: Older patients are at particular risk of experiencing adverse events during hospitalisation.

Objective: To compare the frequencies and types of adverse events during hospitalisation in older persons acutely admitted to either an Emergency Department Short-stay Unit (SSU) or an Internal Medicine Department (IMD).

Methods: Observational study evaluating adverse events during hospitalisation in non-emergent, age-matched, internal medicine patients >= 75 years, acutely admitted to either the SSU or the IMD at Holbaek Hospital, Denmark, from January to August, 2014. Medical records were reviewed by independent assessors to detect adverse events according to predefined criteria. The primary outcome was the proportion of patients with an adverse event during and within 30 days after hospitalisation. Secondary outcomes included 90-day mortality, subtypes of adverse events, and timing of adverse events. Adjusted analyses were conducted to correct for potential confounders.

Results: Four-hundred-fifty patients, 225 patients in each group, were included. Adverse events were found in 67 (30%) patients in the SSU-group and 90 (40%) patients in the IMD group (Odds Ratio (OR) 0.64 (95% Confidence Interval (95% CI) 0.43-0.94, p = 0.02). The result was unchanged in an analysis adjusted for age, Charlson Comorbidity score, and sex. We found no significant difference in 90-day mortality (OR 0.75, 95% CI 0.41-1.38, p = 0.36). The most common adverse events were transfer during hospitalisation, unplanned readmission, and nosocomial infection.

Conclusions: Adverse events of hospitalisation were significantly less common in older patients acutely admitted to an Emergency Department Short-stay Unit as compared to admission to an Internal Medicine Department.

C1 [Strom, Camilla; Kromberg, Laurits Schou; Schmidt, Thomas Andersen] Univ Copenhagen, Holbaek Hosp, Dept Emergency Med, Holbaek, Denmark.

[Rasmussen, Lars Simon] Univ Copenhagen, Dept Anaesthesia, Ctr Head & Orthopaed, Rigshosp, Copenhagen, Denmark.

[Rasmussen, Lars Simon; Schmidt, Thomas Andersen] Univ Copenhagen, Inst Clin Med, Fac Hlth & Med Sci, Copenhagen, Denmark.

[Mollerup, Talie Khadem] Univ Copenhagen, Holbaek Hosp, Dept Anaesthesiol, Holbaek, Denmark.

C3 University of Copenhagen; Holbaek Hospital; Rigshospitalet; University

of Copenhagen; University of Copenhagen; Holbaek Hospital; University of

Copenhagen

RP Strom, C (corresponding author), Univ Copenhagen, Holbaek Hosp, Dept Emergency Med, Holbaek, Denmark.

EM cstr@regionsjaelland.dk

RI Schmidt, Thomas A/GYU-7678-2022

OI Rasmussen, Lars/0000-0002-7480-3004

FU Region Zealand; University of Copenhagen; Trygfonden

FX CS has received research grants from Region Zealand and the University

of Copenhagen in relation to this work. LSR received funding from

Trygfonden for other projects. Both CS and LSR are independent from

funders. The funders had no role in the conception of the trial, the

design, data collection, data analyses, data interpretation, or the

preparation of the manuscript.

CR Aminzadeh F, 2002, ANN EMERG MED, V39, P238, DOI 10.1067/mem.2002.121523

Bagnall NM, 2014, COLORECTAL DIS, V16, P947, DOI 10.1111/codi.12718

Baker GR, 2004, CAN MED ASSOC J, V170, P1678, DOI 10.1503/cmaj.1040498

BECKER PM, 1987, JAMA-J AM MED ASSOC, V257, P2313, DOI 10.1001/jama.257.17.2313

BRENNAN P, 1992, BMJ-BRIT MED J, V304, P1491, DOI 10.1136/bmj.304.6840.1491

Brennan TA, 2004, QUAL SAF HEALTH CARE, V13, P145, DOI 10.1136/qshc.2002.003822

Cowling TE, 2014, J ROY SOC MED, V107, P432, DOI 10.1177/0141076814542669

Daly S, 2003, MED J AUSTRALIA, V178, P559, DOI 10.5694/j.1326-5377.2003.tb05359.x

Damiani G, 2001, MED SCI MONITOR, V17, pSR15

de Vries EN, 2008, QUAL SAF HEALTH CARE, V17, P216, DOI 10.1136/qshc.2007.023622

Downing H, 2008, CLIN MED, V8, P18, DOI 10.7861/clinmedicine.8-1-18

Galipeau J, 2015, ACAD EMERG MED, V22, P893, DOI 10.1111/acem.12730

GASPOZ JM, 1991, AM J CARDIOL, V68, P145, DOI 10.1016/0002-9149(91)90734-3

Jibrin Ismaila, 2008, Crit Pathw Cardiol, V7, P35, DOI 10.1097/HPC.0b013e318163eb83

Khan MA, 2016, ANN ROY COLL SURG, V98, P29, DOI 10.1308/rcsann.2015.0036

LEFEVRE F, 1992, ARCH INTERN MED, V152, P2074, DOI 10.1001/archinte.152.10.2074

Long SJ, 2013, INT J QUAL HEALTH C, V25, P542, DOI 10.1093/intqhc/mzt056

Madeira S, 2007, EUR J INTERN MED, V18, P391, DOI 10.1016/j.ejim.2006.12.009

Michel P, 2004, BRIT MED J, V328, P199, DOI 10.1136/bmj.328.7433.199

Miller CD, 2010, ANN EMERG MED, V56, P209, DOI 10.1016/j.annemergmed.2010.04.009

Petersen DB, 2013, J HOSP ADM, V3, P17, DOI [10.5430/jha.v3n3p17, DOI 10.5430/JHA.V3N3P17]

Skriver Claus, 2011, Ugeskr Laeger, V173, P2490

Spanjersberg WR, 2011, COCHRANE DB SYST REV, DOI 10.1002/14651858.CD007635.pub2

STEEL K, 1981, NEW ENGL J MED, V304, P638, DOI 10.1056/NEJM198103123041104

Thomas EJ, 2000, BRIT MED J, V320, P741, DOI 10.1136/bmj.320.7237.741

Thomas EJ, 2000, MED CARE, V38, P261, DOI 10.1097/00005650-200003000-00003

Wilson RM, 1995, MED J AUSTRALIA, V163, P458, DOI 10.5694/j.1326-5377.1995.tb124691.x

NR 27

TC 11

Z9 12

U1 0

U2 4

PU BMC

PI LONDON

PA CAMPUS, 4 CRINAN ST, LONDON N1 9XW, ENGLAND

SN 1757-7241

J9 SCAND J TRAUMA RESUS

JI Scand. J. Trauma Resusc. Emerg. Med.

PD AUG 15

PY 2017

VL 25

AR 80

DI 10.1186/s13049-017-0422-9

PG 9

WC Emergency Medicine

WE Science Citation Index Expanded (SCI-EXPANDED)

SC Emergency Medicine

GA FE0WR

UT WOS:000407940900001

PM 28810888

OA gold, Green Published

DA 2024-08-08

ER

PT J

AU Draganov, I

Drndarevic, A

Kovacevic, M

Miljkovic, B

Vuksanovic, M

Jankovic, A

Kalaba, A

Kovacevic, SV

AF Draganov, Ivana

Drndarevic, Aneta

Kovacevic, Milena

Miljkovic, Branislava

Vuksanovic, Miljanka

Jankovic, Aleksandar

Kalaba, Ana

Kovacevic, Sandra vezmar

TI DRUG-RELATED PROBLEMS PRIOR TO HOSPITALIZATION ON INTERNAL MEDICINE

WARDS

SO ACTA POLONIAE PHARMACEUTICA

LA English

DT Article

DE drug -related problems; internal medicine; hospital admission

ID RISK-FACTORS; EVENTS; ADMISSIONS; PEOPLE

AB Drug-related hospitalizations pose a significant burden to the health-care system. The aim was to investigate the prevalence of drug-related problems (DRPs) and their association with hospital admissions in five internal medicine wards. The study included patients admitted to the nephrology, cardiology, gastroenterology, endocrinology, and geriatric wards. The Pharmaceutical Care Network Europe classification V9.1 was used for identifying DRPs. In total, 535 patients participated in the study. We identified 954 DRPs (range 1-7) in 80.7% of patients. Most DRPs were identified on the endocrinology, cardiology, and geriatric wards, and they were associated with the efficacy of treatment (71.4%), adverse drug events (10.2%), and unnecessary drug treatment (18.4%). DRPs were associated with the cause of hospitalization in 74.4% of patients on the nephrology ward, 60.1% and 60.6% of patients on the cardiology and endocrinology wards, respectively, whereas this number was lower on the geriatric and gastroenterology wards (26.9% and 8.9%, respectively). Suboptimal drug treatment due to medication omissions was often associated with the potential cause of hospital admission. Focusing on patients with specific diseases and DRPs, rather than reducing the number of medications in primary care, may be potentially rational in an attempt to reduce drug-related hospitalizations.

C1 [Draganov, Ivana; Drndarevic, Aneta; Kovacevic, Milena; Miljkovic, Branislava; Kovacevic, Sandra vezmar] Univ Belgrade, Fac Pharm, Dept Pharmacokinet & Clin Pharm, Belgrade, Serbia.

[Vuksanovic, Miljanka; Jankovic, Aleksandar] Univ Belgrade, Sch Med, Belgrade, Serbia.

[Vuksanovic, Miljanka] Univ Hosp Med Ctr Zvezdara, Dept Endocrinol Diabet & Metab Disorders, Belgrade, Serbia.

[Jankovic, Aleksandar] Univ Hosp Med Ctr Zvezdara, Dept Nephrol & Metab Disorders Dialysis, Belgrade, Serbia.

[Kalaba, Ana] Univ Hosp Med Ctr Zvezdara, Dept Gastroenterol & Hepatol, Belgrade, Serbia.

C3 University of Belgrade; University of Belgrade

RP Kovacevic, M (corresponding author), Univ Belgrade, Fac Pharm, Dept Pharmacokinet & Clin Pharm, Belgrade, Serbia.

EM milenak@pharmacy.bg.ac.rs

OI Kovacevic, Milena/0000-0003-2957-7965

FU Ministry of Science, Technological Development and Innovation, Republic

of Serbia [451-03-65/2024-03/200161, 451-03-66/2024-03/200161]

FX This research was funded by the Ministry of Science, Technological

Development and Innovation, Republic of Serbia, through a Grant

Agreement with the University of Belgrade Faculty of Pharmacy No:

451-03-65/2024-03/200161 and 451-03-66/2024-03/200161.

CR Al Hamid A, 2014, BRIT J CLIN PHARMACO, V78, P202, DOI 10.1111/bcp.12293

Alruqayb WS, 2021, DRUG SAFETY, V44, P1041, DOI 10.1007/s40264-021-01099-3

Ayalew Mohammed Biset, 2019, Bull Emerg Trauma, V7, P339, DOI 10.29252/beat-070401

Bekele F, 2021, PLOS ONE, V16, DOI 10.1371/journal.pone.0248575

Belayneh YM, 2018, BMC HEALTH SERV RES, V18, DOI 10.1186/s12913-018-3612-x

Blum MR, 2021, BMJ-BRIT MED J, V374, DOI 10.1136/bmj.n1585

Cardone KE, 2010, ADV CHRONIC KIDNEY D, V17, P404, DOI 10.1053/j.ackd.2010.06.004

Ernst F R, 2001, J Am Pharm Assoc (Wash), V41, P192

Formica D, 2018, EXPERT OPIN DRUG SAF, V17, P681, DOI 10.1080/14740338.2018.1491547

Gustafsson M, 2016, EUR J CLIN PHARMACOL, V72, P1143, DOI 10.1007/s00228-016-2084-3

HANLON JT, 1992, J CLIN EPIDEMIOL, V45, P1045, DOI 10.1016/0895-4356(92)90144-C

HEPLER CD, 1990, AM J HOSP PHARM, V47, P533, DOI 10.1093/ajhp/47.3.533

Howard RL, 2007, BRIT J CLIN PHARMACO, V63, P136, DOI 10.1111/j.1365-2125.2006.02698.x

Joseph JJ, 2022, CIRCULATION, V145, pE722, DOI 10.1161/CIR.0000000000001040

Kim Kyu, 2020, J Lipid Atheroscler, V9, P1, DOI 10.12997/jla.2020.9.1.1

Konuru V, 2019, J PHARM BIOALLIED SC, V11, P328, DOI 10.4103/jpbs.JPBS_35_18

Lavan AH, 2016, THER ADV DRUG SAF, V7, P11, DOI 10.1177/2042098615615472

Lea M, 2019, PLOS ONE, V14, DOI 10.1371/journal.pone.0220071

Lee GB, 2022, THER ADV DRUG SAF, V13, DOI 10.1177/20420986221100117

Leendertse AJ, 2008, ARCH INTERN MED, V168, P1890, DOI 10.1001/archinternmed.2008.3

Marcum ZA, 2012, J GERONTOL A-BIOL, V67, P867, DOI 10.1093/gerona/gls001

Mongkhon P, 2018, BMJ QUAL SAF, V27, P902, DOI 10.1136/bmjqs-2017-007453

Nebeker JR, 2004, ANN INTERN MED, V140, P795, DOI 10.7326/0003-4819-140-10-200405180-00009

Nivya K, 2015, SAUDI PHARM J, V23, P1, DOI 10.1016/j.jsps.2013.05.006

O'Mahony D, 2015, AGE AGEING, V44, P213, DOI 10.1093/ageing/afu145

Ocovská Z, 2023, FRONT PHARMACOL, V14, DOI 10.3389/fphar.2023.1088900

Ocovska Z, 2022, FRONT PHARMACOL, V13, DOI 10.3389/fphar.2022.899151

Oscanoa TJ, 2017, EUR J CLIN PHARMACOL, V73, P759, DOI 10.1007/s00228-017-2225-3

Pattanaik S, 2009, BRIT J CLIN PHARMACO, V67, P363, DOI 10.1111/j.1365-2125.2008.03346.x

Peterson Cecilia, 2017, Drugs Real World Outcomes, V4, P97, DOI 10.1007/s40801-017-0108-7

Peyriere H, 2003, ANN PHARMACOTHER, V37, P5, DOI 10.1345/aph.1C126

Pharmaceutical Care Network Europe (PCNE), 2022, Classification for Drug related problems v 9.1

Roller-Wirnsberger R, 2020, BRIT J CLIN PHARMACO, V86, P1904, DOI 10.1111/bcp.14074

Samoy LJ, 2006, PHARMACOTHERAPY, V26, P1578, DOI 10.1592/phco.26.11.1578

Shouqair TM, 2022, CUREUS J MED SCIENCE, V14, DOI 10.7759/cureus.24019

Singh H, 2011, J PHARMACOL PHARMACO, V2, P17, DOI 10.4103/0976-500X.77095

Somers A, 2010, J NUTR HEALTH AGING, V14, P477, DOI 10.1007/s12603-009-0237-0

Strauss MH, 2023, CARDIOVASC DRUG THER, V37, P757, DOI 10.1007/s10557-021-07248-1

Wallerstedt SM, 2022, BRIT J CLIN PHARMACO, V88, P541, DOI 10.1111/bcp.15012

WHO, 2019, Guidelines for atc classification and ddd assignment

World Health Organisation (WHO), 1966, Technical report series, V425

NR 41

TC 0

Z9 0

U1 0

U2 0

PU POLSKIE TOWARZYSTWO FARMACEUTYCZNE

PI WARSAW

PA DLUGA 16, 00-238 WARSAW, POLAND

SN 0001-6837

EI 2353-5288

J9 ACTA POL PHARM

JI ACTA POL. PHARM.

PD JAN-FEB

PY 2024

VL 81

IS 1

BP 145

EP 154

DI 10.32383/appdr/182840

PG 10

WC Pharmacology & Pharmacy

WE Science Citation Index Expanded (SCI-EXPANDED)

SC Pharmacology & Pharmacy

GA TU6G9

UT WOS:001243807500011

OA hybrid

DA 2024-08-08

ER

PT J

AU Mannucci, PM

Nobili, A

Pasina, L

AF Mannucci, Pier Mannuccio

Nobili, Alessandro

Pasina, Luca

CA REPOSI Collaborators

TI Polypharmacy in older people: lessons from 10years of experience with

the REPOSIregister

SO INTERNAL AND EMERGENCY MEDICINE

LA English

DT Review

DE Multimorbidity; Polypharmacy; Inappropriate prescription; Deprescribing;

Medication reconciliation

ID ADVERSE CLINICAL EVENTS; ELDERLY-PATIENTS; RISK-FACTORS; PROGNOSTIC

VALUE; AFTER-DISCHARGE; DRUG-USE; COHORT; MORTALITY; PREVALENCE;

GUIDELINES

AB As a consequence of population aging, we have witnessed in internal medicine hospital wards a progressive shift from a population of in-patients relatively young and mainly affected by a single ailment to one of ever older and more and more complex patients with multiple chronic diseases, followed as out-patients by many different specialists with poor integration andinevitably treated with multiple medications. Polypharmacy (defined as the chronic intake of five or more drugs) is associated with increased risks of drug-drug interactions and related adverse effects, prescription and intake errors, poor compliance, re-hospitalization and mortality. With this background, the Italian Society of Internal Medicine chose to start in 2008 a prospective register called REPOSI (REgistro POliterapie SIMI, Societa Italiana di Medicina Interna) in internal medicine and geriatric hospital wards. The country wide register is an ongoing observatory on multimorbidity and polypharmacy in the oldest old, with the goal to improve prescription appropriateness and, thus to avoid potentially inappropriate medications. The main findings of the register, that has accrued so far, 7005 older patients throughout a 10year period, are summarized herewith, with special emphasis on the main patterns of poor prescription appropriateness and related risks of adverse events.

C1 [Mannucci, Pier Mannuccio] Fdn IRCCS Ca Granda Osped Maggiore Policlin, Sci Direct, Via Francesco Sforza 35,Via Pace 9, I-20122 Milan, Italy.

[Nobili, Alessandro; Pasina, Luca] IRCCS Ist Ric Farmacol Mario Negri, Neurosci Dept, Via Giuseppe La Masa 19, I-20156 Milan, Italy.

C3 IRCCS Ca Granda Ospedale Maggiore Policlinico; Istituto di Ricerche

Farmacologiche Mario Negri IRCCS

RP Mannucci, PM (corresponding author), Fdn IRCCS Ca Granda Osped Maggiore Policlin, Sci Direct, Via Francesco Sforza 35,Via Pace 9, I-20122 Milan, Italy.

EM piermannuccio.mannucci@policlinico.mi.it

RI AGOSTI, PASQUALE/L-3148-2019; Boari, Benedetta/AAB-4792-2022; Rossi,

Paolo Dionigi/GRX-7747-2022; Succurro, Elena/K-4138-2016; Purrello,

Francesco/K-9721-2016; Colussi, GianLuca/I-2061-2012; Paoletti,

Giovanni/ABC-1135-2020; Cesari, Matteo/A-4649-2008; Landolfi,

Raffaele/AAL-4496-2020; Zaccherini, Giacomo/A-2368-2015; Manfredini,

Roberto/A-3471-2008; Basile, Giorgio/GLS-6497-2022; Pasina,

Luca/AAB-1877-2020; Mecocci, Patrizia/W-2116-2019; Peyvandi,

Flora/AAK-7437-2020; Catalano, Antonino/H-7109-2016; Nobili,

Alessandro/ABG-4516-2020; Mascherini, Gabriele/E-5773-2016; Fragapani,

Salvatore/KHV-3284-2024; Sáez, Pilar/AAD-4083-2020; Salzano,

Andrea/A-6392-2015; Corrao, Salvatore/AAQ-5768-2020; Liberale,

Luca/H-1560-2018; Sola, Daniele/CAJ-4366-2022; Alessandro,

Nobili/AAA-7512-2020; Rizzo, Maria Rosaria/AHH-3649-2022; Zanoli,

Luca/L-9309-2016; BALLESTRERO, ALBERTO/K-7546-2016; Ticinesi,

Andrea/K-5017-2016; Ottonello, Luciano Carlo/I-3250-2018; Marra, Alberto

Maria/K-5431-2016; Bobbio, Emanuele/K-7313-2016; Lenti, Marco

Vincenzo/F-9044-2018; meschi, tiziana/K-5032-2016; CAPPELLINI, MARIA

DOMENICA/B-4475-2017; Piro, Salvatore/H-9353-2012; Tettamanti,

Mauro/U-8925-2017; brucato, antonio/C-5983-2019; Mirijello,

Antonio/C-4103-2017

OI AGOSTI, PASQUALE/0000-0002-1239-775X; Boari,

Benedetta/0000-0002-9015-7858; Rossi, Paolo Dionigi/0000-0002-1202-5454;

Succurro, Elena/0000-0003-2578-692X; Purrello,

Francesco/0000-0003-3313-8543; Colussi, GianLuca/0000-0003-1673-540X;

Paoletti, Giovanni/0000-0003-3953-9225; Cesari,

Matteo/0000-0002-0348-3664; Landolfi, Raffaele/0000-0002-7913-8576;

Zaccherini, Giacomo/0000-0002-3219-6963; Manfredini,

Roberto/0000-0002-8364-2601; Basile, Giorgio/0000-0002-0040-8618;

Pasina, Luca/0000-0002-4777-1350; Mecocci, Patrizia/0000-0003-0729-5246;

Peyvandi, Flora/0000-0001-7423-9864; Catalano,

Antonino/0000-0003-3890-2299; Nobili, Alessandro/0000-0002-7813-0572;

Mascherini, Gabriele/0000-0002-8842-0354; Fragapani,

Salvatore/0009-0007-1692-4020; Sáez, Pilar/0000-0002-9467-7746; Salzano,

Andrea/0000-0003-2352-2103; Corrao, Salvatore/0000-0001-5621-1374;

Liberale, Luca/0000-0003-1472-7975; Sola, Daniele/0000-0001-7863-8192;

Rizzo, Maria Rosaria/0000-0002-1023-4260; Zanoli,

Luca/0000-0003-1678-3778; CASTELLINO, Pietro/0000-0002-9014-2007;

Arcopinto, Michele/0000-0002-7033-2145; MALATINO, Lorenzo

Salvatore/0000-0003-2944-2729; BALLESTRERO, ALBERTO/0000-0002-2601-7711;

Ticinesi, Andrea/0000-0001-9171-8592; Ottonello, Luciano

Carlo/0000-0001-7431-3841; Poyato Borrego, Manuel/0000-0002-2749-355X;

maresca, andrea maria/0000-0001-7896-2021; FRANCHI,

CARLOTTA/0000-0001-6802-8710; Zucchelli, Alberto/0000-0001-5992-8506;

Mengoli, Caterina/0000-0002-2253-7795; Marra, Alberto

Maria/0000-0002-9679-3003; Bobbio, Emanuele/0000-0002-8287-2448;

ZANETTI, MICHELA/0000-0002-2634-6363; Ruggiero,

Carmelinda/0000-0002-1245-1963; Lenti, Marco

Vincenzo/0000-0002-6654-4911; meschi, tiziana/0000-0002-7538-6863;

Periti, Giulia/0009-0005-1265-4583; Emmi, Giacomo/0000-0001-9575-8321;

Valeriani, Emanuele/0000-0001-9729-3214; Ruiz Cantero,

Alberto/0000-0002-7121-6867; Sirico, Domenico/0000-0002-6444-2292;

Firinu, Davide/0000-0002-5768-391X; Cattaneo, Marco/0000-0002-7343-4534;

CAPPELLINI, MARIA DOMENICA/0000-0001-8676-6864; Zuccala,

Giuseppe/0000-0002-2567-2220; Piro, Salvatore/0000-0002-1781-0902; De

Giorgi, Alfredo/0000-0002-0903-7825; Tettamanti,

Mauro/0000-0001-7345-0887; marengoni, alessandra/0000-0002-4851-1825;

COLANGELO, LUCIANO/0000-0002-5835-9310; Marra,

Alessio/0000-0002-8220-3113; Trotta, Lucia/0000-0002-4130-5519; GIRELLI,

Domenico/0000-0001-9684-1899; brucato, antonio/0000-0002-7566-5600;

BIOLO, GIANNI/0000-0002-6397-1598; Martino, Giuseppe

Pio/0000-0002-4906-0472; Mirijello, Antonio/0000-0003-3932-3803;

Damanti, Sarah/0000-0002-2399-3688; Capeci, William/0000-0002-0884-158X

CR Ardoino I, 2017, BRIT J CLIN PHARMACO, V83, P2528, DOI 10.1111/bcp.13355

Bellelli G, 2015, EUR J INTERN MED, V26, P696, DOI 10.1016/j.ejim.2015.08.006

Cerreta F, 2012, NEW ENGL J MED, V367, P1972, DOI 10.1056/NEJMp1209034

Cesari M, 2018, EUR J INTERN MED, V56, P11, DOI 10.1016/j.ejim.2018.06.001

Corrao S, 2018, EUR J INTERN MED, V54, P53, DOI 10.1016/j.ejim.2018.04.012

Corrao S, 2014, EUR J INTERN MED, V25, P617, DOI 10.1016/j.ejim.2014.06.027

Corsi Nicole, 2018, Eur J Intern Med, V55, P35, DOI 10.1016/j.ejim.2018.05.031

Damanti S, 2017, EUR J INTERN MED, V44, pE18, DOI 10.1016/j.ejim.2017.06.023

De La Higuera L, 2014, INTERN EMERG MED, V9, P735, DOI 10.1007/s11739-013-1028-5

Franchi C, 2017, PHARMACOEPIDEM DR S, V26, P1534, DOI 10.1002/pds.4330

Franchi C, 2016, DRUG AGING, V33, P53, DOI 10.1007/s40266-015-0337-y

Franchi C, 2013, EUR J INTERN MED, V24, P45, DOI 10.1016/j.ejim.2012.10.005

Galazzi A, 2016, INT J CLIN PHARM-NET, V38, P454, DOI 10.1007/s11096-016-0279-4

Gallo P, 2018, EUR J INTERN MED, V54, P60, DOI 10.1016/j.ejim.2018.04.001

Heiat A, 2002, ARCH INTERN MED, V162, P1682, DOI 10.1001/archinte.162.15.1682

Lau DT, 2005, ARCH INTERN MED, V165, P68, DOI 10.1001/archinte.165.1.68

Lazarou J, 1998, JAMA-J AM MED ASSOC, V279, P1200, DOI 10.1001/jama.279.15.1200

Mannucci PM, 2014, INTERN EMERG MED, V9, P723, DOI 10.1007/s11739-014-1124-1

Mannucci PM, 2012, EUR J INTERN MED, V23, P479, DOI 10.1016/j.ejim.2012.06.008

Marcucci M, 2010, EUR J INTERN MED, V21, P516, DOI 10.1016/j.ejim.2010.07.014

Marcucci M, 2017, J GERONTOL A-BIOL, V72, P395, DOI 10.1093/gerona/glw188

Marcucci M, 2013, INTERN EMERG MED, V8, P509, DOI 10.1007/s11739-013-0944-8

Marengoni A, 2013, GERONTOLOGY, V59, P307, DOI 10.1159/000346353

Marengoni A, 2012, INT PSYCHOGERIATR, V24, P606, DOI 10.1017/S1041610211002353

Marengoni A, 2010, REJUV RES, V13, P469, DOI 10.1089/rej.2009.1002

Marengoni A, 2015, INTERN EMERG MED, V10, P305, DOI 10.1007/s11739-014-1131-2

Marengoni A, 2013, J GERONTOL A-BIOL, V68, P419, DOI 10.1093/gerona/gls181

Marengoni A, 2011, AGEING RES REV, V10, P430, DOI 10.1016/j.arr.2011.03.003

Nelson KM, 1996, PHARMACOTHERAPY, V16, P701

Nobili A, 2011, EUR J INTERN MED, V22, P597, DOI 10.1016/j.ejim.2011.08.029

Nobili A, 2011, EUR J CLIN PHARMACOL, V67, P507, DOI 10.1007/s00228-010-0977-0

Opondo D, 2012, PLOS ONE, V7, DOI 10.1371/journal.pone.0043617

Paciullo F, 2018, DRUG AGING, V35, P365, DOI 10.1007/s40266-018-0532-8

Pasina L, 2014, EUR J CLIN PHARMACOL, V70, P1495, DOI 10.1007/s00228-014-1752-4

Pasina L, 2014, J CLIN PHARM THER, V39, P511, DOI 10.1111/jcpt.12178

Pasina L, 2011, EUR J INTERN MED, V22, P205, DOI 10.1016/j.ejim.2010.11.009

Pasina L, 2018, ARCH GERONTOL GERIAT, V74, P169, DOI 10.1016/j.archger.2017.10.016

Pasina L, 2017, EUR J INTERN MED, V38, pE11, DOI 10.1016/j.ejim.2016.10.016

Pasina L, 2016, DRUG AGING, V33, P647, DOI 10.1007/s40266-016-0395-9

Pasina L, 2013, PHARMACOEPIDEM DR S, V22, P1054, DOI 10.1002/pds.3510

Pasina L, 2013, DRUG AGING, V30, P103, DOI 10.1007/s40266-012-0044-x

Proietti M, 2016, CLIN RES CARDIOL, V105, P912, DOI 10.1007/s00392-016-0999-4

Reeve E, 2014, BRIT J CLIN PHARMACO, V78, P738, DOI 10.1111/bcp.12386

Renaudin P, 2016, BRIT J CLIN PHARMACO, V82, P1660, DOI 10.1111/bcp.13085

Riva E, 2017, ARCH GERONTOL GERIAT, V69, P21, DOI 10.1016/j.archger.2016.11.005

Rossio R, 2015, EUR J INTERN MED, V26, P330, DOI 10.1016/j.ejim.2015.04.002

Santalucia P, 2015, EUR J INTERN MED, V26, P483, DOI 10.1016/j.ejim.2015.07.006

Scott IA, 2015, JAMA INTERN MED, V175, P827, DOI 10.1001/jamainternmed.2015.0324

Tinetti ME, 2004, NEW ENGL J MED, V351, P2870, DOI 10.1056/NEJMsb042458

NR 49

TC 39

Z9 41

U1 0

U2 12

PU SPRINGER-VERLAG ITALIA SRL

PI MILAN

PA VIA DECEMBRIO, 28, MILAN, 20137, ITALY

SN 1828-0447

EI 1970-9366

J9 INTERN EMERG MED

JI Intern. Emerg. Med.

PD DEC

PY 2018

VL 13

IS 8

BP 1191

EP 1200

DI 10.1007/s11739-018-1941-8

PG 10

WC Medicine, General & Internal

WE Science Citation Index Expanded (SCI-EXPANDED)

SC General & Internal Medicine

GA HD0EM

UT WOS:000452180500008

PM 30171585

OA Green Submitted, Green Published

DA 2024-08-08

ER

PT J

AU Díez-Manglano, J

Espuelas-Monge, M

Gomes-Martín, J

Lerín-Sánchez, J

Odriozola-Grijalba, M

Piqueras-Serrano, C

Sánchez-Rubio-Lezcano, P

Zamora-Mur, A

AF Diez-Manglano, Jesus

Espuelas-Monge, Maria

Gomes-Martin, Javier

Lerin-Sanchez, Javier

Odriozola-Grijalba, Monica

Piqueras-Serrano, Carmen

Sanchez-Rubio-Lezcano, Pablo

Zamora-Mur, Alfredo

TI Reliability of three questions for the screening of advanced cognitive

impairment in polypathological patients

SO GERIATRICS & GERONTOLOGY INTERNATIONAL

LA English

DT Article

DE dementia; polypathological patient; screening; sensitivity; specificity

ID MENTAL STATUS QUESTIONNAIRE; INTERNAL-MEDICINE; VALIDATION; DEMENTIA;

DEPARTMENTS; FEATURES; INDEX

AB AimTo determine whether answering three questions erroneously (what is the date today?, when were you born? and how old are you?) allows for the detection of advanced cognitive impairment in polypathological patients.

MethodsA cross-sectional study of patients admitted to the internal medicine and geriatrics departments was carried out. Advanced cognitive impairment was diagnosed when patients made 7-10 errors in the Short Portable Mental Status Questionnaire.

ResultsWe included 441 polypathological patients, 330 of them from the internal medicine departments and 111 from the geriatrics department. Their mean age was 80.8 years (8.9 years). Of them, 141 (32.0%) answered one question incorrectly, 58 (13.1%) answered two and 89 (20.2%) answered all three questions incorrectly. The prevalence of advanced cognitive impairment was 27.7%. The sensitivity, specificity, and positive and negative predictive values of the test were 0.705, 0.991, 0.966 and 0.898, respectively. The accuracy of the test was 0.912. The area under the receiver operating characteristic curve was 0.947, 95% CI 0.923-0.970.

ConclusionsThe three questions test has very good specificity and positive predictive value, and it can be used for the screening of advanced cognitive impairment in polypathological patients. Geriatr Gerontol Int 2018; 18: 441-447.

C1 [Diez-Manglano, Jesus; Gomes-Martin, Javier] Hosp Royo Villanova, Dept Internal Med, Zaragoza, Spain.

[Diez-Manglano, Jesus; Espuelas-Monge, Maria; Odriozola-Grijalba, Monica; Sanchez-Rubio-Lezcano, Pablo] Aragon Hlth Sci Inst, Res Grp Comorbid & Polypathol Aragon, Zaragoza, Spain.

[Diez-Manglano, Jesus] Univ Zaragoza, Sch Med, Dept Med Dermatol & Psychiat, Zaragoza, Spain.

[Espuelas-Monge, Maria; Odriozola-Grijalba, Monica] Hosp Miguel Servet, Dept Internal Med, Zaragoza, Spain.

[Lerin-Sanchez, Javier] Hosp Obispo Polanco, Dept Internal Med, Teruel, Spain.

[Piqueras-Serrano, Carmen] Hosp Alcaniz, Dept Internal Med, Alcaniz, Spain.

[Sanchez-Rubio-Lezcano, Pablo] Hosp Barbastro, Dept Internal Med, Huesca, Spain.

[Zamora-Mur, Alfredo] Hosp Barbastro, Dept Geriatr, Huesca, Spain.

C3 University of Zaragoza; Miguel Servet University Hospital

RP Díez-Manglano, J (corresponding author), Duquesa Villahermosa 163,8 D, Zaragoza 50009, Spain.

EM jdiez@aragon.es

RI Díez-Manglano, J/AAC-3198-2020

OI Díez-Manglano, J/0000-0002-3132-2171; Garces Horna,

Vanesa/0000-0002-3485-0789; Zamora, Alfredo/0000-0001-8791-3838

CR Alarcon M, 1998, Rev Esp Geriatr Gerontol, V33, P178

[Anonymous], 1986, International J. Geriatr. Psychiatry, DOI [DOI 10.1002/GPS.930010209, 10.1002/gps.930010209]

Bejarano Tello E, 2011, REV CLIN ESP, V211, P194

Bernabeu-Wittel M, 2011, ARCH GERONTOL GERIAT, V53, P284, DOI 10.1016/j.archger.2010.12.006

Bernabeu-Wittel M, 2011, EUR J INTERN MED, V22, P311, DOI 10.1016/j.ejim.2010.11.012

Borson S, 2000, INT J GERIATR PSYCH, V15, P1021, DOI 10.1002/1099-1166(200011)15:11<1021::AID-GPS234>3.0.CO;2-6

Buschke H, 1999, NEUROLOGY, V52, P231, DOI 10.1212/WNL.52.2.231

CHARLSON ME, 1987, J CHRON DIS, V40, P373, DOI 10.1016/0021-9681(87)90171-8

de la Iglesia JM, 2001, MED CLIN-BARCELONA, V117, P129

Díez-Manglano J, 2015, INTERN EMERG MED, V10, P915, DOI 10.1007/s11739-015-1252-2

Díez-Manglano J, 2013, EUR J INTERN MED, V24, P767, DOI 10.1016/j.ejim.2013.07.010

FOLSTEIN MF, 1975, J PSYCHIAT RES, V12, P189, DOI 10.1016/0022-3956(75)90026-6

García-Morillo JS, 2005, MED CLIN-BARCELONA, V125, P5, DOI 10.1157/13076399

LAWTON MP, 1969, GERONTOLOGIST, V9, P179, DOI 10.1093/geront/9.3_Part_1.179

MAHONEY F I, 1965, Md State Med J, V14, P61

Moreno-Gaviño L, 2012, INT J GERONTOL, V6, P84, DOI 10.1016/j.ijge.2011.09.026

Ollero M, 2007, PROCESO ASISTENCIAL

PFEIFFER E, 1975, J AM GERIATR SOC, V23, P433, DOI 10.1111/j.1532-5415.1975.tb00927.x

Pilotto A, 2008, REJUV RES, V11, P151, DOI 10.1089/rej.2007.0569

Ramírez-Duque N, 2008, REV CLIN ESP, V208, P4, DOI 10.1157/13115000

REISBERG B, 1982, AM J PSYCHIAT, V139, P1136

REISBERG B, 1988, PSYCHOPHARMACOL BULL, V24, P653

Sager Mark A, 2006, WMJ, V105, P25

Tsoi KKF, 2015, JAMA INTERN MED, V175, P1450, DOI 10.1001/jamainternmed.2015.2152

Van Den Akker M, 1996, Eur J Gen Pract, V2, P65, DOI [10.3109/13814789609162146, DOI 10.3109/13814789609162146]

Ventura T, 2013, J AM MED DIR ASSOC, V14, DOI 10.1016/j.jamda.2013.05.006

NR 26

TC 1

Z9 1

U1 0

U2 2

PU WILEY

PI HOBOKEN

PA 111 RIVER ST, HOBOKEN 07030-5774, NJ USA

SN 1444-1586

EI 1447-0594

J9 GERIATR GERONTOL INT

JI Geriatr. Gerontol. Int.

PD MAR

PY 2018

VL 18

IS 3

BP 441

EP 447

DI 10.1111/ggi.13204

PG 7

WC Geriatrics & Gerontology; Gerontology

WE Science Citation Index Expanded (SCI-EXPANDED); Social Science Citation Index (SSCI)

SC Geriatrics & Gerontology

GA FZ4MO

UT WOS:000427566500010

PM 29214720

DA 2024-08-08

ER

PT J

AU Ahmed, NN

Farnie, M

Dyer, CB

AF Ahmed, Nasiya N.

Farnie, Mark

Dyer, Carmel B.

TI The Effect of Geriatric and Palliative Medicine Education on the

Knowledge and Attitudes of Internal Medicine Residents

SO JOURNAL OF THE AMERICAN GERIATRICS SOCIETY

LA English

DT Article

DE geriatric; education; resident training

ID SUBSPECIALTY HOUSE OFFICERS; NATIONAL-SURVEY; STUDENTS; CARE;

RECOMMENDATIONS; PROGRAMS; NEEDS

AB A recent Institute of Medicine report on geriatric work force issues recommends training residents in settings with geriatric patients and increasing certification requirements to include competence in the care of older adults. Although the number of internal medicine programs with a geriatric curriculum has increased, the scope and effectiveness of these programs vary. The purpose of this study was to evaluate the effect of a new academic geriatric and palliative medicine curriculum on the knowledge and attitudes of third-year internal medicine and fourth-year medicine and pediatrics residents. The study was conducted at The University of Texas Medical School at Houston. A new Division of Geriatric and Palliative medicine was created that offered inpatient, consultation, ambulatory, and home visit experiences in addition to didactic lectures. The University of Michigan Geriatrics Clinical Decision Making Assessment and the University of California at Los Angeles Geriatric Attitude Test was used to evaluate pre- and post-rotation knowledge and attitudes. Residents' knowledge improved after completing the rotation, as shown by a 6.9-point increase in posttest scores (P <.001). There was also a 10-point improvement in pretest scores over the course of the year (P=.03). Fifty-seven percent of residents had an improvement in attitude. This study shows that an increase in geriatric and palliative teaching opportunities provided by the establishment of a geriatric and palliative medicine division improves residents' knowledge significantly.

C1 [Ahmed, Nasiya N.; Dyer, Carmel B.] Univ Texas Hlth Sci Ctr, Div Geriatr & Palliat Med, Houston, TX 77030 USA.

[Farnie, Mark] Univ Texas Hlth Sci Ctr, Dept Internal Med, Houston, TX 77030 USA.

C3 University of Texas System; University of Texas Health Science Center

Houston; University of Texas System; University of Texas Health Science

Center Houston

RP Ahmed, NN (corresponding author), Univ Texas Hlth Sci Ctr, Div Geriatr & Palliat Med, 6431 Fannin,MSB 4-200, Houston, TX 77030 USA.

EM Nasiya.Ahmed@uth.tmc.edu

FU Geriatric Academic Career Award

FX There were no sponsors for the study. Dr. Ahmed is supported by a

Geriatric Academic Career Award.

CR *ACCR COUNC GRAD M, 2010, RES REV COMM COMM PR

*ADM AG, 2001, PROF OLD AM

[Anonymous], 2008, RET AG AM BUILD HEAL

Bragg EJ, 2005, ACAD MED, V80, P279, DOI 10.1097/00001888-200503000-00014

*CDC, 1999, NAT AMB MED CAR SURV

Chodosh J., 1999, GERONTOLOGY GERIATRI, V20, P19, DOI DOI 10.1300/J021v20n02_03

Drickamer MA, 2006, J GEN INTERN MED, V21, P1230, DOI 10.1111/j.1525-1497.2006.00585.x

Eleazer GP, 2000, ACAD MED, V75, P252

Fitzgerald JT, 2003, GERONTOLOGIST, V43, P849, DOI 10.1093/geront/43.6.849

GALE J, 1974, AGE AGEING, V49, P6

Kishimoto M, 2005, J AM GERIATR SOC, V53, P99, DOI 10.1111/j.1532-5415.2005.53018.x

Krain LP, 2007, J AM GERIATR SOC, V55, P2056, DOI 10.1111/j.1532-5415.2007.01475.x

Lindberg MC, 1996, J GEN INTERN MED, V11, P397, DOI 10.1007/BF02600185

Montagnini Marcos, 2004, J Palliat Med, V7, P652, DOI 10.1089/jpm.2004.7.652

Pan Cynthia X, 2002, Acad Med, V77, P936, DOI 10.1097/00001888-200209000-00042

Reuben D, 1998, J AM GERIATR SOC, V46, pS9

Tanner CE, 2006, J GEN INTERN MED, V21, P51, DOI 10.1111/j.1525-1497.2005.0281.x

Warshaw G.A., 2002, Geriatric medicine training and practice in the United States at the beginning of the 21st century

Warshaw GA, 2003, J GEN INTERN MED, V18, P679, DOI 10.1046/j.1525-1497.2003.20906.x

Warshaw GA, 2002, JAMA-J AM MED ASSOC, V288, P2313, DOI 10.1001/jama.288.18.2313

Warshaw GA, 2006, J AM GERIATR SOC, V54, P1603, DOI 10.1111/j.1532-5415.2006.00895.x

Williams BC, 2006, J GEN INTERN MED, V21, P490, DOI 10.1111/j.1525-1497.2006.00433.x

NR 22

TC 22

Z9 22

U1 0

U2 2

PU WILEY-BLACKWELL

PI MALDEN

PA COMMERCE PLACE, 350 MAIN ST, MALDEN 02148, MA USA

SN 0002-8614

J9 J AM GERIATR SOC

JI J. Am. Geriatr. Soc.

PD JAN

PY 2011

VL 59

IS 1

BP 143

EP 147

DI 10.1111/j.1532-5415.2010.03235.x

PG 5

WC Geriatrics & Gerontology; Gerontology

WE Science Citation Index Expanded (SCI-EXPANDED); Social Science Citation Index (SSCI)

SC Geriatrics & Gerontology

GA 706HG

UT WOS:000286208200022

PM 21226684

DA 2024-08-08

ER

PT J

AU Franchi, C

Nobili, A

Mari, D

Tettamanti, M

Djade, CD

Pasina, L

Salerno, F

Corrao, S

Marengoni, A

Iorio, A

Marcucci, M

Mannucci, PM

AF Franchi, Carlotta

Nobili, Alessandro

Mari, Daniela

Tettamanti, Mauro

Djade, Codjo D.

Pasina, Luca

Salerno, Francesco

Corrao, Salvatore

Marengoni, Alessandra

Iorio, Alfonso

Marcucci, Maura

Mannucci, Pier Mannuccio

CA REPOSI Investigators

TI Risk factors for hospital readmission of elderly patients

SO EUROPEAN JOURNAL OF INTERNAL MEDICINE

LA English

DT Article

DE Hospital readmission; Internal medicine and geriatric wards; Risk

factors; Elderly

ID VALIDATION; WARDS

AB Background: The aim of this study was to identify which factors were associated with a risk of hospital readmission within 3 months after discharge of a sample of elderly patients admitted to internal medicine and geriatric wards.

Methods: Of the 1178 patients aged 65 years or more and discharged from one of the 66 wards of the 'Registry Politerapie SIMI (REPOSI)' during 2010, 766 were followed up by phone interview 3 months after discharge and were included in this analysis. Univariate and multivariate logistic regression models were used to evaluate the association of several variables with rehospitalization within 3 months from discharge.

Results: Nineteen percent of patients were readmitted at least once within 3 months after discharge. By univariate analysis in-hospital clinical adverse events (AEs), a previous hospital admission, number of diagnoses and drugs, comorbidity and severity index (according to Cumulative Illness Rating Scale-CIRS), vascular and liver diseases with a level of impairment at discharge of 3 or more at CIRS were significantly associated with risk of readmission. Multivariate logistic regression analysis showed that only AEs during hospitalization, previous hospital admission, and vascular and liver diseases were significantly associated with the likelihood of readmission.

Conclusions: The results demonstrate the need for increased medical attention towards elderly patients discharged from hospital with characteristics such as AEs during the hospitalization, previous admission, vascular and liver diseases. (C) 2012 European Federation of Internal Medicine. Published by Elsevier B.V. All rights reserved.

C1 [Franchi, Carlotta] Mario Negri Inst Pharmacol Res, Lab Qual Assessment Geriatr Therapies & Serv, I-20156 Milan, Italy.

[Mari, Daniela] IRCCS Maggiore Hosp Fdn, Dipartimento Sci Clin & Comunita, Milan, Italy.

[Salerno, Francesco] Univ Milan, Policlin IRCCS San Donato, I-20122 Milan, Italy.

[Corrao, Salvatore] Univ Palermo, Dipartimento Biomed Med Interna & Specialist, I-90133 Palermo, Italy.

[Marengoni, Alessandra] Univ Brescia, Spedali Civili, Dept Med & Surg Sci, Geriatr Unit, I-25121 Brescia, Italy.

[Iorio, Alfonso; Marcucci, Maura] Univ Perugia, Dept Internal Med, I-06100 Perugia, Italy.

[Mannucci, Pier Mannuccio] IRCCS Maggiore Hosp Fdn, Sci Direct, Milan, Italy.

C3 Istituto di Ricerche Farmacologiche Mario Negri IRCCS; IRCCS Policlinico

San Donato; University of Milan; University of Palermo; Hospital Spedali

Civili Brescia; University of Brescia; University of Perugia

RP Franchi, C (corresponding author), Mario Negri Inst Pharmacol Res, Lab Qual Assessment Geriatr Therapies & Serv, Via La Masa 19, I-20156 Milan, Italy.

EM carlotta.franchi@marionegri.it

RI Perego, Francesca/K-8990-2016; Tettamanti, Mauro/U-8925-2017; Franchi,

Carlotta/AAB-9666-2019; De Vincentis, Antonio/AAB-7965-2019; Alessandro,

Nobili/AAA-7512-2020; serviddio, gaetano/C-7629-2011; PICARDI,

ANTONIO/C-2337-2012; Corrao, Salvatore/AAQ-5768-2020; Manfredini,

Roberto/A-3471-2008; Landolfi, Raffaele/AAL-4496-2020; FRANCHI,

CARLOTTA/AAB-8774-2019; Nobili, Alessandro/ABG-4516-2020; Rossi, Paolo

Dionigi/GRX-7747-2022; Rizzo, Maria Rosaria/AHH-3649-2022; Pasina,

Luca/AAB-1877-2020; Fanelli, Filippo Rossi/E-9587-2011; Iorio,

Alfonso/B-9478-2013; Ottonello, Luciano Carlo/I-3250-2018; Fabio,

Giovanna/M-7595-2015; Violi, Francesco/K-1509-2016; cicardi,

marco/K-9219-2016; Mari, Daniela/K-5663-2016; Basili,

Stefania/K-4024-2016

OI Perego, Francesca/0000-0003-3973-6931; Tettamanti,

Mauro/0000-0001-7345-0887; Franchi, Carlotta/0000-0001-6802-8710; De

Vincentis, Antonio/0000-0003-0220-0500; serviddio,

gaetano/0000-0002-6424-7841; PICARDI, ANTONIO/0000-0002-0230-218X;

Corrao, Salvatore/0000-0001-5621-1374; Manfredini,

Roberto/0000-0002-8364-2601; Landolfi, Raffaele/0000-0002-7913-8576;

FRANCHI, CARLOTTA/0000-0001-6802-8710; Nobili,

Alessandro/0000-0002-7813-0572; Rossi, Paolo

Dionigi/0000-0002-1202-5454; Rizzo, Maria Rosaria/0000-0002-1023-4260;

Pasina, Luca/0000-0002-4777-1350; Iorio, Alfonso/0000-0002-3331-8766;

Ottonello, Luciano Carlo/0000-0001-7431-3841; Fabio,

Giovanna/0000-0002-8805-2942; marengoni, alessandra/0000-0002-4851-1825;

maroni, lorenzo/0000-0001-5063-1941; Violi,

Francesco/0000-0002-6610-7068; Sasso, Ferdinando

Carlo/0000-0002-9142-7848; cicardi, marco/0000-0003-1251-225X; Mari,

Daniela/0000-0002-6950-0789; Motta, Irene/0000-0001-5701-599X; Basili,

Stefania/0000-0002-6987-1926; GUASTI, LUIGINA/0000-0002-4524-4864;

ZANETTI, MICHELA/0000-0002-2634-6363; salvatore,

teresa/0000-0002-6532-7122; Marcucci, Maura/0000-0002-8468-7991;

Persico, Marcello/0000-0002-1399-6498

CR Alarcón T, 1999, AGE AGEING, V28, P429, DOI 10.1093/ageing/28.5.429

Alonso Martinez J L, 2001, An Med Interna, V18, P248

BERNARDINI B, 1993, J AM GERIATR SOC, V41, P105, DOI 10.1111/j.1532-5415.1993.tb02041.x

Bisharat N, 2012, EUR J INTERN MED, V23, P457, DOI 10.1016/j.ejim.2012.03.004

Campbell SE, 2004, AGE AGEING, V33, P110, DOI 10.1093/ageing/afh036

Coleman EA, 2006, ARCH INTERN MED, V166, P1822, DOI 10.1001/archinte.166.17.1822

Cornette P, 2005, AGING CLIN EXP RES, V17, P322, DOI 10.1007/BF03324617

Di Iorio A, 1998, AGING CLIN EXP RES, V10, P339, DOI 10.1007/BF03339797

García-Pérez L, 2011, QJM-INT J MED, V104, P639, DOI 10.1093/qjmed/hcr070

HICKIE C, 1987, Clinical Gerontologist, V6, P51

Jencks SF, 2009, NEW ENGL J MED, V360, P1418, DOI 10.1056/NEJMsa0803563

Jones J, 1986, Health Serv J, V96, P825

Kalra AD, 2010, J GEN INTERN MED, V25, P930, DOI 10.1007/s11606-010-1370-5

Kansagara D, 2011, JAMA-J AM MED ASSOC, V306, P1688, DOI 10.1001/jama.2011.1515

KATZMAN R, 1983, AM J PSYCHIAT, V140, P734

Lanièce I, 2008, AGE AGEING, V37, P416, DOI 10.1093/ageing/afn093

Marengoni A., 2011, INT PSYCHOGERIATR, P1

Martín RB, 2000, REV CLIN ESP, V200, P252

Maurer PP, 2004, SWISS MED WKLY, V134, P606

Nobili A, 2011, EUR J CLIN PHARMACOL, V67, P507, DOI 10.1007/s00228-010-0977-0

PARMELEE PA, 1995, J AM GERIATR SOC, V43, P130, DOI 10.1111/j.1532-5415.1995.tb06377.x

SHAH S, 1989, J CLIN EPIDEMIOL, V42, P703, DOI 10.1016/0895-4356(89)90065-6

Silverstein Marc D, 2008, Proc (Bayl Univ Med Cent), V21, P363

Tavazzi L, 2006, EUR HEART J, V27, P1207, DOI 10.1093/eurheartj/ehi845

WILLIAMS EI, 1988, BRIT MED J, V297, P784, DOI 10.1136/bmj.297.6651.784

Zamir D, 2006, EUR J INTERN MED, V17, P20, DOI 10.1016/j.ejim.2005.10.004

Zanocchi M, 2006, AGING CLIN EXP RES, V18, P63

Zapatero A, 2012, EUR J INTERN MED, V23, P451, DOI 10.1016/j.ejim.2012.01.005

NR 28

TC 69

Z9 76

U1 0

U2 34

PU ELSEVIER

PI AMSTERDAM

PA RADARWEG 29, 1043 NX AMSTERDAM, NETHERLANDS

SN 0953-6205

EI 1879-0828

J9 EUR J INTERN MED

JI Eur. J. Intern. Med.

PD JAN

PY 2013

VL 24

IS 1

BP 45

EP 51

DI 10.1016/j.ejim.2012.10.005

PG 7

WC Medicine, General & Internal

WE Science Citation Index Expanded (SCI-EXPANDED)

SC General & Internal Medicine

GA 061EP

UT WOS:000312831000018

PM 23142413

OA Green Published

DA 2024-08-08

ER

PT J

AU Loewenthal, JV

Beltran, CP

Atalay, A

Schwartz, AW

Ramani, S

AF Loewenthal, Julia V.

Beltran, Christine P.

Atalay, Alev

Schwartz, Andrea Wershof

Ramani, Subha

TI "What's Going to Happen?": Internal Medicine Resident Experiences of

Uncertainty in the Care of Older Adults

SO JOURNAL OF GENERAL INTERNAL MEDICINE

LA English

DT Article; Early Access

DE geriatrics; graduate medical education; internal medicine; residency;

residents; uncertainty; age-friendly; elderly; older adults

ID ELDERLY-PATIENTS; GERIATRICS; EDUCATION; PROGRAMS; STRATEGIES;

PHYSICIANS; COMPLEXITY; TOLERANCE; BARRIERS; AGE

AB PurposeInternal medicine residents care for clinically complex older adults and may experience increased moral distress due to knowledge gaps, time constraints, and institutional barriers. We conducted a phenomenological study to explore residents' experiences and challenges through the lens of uncertainty.MethodsBetween January and March 2022, six focus groups were conducted comprising a total of 13 internal medicine residents in postgraduate years 2 and 3, who had completed a required 2-week geriatrics rotation. Applying the Beresford taxonomy of uncertainty as a conceptual model, data were analyzed using the framework method.ResultsAll challenging experiences described by residents caring for older adults were linked to uncertainty. Sources of uncertainty were categorized and mapped to the Beresford taxonomy: (1) lack of geriatrics knowledge or clinical guidelines (technical); (2) difficulty applying knowledge to complex older adults (conceptual); and (3) lack of longitudinal relationship with the older patient (personal). Residents identified capacity evaluation and discharge planning as two major geriatric knowledge areas linked with uncertainty. While the majority of residents reacted to uncertainty with some degree of distress, several reported positive coping strategies.ConclusionsInternal medicine residents face uncertainty when caring for older adults, particularly related to technical and conceptual factors. Strategies for mitigating uncertainty in the care of older adults are needed given links with moral distress and trainee well-being.

C1 [Loewenthal, Julia V.; Schwartz, Andrea Wershof] Brigham & Womens Hosp, Div Aging, Boston, MA 02115 USA.
[truncated: 5,273,486 more chars]
